# Supplementary material for: Epigenetic modulators link mitochondrial redox homeostasis to cardiac function in a sex-dependent manner
Source: Nat Commun. 2024 Mar 20;15:2358. doi: 10.1038/s41467-024-46384-8 (PMC10954618; doi:10.1038/s41467-024-46384-8)
Supplement: Supplementary file 1 — Supplementary Information [file 41467_2024_46384_MOESM1_ESM.pdf]

Supplementary Information

Epigenetic modulators link mitochondrial redox homeostasis to cardiac function in a sex-dependent manner

Zaher ElBeck[1,2*], Mohammad Bakhtiar Hossain[3], Humam Siga[1], Nikolay Oskolkov[4], Fredrik Karlsson[5], Julia Lindgren[6], Anna Walentinsson[7], Dominique Koppenhöfer[1], Rebecca Jarvis[8], Roland Bürli[8], Tanguy Jamier[8], Elske Franssen[8], Mike Firth[5], Andrea Degasperi[5,9], Claus Bendtsen[5], Robert I. Menzies[3], Katrin Streckfuss-Bömeke[10,11,12], Michael Kohlhaas[12], Alexander G. Nickel[12], Lars H. Lund[13], Christoph Maack[12], Ákos Végvári[14] and Christer Betsholtz[1,2]

[1]Department of Medicine Huddinge, Karolinska Institutet, Campus Flemingsberg, 141 57 Huddinge, Sweden. [2]Departmenty of Immunology, Genetics and Pathology, Rudbeck Laboratory, Uppsala University, Uppsala, Sweden. [3]Bioscience Renal, Research and Early Development, Cardiovascular, Renal and Metabolism (CVRM), BioPharmaceuticals R&D, AstraZeneca, Gothenburg, Sweden. [4]Department of Biology, National Bioinformatics Infrastructure Sweden, Science for Life Laboratory, Lund University, Sweden. [5]Data Sciences and Quantitative Biology, Discovery Sciences, R&D, AstraZeneca, Gothenburg, Sweden. [6] Translational Genomics, Centre for Genomics Research, Discovery Sciences, R&D, AstraZeneca, Gothenburg, Sweden. [7]Translational Science & Experimental Medicine, Research and Early Development, Cardiovascular, Renal and Metabolism (CVRM), BioPharmaceuticals R&D, AstraZeneca, Gothenburg, Sweden. [8]Neuroscience, BioPharmaceuticals R&D, AstraZeneca, Cambridge, United Kingdom. [9]Early Cancer Institute, University of Cambridge, Cambridge, United Kingdom. [10]Institute of Pharmacology and Toxicology, University of Würzburg, Germany [11]Clinic for Cardiology and Pneumology, Georg-August University Göttingen and DZHK (German Center for Cardiovascular Research), Partner Site Göttingen, Germany. [12]Department of Translational Research, Comprehensive Heart Failure Center (CHFC), University Clinic Würzburg, Würzburg, Germany. [13]Department of Medicine Karolinska Institutet, and Department of Cardiology, Karolinska University Hospital, Stockholm, Sweden. [14]Division of Chemistry I, Department of Medical Biochemistry & Biophysics, Karolinska Institutet, Sweden.

***Corresponding author:** zaher.elbeck@ki.se

This Supplementary Information file contains:

1- **Supplementary Figures 1-8**
2- **Supplementary Tables 1-7**

1- Supplementary figures 1-8:

Supplementary fig.1

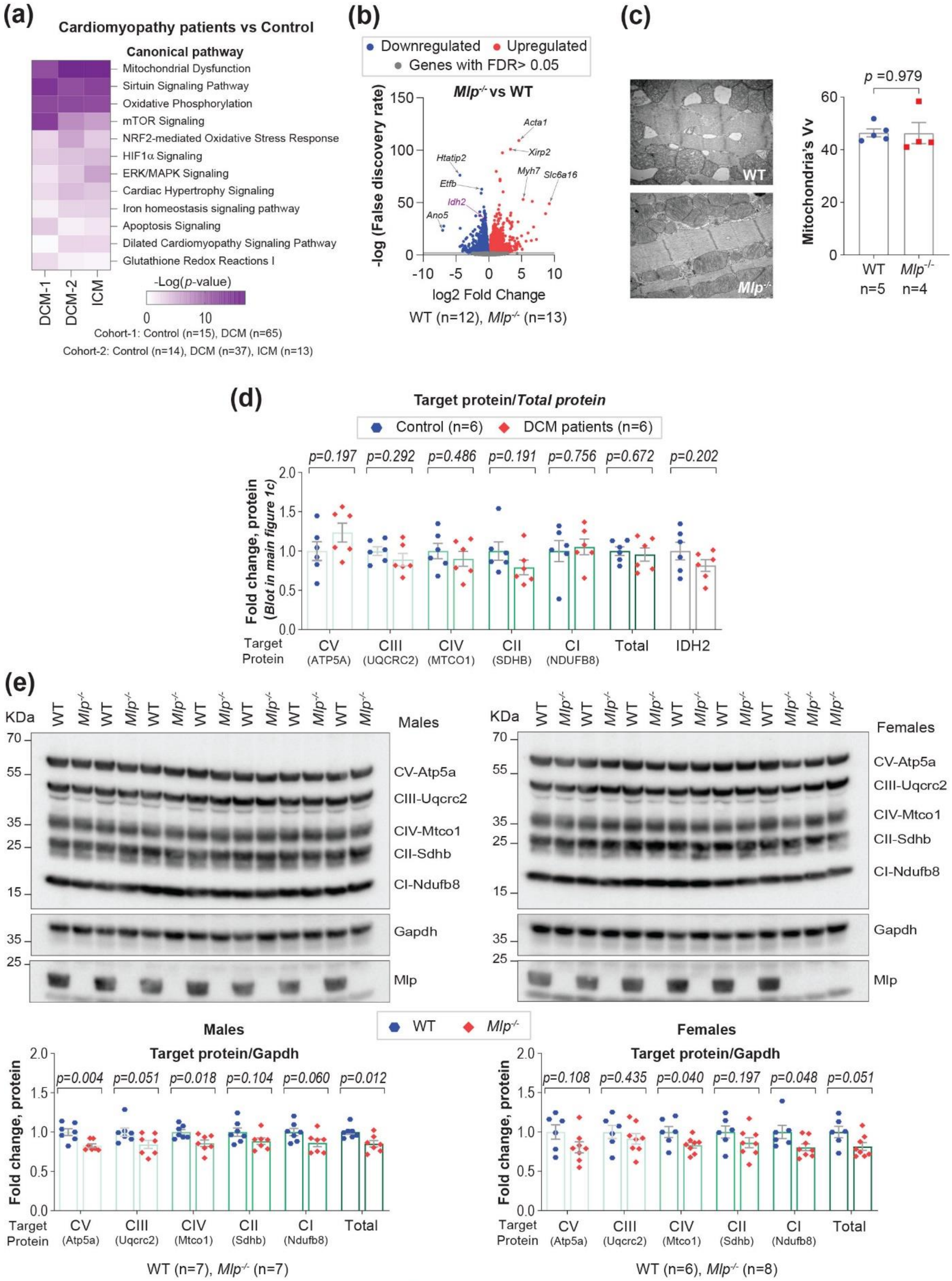

Supplementary fig.1 continues to the next page . . .

Continuation of Supplementary fig.1

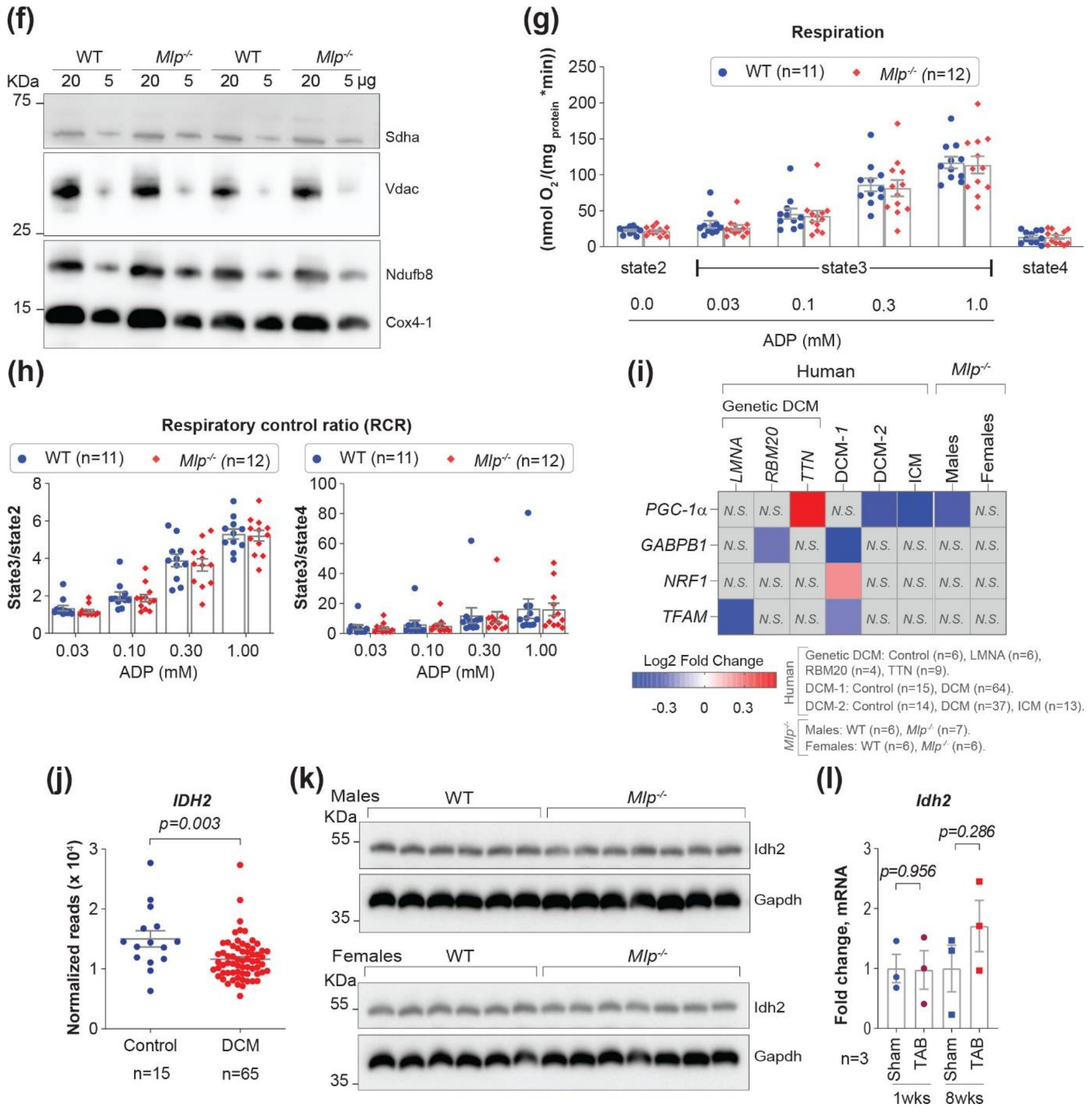

Supplementary Figure 1: Mitochondrial dysfunction and downregulation of IDH2 expression in association with eccentric hypertrophy

a) Selected pathways from IPA enrichment analysis on published transcriptomic data from cardiac tissue of patients with idiopathic dilated cardiomyopathy (DCM-1, cohort-1) [1] and (DCM-2, cohort-2) [2], as well as, ischemic cardiomyopathy (ICM, cohort-2) [2].

b) Volcano plot of transcriptomic data from left ventricles (LV) of male and female *Mlp*$^{-/-}$ mice. FDR: False discovery rate

c) Transmission electron microscopy images of the left ventricle (LV) of *Mlp*$^{-/-}$ mice and quantification of mitochondrial volume density (Vv). Wild type (WT) and *Mlp*$^{-/-}$ mice were from both sexes and of different ages.

d) Quantification of human mitochondrial electron transport chain and IDH2 expression by Western blotting. The blots are presented in Figure 1c.

e) Western blotting of OXPHOS complexes in the left ventricle (LV) of male and female *Mlp*$^{-/-}$ mice.

f) Western blotting of mitochondrial proteins (Vdac, Sdha, Ndufb8 and Cox4-1) in 5 and 20 µg of freshly isolated mitochondria from the LV of *Mlp*$^{-/-}$ mice.

g and h) Oxygen consumption and respiratory control ratio (RCR) in freshly isolated mitochondria from the LV of *Mlp*$^{-/-}$ mice.

i) Expression of genes involved in regulating mitochondrial biogenesis in the LV of patients with genetic DCM [3], idiopathic DCM (DCM 1 [1] and DCM 2 [2]), ICM [2], and, male/female *Mlp*$^{-/-}$ mice, analyzed by RNA sequencing. N.S. indicates differentially expressed genes that are not statistically significant, i.e., FDR>0.05. A complete list of differentially expressed genes is provided in Supplementary Data file 3.

j) *IDH2* expression in the LV of patients with eccentric DCM extracted from a public repository [1].

k) Western blotting of Idh2 in the LV of male and female *Mlp*$^{-/-}$ mice (the quantification shown in Figure 1h is the merge of males and females normalized bands' intensities of these two blots).

l) *Idh2* expression in animals with transaortic banding (TAB). Data, extracted from a public repository [4], show mRNA fold change 1- and 8-weeks after banding.

Source data and uncropped blots are available in Source Data file. Bars represent mean ± SEM, analyzed with unpaired two-tailed t-test. Sample size is indicated on all panels.

Supplementary fig. 2

(a)

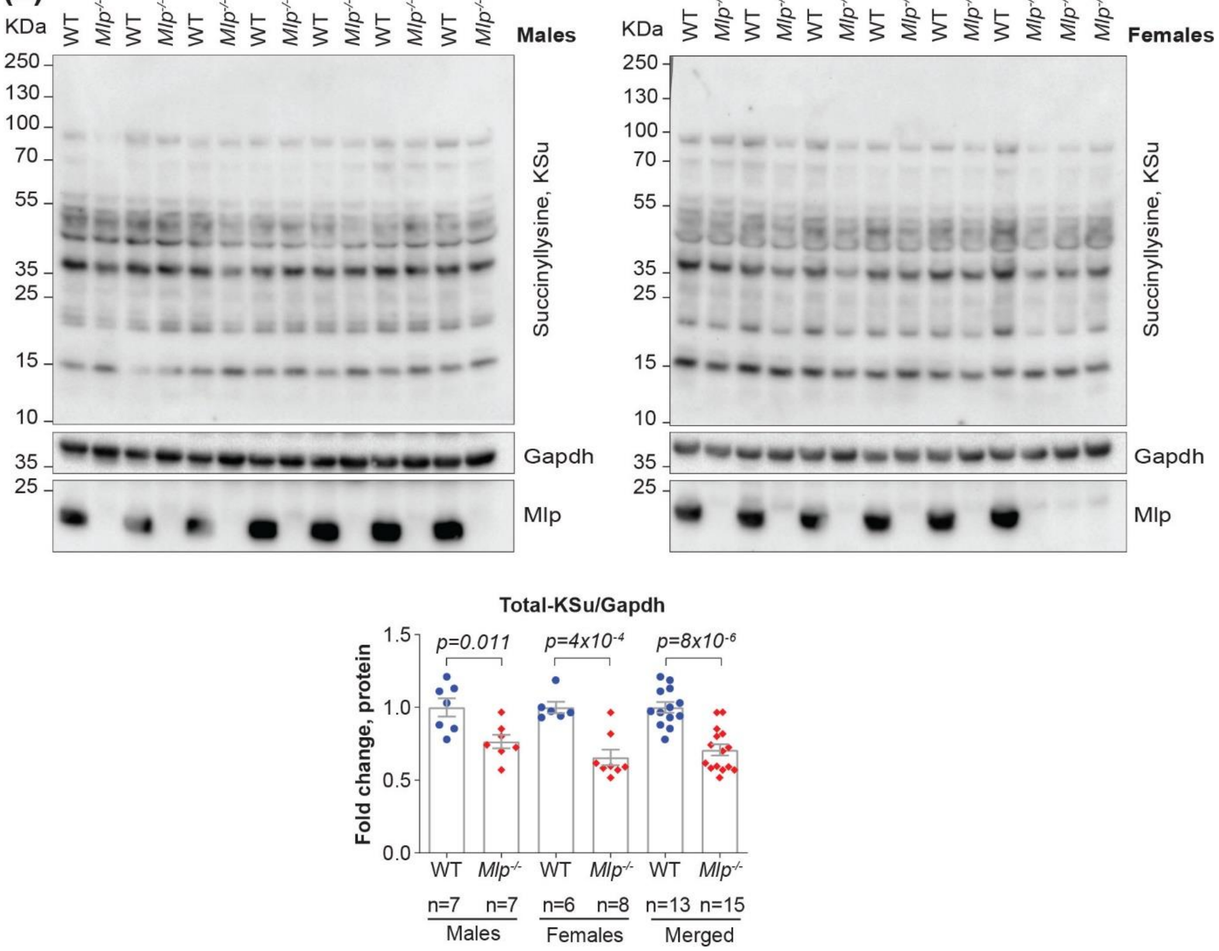

Supplementary Figure 2: Desuccinylation of proteins in the LV of male and female *Mlp*$^{-/}$ mice.

a) Western blotting of succinylated proteins in the LV of male and female *Mlp*$^{-/-}$ mice. Total band intensities were quantified and normalized to Gapdh. Bars represent mean ±SEM, analyzed with unpaired two-tailed *t*-test. Source data and uncropped blots are available in Source Data file.

Supplementary fig.3

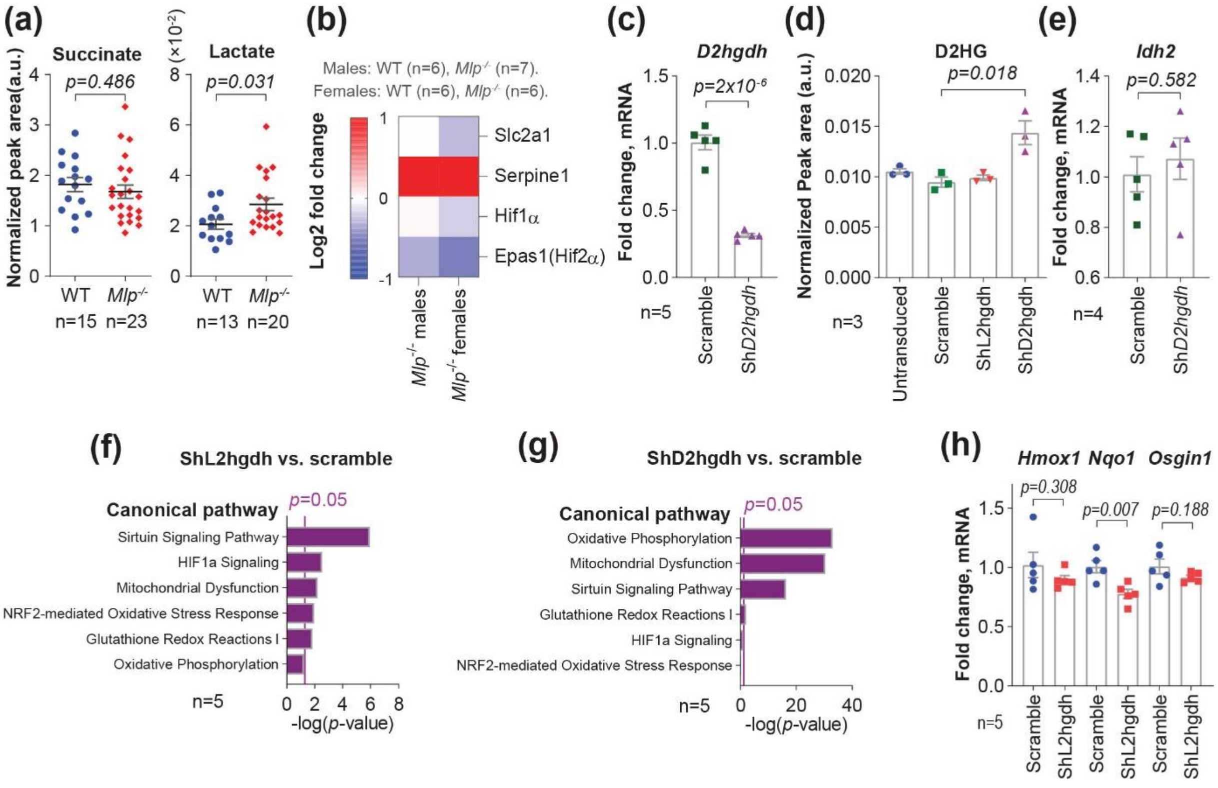

Supplementary Figure 3: Antioxidative role of L2HG in the heart

a) Targeted LC-MS/MS analysis of succinate and lactate levels in the LV of *Mlp*$^{-/-}$ mice, normalized to tissue weight. Horizontal lines represent mean ±SEM, analyzed with unpaired two-tailed *t*-test.

b) Expression of genes regulating hypoxia in the LV of *Mlp*$^{-/-}$ mice analyzed by RNA sequencing. Complete list of differentially expressed genes in the LV of *Mlp*$^{-/-}$ is provided in Supplementary Data file 3.

c-e) Transduced NRCMs with ShRNA targeting *D2hgdh* were analyzed for the expression of *D2hgdh* and *Idh2* by qPCR in panels **(c and e),** and for D2HG level by a targeted LC-MS in panel **(d).** Bars represent mean ±SEM, analyzed with unpaired two-tailed *t*-test.

f and g) Selected pathways from IPA enrichment analysis on transcriptomic data from NRCMs transduced with ShRNA targeting *L2hgdh* or *D2hgdh*.

h) qPCR analysis of *Hmox1*, *Nqo1* and *Osgin1* expression in NRCMs transduced with ShRNA targeting *L2hgdh*. Bars represent mean ±SEM, analyzed with unpaired two-tailed *t*-test.

Sample size n is indicated on all panels. Source data are available in Source Data file.

Supplementary fig.4

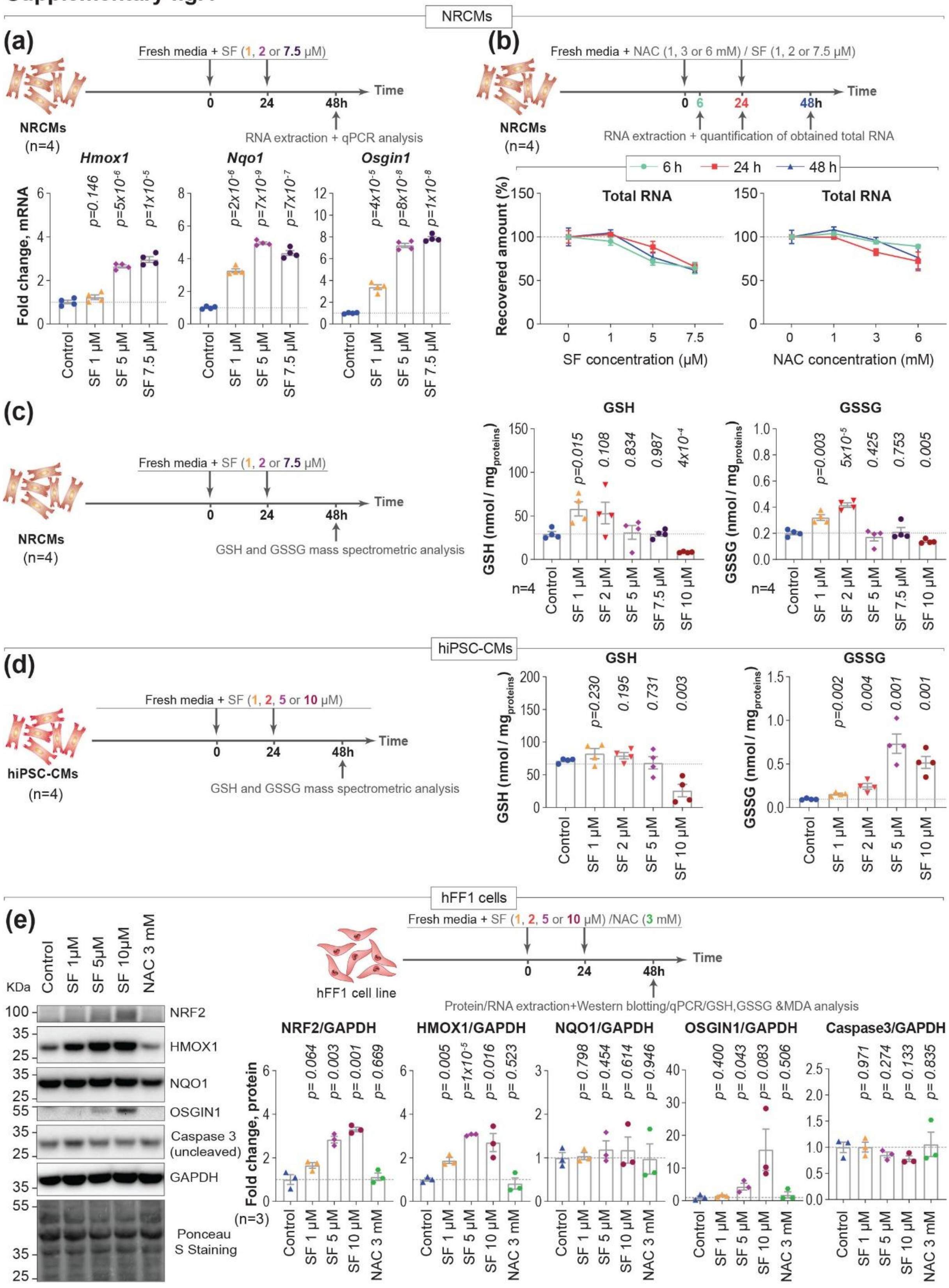

Supplementary fig. 4 continues to the next page . . .

Continuation of Supplementary fig. 4

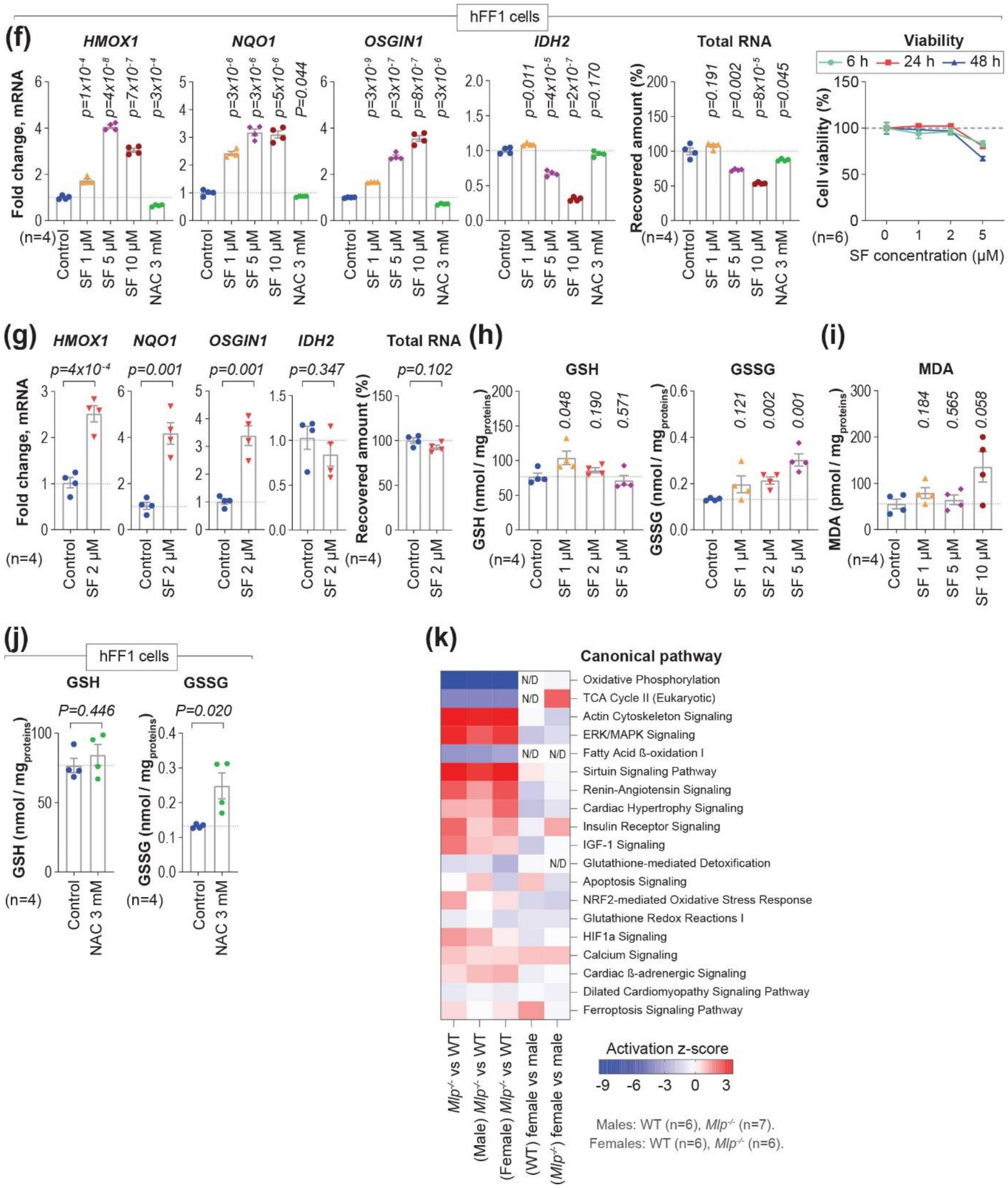

Supplementary Figure 4: Mutual redox regulation downregulates *IDH2*

a) qPCR analysis of *Hmox1*, *Nqo1* and *Osgin1* expression in NRCMs treated with increasing doses of sulforaphane (SF), normalized to untreated control.

b) Recovered amounts of extracted RNA from each well of cultured NRCMs treated with increasing doses of SF or N-acetylcysteine (NAC).

c and d) Targeted LC-MS/MS analysis of GSH and GSSG levels in NRCMs (in panel **c**), and in human cardiomyocytes derived from induced pluripotent stem cells (hiPSC-CMs) (in panel **d**) treated with increasing concentrations of SF for 48 h, compared to untreated control.

e) Western blotting of NRF2, HMOX1, NQO1, OSGIN1 and Caspase 3 in hFF1 cells treated with SF or NAC. Bands' intensities of target proteins were normalized to GAPDH. The migratory molecular weight of NRF2 differs from its real molecular weight.

NRF2 has the molecular weight of ~55–65 KDa, but it runs on the SDS gel at ~95–110 kDa, most likely due to its abundant acidic residues [5].

f and g) qPCR analysis of *HMOX1*, *NQO1*, *OSGIN1* and *IDH2* expression, recovered amounts of extracted total RNA, and the percentage of cell viability in treated hFF1 cells normalized to untreated control.

h, i and j) Targeted LC-MS/MS analysis of GSH, GSSG and MDA levels in hFF1 cells treated with increasing concentrations of SF for 48 h (in panels **h and i**), or with 3mM of NAC for 48 hours in panel (**j**) compared to controls. The control group used in panel (**j**) is the same as the one presented in (**h**), as cells were treated with NAC or SF in the same plate and processed together.

k) Selected pathways from IPA comparison analysis on transcriptomic data from LV of male and female *Mlp*$^{-/-}$ mice.

The bar plots of the NRCMs, the hiPSC-CMs and the hFF1 cells in panels (**c, d, h**) are for the same data depicted in main Figures 4d, e, but with *p*-values included and individual replicates visualized.

Sample size n is indicated on all panels. Bars in all panels represent mean ±SEM, analyzed with unpaired two-tailed *t*-test. Source data and uncropped blots are available in Source Data file.

Supplementary fig.5

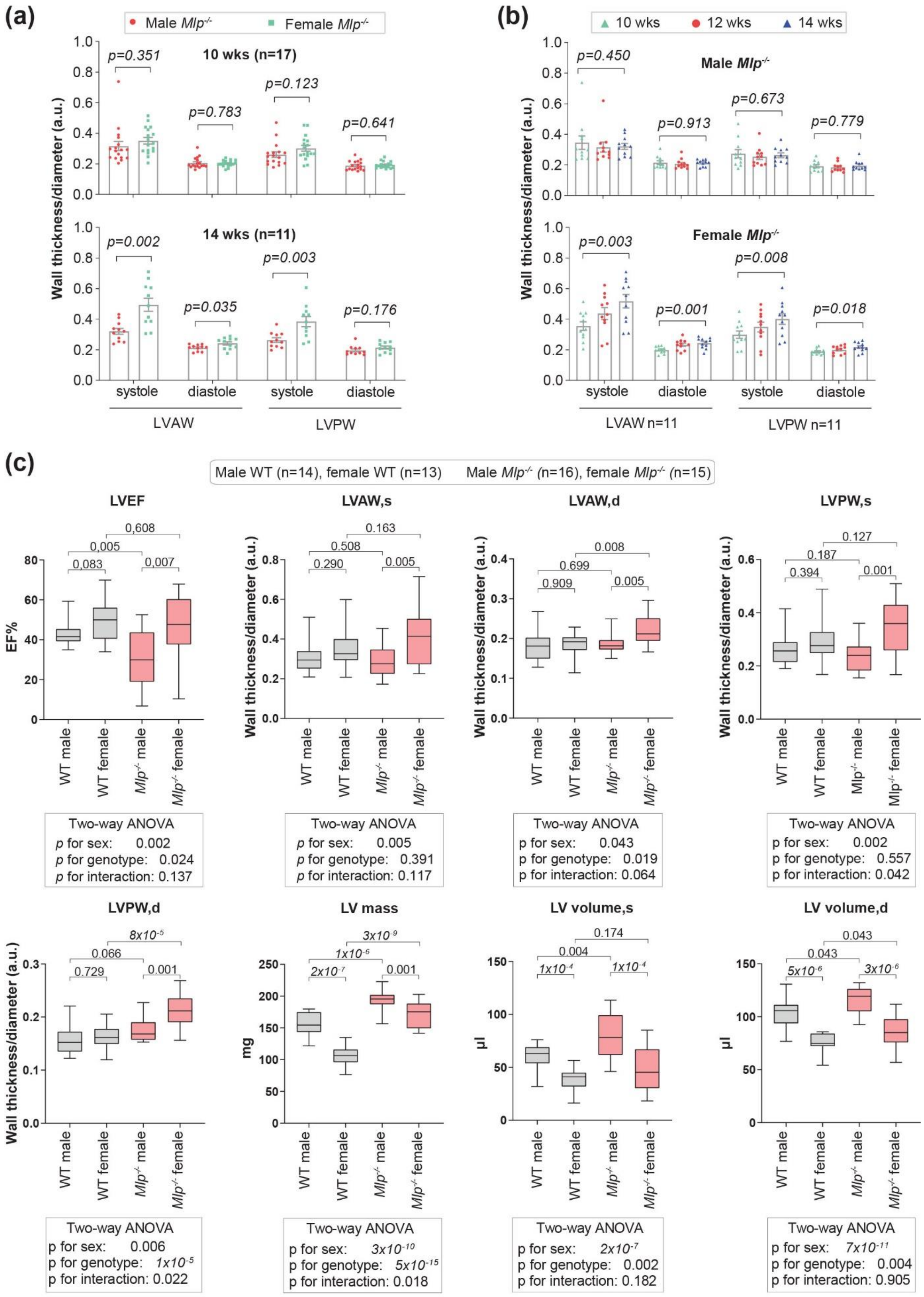

Supplementary fig. 5 continues to the next page . . .

Continuation of Supplementary fig. 5

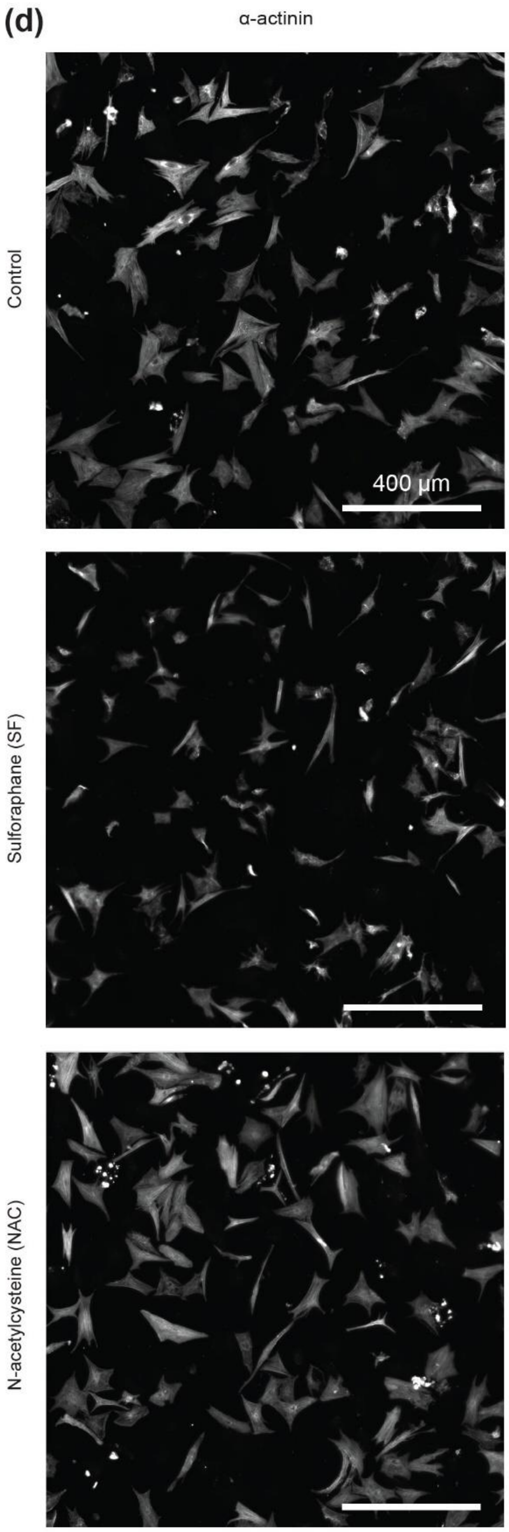

Supplementary Figure 5: Redox associated sex dimorphism in cardiac phenotype

a) Thickness of LV anterior wall (LVAW) and posterior wall (LVPW), normalized to LV diameter, in male and female *Mlp*$^{-/-}$ mice at the age of 10 and 14 weeks. Values represent mean ±SEM, analyzed with unpaired two-tailed *t*-test. The values in the lower figure of this panel (14 weeks) are the same values utilized in Figure 5b. It was plotted here again for easy comparison with upper panel (10 weeks), and to visualize the differences in wall thickness between systole and diastole.

b) Changes over age in the thickness of LV wall in males and females, normalized to LV diameter. Bars represent mean ±SEM, analyzed with paired *t*-test.

c) The effect of sex and genotype on certain cardiac parameters in *Mlp*$^{-/-}$ and their littermate WT control (2-way ANOVA). The *p*-values displayed on the plots represent unpaired two-tailed *t*-test, while the *p*-values in the boxes beneath the plots represent the effect and interaction of the 2-way ANOVA analysis. LV(A/P)W(s/d) refers to the thickness of the left ventricle anterior wall (LVAW) or posterior wall (LVPW) in systole (s) and diastole (d), respectively, normalized to LV diameter. The age of the animals in this figure was between 10-19 weeks with most of them at 12-13 weeks of age. Sample size is indicated on all panels.

Source data for panels (**a, b,** and **c**) are available in Source Data file.

d) The effects of SF and NAC treatment on NRCM cell-shape. NRCMs were stained for α-actinin and imaged with a bright field fluorescent microscope. These images were used for the quantifications of cell surface area and the minimum caliper diameter with ImageJ shown in Figure 5e.

Supplementary fig. 6

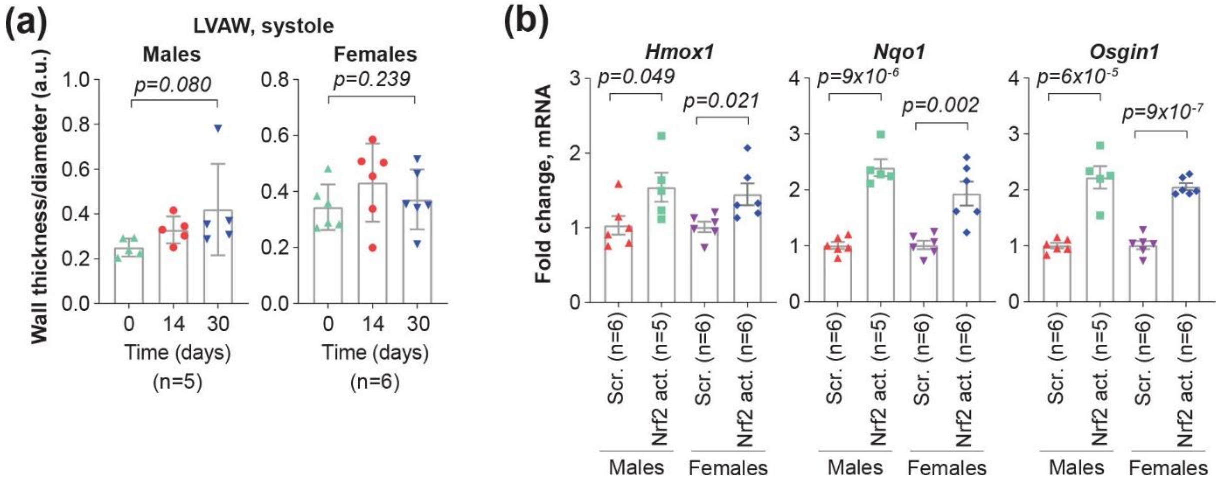

Supplementary Figure 6: Selective improvements in cardiac phenotype by antioxidative treatment *in vivo*

a) Changes over time in the thickness of the LV anterior wall (LVAW) in systole, normalized to left ventricle diameter, in male and female *Mlp*$^{-/-}$ mice during treatment with Nrf2 activator. Bars represent mean ± SEM with paired one-tailed *t*-test.

b) qPCR analysis of *Hmox1*, *Nqo1*, *Osgin1* expression in the LV of *Mlp*$^{-/-}$ mice treated with Nrf2 activator, normalized to the scrambled control group. Bars represent mean ± SEM, analyzed with unpaired two-tailed *t*-test.

Sample size is indicated on all panels. Source data are available in Source Data file.

Supplementary fig. 7

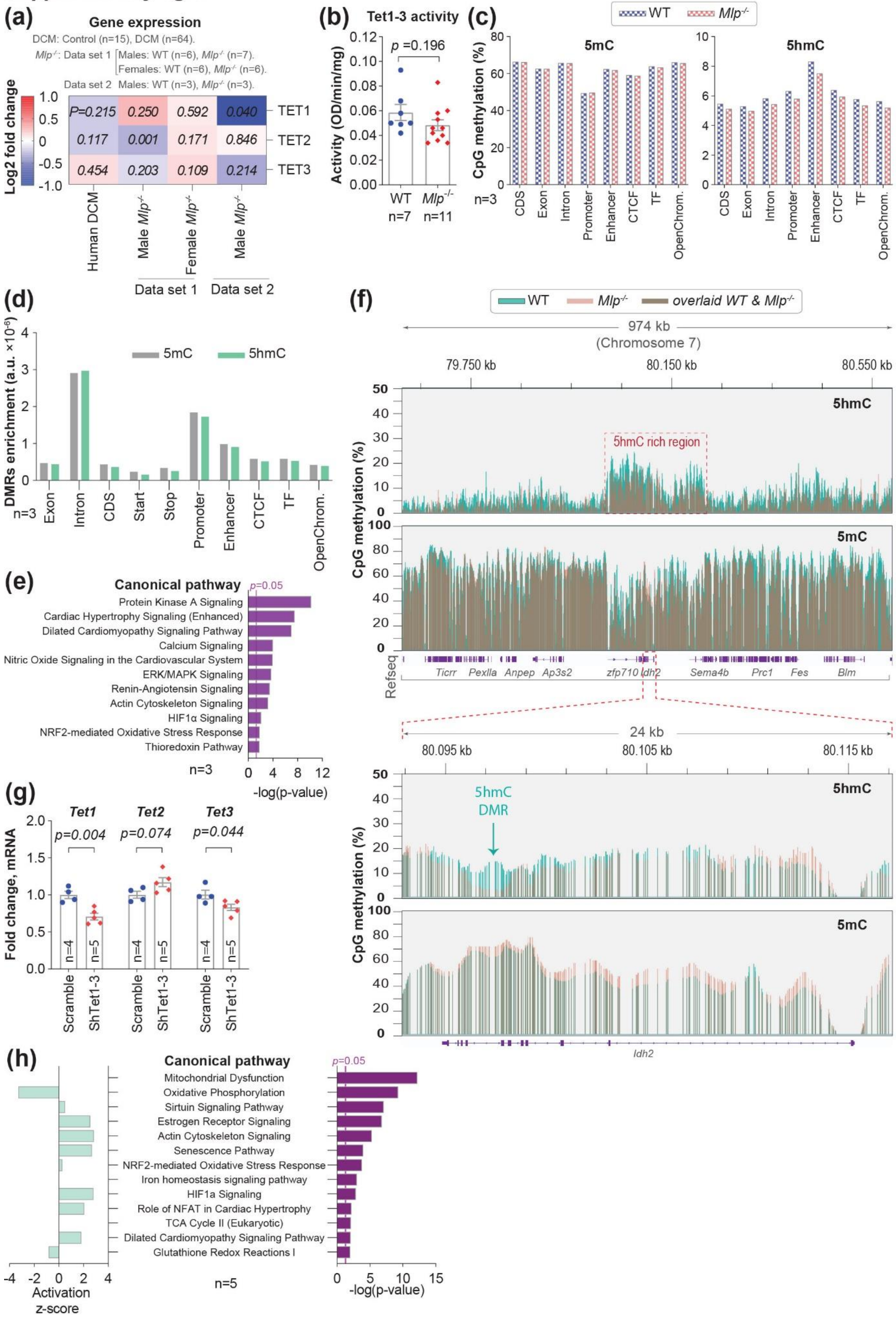

Supplementary Figure 7: Unique epigenetic control of *Idh2* expression and redox response.

a) Expression of *TET1,2,3* genes in the LV of patients with DCM [1] and in male and female *Mlp*$^{-/-}$ mice analyzed by RNA sequencing. Data set 1 of *Mlp*$^{-/-}$ mice were produced for this work, and the complete list of differentially expressed genes is provided in

Supplementary Data file 3, whereas Data set 2 of *Mlp*$^{-/-}$ mice was analyzed from the publicly available raw sequencing data on *Mlp*$^{-/-}$ hearts (SRA accession ID: PRJNA327790). These data are from same animals of which whole-genome oxidative bisulfite (OxBS) and bisulfite (BS) raw sequencing data was generated, which is also publicly available under same accession ID on SRA.

b) The activity of Tet1-3 enzymes in the LV of male *Mlp*$^{-/-}$ mice, at 12 weeks of the age. Bars represent mean ±SEM, analyzed with unpaired two-tailed *t*-test.

c) Percentages of whole genome 5mC or 5hmC-methylated CpGs within each functional genetic region in the myocardium of *Mlp*$^{-/-}$ mice, n=3. (CDS: Coding regions, CTCF: Transcriptional repressor CTCF binding sites, TF: transcription factors binding sites).

d) Intersection over Union metric to visualize the enrichment of DMRs with respect to their regulatory features, in the LV of *Mlp*$^{-/-}$ mice, n=3.

e) Selected pathways from IPA analysis on genes that harbored DMRs for 5hmC located in introns, in the LV of *Mlp*$^{-/-}$ mice.

f) Screenshots from the Integrative Genomic Viewer for genomic 5hmC rich region harboring *Idh2* locus in chromosome 7 of the murine genome (n=3).

g) qPCR analysis of *Tet1-3* expression in NRCMs transduced with ShTet1-3. Bars represent mean ±SEM, analyzed with unpaired two-tailed *t*-test. The sample size n represents individual wells, and it is indicated on the figures.

h) Selected pathways from IPA enrichment analysis on transcriptomic data from NRCMs transduced with ShRNAs against Tet1-3.

Sample size is indicated on all panels. Source data are available in Source Data file.

Supplementary fig. 8

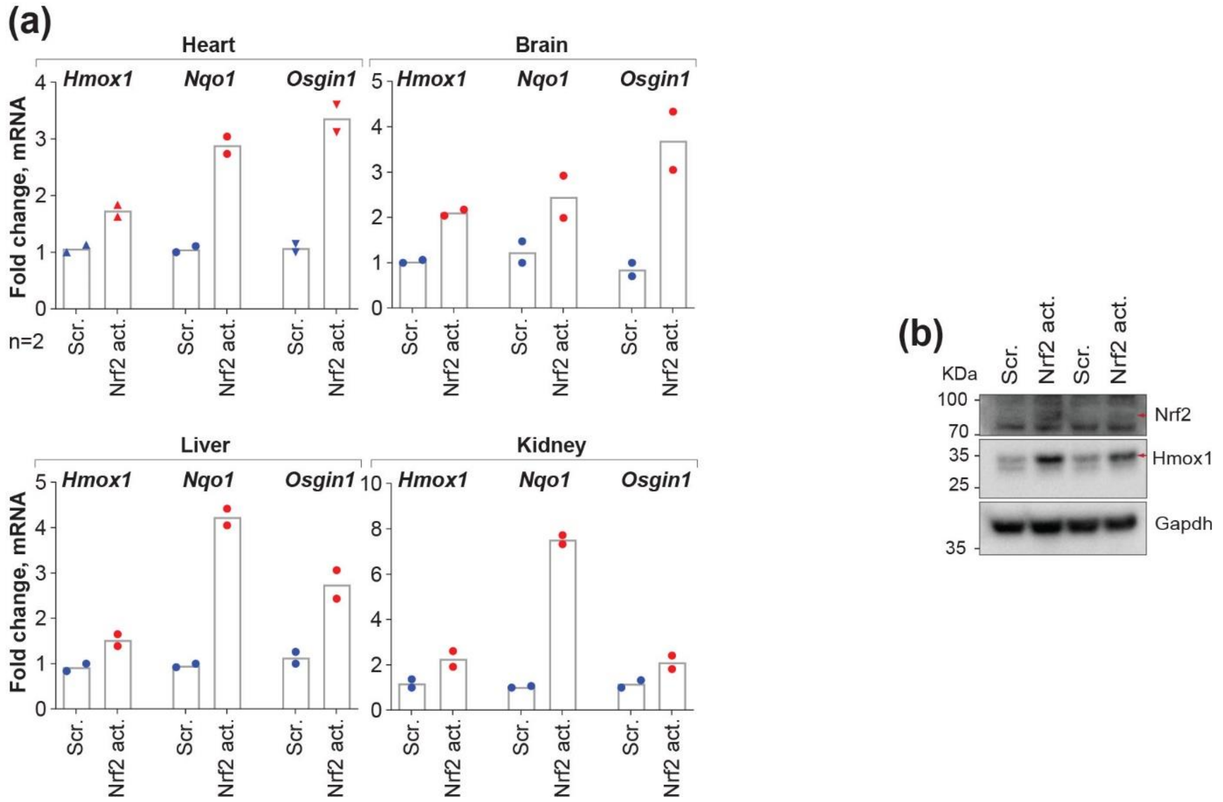

Supplementary Fig. 8: Pilot *in vivo* study with Keap1 inhibitor (AZ925)

a) qPCR analysis of *Hmox1*, *Nqo1*, *Osgin1* levels in heart, brain, liver and kidney tissues, 9 hours post-administration of a single dose of 10 mg/kg of AZ925 or scrambled control by oral gavage. This pilot experiment was conducted in accordance with the PREPARE guidelines (Smith et al., 2018) that preceded actual *in vivo* experiment presented in main Figure 6. The aim of this experiment was to verify whether KEAP1 inhibitor would induce the expression of Nrf2-downstream target genes (single-dose, 10 mg/kg, 9 hours post-administration) in the heart, similar to what had been observed in the brain during developing the compound. Sample size is indicated on the figures.

b) Western blotting of Nrf2, Hmox1 and Gapdh in LV from mice described in panel (**a**).

Source data and uncropped blots are available in Source Data file.

2- Supplementary Tables 1- 7:

Supplementary Table 1: Characteristic of human samples utilized in figure 1C, and provided by Sydney Heart Bank

Lane in fig. 1c	sex	Age	Mutated Gene	General Clinical notes	References
1	Male	20-25	TTN or OBSCN	LVEF ≤ 25%, with normal coronaries	[6,7]
3	Female	20-25			[7]
5	Male	40-45			[6,7]
7	Female	60-65			[7]
9	Male	20-25			[7]
11	Male	35-40			[7]
2	Male	35-40	Controls	Massive posterior basilar artery infarct, hypoxic brain injury, CoD L middle cerebral artery bleed and aneurism, subarachnoid haemorrhage or spontaneous intracranial haemorrhage. No observable coronary disease	[8-10]
4	Male	20-25			[7,11,12]
6	Male	40-45			
8	Female	40-45			[12]
10	Female	40-45			
12	Male	45-50			[11]

Supplementary Table 2: Sequences and source links of ShRNAs utilized in the current work

Construct ID	Target gene	ShRNA sequences	Map	References
**1038dup**	Tet1-3	Tet1: GGAGGGATTTCTCACGTTA<br>Tert2 GGATGTAAGTTTGCCAGAAGC<br>Tet3: GCTCCAACGAGAAGCTATTTG	https://en.vectorbuilder.com/vector/VB191127-1038dup.html	[13]
**1073xvx**	Tet1-3	Tet1: GCTCATGGAGACTAGGTATGG<br>Tet2: CTCAGGGATGTCCTATTGCTAAA<br>Tet3: AAGCGCAACCTATTCTTGGAA	https://en.vectorbuilder.com/vector/VB191127-1073xvx.html	[13,14]
**1186ueu**	L2hgdh	AGTAAGGATGGGATGAAATAT,<br>GGAGTCACTGAAAGCTAAATT	https://en.vectorbuilder.com/vector/VB200603-1186ueu.html	-
**1008zgf**	D2hgdh	ACGTGTTCAAGTATGACTTAT,<br>TTGGTGCCTTGGAGCCTTATG,<br>CTGAACTGCCTGACCTCTTTG	https://en.vectorbuilder.com/vector/VB200529-1008zgf.html	-
**1020znr**	Scramble	CCTAAGGTTAAGTCGCCCTCG	https://www.vectorbuilder.kr/vector/VB180117-1020znr.html	-

Supplementary Table 3: List of inclusion and fragment mass

**Compound Name**	**Formula**	**Adduct**	***m/z***	**z**	**t (min)**		**HCD Collision Energy (%)**	**Fragment 1**		**Fragment 2**	
					**start**	**stop**		***m/z***	**Formula**	***m/z***	**Formula**
**Lactic acid**	$C_3H_6O_3$	-H	89.0244	1	0	30	27	71.0139	$C_3H_3O_2$		
**SA**	$C_4H_6O_4$	-H	117.0193	1	2.53	4.53	27	73.0295	$C_3H_5O_2$	99.0088	$C_4H_3O_3$
**2HG-TSPC**	$C_{21}H_{23}NO_8S$	-H	448.1072	1	16.23	19.23	27	318.0806	$C_{16}H_{16}NO_4S$	155.0172	$C_7H_7O_2S$
**2HG-TSPC_heavy**	$C_{21}H_{20}D_3NO_8S$	-H	451.126	1	16.23	19.23	27	318.0806	$C_{16}H_{16}NO_4S$	155.0172	$C_7H_7O_2S$
**MA-TSPC**	$C_{20}H_{21}NO_8S$	-H	434.0915	1	19.16	21.16	27	318.0806	$C_{16}H_{16}NO_4S$	155.0172	$C_7H_7O_2S$
**GSH-NEM**	$C_{16}H_{24}N_4O_8S$	+H	433.1388	1	2.4	2.9	30	201.0692	$C_8H_{12}N_2O_2S$	358.1279	$C_{11}H_{23}N_3O_8S$
**GSH_D5-NEM**	$C_{16}H_{19}D_5N_4O_8S$	+H	438.1701	1	2.4	2.9	30	201.0692	$C_8H_{12}N_2O_2S$	363.1383	$C_{11}H_{18}D_5N_3O_8S$
**GSSG**	$C_{20}H_{32}N_6O_{12}S_2$	+H	613.1592	1	1.34	1.87	30	355.0741	$C_{10}H_{18}N_4O_6S_2$	231.0434	$C_8H_{10}N_2O_4S$
**GSSG ($^{[13]}C_4{}^{[15]}N_2$)**	$C_{16}{}^{[13]}C_4H_{32}N_4{}^{[15]}N_2O_{12}S_2$	+H	619.1667	1	1.34	1.87	30	361.0815	$C_7{}^{[13]}C_4H_{23}N_2{}^{[15]}N_2O_8S$		
**MDA-DNPH**	$C_9H_6N_4O_4$	+H	235.0462	1	8.3	8.5	30	189.0533	$C_9H_6N_3O_2$		
**MDA-D2-DNPH**	$C_9H_4D_2N_4O_4$	+H	237.0587	1	8.3	8.5	30	191.0658	$C_9H_4D_2N_3O_2$	161.0526	$C_5H_4D_2N_2O_4$

Supplementary Table 4: Primary and secondary antibodies utilized in the study.

Antibody	Reference number	Provider	dilution	Incubation
**OxPhos cocktail**	45-8099	Invitrogen	1:500	Overnight ,4ºC /2.h, RT*
**IDH2**	MA5-17271	Invitrogen	1:1000	Overnight ,4ºC /2.h, RT*
**GAPDH**	MA5-15738	Invitrogen	1:2500	Overnight ,4ºC /2.h, RT*
**MLP/CSRP3**	ab155538	Abcam	1:2000	Overnight, 4ºC
**Succinyllysine**	PTM-401	PTM Biolabs	1:500	Overnight, 4ºC
**L2HGDH**	15707-1-AP	Proteintech	1:200	2 h, RT
**β-actin**	A5441	Sigma-Aldrich	1:5000	Overnight, 4ºC
**SDHA**	5839	Cell Signalling	1:1000	Overnight, 4ºC
**HMOX1**	10701-1-AP	Proteintech	1:500	Overnight, 4ºC
**NQO1**	11451-1-AP	Proteintech	1:500	Overnight, 4ºC
**OSGIN1**	15248-1-AP	Proteintech	1:500	2 h, RT
**NRF2**	16396-1-AP	Proteintech	1:1500	2 h, RT
**Caspase 3**	9662S	Cell Signalling	1:1000	Overnight, 4ºC
**Vdac**	#1515	Gifted, self-made[15]		Overnight, 4ºC
**Ndufb8**	#3764	Gifted, self-made[15]		Overnight, 4ºC
**Cox4-1**	#1522	Gifted, self-made[15]		Overnight, 4ºC
**Secondary anti-rabbit IgG**	NA934	Sigma-Aldrich	1:5000	1 h, RT
**Secondary anti-mouse IgG**	A9044	Sigma-Aldrich	1:10 000	1 h, RT
**α-actinin**	A7811	Sigma-Aldrich	1:1000	Overnight, 4oC
**Goat anti-mouse, Alexa Fluor 568-conjugated antibody**	ab175473	Abcam	1:1000	1 h, RT

*The incubation time is 2h at RT for the human samples, and overnight at 4ºC for the murine samples

Supplementary Table 5: qPCR primers utilized in this study

Gene	Forward primer	Reverse primer	Species
IDH2	GCGAAGCCCGTGGTGGAGAT	AGTCTGGTCACGGTTTGGGA	Human
HMOX1	CCAGGCAGAGAATGCTGAGTTC	AAGACTGGGCTCTCCTTGTTGC	Human
NQO1	GAAGAGCACTGATCGTACTGGC	GGATACTGAAAGTTCGCAGGG	Human
OSGIN1	CCCGGTCATCATTGTGGGTAA	GCTTCGTGTAGGGTGTGTAGC	Human
GAPDH	ATGACATCAAGAAGGTGGTG	CATACCAGGAAATGAGCTTG	Human
Idh2	GGAGAAGCCGGTAGTGGAGAT	GGTCTGGTCACGGTTTGGAA	Mouse & rat
Hmox1	CACGCATATACCCGCTACCT	CCAGAGTGTTCATTCGAGCA	mouse
Nqo1	AGGATGGGAGGTACTCGAATC	AGGCGTCCTTCCTTATATGCTA	mouse
Osgin1	CCTCCGGTATCTGCCTGTC	GGAAAGGTACTCTAGGTCCTGG	mouse
Gapdh	GGGTGTGAACCACGAGAAAT	GTCTTCTGGGTGGCAGTGAT	mouse
Hmox1	GAAGAAGATTGCGCAGAAG	GAAGGCGGTCTTAGCCTCTT	Rat
Nqo1	CTCGCCTCATGCGTTTTTG	CCCCTAATCTGACCTCGTTCAT	Rat
Osgin1	CCTCGGGTATCTGTCTATC	GGAAAGGTACTCTAGGTCCTGA	Rat
L2hgdh	TAGTCATCGTTGGTGGTGGAA	TCCAGTCTGGTGAAGAGCCAAAT	Rat
D2hgdh	GGCTGCCGTTTTCTACCGTGT	GCAGTGCCTAAGAATCTGGGAG	Rat
Tet1	TCCTCAACCCGAGGATGGTA	CTCTTTCCGGGCACACTCAA	Rat
Tet2	TGGTGCTTATGTTCCGTGCT	CAACACCCAGCTCTGTAGGG	Rat
Tet3	CTACATGCCACCACACCACTC	CTGGTTGAGGTTCTTGTGCTG	Rat
Gapdh	GACATGCCGCCTGGAGAAAC	AGCCCAGGATGCCCTTTAGT	Rat

Supplementary Table 6: Reagents used in this study

Material	Notes	Manufacturer	Reference ID
DMEM	DMEM, GlutaMax, 4.5g/L LD-glucose, pyruvate	Gibco	31966-021
Medium 199	Contains Eale's salts and L-glutamine	Gibco	31150-022
Horse serum		SigmaAldrich	H1138
HEPES	1M	ThermoFisherScientific	15630-080
Penicillin/streptomycin	100X	ThermoFisherScientific	15140-122
Cytarabine		SigmaAldrich	C1768
DL-Sulforaphane		SigmaAldrich	S4441
Gelatin	Gelatin from porcine skin	SigmaAldrich	G1890
RIPA buffer		Substratenheten Huddinge	MIK2933-1000
Halt™ Protease and Phosphatase Inhibitor Cocktail	100 X	Thermo Scientific	78440
Stainless Steel Beads	5 mm	Qiagen	69989
NP40 Lysis Buffer		Invitrogen/Thermo Fisher Scientific	FNN0021
Pierce™ BCA Protein Assay Kit		Thermo Fisher Scientific	23227
NuPAGE™ LDS Sample Buffer	4X	Invitrogen/ Thermo Fisher Scientific	NP0007
NuPAGE Sample Reducing Agent	10X	Invitrogen/ Thermo Fisher Scientific	NP0009
NuPAGE™ 4 to 12%, Bis-Tris, 1.0 mm, Mini Protein Gel.	15-well	Invitrogen/ Thermo Fisher Scientific	NP0323BOX
NuPAGE™ MES SDS Running Buffer	(20X)	Invitrogen/ Thermo Fisher Scientific	NP0002
PageRuler™ Plus Prestained Protein Ladder,	10 to 250 kDa	Thermo Scientific™	26619
TBST	pH=7.5	Substratenheten Huddinge	MIK2334-1000
SuperSignal West Pico PLUS Chemiluminescent Substrate		Thermo Scientific	34580
TRIzol™ Reagent		Invitrogen/ThermoFisher Scintific	15596026
Chloroform		Sigma-Aldrich	C2432
Glycogen		Roche/Sigma-Alrdich	10901393001
Qubit™ RNA HS Assay Kit		Invitrogen/ThermoFisher Scintific	Q32855
Nextera XT Index Kit v2	Sets A-D	Illumina	FC-131-2001,2,3,4
(RS)-2-Hydroxyglutaric acid, disodium salt	(2,3,3-D3; OD, 98%) CP 95%	Cambridge Isotope Laboratories	DLM-9104-PK
L2HG standard		Sigma-Aldrich	90790
D2HG standard		Sigma-Aldrich	16859
TrypLE		ThermoFisher Scintific	12604013
isofluran		Baxter Medical AB	N01AB06
Mini-PROTEAN® TGX Stain-free gels	4-20 %	BioRad	4568095
Precision Plus Protein Dual Color Standards		BioRad	1610374
Laemmli sample buffer	4x	BioRad	1610747
Tris/Glycine/SDS Electrophoresis Buffer	10x	BioRad	1610772EDU
Albumin		Roth	8076.3
CHIR99021		Millipore	361559-5MG
IWP2		Millipore	681671
Geltrex		Thermo Fisher Scientific	A1413203
L-ascorbic acid 2-phosphate		Sigma Aldrich	A8960
Lactate		Sigma Aldrich	L4263
0.25 % Trypsin-EDTA		Thermo Fisher Scientific	25200-056

RPMI 1640 (1x) with HEPES, with Glutamax		Thermo Fisher Scientific	72400021
B27 Supplement with insulin (50x)		Thermo Fisher Scientific	17504-001
E8 Basal medium + Essential 8 supplement (50x)		Thermo Fisher Scientific	A1517001
Cardiac Troponin T Ab, REAfinity		Miltenyi Biotec	130-129-225

Supplementary Table 7: Characteristics of some of the utilized accessories

Material	Notes	Manufacturer	Reference ID
Multiply ® -µStrip Pro 8-strip	RNase free	Sarstedt	72.991.002
SafeSeal micro tube 2ml		Sarstedt	72.695.500
Assay plate 384 well	Black with clear flat bottom	Corning	3683
Hard-Shell® 384-Well PCR Plates, thin wall, skirted, clear/white		BioRad	HSP3905
Falcon® 6-well Clear Flat Bottom TC-treated Multiwell Cell Culture Plate		Corning	353046
Falcon® 24-well Clear Flat Bottom TC-treated Multiwell Cell Culture Plate		Corning	353047
Retsch Grinding Ball ss 2 Ø		Retsch /Germany	22.455.0010
Corning® 100 mm TC-treated Culture Dish		Corning	430167

References (Supplementary Information):

1 van Heesch, S., Witte, F., Schneider-Lunitz, V., Schulz, J. F., Adami, E., Faber, A. B., Kirchner, M., Maatz, H., Blachut, S., Sandmann, C. L., Kanda, M., Worth, C. L., Schafer, S., Calviello, L., Merriott, R., Patone, G., Hummel, O., Wyler, E., Obermayer, B., Mucke, M. B., Lindberg, E. L., Trnka, F., Memczak, S., Schilling, M., Felkin, L. E., Barton, P. J. R., Quaife, N. M., Vanezis, K., Diecke, S., Mukai, M., Mah, N., Oh, S. J., Kurtz, A., Schramm, C., Schwinge, D., Sebode, M., Harakalova, M., Asselbergs, F. W., Vink, A., de Weger, R. A., Viswanathan, S., Widjaja, A. A., Gartner-Rommel, A., Milting, H., Dos Remedios, C., Knosalla, C., Mertins, P., Landthaler, M., Vingron, M., Linke, W. A., Seidman, J. G., Seidman, C. E., Rajewsky, N., Ohler, U., Cook, S. A. & Hubner, N. The Translational Landscape of the Human Heart. *Cell* **178**, 242-260 e229, (2019).

2 Sweet, M. E., Cocciolo, A., Slavov, D., Jones, K. L., Sweet, J. R., Graw, S. L., Reece, T. B., Ambardekar, A. V., Bristow, M. R., Mestroni, L. & Taylor, M. R. G. Transcriptome analysis of human heart failure reveals dysregulated cell adhesion in dilated cardiomyopathy and activated immune pathways in ischemic heart failure. *BMC genomics* **19**, 812, (2018).

3 Sielemann, K., Elbeck, Z., Gartner, A., Brodehl, A., Stanasiuk, C., Fox, H., Paluszkiewicz, L., Tiesmeier, J., Wlost, S., Gummert, J., Albaum, S. P., Sielemann, J., Knoll, R. & Milting, H. Distinct Myocardial Transcriptomic Profiles of Cardiomyopathies Stratified by the Mutant Genes. *Genes (Basel)* **11**, (2020).

4 Vigil-Garcia, M., Demkes, C. J., Eding, J. E. C., Versteeg, D., de Ruiter, H., Perini, I., Kooijman, L., Gladka, M. M., Asselbergs, F. W., Vink, A., Harakalova, M., Bossu, A., van Veen, T. A. B., Boogerd, C. J. & van Rooij, E. Gene expression profiling of hypertrophic cardiomyocytes identifies new players in pathological remodelling. *Cardiovascular research* **117**, 1532-1545, (2021).

5 Lau, A., Tian, W., Whitman, S. A. & Zhang, D. D. The predicted molecular weight of Nrf2: it is what it is not. *Antioxidants & redox signaling* **18**, 91-93, (2013).

6 Bos, J. M., Hebl, V. B., Oberg, A. L., Sun, Z., Herman, D. S., Teekakirikul, P., Seidman, J. G., Seidman, C. E., Dos Remedios, C. G., Maleszewski, J. J., Schaff, H. V., Dearani, J. A., Noseworthy, P. A., Friedman, P. A., Ommen, S. R., Brozovich, F. V. & Ackerman, M. J. Marked

Up-Regulation of ACE2 in Hearts of Patients With Obstructive Hypertrophic Cardiomyopathy: Implications for SARS-CoV-2-Mediated COVID-19. *Mayo Clin Proc* **95**, 1354-1368, (2020).

7 Vikhorev, P. G., Smoktunowicz, N., Munster, A. B., Copeland, O., Kostin, S., Montgiraud, C., Messer, A. E., Toliat, M. R., Li, A., Dos Remedios, C. G., Lal, S., Blair, C. A., Campbell, K. S., Guglin, M., Richter, M., Knoll, R. & Marston, S. B. Abnormal contractility in human heart myofibrils from patients with dilated cardiomyopathy due to mutations in TTN and contractile protein genes. *Scientific reports* **7**, 14829, (2017).

8 Land, S., Park-Holohan, S. J., Smith, N. P., Dos Remedios, C. G., Kentish, J. C. & Niederer, S. A. A model of cardiac contraction based on novel measurements of tension development in human cardiomyocytes. *Journal of Molecular and Cellular Cardiology* **106**, 68-83, (2017).

9 Martin-Garrido, A., Biesiadecki, B. J., Salhi, H. E., Shaifta, Y., Dos Remedios, C. G., Ayaz-Guner, S., Cai, W., Ge, Y., Avkiran, M. & Kentish, J. C. Monophosphorylation of cardiac troponin-I at Ser-23/24 is sufficient to regulate cardiac myofibrillar Ca(2+) sensitivity and calpain-induced proteolysis. *Journal of Biological Chemistry* **293**, 8588-8599, (2018).

10 Brayson, D., Frustaci, A., Verardo, R., Chimenti, C., Russo, M. A., Hayward, R., Ahmad, S., Vizcay-Barrena, G., Protti, A., Zammit, P. S., dos Remedios, C. G., Ehler, E., Shah, A. M. & Shanahan, C. M. Prelamin A mediates myocardial inflammation in dilated and HIV-associated cardiomyopathies. *JCI Insight* **4**, (2019).

11 Mollova, M., Bersell, K., Walsh, S., Savla, J., Das, L. T., Park, S. Y., Silberstein, L. E., Dos Remedios, C. G., Graham, D., Colan, S. & Kuhn, B. Cardiomyocyte proliferation contributes to heart growth in young humans. *Proceedings of the National Academy of Sciences* **110**, 1446-1451, (2013).

12 McNamara, J. W., Li, A., Lal, S., Bos, J. M., Harris, S. P., van der Velden, J., Ackerman, M. J., Cooke, R. & Dos Remedios, C. G. MYBPC3 mutations are associated with a reduced super-relaxed state in patients with hypertrophic cardiomyopathy. *PloS one* **12**, e0180064, (2017).

13 Zhao, X., Dai, J., Ma, Y., Mi, Y., Cui, D., Ju, G., Macklin, W. B. & Jin, W. Dynamics of ten-eleven translocation hydroxylase family proteins and 5-hydroxymethylcytosine in oligodendrocyte differentiation. *Glia* **62**, 914-926, (2014).

14 Hsieh, M. C., Lai, C. Y., Ho, Y. C., Wang, H. H., Cheng, J. K., Chau, Y. P. & Peng, H. Y. Tet1-dependent epigenetic modification of BDNF expression in dorsal horn neurons mediates neuropathic pain in rats. *Scientific reports* **6**, 37411, (2016).

15 Poerschke, S., Oeljeklaus, S., Cruz-Zaragoza, L. D., Schenzielorz, A., Dahal, D., Hillen, H. S., Das, H., Kremer, L. S., Valpadashi, A., Breuer, M., Sattmann, J., Richter-Dennerlein, R., Warscheid, B., Dennerlein, S. & Rehling, P. Identification of TMEM126A as OXA1L-interacting protein reveals cotranslational quality control in mitochondria. *Molecular cell* **84**, 345-358 e345, (2024).
