# Supplementary material for: Epigenetic modulators link mitochondrial redox homeostasis to cardiac function in a sex-dependent manner
Source: Nat Commun. 2024 Mar 20;15:2358. doi: 10.1038/s41467-024-46384-8 (PMC10954618; doi:10.1038/s41467-024-46384-8)
Supplement: Supplementary file 3 — Description of Additional Supplementary Files [file 41467_2024_46384_MOESM3_ESM.pdf]

Description of Additional Supplementary Files for

Epigenetic modulators link mitochondrial redox homoeostasis to cardiac function in a sex-dependent manner

Zaher ElBeck[1,2*], Mohammad Bakhtiar Hossain[3], Humam Siga[1], Nikolay Oskolkov[4], Fredrik Karlsson[5], Julia Lindgren[6], Anna Walentinsson[7], Dominique Koppenhöfer[1], Rebecca Jarvis[8], Roland Bürli[8], Tanguy Jamier[8], Elske Franssen[8], Mike Firth[5], Andrea Degasperi[5,9], Claus Bendtsen[5], Robert I. Menzies[3], Katrin Streckfuss-Bömeke[10,11,12], Michael Kohlhaas[12], Alexander G. Nickel[12], Lars H. Lund[13], Christoph Maack[12], Ákos Végvári[14] and Christer Betsholtz[1,2]

File Name: Supplementary Data 1.

Description: This Excel file contains multiple sheets with complete lists of enriched pathways obtained through IPA pathway analyses. Selected pathways from each list were plotted in various figures and referenced in this Supplementary Data file for the complete list.

File Name: Supplementary Data 2.

Description: This file presents densitometry quantifications for all Western blotting bands featured in this study, extracted from ImageLab software.

File Name: Supplementary Data 3.

Description: This Excel file contains multiple sheets with complete lists of differentially expressed genes generated in this study. Additionally, it includes a list of differentially succinylated peptides in mitochondrial metabolic enzymes, obtained from Ali et al., 2020, which was used to generate Figure 2b. Furthermore, the file includes a list of top differentially hydroxymethylated genes in their introns, utilized for IPA pathway analysis presented in Supplementary Figure 7e.

File Name: Supplementary Data 4

Description: This Excel file contains two sheets with complete lists of annotated differentially methylated regions (DMR) identified for 5mC and for 5hmC in $Mlp^{-/-}$.
