# Supplementary material for: Epigenetic modulators link mitochondrial redox homeostasis to cardiac function in a sex-dependent manner
Source: Nat Commun. 2024 Mar 20;15:2358. doi: 10.1038/s41467-024-46384-8 (PMC10954618; doi:10.1038/s41467-024-46384-8)
Supplement: Supplementary file 5 — Supplementary Data 2 [file 41467_2024_46384_MOESM5_ESM.pdf]

Supplementary Data File 2

Epigenetic modulators link mitochondrial redox homeostasis to cardiac function in a sex-dependent manner

Zaher ElBeck[1,2*], Mohammad Bakhtiar Hossain[3], Humam Siga[1], Nikolay Oskolkov[4], Fredrik Karlsson[5], Julia Lindgren[6], Anna Walentinsson[7], Dominique Koppenhöfer[1], Rebecca Jarvis[8], Roland Bürli[8], Tanguy Jamier[8], Elske Franssen[8], Mike Firth[5], Andrea Degasperi[5,9], Claus Bendtsen[5], Robert I. Menzies[3], Katrin Streckfuss-Bömeke[10,11,12], Michael Kohlhaas[12], Alexander G. Nickel[12], Lars H. Lund[13], Christoph Maack[12], Ákos Végvári[14] and Christer Betsholtz[1,2]

[1]Department of Medicine Huddinge, Karolinska Institutet, Campus Flemingsberg, 141 57 Huddinge, Sweden. [2]Departmenty of Immunology, Genetics and Pathology, Rudbeck Laboratory, Uppsala University, Uppsala, Sweden. [3]Bioscience Renal, Research and Early Development, Cardiovascular, Renal and Metabolism (CVRM), BioPharmaceuticals R&D, AstraZeneca, Gothenburg, Sweden. [4]Department of Biology, National Bioinformatics Infrastructure Sweden, Science for Life Laboratory, Lund University, Sweden. [5]Data Sciences and Quantitative Biology, Discovery Sciences, R&D, AstraZeneca, Gothenburg, Sweden. [6] Translational Genomics, Centre for Genomics Research, Discovery Sciences, R&D, AstraZeneca, Gothenburg, Sweden. [7]Translational Science & Experimental Medicine, Research and Early Development, Cardiovascular, Renal and Metabolism (CVRM), BioPharmaceuticals R&D, AstraZeneca, Gothenburg, Sweden. [8]Neuroscience, BioPharmaceuticals R&D, AstraZeneca, Cambridge, United Kingdom. [9]Early Cancer Institute, University of Cambridge, Cambridge, United Kingdom. [10]Institute of Pharmacology and Toxicology, University of Würzburg, Germany [11]Clinic for Cardiology and Pneumology, Georg-August University Göttingen and DZHK (German Center for Cardiovascular Research), Partner Site Göttingen, Germany. [12]Department of Translational Research, Comprehensive Heart Failure Center (CHFC), University Clinic Würzburg, Würzburg, Germany. [13]Department of Medicine Karolinska Institutet, and Department of Cardiology, Karolinska University Hospital, Stockholm, Sweden. [14]Division of Chemistry I, Department of Medical Biochemistry & Biophysics, Karolinska Institutet, Sweden.

***Corresponding author:** zaher.elbeck@ki.se

Description: This file presents densitometry quantifications for all Western blotting bands featured in this study, extracted from ImageLab software.

Image Report: Intensity analysis of Fig. 1c (IDH2 panel)

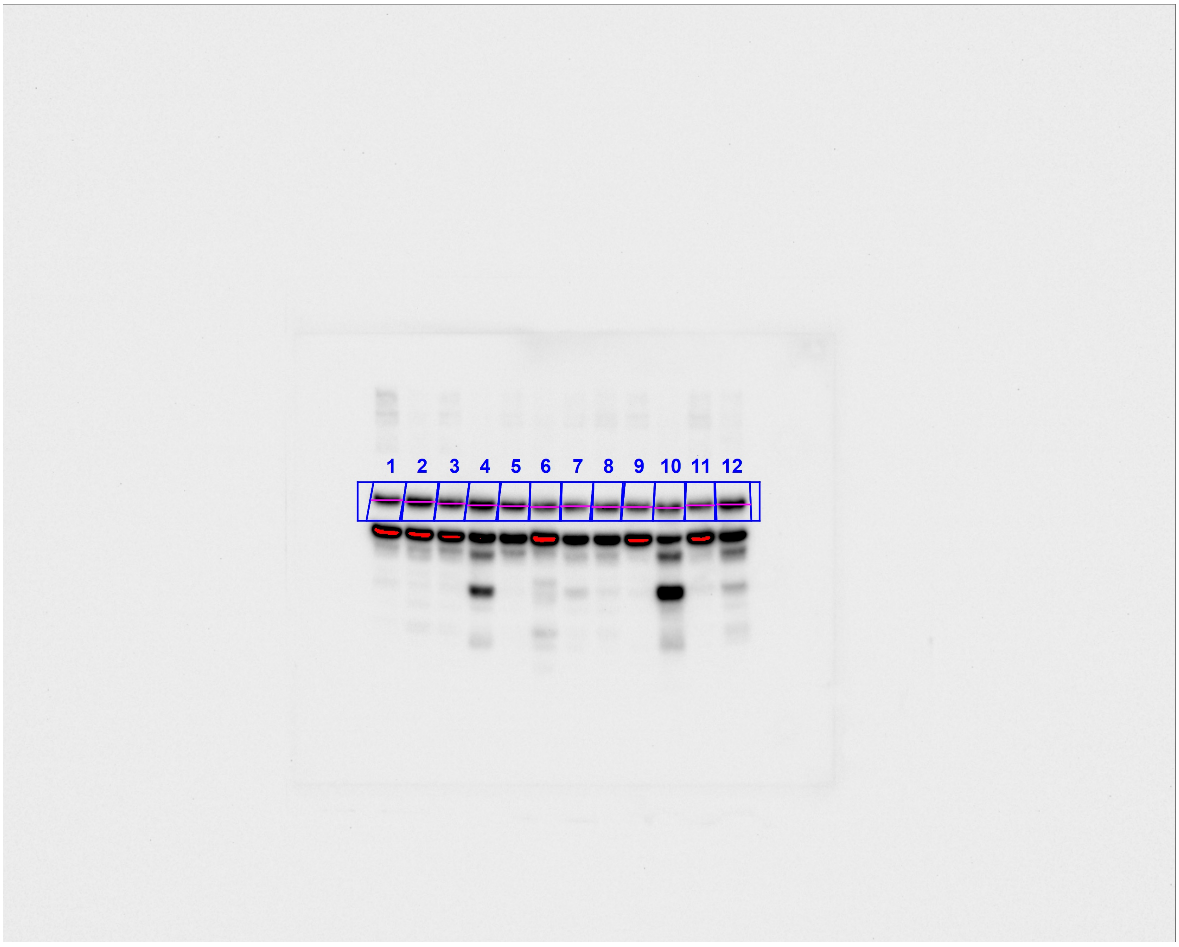

Acquisition Information

Imager	ChemiDoc Touch
Exposure Time (sec)	500.500 (Signal Accumulation)
Serial Number	732BR0263
Software Version	2.3.0.07
Application	Chemiluminescence
Excitation Source	No Illumination
Emission Filter	No Filter
Binning	2x2

Image Information

Acquisition Date	7/12/2022 12:25:34
User Name	m
Image Area (mm)	X: 180.0 Y: 144.1
Pixel Size (µm)	X: 130.5 Y: 130.5
Data Range (Int)	500 - 65535

Analysis Settings

Detection	Lane detection:<br>Manually created lanes (Copied)<br><br>Band detection:<br>Automatically detected bands with sensitivity: Low<br><br>Lane Background Subtraction:<br>Lane background subtracted with disk size: 79.9<br><br>Lane width: Variable

Lane Statistics

Lane No.	Adj. Total Band Vol. (Int)	Total Band Vol. (Int)	Adj. Total Lane Vol. (Int)	Total Lane Vol. (Int)	Bkgd. Vol. (Int)	Norm. Factor
1	6 887 907	7 729 254	7 328 763	8 731 008	1 402 245	N/A
2	9 108 334	10 028 314	9 346 822	10 726 792	1 379 970	N/A
3	6 880 790	7 867 370	7 024 360	8 555 260	1 530 900	N/A
4	9 875 411	10 968 613	9 961 399	11 408 284	1 446 885	N/A
5	7 050 564	7 832 520	7 191 072	8 404 452	1 213 380	N/A
6	5 614 972	6 547 483	5 703 032	7 056 677	1 353 645	N/A
7	4 846 900	5 599 984	4 977 050	6 232 190	1 255 140	N/A
8	5 784 136	6 611 086	5 854 695	7 095 120	1 240 425	N/A
9	5 410 934	6 259 094	5 487 428	6 759 668	1 272 240	N/A
10	6 159 538	7 009 798	6 261 584	7 536 974	1 275 390	N/A
11	5 376 315	6 148 555	5 585 160	6 826 260	1 241 100	N/A
12	9 849 676	10 841 056	10 122 572	11 561 672	1 439 100	N/A

Lane And Band Analysis

Lane 1

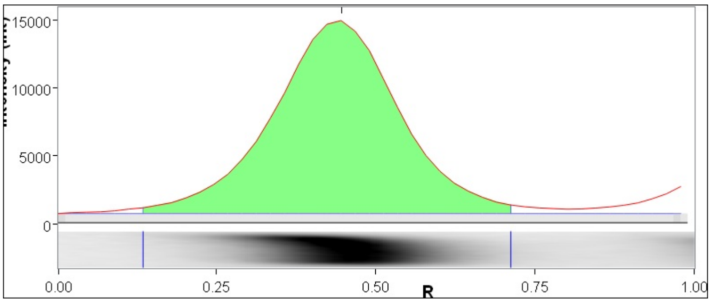

Band No.	Band Label	Mol. Wt. (KDa)	Relative Front	Adj. Volume (Int)	Volume (Int)	Abs. Quant.	Rel. Quant.	Band %	Lane %
1		N/A	0,467	6 887 907	7 729 254	N/A	N/A	100,0	94,0

Band Detection	Automatically detected bands with sensitivity: Low
Lane Background	Lane background subtracted with disk size: 79.9
Lane Width	5.09 mm

Lane 2

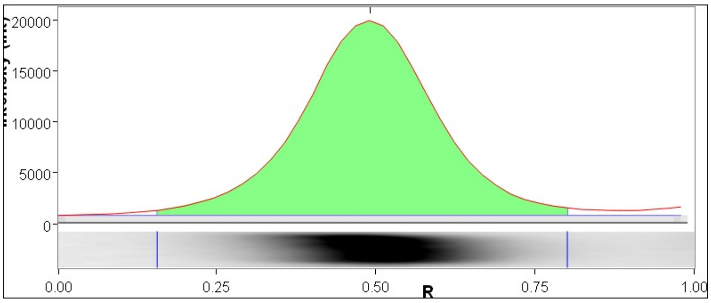

Band No.	Band Label	Mol. Wt. (KDa)	Relative Front	Adj. Volume (Int)	Volume (Int)	Abs. Quant.	Rel. Quant.	Band %	Lane %
1		N/A	0,511	9 108 334	10 028 314	N/A	N/A	100,0	97,4

Band Detection	Automatically detected bands with sensitivity: Low
Lane Background	Lane background subtracted with disk size: 79.9
Lane Width	4.96 mm

Lane 3

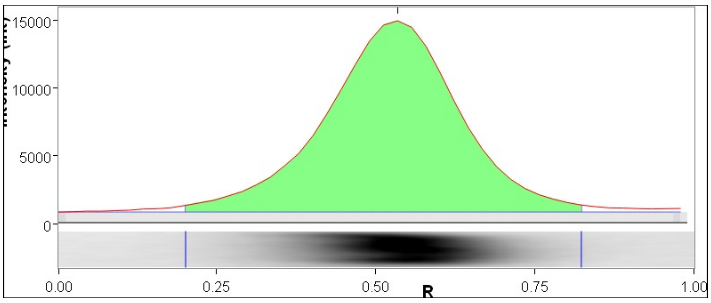

Band No.	Band Label	Mol. Wt. (KDa)	Relative Front	Adj. Volume (Int)	Volume (Int)	Abs. Quant.	Rel. Quant.	Band %	Lane %
1		N/A	0,556	6 880 790	7 867 370	N/A	N/A	100,0	98,0

Band Detection	Automatically detected bands with sensitivity: Low
Lane Background	Lane background subtracted with disk size: 79.9
Lane Width	4.57 mm

Lane 4

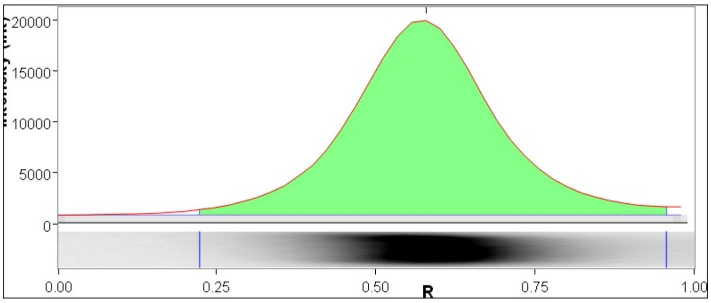

Band No.	Band Label	Mol. Wt. (KDa)	Relative Front	Adj. Volume (Int)	Volume (Int)	Abs. Quant.	Rel. Quant.	Band %	Lane %
1		N/A	0,600	9 875 411	10 968 613	N/A	N/A	100,0	99,1

Band Detection	Automatically detected bands with sensitivity: Low

Lane Background	Lane background subtracted with disk size: 79.9
Lane Width	4.83 mm

Lane 5

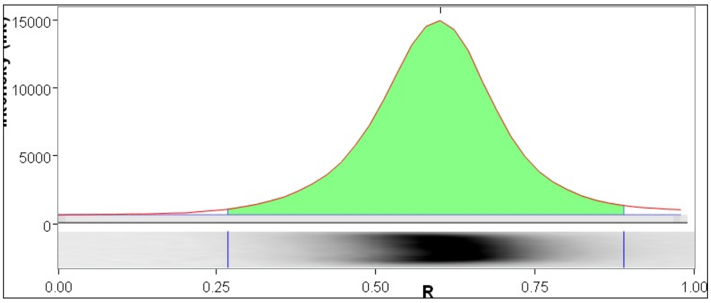

Band No.	Band Label	Mol. Wt. (KDa)	Relative Front	Adj. Volume (Int)	Volume (Int)	Abs. Quant.	Rel. Quant.	Band %	Lane %
1		N/A	0,622	7 050 564	7 832 520	N/A	N/A	100,0	98,0

Band Detection	Automatically detected bands with sensitivity: Low
Lane Background	Lane background subtracted with disk size: 79.9
Lane Width	4.70 mm

Lane 6

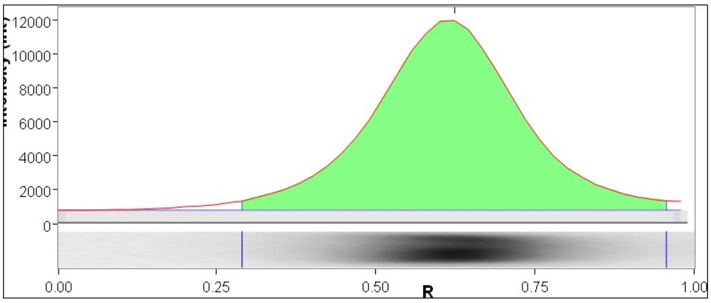

Band No.	Band Label	Mol. Wt. (KDa)	Relative Front	Adj. Volume (Int)	Volume (Int)	Abs. Quant.	Rel. Quant.	Band %	Lane %
1		N/A	0,644	5 614 972	6 547 483	N/A	N/A	100,0	98,5

Band Detection	Automatically detected bands with sensitivity: Low
Lane Background	Lane background subtracted with disk size: 79.9
Lane Width	4.83 mm

Lane 7

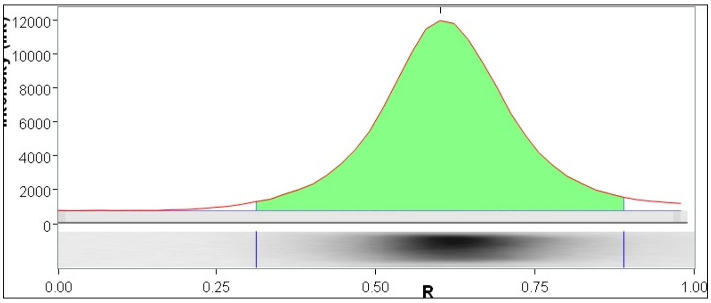

Band No.	Band Label	Mol. Wt. (KDa)	Relative Front	Adj. Volume (Int)	Volume (Int)	Abs. Quant.	Rel. Quant.	Band %	Lane %
1		N/A	0,622	4 846 900	5 599 984	N/A	N/A	100,0	97,4

Band Detection	Automatically detected bands with sensitivity: Low
Lane Background	Lane background subtracted with disk size: 79.9
Lane Width	4.96 mm

Lane 8

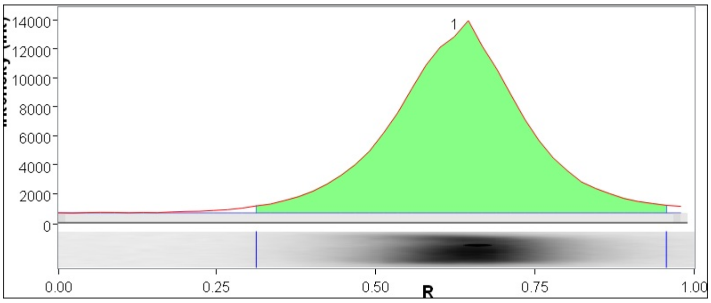

Band No.	Band Label	Mol. Wt. (KDa)	Relative Front	Adj. Volume (Int)	Volume (Int)	Abs. Quant.	Rel. Quant.	Band %	Lane %
1		N/A	0,644	5 784 136	6 611 086	N/A	N/A	100,0	98,8

Band Detection	Automatically detected bands with sensitivity: Low
Lane Background	Lane background subtracted with disk size: 79.9
Lane Width	4.83 mm

Lane 9

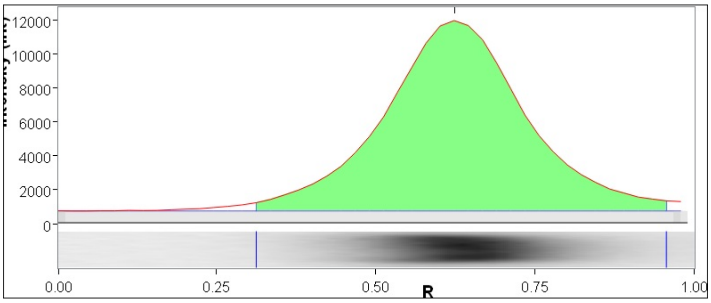

Band No.	Band Label	Mol. Wt. (KDa)	Relative Front	Adj. Volume (Int)	Volume (Int)	Abs. Quant.	Rel. Quant.	Band %	Lane %
1		N/A	0,644	5 410 934	6 259 094	N/A	N/A	100,0	98,6

Band Detection	Automatically detected bands with sensitivity: Low
Lane Background	Lane background subtracted with disk size: 79.9
Lane Width	4.96 mm

Lane 10

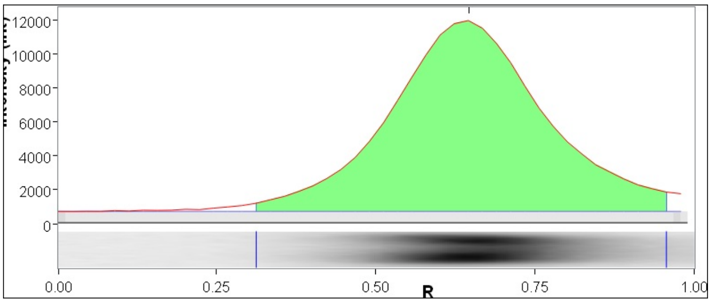

Band No.	Band Label	Mol. Wt. (KDa)	Relative Front	Adj. Volume (Int)	Volume (Int)	Abs. Quant.	Rel. Quant.	Band %	Lane %
1		N/A	0,667	6 159 538	7 009 798	N/A	N/A	100,0	98,4

Band Detection	Automatically detected bands with sensitivity: Low
Lane Background	Lane background subtracted with disk size: 79.9
Lane Width	4.83 mm

Lane 11

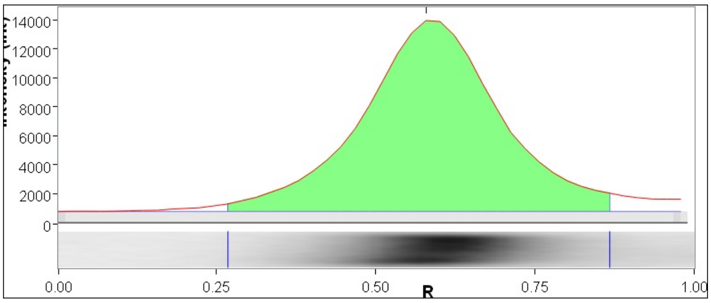

Band No.	Band Label	Mol. Wt. (KDa)	Relative Front	Adj. Volume (Int)	Volume (Int)	Abs. Quant.	Rel. Quant.	Band %	Lane %
1		N/A	0,600	5 376 315	6 148 555	N/A	N/A	100,0	96,3

Band Detection	Automatically detected bands with sensitivity: Low
Lane Background	Lane background subtracted with disk size: 79.9
Lane Width	4.57 mm

Lane 12

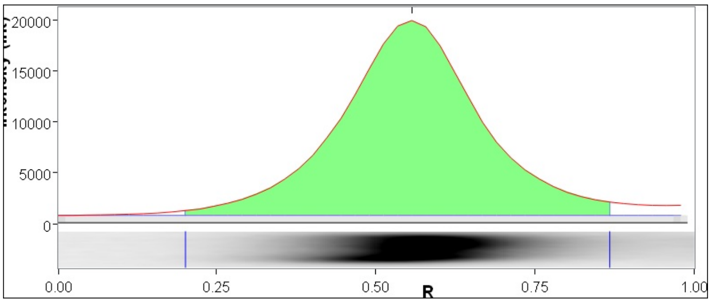

Band No.	Band Label	Mol. Wt. (KDa)	Relative Front	Adj. Volume (Int)	Volume (Int)	Abs. Quant.	Rel. Quant.	Band %	Lane %
1		N/A	0,578	9 849 676	10 841 056	N/A	N/A	100,0	97,3

Band Detection	Automatically detected bands with sensitivity: Low

Lane Background	Lane background subtracted with disk size: 79.9
Lane Width	5.35 mm

Image Report: Fig. 1c (Oxphos_ individual oxphos panels)

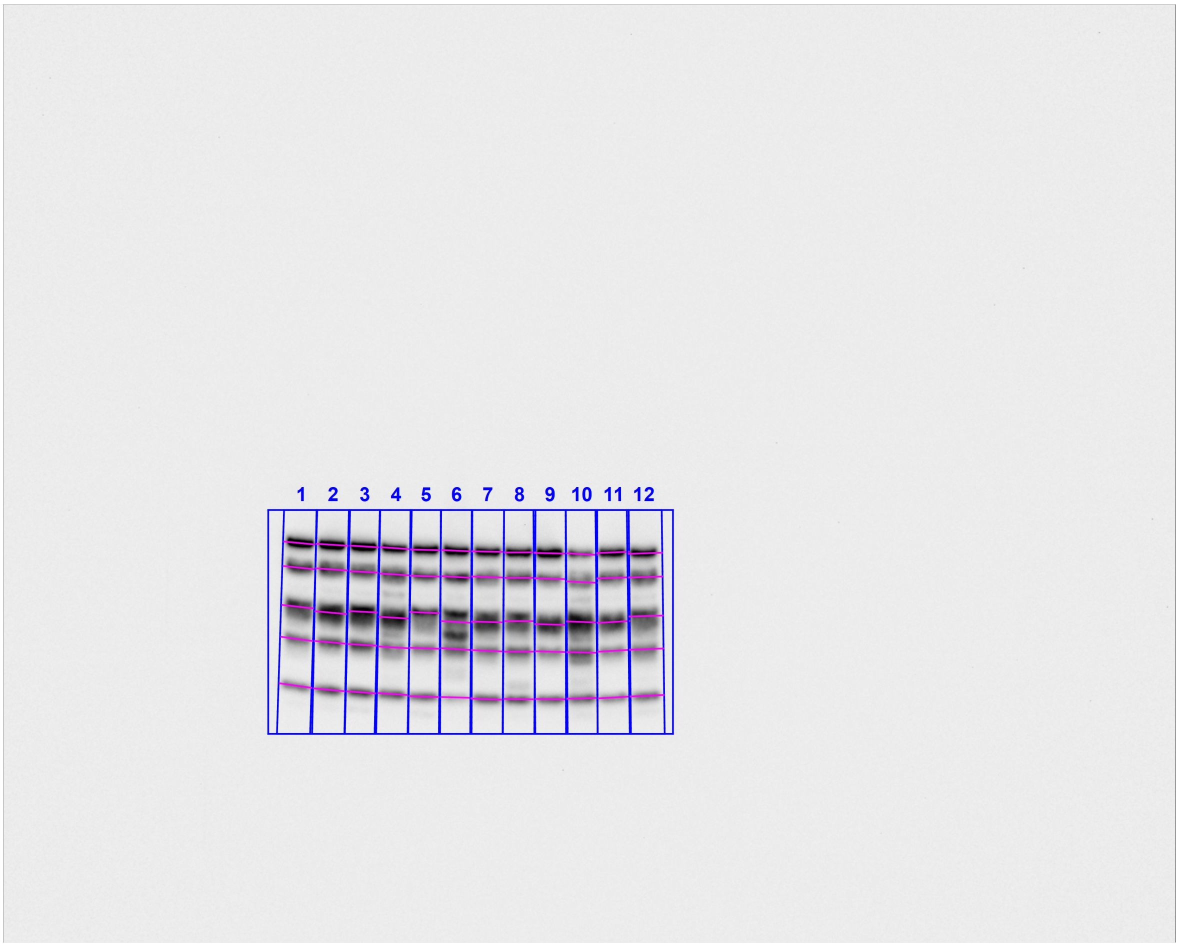

Acquisition Information

Imager	ChemiDoc Touch
Exposure Time (sec)	45.222 (Signal Accumulation)
Serial Number	732BR0263
Software Version	2.3.0.07
Application	Chemiluminescence
Excitation Source	No Illumination
Emission Filter	No Filter
Binning	2x2

Image Information

Acquisition Date	7/12/2022 9:58:33
User Name	m
Image Area (mm)	X: 180.0 Y: 144.1
Pixel Size (µm)	X: 130.5 Y: 130.5

Data Range (Int)	500 - 36351

Analysis Settings

Detection	Lane detection:<br>Manually created lanes<br><br>Band detection:<br>Automatically detected bands with sensitivity: Low<br>Manually adjusted bands<br><br>Lane Background Subtraction:<br>Lane background subtracted with disk size: 79.9<br><br>Lane width: Variable

Lane Statistics

Lane No.	Adj. Total Band Vol. (Int)	Total Band Vol. (Int)	Adj. Total Lane Vol. (Int)	Total Lane Vol. (Int)	Bkgd. Vol. (Int)	Norm. Factor
1	30 862 962	33 962 058	32 898 723	39 241 059	6 342 336	N/A
2	36 699 412	40 018 864	38 735 034	45 085 290	6 350 256	N/A
3	37 366 350	40 564 545	39 170 320	45 074 680	5 904 360	N/A
4	33 351 985	36 777 593	36 051 505	42 420 241	6 368 736	N/A
5	28 031 400	31 181 472	29 925 000	36 131 112	6 206 112	N/A
6	32 896 478	36 100 382	35 917 491	42 325 299	6 407 808	N/A
7	30 414 288	33 839 836	32 610 118	39 211 174	6 601 056	N/A
8	30 540 059	33 991 715	33 074 004	39 677 172	6 603 168	N/A
9	30 140 992	33 512 010	31 996 456	38 687 800	6 691 344	N/A
10	31 834 171	35 346 211	34 715 768	41 338 472	6 622 704	N/A
11	26 263 029	29 597 685	28 097 082	34 974 810	6 877 728	N/A
12	30 318 106	33 980 390	32 962 852	40 020 100	7 057 248	N/A

Lane And Band Analysis

Lane 1

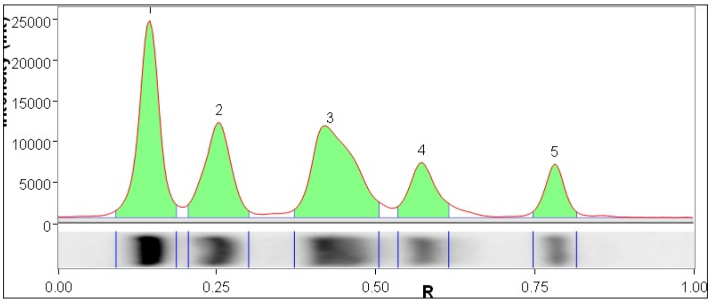

Band No.	Band Label	Mol. Wt. (KDa)	Relative Front	Adj. Volume (Int)	Volume (Int)	Abs. Quant.	Rel. Quant.	Band %	Lane %
1		N/A	0,148	9 653 709	10 278 333	N/A	N/A	31,3	29,3
2		N/A	0,258	6 038 136	6 662 760	N/A	N/A	19,6	18,4
3		N/A	0,432	9 217 728	10 082 592	N/A	N/A	29,9	28,0
4		N/A	0,576	3 445 143	3 973 671	N/A	N/A	11,2	10,5
5		N/A	0,784	2 508 246	2 964 702	N/A	N/A	8,1	7,6

Band Detection	Automatically detected bands with sensitivity: Low
Lane Background	Lane background subtracted with disk size: 79.9
Lane Width	5.09 mm

Lane 2

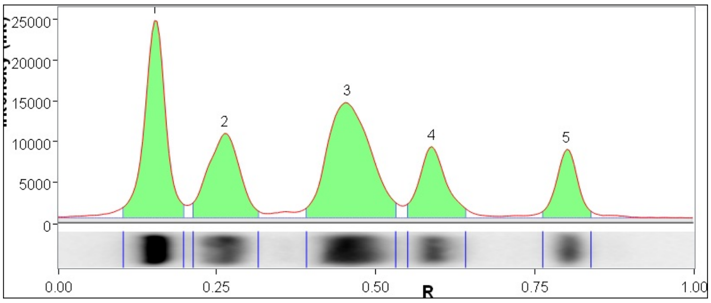

Band No.	Band Label	Mol. Wt. (KDa)	Relative Front	Adj. Volume (Int)	Volume (Int)	Abs. Quant.	Rel. Quant.	Band %	Lane %
1		N/A	0,155	10 077 106	10 702 510	N/A	N/A	27,5	26,0
2		N/A	0,265	6 428 346	7 101 858	N/A	N/A	17,5	16,6
3		N/A	0,458	12 112 462	13 026 514	N/A	N/A	33,0	31,3
4		N/A	0,591	4 586 334	5 187 684	N/A	N/A	12,5	11,8
5		N/A	0,803	3 495 164	4 000 298	N/A	N/A	9,5	9,0

Band Detection	Automatically detected bands with sensitivity: Low
Lane Background	Lane background subtracted with disk size: 79.9
Lane Width	4.96 mm

Lane 3

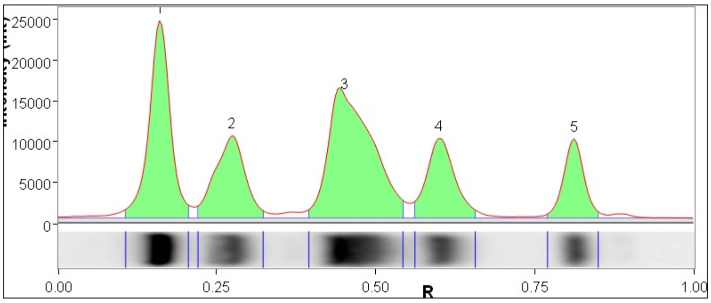

Band No.	Band Label	Mol. Wt. (KDa)	Relative Front	Adj. Volume (Int)	Volume (Int)	Abs. Quant.	Rel. Quant.	Band %	Lane %
1		N/A	0,163	9 672 530	10 276 385	N/A	N/A	25,9	24,7
2		N/A	0,277	5 684 735	6 310 955	N/A	N/A	15,2	14,5
3		N/A	0,455	13 142 745	14 037 345	N/A	N/A	35,2	33,6
4		N/A	0,602	5 143 355	5 724 845	N/A	N/A	13,8	13,1
5		N/A	0,814	3 722 985	4 215 015	N/A	N/A	10,0	9,5

Band Detection	Automatically detected bands with sensitivity: Low
Lane Background	Lane background subtracted with disk size: 79.9
Lane Width	4.57 mm

Lane 4

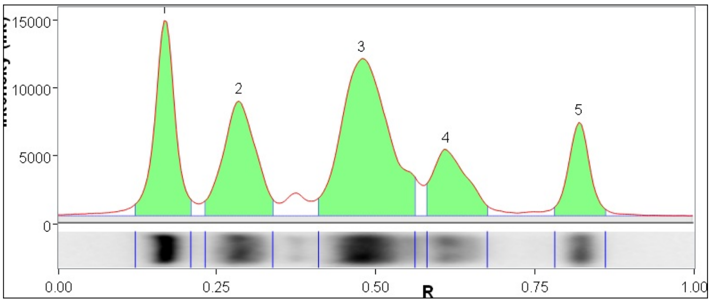

Band No.	Band Label	Mol. Wt. (KDa)	Relative Front	Adj. Volume (Int)	Volume (Int)	Abs. Quant.	Rel. Quant.	Band %	Lane %
1		N/A	0,170	6 844 704	7 423 680	N/A	N/A	20,5	19,0
2		N/A	0,288	6 420 832	7 120 428	N/A	N/A	19,3	17,8
3		N/A	0,481	12 801 593	13 790 677	N/A	N/A	38,4	35,5
4		N/A	0,614	3 875 972	4 503 196	N/A	N/A	11,6	10,8
5		N/A	0,822	3 408 884	3 939 612	N/A	N/A	10,2	9,5

Band Detection	Automatically detected bands with sensitivity: Low
Lane Background	Lane background subtracted with disk size: 79.9
Lane Width	4.83 mm

Lane 5

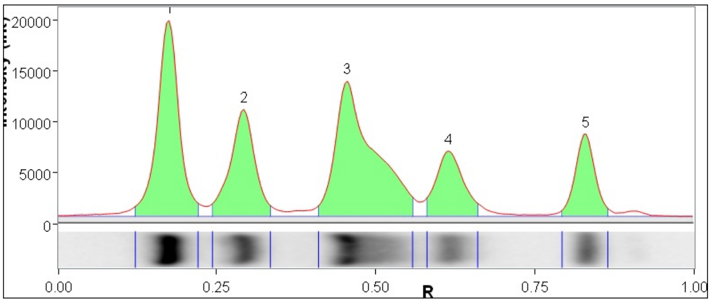

Band No.	Band Label	Mol. Wt. (KDa)	Relative Front	Adj. Volume (Int)	Volume (Int)	Abs. Quant.	Rel. Quant.	Band %	Lane %
1		N/A	0,178	7 703 532	8 338 248	N/A	N/A	27,5	25,7
2		N/A	0,295	4 753 044	5 340 744	N/A	N/A	17,0	15,9
3		N/A	0,458	9 425 448	10 365 768	N/A	N/A	33,6	31,5
4		N/A	0,617	3 209 076	3 726 252	N/A	N/A	11,4	10,7
5		N/A	0,833	2 940 300	3 410 460	N/A	N/A	10,5	9,8

Band Detection	Automatically detected bands with sensitivity: Low
Lane Background	Lane background subtracted with disk size: 79.9
Lane Width	4.70 mm

Lane 6

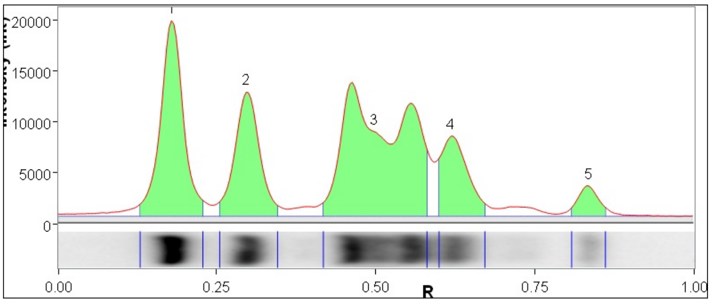

Band No.	Band Label	Mol. Wt. (KDa)	Relative Front	Adj. Volume (Int)	Volume (Int)	Abs. Quant.	Rel. Quant.	Band %	Lane %
1		N/A	0,182	8 158 537	8 813 881	N/A	N/A	24,8	22,7
2		N/A	0,299	5 593 734	6 200 534	N/A	N/A	17,0	15,6
3		N/A	0,500	14 161 380	15 253 620	N/A	N/A	43,0	39,4
4		N/A	0,621	3 871 310	4 356 750	N/A	N/A	11,8	10,8
5		N/A	0,837	1 111 517	1 475 597	N/A	N/A	3,4	3,1

Band Detection	Automatically detected bands with sensitivity: Low
Lane Background	Lane background subtracted with disk size: 79.9
Lane Width	4.83 mm

Lane 7

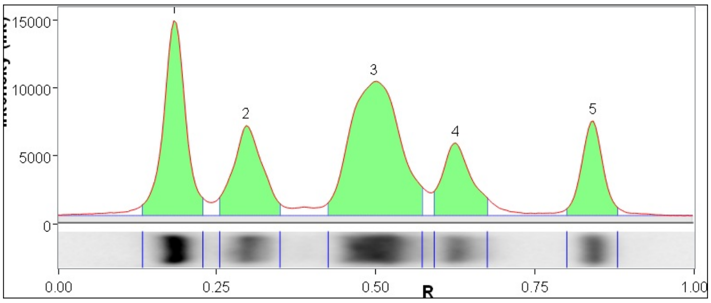

Band No.	Band Label	Mol. Wt. (KDa)	Relative Front	Adj. Volume (Int)	Volume (Int)	Abs. Quant.	Rel. Quant.	Band %	Lane %
1		N/A	0,186	7 374 394	8 024 498	N/A	N/A	24,2	22,6
2		N/A	0,299	4 500 986	5 151 090	N/A	N/A	14,8	13,8
3		N/A	0,500	11 535 090	12 535 250	N/A	N/A	37,9	35,4
4		N/A	0,629	3 556 648	4 131 740	N/A	N/A	11,7	10,9
5		N/A	0,845	3 447 170	3 997 258	N/A	N/A	11,3	10,6

Band Detection	Automatically detected bands with sensitivity: Low
Lane Background	Lane background subtracted with disk size: 79.9
Lane Width	4.96 mm

Lane 8

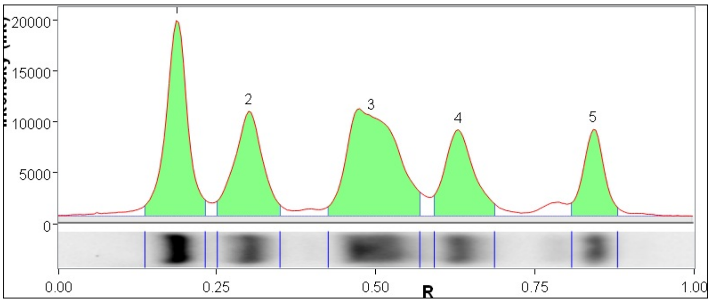

Band No.	Band Label	Mol. Wt. (KDa)	Relative Front	Adj. Volume (Int)	Volume (Int)	Abs. Quant.	Rel. Quant.	Band %	Lane %
1		N/A	0,189	7 663 477	8 313 789	N/A	N/A	25,1	23,2
2		N/A	0,303	5 347 425	6 022 749	N/A	N/A	17,5	16,2
3		N/A	0,496	9 683 973	10 659 441	N/A	N/A	31,7	29,3
4		N/A	0,633	4 570 055	5 220 367	N/A	N/A	15,0	13,8
5		N/A	0,845	3 275 129	3 775 369	N/A	N/A	10,7	9,9

Band Detection	Automatically detected bands with sensitivity: Low
Lane Background	Lane background subtracted with disk size: 79.9
Lane Width	4.83 mm

Lane 9

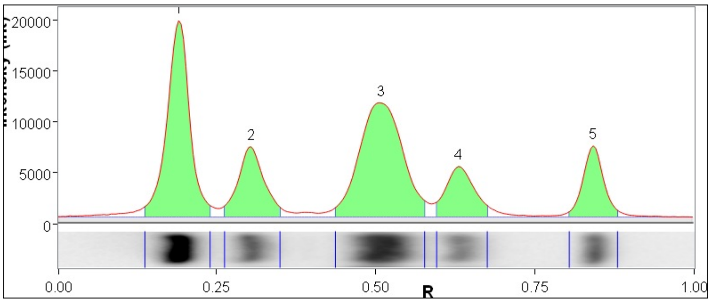

Band No.	Band Label	Mol. Wt. (KDa)	Relative Front	Adj. Volume (Int)	Volume (Int)	Abs. Quant.	Rel. Quant.	Band %	Lane %
1		N/A	0,193	9 523 066	10 232 754	N/A	N/A	31,6	29,8
2		N/A	0,307	3 873 910	4 482 214	N/A	N/A	12,9	12,1
3		N/A	0,511	10 600 138	11 563 286	N/A	N/A	35,2	33,1
4		N/A	0,633	3 035 592	3 593 204	N/A	N/A	10,1	9,5
5		N/A	0,845	3 108 286	3 640 552	N/A	N/A	10,3	9,7

Band Detection	Automatically detected bands with sensitivity: Low
Lane Background	Lane background subtracted with disk size: 79.9
Lane Width	4.96 mm

Lane 10

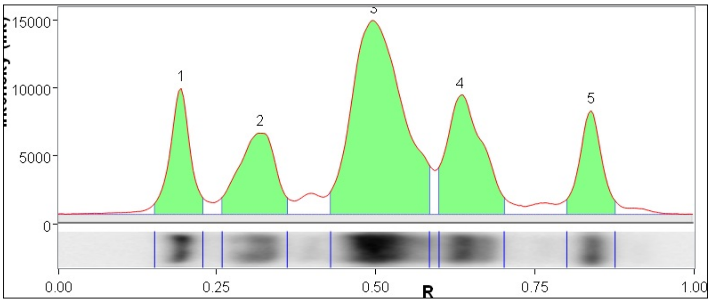

Band No.	Band Label	Mol. Wt. (KDa)	Relative Front	Adj. Volume (Int)	Volume (Int)	Abs. Quant.	Rel. Quant.	Band %	Lane %
1		N/A	0,197	3 657 820	4 184 626	N/A	N/A	11,5	10,5
2		N/A	0,322	4 329 259	5 031 667	N/A	N/A	13,6	12,5
3		N/A	0,500	14 336 612	15 390 224	N/A	N/A	45,0	41,3
4		N/A	0,636	6 163 978	6 866 386	N/A	N/A	19,4	17,8
5		N/A	0,841	3 346 502	3 873 308	N/A	N/A	10,5	9,6

Band Detection	Automatically detected bands with sensitivity: Low
Lane Background	Lane background subtracted with disk size: 79.9
Lane Width	4.83 mm

Lane 11

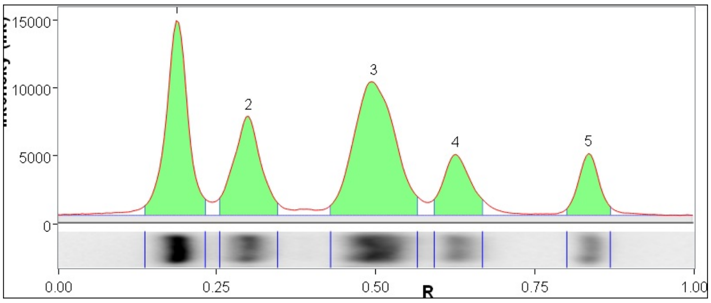

Band No.	Band Label	Mol. Wt. (KDa)	Relative Front	Adj. Volume (Int)	Volume (Int)	Abs. Quant.	Rel. Quant.	Band %	Lane %
1		N/A	0,189	7 256 964	7 934 316	N/A	N/A	27,6	25,8
2		N/A	0,303	4 594 902	5 246 202	N/A	N/A	17,5	16,4
3		N/A	0,500	9 456 330	10 420 254	N/A	N/A	36,0	33,7
4		N/A	0,629	2 787 798	3 334 890	N/A	N/A	10,6	9,9
5		N/A	0,837	2 167 035	2 662 023	N/A	N/A	8,3	7,7

Band Detection	Automatically detected bands with sensitivity: Low
Lane Background	Lane background subtracted with disk size: 79.9
Lane Width	5.09 mm

Lane 12

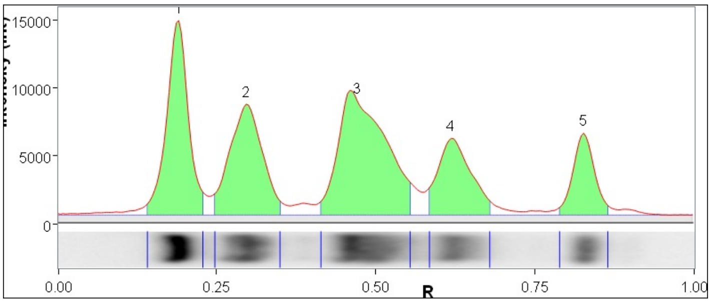

Band No.	Band Label	Mol. Wt. (KDa)	Relative Front	Adj. Volume (Int)	Volume (Int)	Abs. Quant.	Rel. Quant.	Band %	Lane %
1		N/A	0,193	7 119 158	7 760 726	N/A	N/A	23,5	21,6
2		N/A	0,299	6 113 264	6 861 760	N/A	N/A	20,2	18,5
3		N/A	0,473	9 835 326	10 851 142	N/A	N/A	32,4	29,8
4		N/A	0,621	4 271 421	4 966 453	N/A	N/A	14,1	13,0
5		N/A	0,830	2 978 937	3 540 309	N/A	N/A	9,8	9,0

Band Detection	Automatically detected bands with sensitivity: Low
Lane Background	Lane background subtracted with disk size: 79.9
Lane Width	5.35 mm

Image Report: Fig. 1c (Oxphos_ Total oxphos panel)

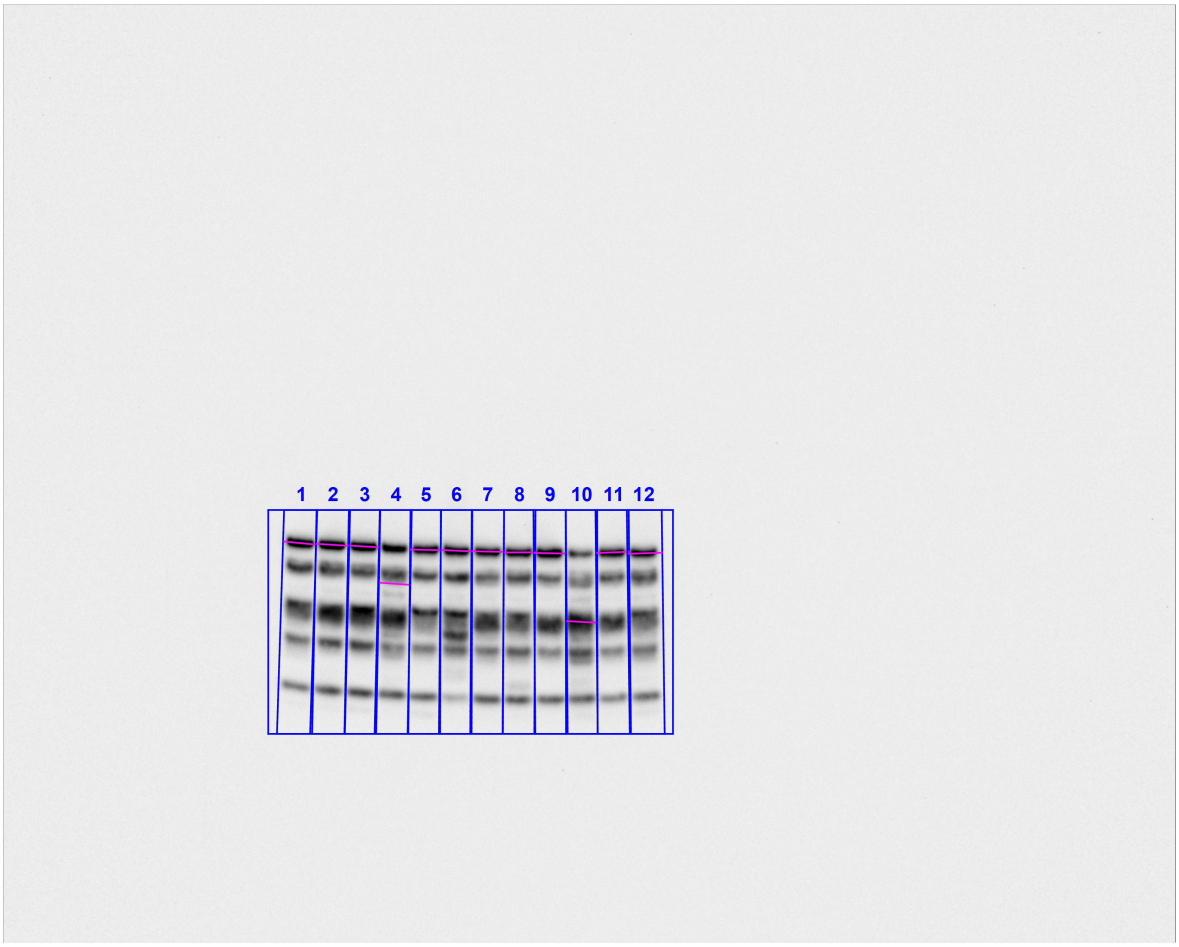

Acquisition Information

Imager	ChemiDoc Touch
Exposure Time (sec)	45.222 (Signal Accumulation)
Serial Number	732BR0263
Software Version	2.3.0.07
Application	Chemiluminescence
Excitation Source	No Illumination
Emission Filter	No Filter
Binning	2x2

Image Information

Acquisition Date	7/12/2022 9:58:33
User Name	m
Image Area (mm)	X: 180.0 Y: 144.1
Pixel Size (µm)	X: 130.5 Y: 130.5

Data Range (Int)	500 - 36351

Analysis Settings

Detection	Lane detection:<br>Manually created lanes<br><br>Band detection:<br>Automatically detected bands with sensitivity: Low<br>Manually adjusted bands<br><br>Lane Background Subtraction:<br>Lane background subtracted with disk size: 79.9<br><br>Lane width: Variable

Lane Statistics

Lane No.	Adj. Total Band Vol. (Int)	Total Band Vol. (Int)	Adj. Total Lane Vol. (Int)	Total Lane Vol. (Int)	Bkgd. Vol. (Int)	Norm. Factor
1	32 687 499	37 445 499	33 056 400	39 336 960	6 280 560	N/A
2	38 211 812	43 022 612	38 774 592	45 124 848	6 350 256	N/A
3	38 656 765	43 152 130	39 202 800	45 107 160	5 904 360	N/A
4	35 610 391	40 411 067	36 074 519	42 443 255	6 368 736	N/A
5	29 410 452	34 135 560	29 931 264	36 137 376	6 206 112	N/A
6	35 366 598	40 245 270	35 920 266	42 328 074	6 407 808	N/A
7	32 179 920	37 230 728	32 609 776	39 210 832	6 601 056	N/A
8	32 562 812	37 590 224	33 073 782	39 676 950	6 603 168	N/A
9	31 485 888	36 555 088	31 989 996	38 681 340	6 691 344	N/A
10	34 318 240	39 260 182	34 731 086	41 353 790	6 622 704	N/A
11	27 763 242	32 984 016	28 128 672	34 985 808	6 857 136	N/A
12	32 439 487	37 697 614	32 993 643	40 040 067	7 046 424	N/A

Lane And Band Analysis

Lane 1

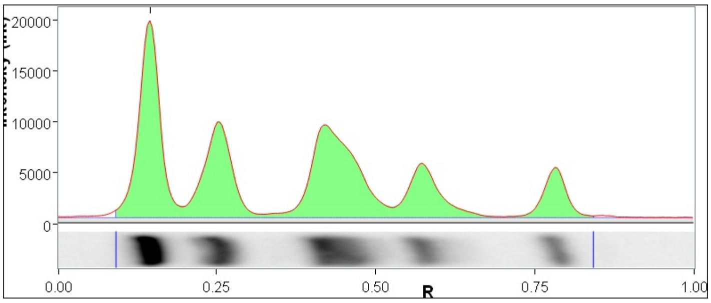

Band No.	Band Label	Mol. Wt. (KDa)	Relative Front	Adj. Volume (Int)	Volume (Int)	Abs. Quant.	Rel. Quant.	Band %	Lane %
1		N/A	0,148	32 687 499	37 445 499	N/A	N/A	100,0	98,9

Band Detection	Automatically detected bands with sensitivity: Low
Lane Background	Lane background subtracted with disk size: 79.9
Lane Width	5.09 mm

Lane 2

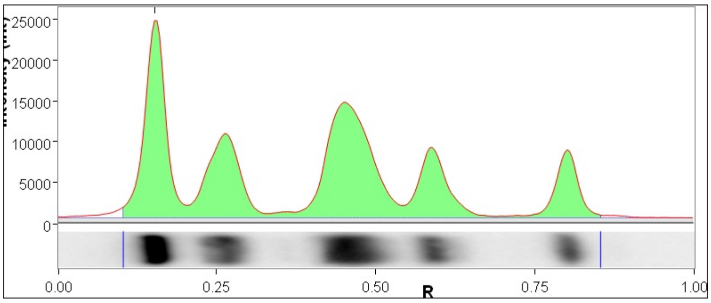

Band No.	Band Label	Mol. Wt. (KDa)	Relative Front	Adj. Volume (Int)	Volume (Int)	Abs. Quant.	Rel. Quant.	Band %	Lane %
1		N/A	0,155	38 211 812	43 022 612	N/A	N/A	100,0	98,5

Band Detection	Automatically detected bands with sensitivity: Low
Lane Background	Lane background subtracted with disk size: 79.9
Lane Width	4.96 mm

Lane 3

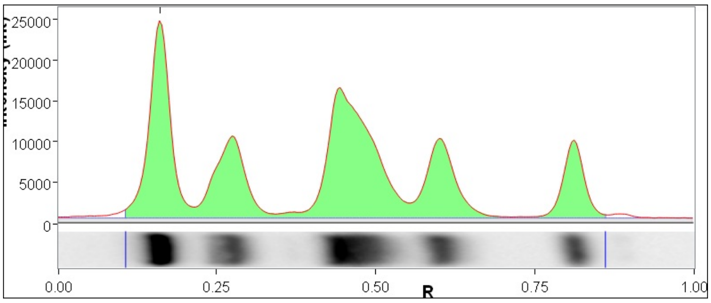

Band No.	Band Label	Mol. Wt. (KDa)	Relative Front	Adj. Volume (Int)	Volume (Int)	Abs. Quant.	Rel. Quant.	Band %	Lane %
1		N/A	0,163	38 656 765	43 152 130	N/A	N/A	100,0	98,6

Band Detection	Automatically detected bands with sensitivity: Low
Lane Background	Lane background subtracted with disk size: 79.9
Lane Width	4.57 mm

Lane 4

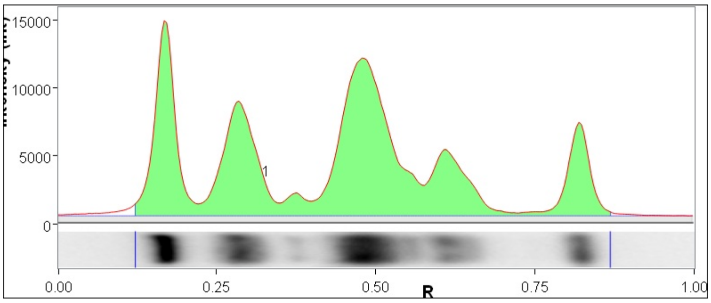

Band No.	Band Label	Mol. Wt. (KDa)	Relative Front	Adj. Volume (Int)	Volume (Int)	Abs. Quant.	Rel. Quant.	Band %	Lane %
1		N/A	0,330	35 610 391	40 411 067	N/A	N/A	100,0	98,7

Band Detection	Automatically detected bands with sensitivity: Low

Lane Background	Lane background subtracted with disk size: 79.9
Lane Width	4.83 mm

Lane 5

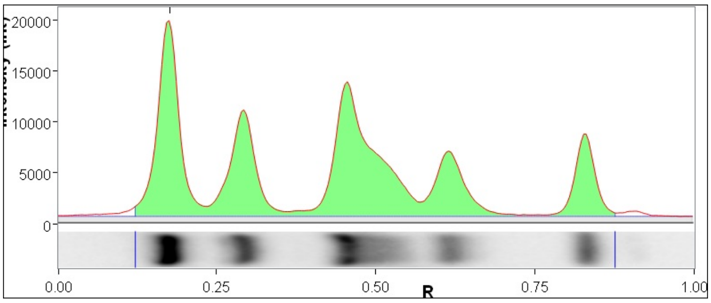

Band No.	Band Label	Mol. Wt. (KDa)	Relative Front	Adj. Volume (Int)	Volume (Int)	Abs. Quant.	Rel. Quant.	Band %	Lane %
1		N/A	0,178	29 410 452	34 135 560	N/A	N/A	100,0	98,3

Band Detection	Automatically detected bands with sensitivity: Low
Lane Background	Lane background subtracted with disk size: 79.9
Lane Width	4.70 mm

Lane 6

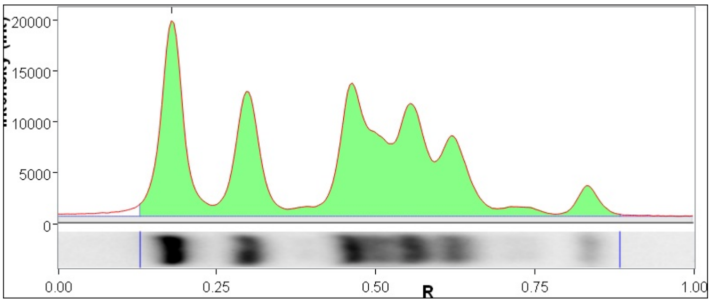

Band No.	Band Label	Mol. Wt. (KDa)	Relative Front	Adj. Volume (Int)	Volume (Int)	Abs. Quant.	Rel. Quant.	Band %	Lane %
1		N/A	0,182	35 366 598	40 245 270	N/A	N/A	100,0	98,5

Band Detection	Automatically detected bands with sensitivity: Low
Lane Background	Lane background subtracted with disk size: 79.9
Lane Width	4.83 mm

Lane 7

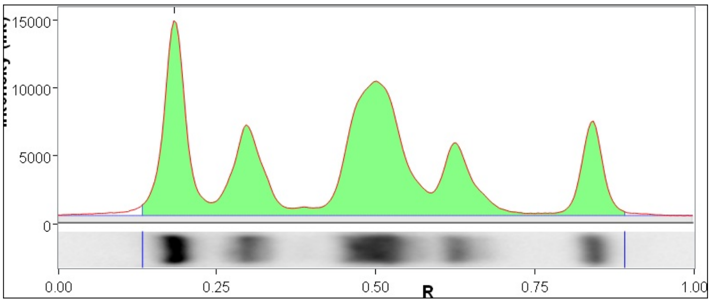

Band No.	Band Label	Mol. Wt. (KDa)	Relative Front	Adj. Volume (Int)	Volume (Int)	Abs. Quant.	Rel. Quant.	Band %	Lane %
1		N/A	0,186	32 179 920	37 230 728	N/A	N/A	100,0	98,7

Band Detection	Automatically detected bands with sensitivity: Low
Lane Background	Lane background subtracted with disk size: 79.9
Lane Width	4.96 mm

Lane 8

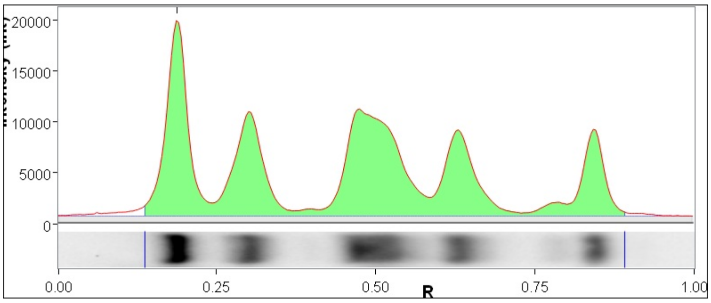

Band No.	Band Label	Mol. Wt. (KDa)	Relative Front	Adj. Volume (Int)	Volume (Int)	Abs. Quant.	Rel. Quant.	Band %	Lane %
1		N/A	0,189	32 562 812	37 590 224	N/A	N/A	100,0	98,5

Band Detection	Automatically detected bands with sensitivity: Low
Lane Background	Lane background subtracted with disk size: 79.9
Lane Width	4.83 mm

Lane 9

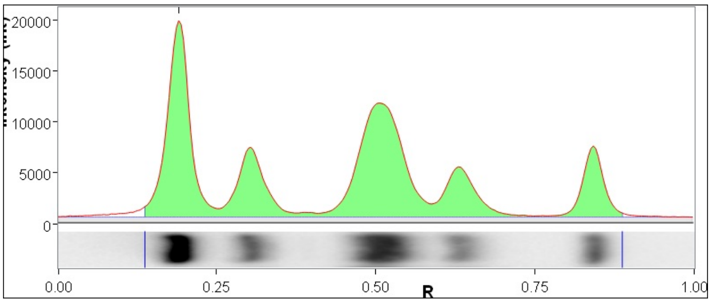

Band No.	Band Label	Mol. Wt. (KDa)	Relative Front	Adj. Volume (Int)	Volume (Int)	Abs. Quant.	Rel. Quant.	Band %	Lane %
1		N/A	0,193	31 485 888	36 555 088	N/A	N/A	100,0	98,4

Band Detection	Automatically detected bands with sensitivity: Low
Lane Background	Lane background subtracted with disk size: 79.9
Lane Width	4.96 mm

Lane 10

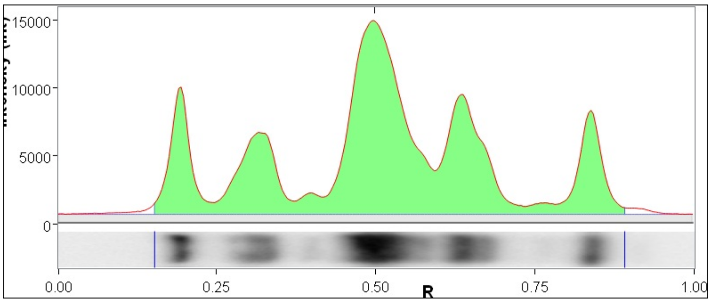

Band No.	Band Label	Mol. Wt. (KDa)	Relative Front	Adj. Volume (Int)	Volume (Int)	Abs. Quant.	Rel. Quant.	Band %	Lane %
1		N/A	0,500	34 318 240	39 260 182	N/A	N/A	100,0	98,8

Band Detection	Automatically detected bands with sensitivity: Low
Lane Background	Lane background subtracted with disk size: 79.9
Lane Width	4.83 mm

Lane 11

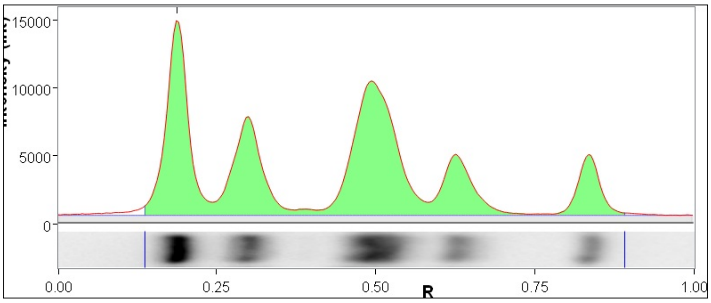

Band No.	Band Label	Mol. Wt. (KDa)	Relative Front	Adj. Volume (Int)	Volume (Int)	Abs. Quant.	Rel. Quant.	Band %	Lane %
1		N/A	0,189	27 763 242	32 984 016	N/A	N/A	100,0	98,7

Band Detection	Automatically detected bands with sensitivity: Low
Lane Background	Lane background subtracted with disk size: 79.9
Lane Width	5.09 mm

Lane 12

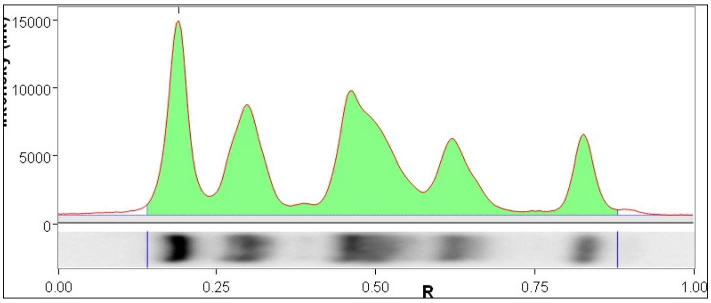

Band No.	Band Label	Mol. Wt. (KDa)	Relative Front	Adj. Volume (Int)	Volume (Int)	Abs. Quant.	Rel. Quant.	Band %	Lane %
1		N/A	0,193	32 439 487	37 697 614	N/A	N/A	100,0	98,3

Band Detection	Automatically detected bands with sensitivity: Low

Lane Background	Lane background subtracted with disk size: 79.9
Lane Width	5.35 mm

Image Report: Fig. 1c (Ponceau S staining)

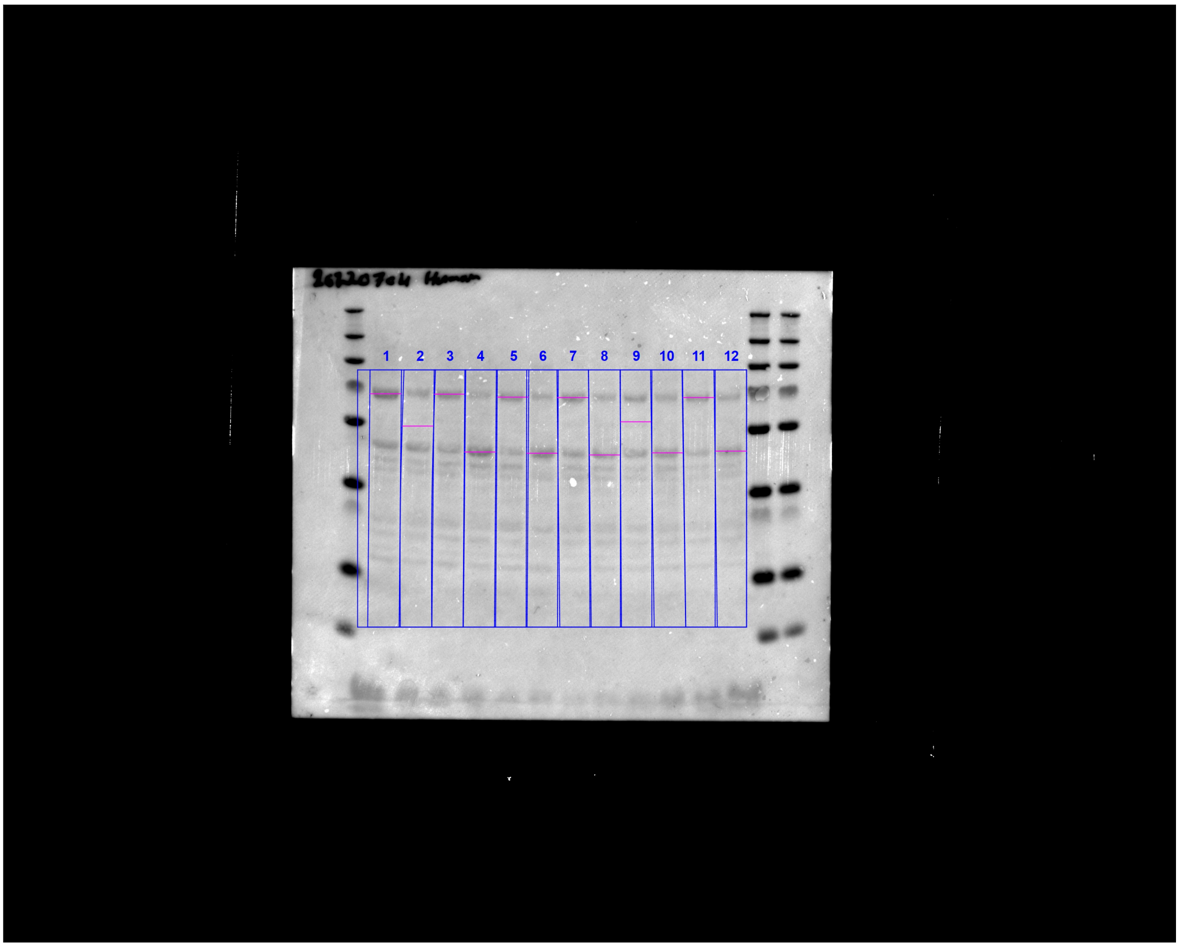

Acquisition Information

Imager	ChemiDoc Touch
Exposure Time (sec)	0.430 (Optimal Auto-exposure)
Serial Number	732BR0263
Software Version	2.3.0.07
Application	Colorimetric
Excitation Source	White Epi Illumination
Emission Filter	590/110 Filter

Image Information

Acquisition Date	7/5/2022 2:51:41
User Name	m
Image Area (mm)	X: 180.0 Y: 144.1
Pixel Size (µm)	X: 65.3 Y: 65.3
Data Range (Int)	0 - 65446

Analysis Settings

Detection	Lane detection:<br>Manually created lanes<br><br>Band detection:<br><br>Manually adjusted bands<br><br>Lane Background Subtraction:<br>Lane background subtracted with disk size: 19.3<br><br>Lane width: 4.83 mm

Lane Statistics

Lane No.	Adj. Total Band Vol. (Int)	Total Band Vol. (Int)	Adj. Total Lane Vol. (Int)	Total Lane Vol. (Int)	Bkgd. Vol. (Int)	Norm. Factor
1	14 681 156	1 727 393 766	14 681 156	1 727 393 766	1 712 712 610	N/A
2	10 883 994	1 719 169 702	10 883 994	1 719 169 702	1 708 285 708	N/A
3	10 444 730	1 722 149 386	10 444 730	1 722 149 386	1 711 704 656	N/A
4	12 882 438	1 724 588 870	12 882 438	1 724 588 870	1 711 706 432	N/A
5	11 506 630	1 720 502 442	11 506 630	1 720 502 442	1 708 995 812	N/A
6	10 877 556	1 719 197 230	10 877 556	1 719 197 230	1 708 319 674	N/A
7	13 422 268	1 716 009 310	13 422 268	1 716 009 310	1 702 587 042	N/A
8	12 989 960	1 713 433 962	12 989 960	1 713 433 962	1 700 444 002	N/A
9	9 521 728	1 710 942 012	9 521 728	1 710 942 012	1 701 420 284	N/A
10	9 731 962	1 715 047 384	9 731 962	1 715 047 384	1 705 315 422	N/A
11	7 722 344	1 710 223 842	7 722 344	1 710 223 842	1 702 501 498	N/A
12	10 662 364	1 715 353 744	10 662 364	1 715 353 744	1 704 691 380	N/A

Lane And Band Analysis

Lane 1

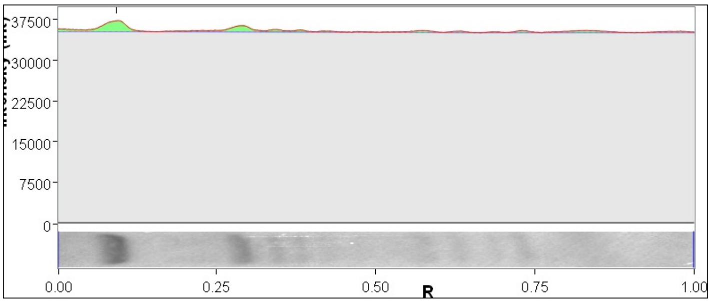

Band No.	Band Label	Mol. Wt. (KDa)	Relative Front	Adj. Volume (Int)	Volume (Int)	Abs. Quant.	Rel. Quant.	Band %	Lane %
1		N/A	0,093	14 681 156	1 727 393 766	N/A	N/A	100,0	100,0

Lane Background	Lane background subtracted with disk size: 19.3
Lane Width	4.83 mm

Lane 2

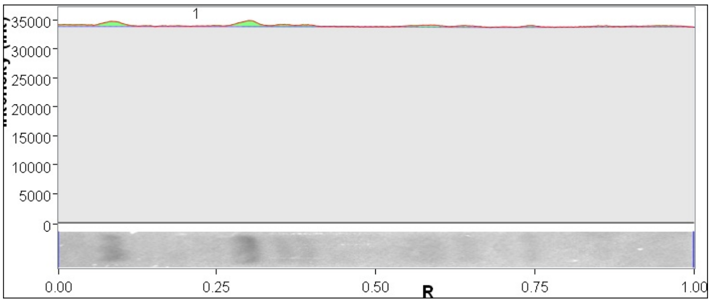

Band No.	Band Label	Mol. Wt. (KDa)	Relative Front	Adj. Volume (Int)	Volume (Int)	Abs. Quant.	Rel. Quant.	Band %	Lane %
1		N/A	0,218	10 883 994	1 719 169 702	N/A	N/A	100,0	100,0

Lane Background	Lane background subtracted with disk size: 19.3
Lane Width	4.83 mm

Lane 3

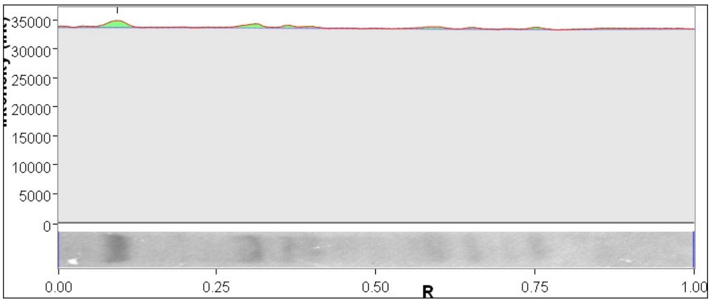

Band No.	Band Label	Mol. Wt. (KDa)	Relative Front	Adj. Volume (Int)	Volume (Int)	Abs. Quant.	Rel. Quant.	Band %	Lane %
1		N/A	0,094	10 444 730	1 722 149 386	N/A	N/A	100,0	100,0

Lane Background	Lane background subtracted with disk size: 19.3
Lane Width	4.83 mm

Lane 4

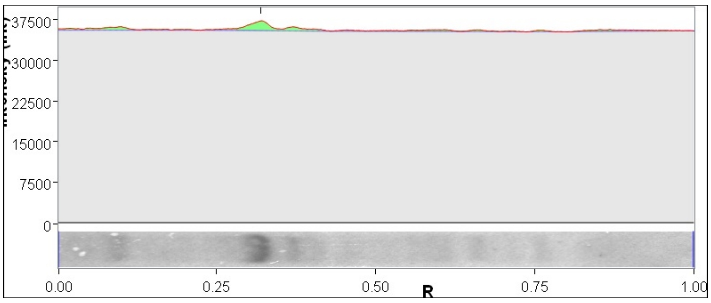

Band No.	Band Label	Mol. Wt. (KDa)	Relative Front	Adj. Volume (Int)	Volume (Int)	Abs. Quant.	Rel. Quant.	Band %	Lane %
1		N/A	0,319	12 882 438	1 724 588 870	N/A	N/A	100,0	100,0

Lane Background	Lane background subtracted with disk size: 19.3
Lane Width	4.83 mm

Lane 5

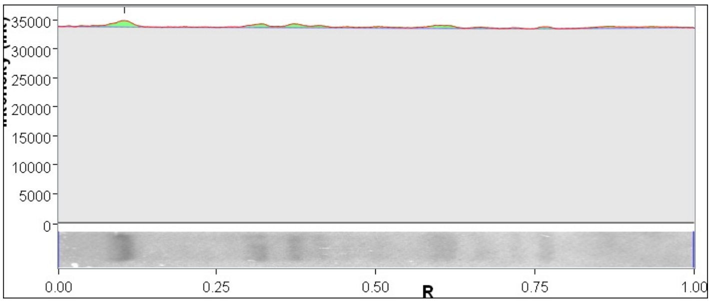

Band No.	Band Label	Mol. Wt. (KDa)	Relative Front	Adj. Volume (Int)	Volume (Int)	Abs. Quant.	Rel. Quant.	Band %	Lane %
1		N/A	0,106	11 506 630	1 720 502 442	N/A	N/A	100,0	100,0

Lane Background	Lane background subtracted with disk size: 19.3
Lane Width	4.83 mm

Lane 6

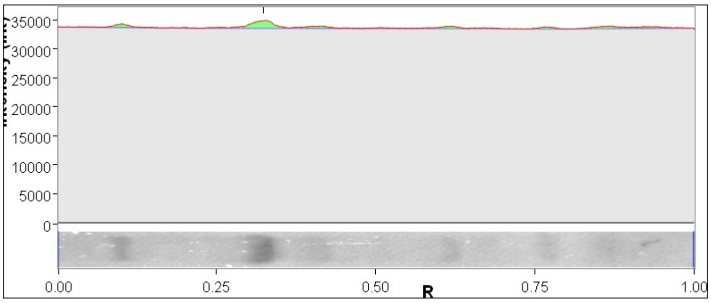

Band No.	Band Label	Mol. Wt. (KDa)	Relative Front	Adj. Volume (Int)	Volume (Int)	Abs. Quant.	Rel. Quant.	Band %	Lane %
1		N/A	0,324	10 877 556	1 719 197 230	N/A	N/A	100,0	100,0

Lane Background	Lane background subtracted with disk size: 19.3
Lane Width	4.83 mm

Lane 7

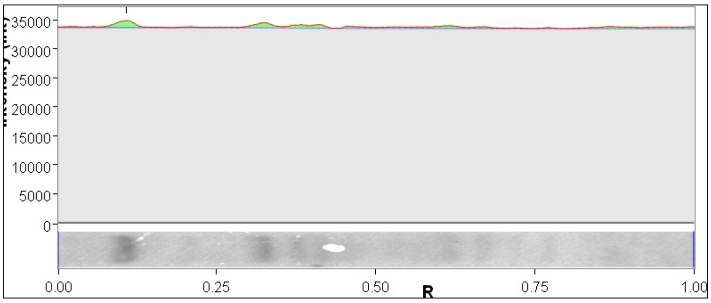

Band No.	Band Label	Mol. Wt. (KDa)	Relative Front	Adj. Volume (Int)	Volume (Int)	Abs. Quant.	Rel. Quant.	Band %	Lane %
1		N/A	0,107	13 422 268	1 716 009 310	N/A	N/A	100,0	100,0

Lane Background	Lane background subtracted with disk size: 19.3
Lane Width	4.83 mm

Lane 8

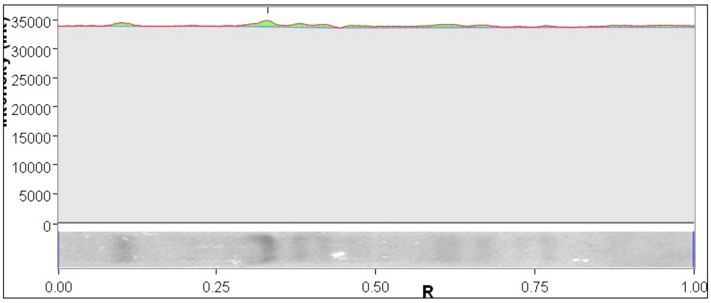

Band No.	Band Label	Mol. Wt. (KDa)	Relative Front	Adj. Volume (Int)	Volume (Int)	Abs. Quant.	Rel. Quant.	Band %	Lane %
1		N/A	0,331	12 989 960	1 713 433 962	N/A	N/A	100,0	100,0

Lane Background	Lane background subtracted with disk size: 19.3
Lane Width	4.83 mm

Lane 9

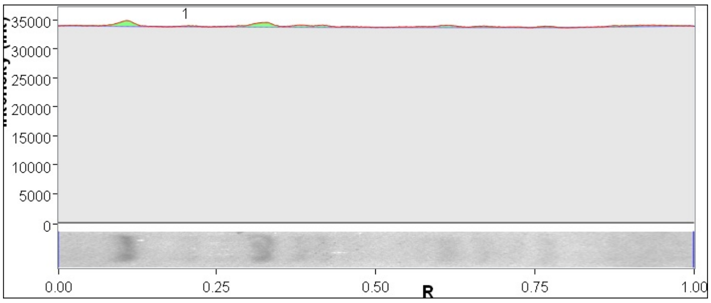

Band No.	Band Label	Mol. Wt. (KDa)	Relative Front	Adj. Volume (Int)	Volume (Int)	Abs. Quant.	Rel. Quant.	Band %	Lane %
1		N/A	0,202	9 521 728	1 710 942 012	N/A	N/A	100,0	100,0

Lane Background	Lane background subtracted with disk size: 19.3
Lane Width	4.83 mm

Lane 10

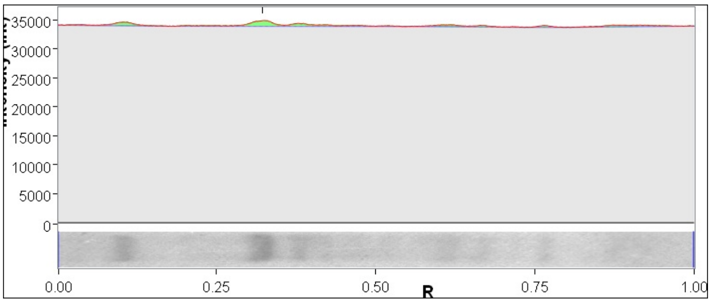

Band No.	Band Label	Mol. Wt. (KDa)	Relative Front	Adj. Volume (Int)	Volume (Int)	Abs. Quant.	Rel. Quant.	Band %	Lane %
1		N/A	0,322	9 731 962	1 715 047 384	N/A	N/A	100,0	100,0

Lane Background	Lane background subtracted with disk size: 19.3
Lane Width	4.83 mm

Lane 11

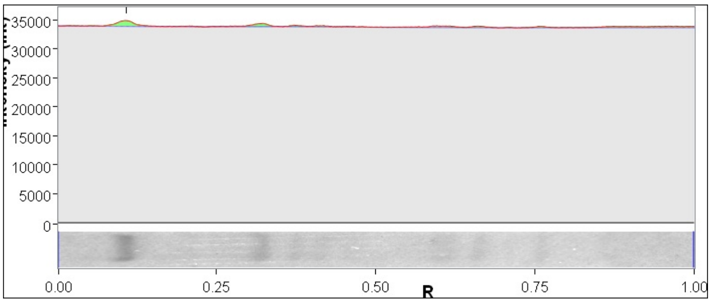

Band No.	Band Label	Mol. Wt. (KDa)	Relative Front	Adj. Volume (Int)	Volume (Int)	Abs. Quant.	Rel. Quant.	Band %	Lane %
1		N/A	0,107	7 722 344	1 710 223 842	N/A	N/A	100,0	100,0

Lane Background	Lane background subtracted with disk size: 19.3
Lane Width	4.83 mm

Lane 12

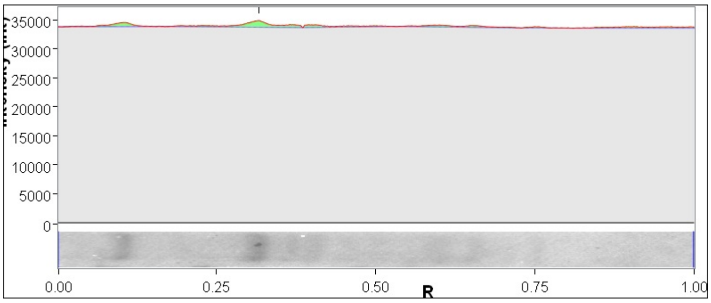

Band No.	Band Label	Mol. Wt. (KDa)	Relative Front	Adj. Volume (Int)	Volume (Int)	Abs. Quant.	Rel. Quant.	Band %	Lane %
1		N/A	0,316	10 662 364	1 715 353 744	N/A	N/A	100,0	100,0

Lane Background	Lane background subtracted with disk size: 19.3
Lane Width	4.83 mm

Image Report: Intensity analysis of Idh2 panel in Fig. 3g

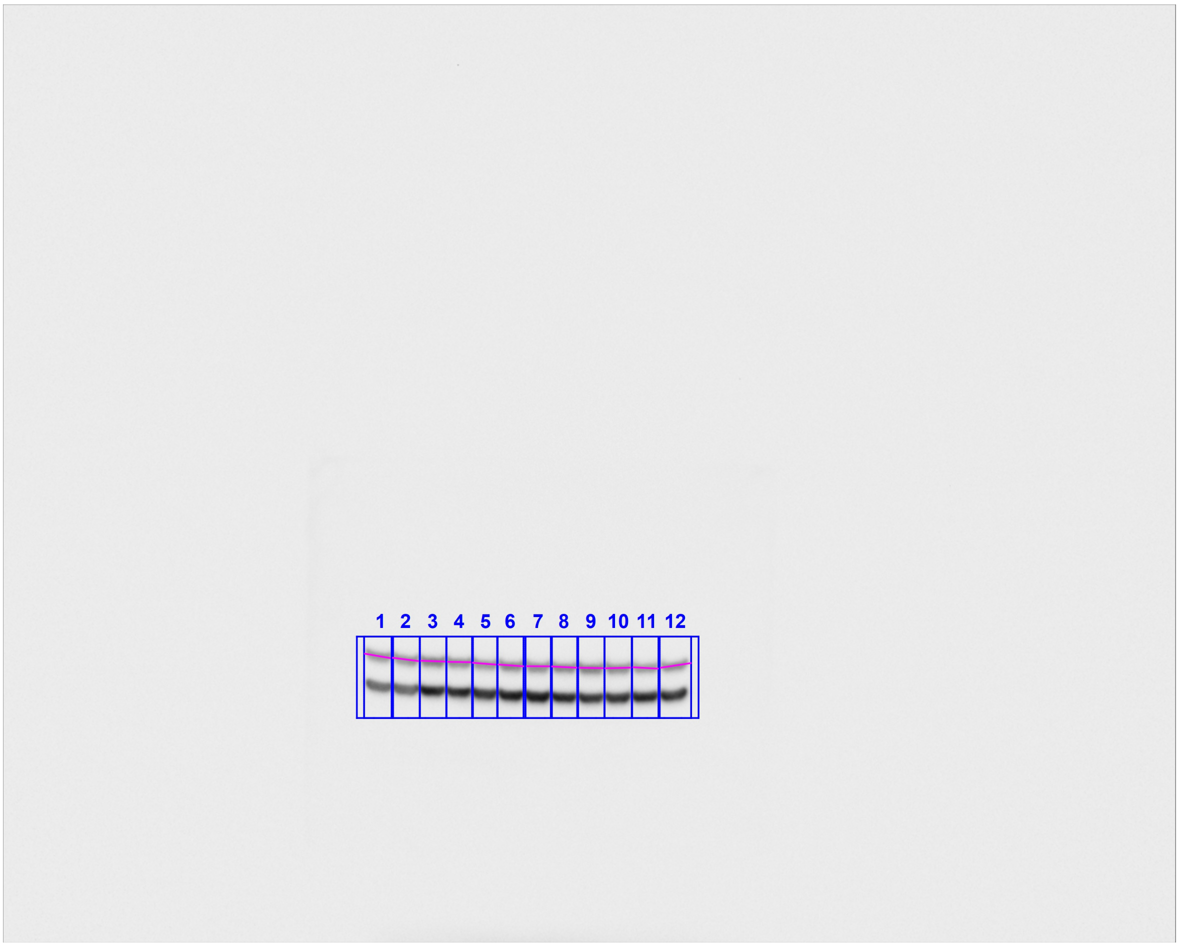

Acquisition Information

Imager	ChemiDoc Touch
Exposure Time (sec)	1.000 (Signal Accumulation)
Serial Number	732BR1240
Software Version	1.1.0.04
Application	Chemiluminescence
Excitation Source	No Illumination
Emission Filter	No Filter
Binning	2x2

Image Information

Acquisition Date	3/17/2021 4:00:40
User Name	
Image Area (mm)	X: 210.0 Y: 168.1
Pixel Size (µm)	X: 152.3 Y: 152.3
Data Range (Int)	500 - 19534

Analysis Settings

Detection	Lane detection:<br>Manually created lanes (Copied)<br><br>Band detection:<br>Automatically detected bands with sensitivity: Low<br>Manually adjusted bands<br><br>Lane Background Subtraction:<br>Lane background subtracted with disk size: 59.7<br><br>Lane width: Variable

Lane Statistics

Lane No.	Adj. Total Band Vol. (Int)	Total Band Vol. (Int)	Adj. Total Lane Vol. (Int)	Total Lane Vol. (Int)	Bkgd. Vol. (Int)	Norm. Factor
1	1 985 362	2 505 392	5 586 506	7 757 066	2 170 560	N/A
2	2 004 354	2 530 341	5 924 721	8 120 145	2 195 424	N/A
3	2 496 523	3 003 931	8 226 532	10 256 164	2 029 632	N/A
4	2 098 917	2 595 878	7 634 556	9 708 828	2 074 272	N/A
5	1 910 610	2 409 570	7 488 930	9 484 770	1 995 840	N/A
6	2 284 018	2 789 194	8 392 134	10 412 838	2 020 704	N/A
7	2 172 697	2 683 081	8 623 890	10 665 426	2 041 536	N/A
8	2 231 349	2 730 573	7 858 128	9 855 024	1 996 896	N/A
9	2 560 290	3 085 740	7 622 621	9 640 349	2 017 728	N/A
10	2 294 912	2 814 848	7 755 232	9 834 976	2 079 744	N/A
11	1 786 468	2 256 366	7 551 538	9 602 002	2 050 464	N/A
12	2 137 120	2 721 757	7 901 979	10 342 203	2 440 224	N/A

Lane And Band Analysis

Lane 1

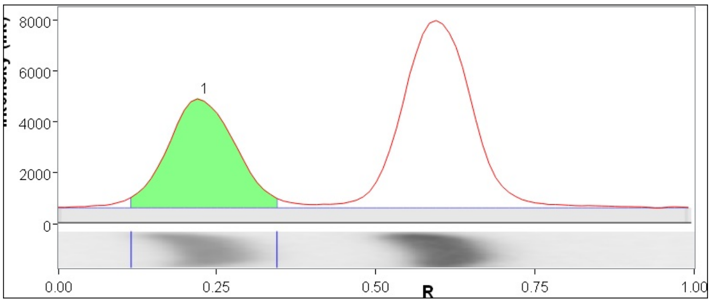

Band No.	Band Label	Mol. Wt. (KDa)	Relative Front	Adj. Volume (Int)	Volume (Int)	Abs. Quant.	Rel. Quant.	Band %	Lane %
1		N/A	0,240	1 985 362	2 505 392	N/A	N/A	100,0	35,5

Band Detection	Automatically detected bands with sensitivity: Low
Lane Background	Lane background subtracted with disk size: 59.7
Lane Width	5.18 mm

Lane 2

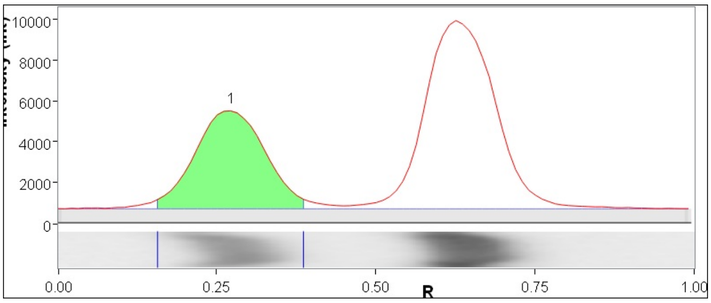

Band No.	Band Label	Mol. Wt. (KDa)	Relative Front	Adj. Volume (Int)	Volume (Int)	Abs. Quant.	Rel. Quant.	Band %	Lane %
1		N/A	0,281	2 004 354	2 530 341	N/A	N/A	100,0	33,8

Band Detection	Automatically detected bands with sensitivity: Low
Lane Background	Lane background subtracted with disk size: 59.7
Lane Width	5.03 mm

Lane 3

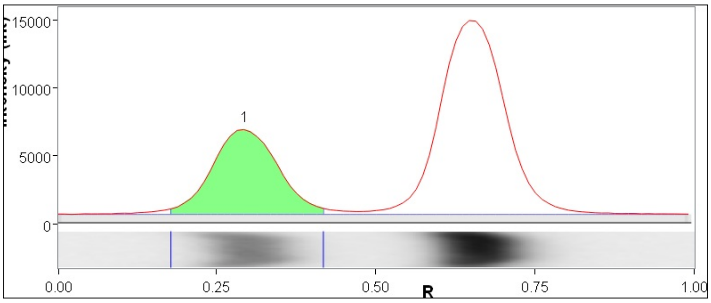

Band No.	Band Label	Mol. Wt. (KDa)	Relative Front	Adj. Volume (Int)	Volume (Int)	Abs. Quant.	Rel. Quant.	Band %	Lane %
1		N/A	0,302	2 496 523	3 003 931	N/A	N/A	100,0	30,3

Band Detection	Automatically detected bands with sensitivity: Low
Lane Background	Lane background subtracted with disk size: 59.7
Lane Width	4.72 mm

Lane 4

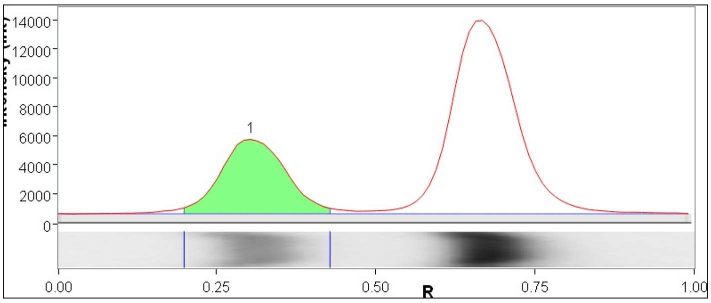

Band No.	Band Label	Mol. Wt. (KDa)	Relative Front	Adj. Volume (Int)	Volume (Int)	Abs. Quant.	Rel. Quant.	Band %	Lane %
1		N/A	0,313	2 098 917	2 595 878	N/A	N/A	100,0	27,5

Band Detection	Automatically detected bands with sensitivity: Low

Lane Background	Lane background subtracted with disk size: 59.7
Lane Width	4.72 mm

Lane 5

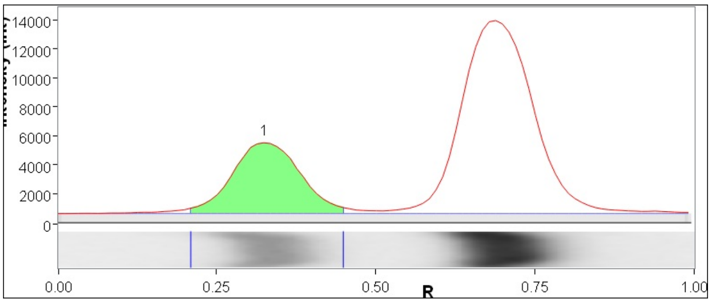

Band No.	Band Label	Mol. Wt. (KDa)	Relative Front	Adj. Volume (Int)	Volume (Int)	Abs. Quant.	Rel. Quant.	Band %	Lane %
1		N/A	0,333	1 910 610	2 409 570	N/A	N/A	100,0	25,5

Band Detection	Automatically detected bands with sensitivity: Low
Lane Background	Lane background subtracted with disk size: 59.7
Lane Width	4.57 mm

Lane 6

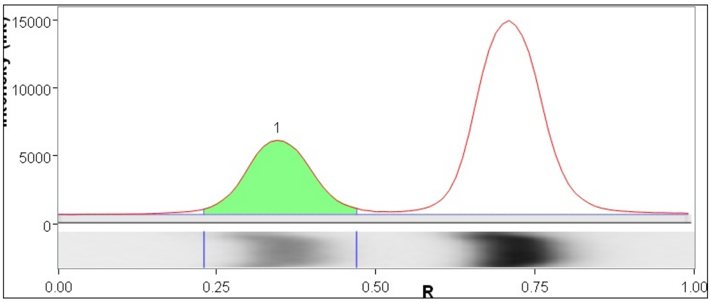

Band No.	Band Label	Mol. Wt. (KDa)	Relative Front	Adj. Volume (Int)	Volume (Int)	Abs. Quant.	Rel. Quant.	Band %	Lane %
1		N/A	0,354	2 284 018	2 789 194	N/A	N/A	100,0	27,2

Band Detection	Automatically detected bands with sensitivity: Low
Lane Background	Lane background subtracted with disk size: 59.7
Lane Width	4.72 mm

Lane 7

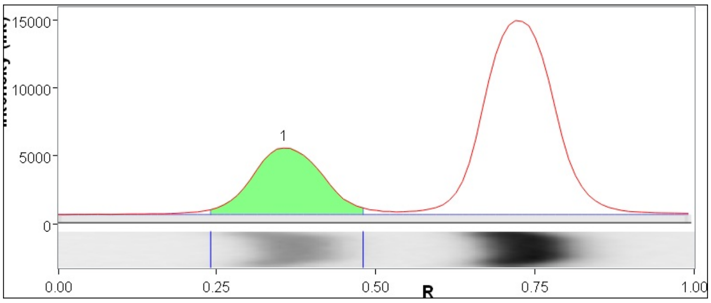

Band No.	Band Label	Mol. Wt. (KDa)	Relative Front	Adj. Volume (Int)	Volume (Int)	Abs. Quant.	Rel. Quant.	Band %	Lane %
1		N/A	0,365	2 172 697	2 683 081	N/A	N/A	100,0	25,2

Band Detection	Automatically detected bands with sensitivity: Low
Lane Background	Lane background subtracted with disk size: 59.7
Lane Width	4.72 mm

Lane 8

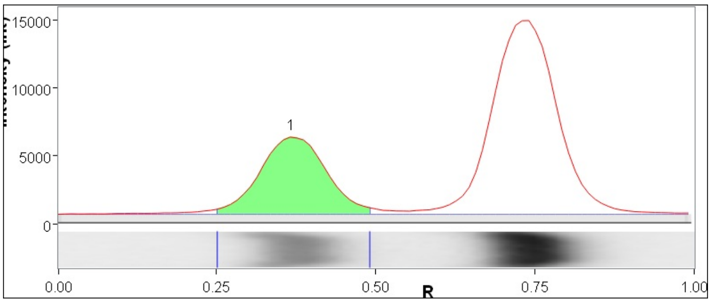

Band No.	Band Label	Mol. Wt. (KDa)	Relative Front	Adj. Volume (Int)	Volume (Int)	Abs. Quant.	Rel. Quant.	Band %	Lane %
1		N/A	0,375	2 231 349	2 730 573	N/A	N/A	100,0	28,4

Band Detection	Automatically detected bands with sensitivity: Low
Lane Background	Lane background subtracted with disk size: 59.7
Lane Width	4.72 mm

Lane 9

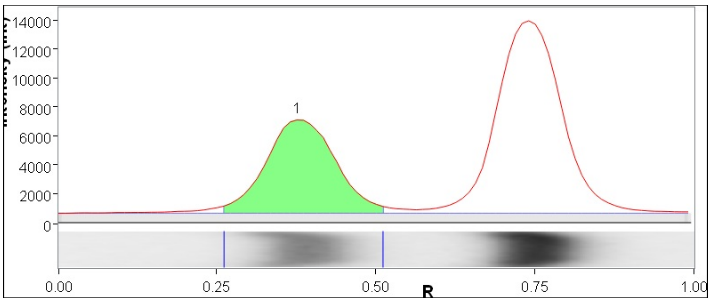

Band No.	Band Label	Mol. Wt. (KDa)	Relative Front	Adj. Volume (Int)	Volume (Int)	Abs. Quant.	Rel. Quant.	Band %	Lane %
1		N/A	0,385	2 560 290	3 085 740	N/A	N/A	100,0	33,6

Band Detection	Automatically detected bands with sensitivity: Low
Lane Background	Lane background subtracted with disk size: 59.7
Lane Width	4.72 mm

Lane 10

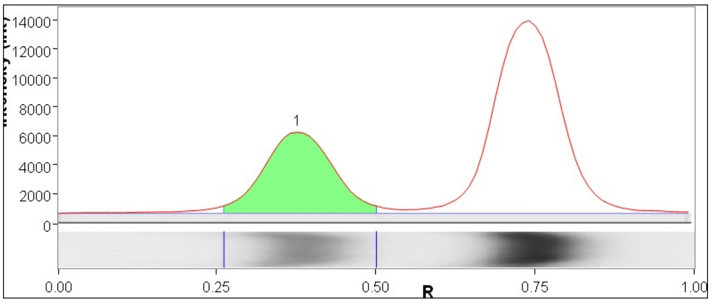

Band No.	Band Label	Mol. Wt. (KDa)	Relative Front	Adj. Volume (Int)	Volume (Int)	Abs. Quant.	Rel. Quant.	Band %	Lane %
1		N/A	0,385	2 294 912	2 814 848	N/A	N/A	100,0	29,6

Band Detection	Automatically detected bands with sensitivity: Low
Lane Background	Lane background subtracted with disk size: 59.7
Lane Width	4.87 mm

Lane 11

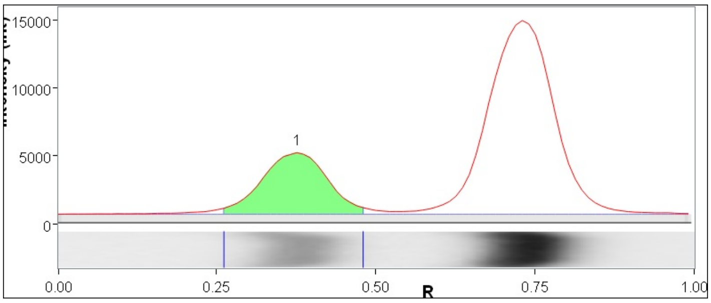

Band No.	Band Label	Mol. Wt. (KDa)	Relative Front	Adj. Volume (Int)	Volume (Int)	Abs. Quant.	Rel. Quant.	Band %	Lane %
1		N/A	0,385	1 786 468	2 256 366	N/A	N/A	100,0	23,7

Band Detection	Automatically detected bands with sensitivity: Low
Lane Background	Lane background subtracted with disk size: 59.7
Lane Width	4.72 mm

Lane 12

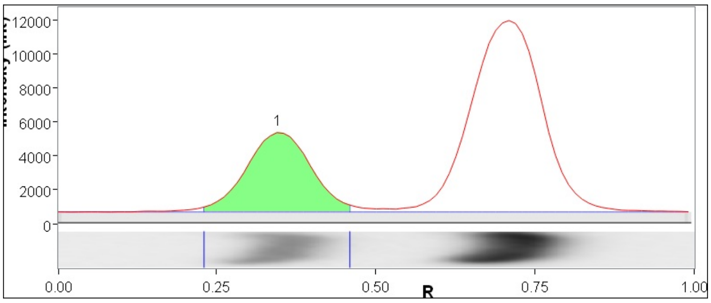

Band No.	Band Label	Mol. Wt. (KDa)	Relative Front	Adj. Volume (Int)	Volume (Int)	Abs. Quant.	Rel. Quant.	Band %	Lane %
1		N/A	0,354	2 137 120	2 721 757	N/A	N/A	100,0	27,0

Band Detection	Automatically detected bands with sensitivity: Low

Lane Background	Lane background subtracted with disk size: 59.7
Lane Width	5.63 mm

Image Report: Intensity analysis of β-actin panel in Fig. 3g

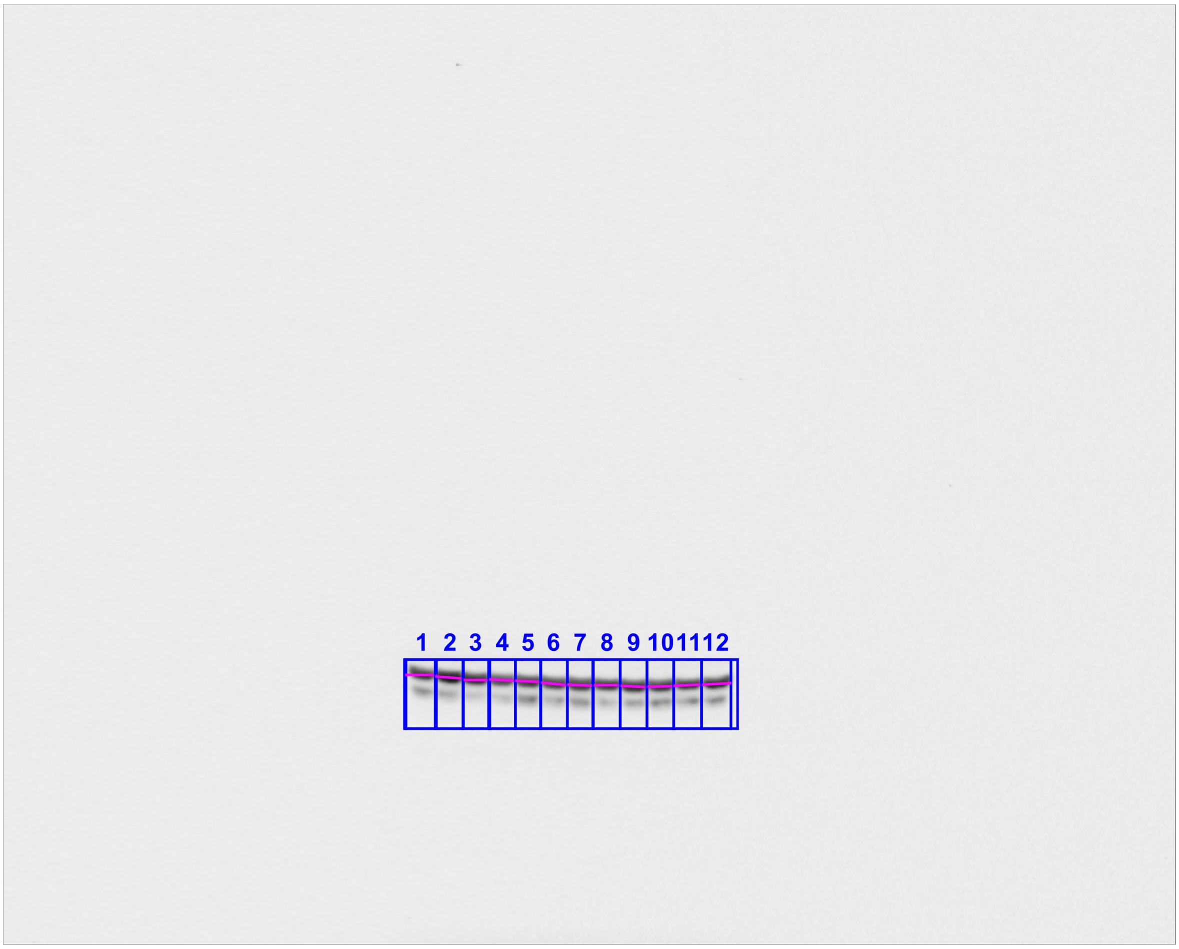

Acquisition Information

Imager	ChemiDoc Touch
Exposure Time (sec)	2.000 (Signal Accumulation)
Serial Number	732BR1240
Software Version	1.1.0.04
Application	Chemiluminescence
Excitation Source	No Illumination
Emission Filter	No Filter
Binning	3x3

Image Information

Acquisition Date	3/25/2021 10:45:04
User Name	
Image Area (mm)	X: 210.0 Y: 168.2
Pixel Size (µm)	X: 228.5 Y: 228.5
Data Range (Int)	500 - 19039

Analysis Settings

Detection	Lane detection:<br>Manually created lanes<br><br>Band detection:<br>Automatically detected bands with sensitivity: Low<br>Manually adjusted bands<br><br>Lane Background Subtraction:<br>Lane background subtracted with disk size: 59.7<br><br>Lane width: Variable

Lane Statistics

Lane No.	Adj. Total Band Vol. (Int)	Total Band Vol. (Int)	Adj. Total Lane Vol. (Int)	Total Lane Vol. (Int)	Bkgd. Vol. (Int)	Norm. Factor
1	2 260 775	2 556 700	2 937 300	3 778 350	841 050	N/A
2	3 089 702	3 398 010	3 674 000	4 430 756	756 756	N/A
3	2 481 234	2 740 185	2 841 804	3 577 770	735 966	N/A
4	1 933 029	2 179 863	2 365 104	3 105 606	740 502	N/A
5	2 114 020	2 364 580	2 987 400	3 739 080	751 680	N/A
6	2 141 853	2 407 965	2 726 724	3 525 060	798 336	N/A
7	2 271 800	2 524 160	3 001 880	3 758 960	757 080	N/A
8	2 246 412	2 519 727	2 696 295	3 473 085	776 790	N/A
9	2 466 135	2 739 849	3 029 082	3 807 006	777 924	N/A
10	2 274 825	2 539 047	3 056 718	3 849 384	792 666	N/A
11	2 146 826	2 411 354	2 863 718	3 657 302	793 584	N/A
12	2 476 939	2 747 695	3 185 684	3 997 952	812 268	N/A

Lane And Band Analysis

Lane 1

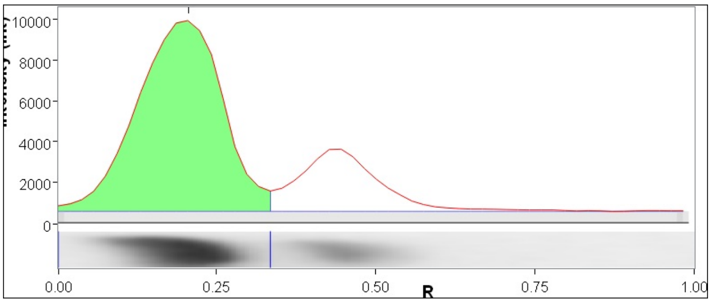

Band No.	Band Label	Mol. Wt. (KDa)	Relative Front	Adj. Volume (Int)	Volume (Int)	Abs. Quant.	Rel. Quant.	Band %	Lane %
1		N/A	0,222	2 260 775	2 556 700	N/A	N/A	100,0	77,0

Band Detection	Automatically detected bands with sensitivity: Low
Lane Background	Lane background subtracted with disk size: 59.7
Lane Width	5.71 mm

Lane 2

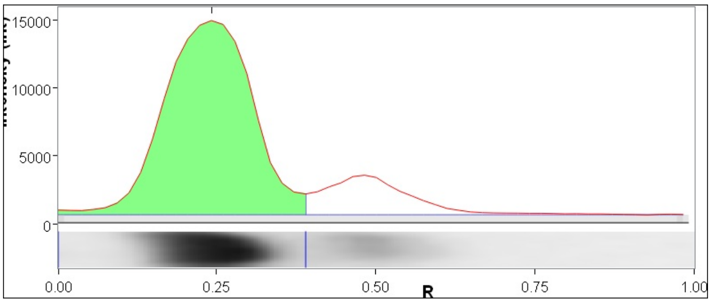

Band No.	Band Label	Mol. Wt. (KDa)	Relative Front	Adj. Volume (Int)	Volume (Int)	Abs. Quant.	Rel. Quant.	Band %	Lane %
1		N/A	0,259	3 089 702	3 398 010	N/A	N/A	100,0	84,1

Band Detection	Automatically detected bands with sensitivity: Low
Lane Background	Lane background subtracted with disk size: 59.7
Lane Width	5.03 mm

Lane 3

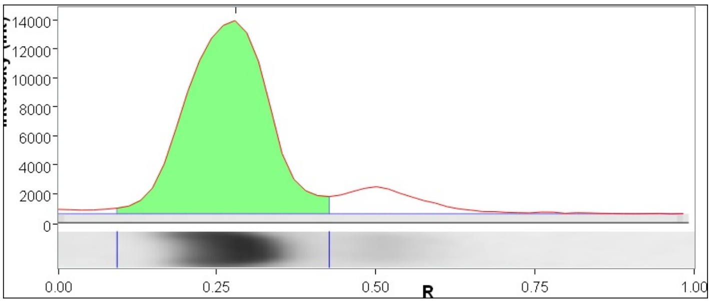

Band No.	Band Label	Mol. Wt. (KDa)	Relative Front	Adj. Volume (Int)	Volume (Int)	Abs. Quant.	Rel. Quant.	Band %	Lane %
1		N/A	0,296	2 481 234	2 740 185	N/A	N/A	100,0	87,3

Band Detection	Automatically detected bands with sensitivity: Low
Lane Background	Lane background subtracted with disk size: 59.7
Lane Width	4.80 mm

Lane 4

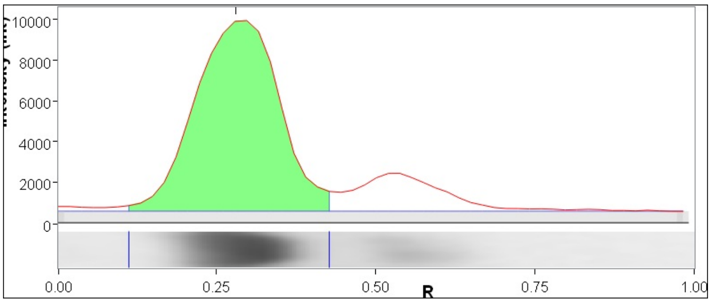

Band No.	Band Label	Mol. Wt. (KDa)	Relative Front	Adj. Volume (Int)	Volume (Int)	Abs. Quant.	Rel. Quant.	Band %	Lane %
1		N/A	0,296	1 933 029	2 179 863	N/A	N/A	100,0	81,7

Band Detection	Automatically detected bands with sensitivity: Low

Lane Background	Lane background subtracted with disk size: 59.7
Lane Width	4.80 mm

Lane 5

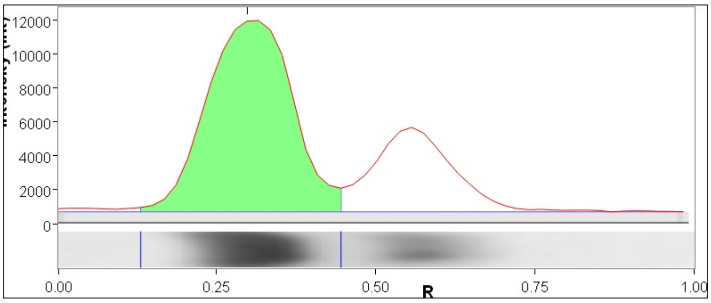

Band No.	Band Label	Mol. Wt. (KDa)	Relative Front	Adj. Volume (Int)	Volume (Int)	Abs. Quant.	Rel. Quant.	Band %	Lane %
1		N/A	0,315	2 114 020	2 364 580	N/A	N/A	100,0	70,8

Band Detection	Automatically detected bands with sensitivity: Low
Lane Background	Lane background subtracted with disk size: 59.7
Lane Width	4.57 mm

Lane 6

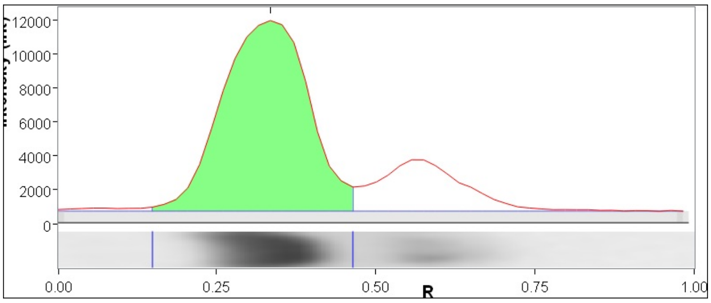

Band No.	Band Label	Mol. Wt. (KDa)	Relative Front	Adj. Volume (Int)	Volume (Int)	Abs. Quant.	Rel. Quant.	Band %	Lane %
1		N/A	0,352	2 141 853	2 407 965	N/A	N/A	100,0	78,6

Band Detection	Automatically detected bands with sensitivity: Low
Lane Background	Lane background subtracted with disk size: 59.7
Lane Width	4.80 mm

Lane 7

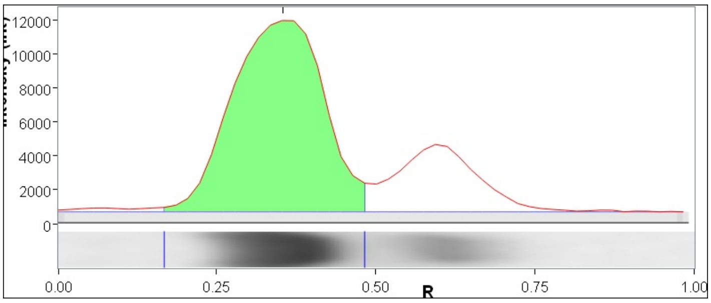

Band No.	Band Label	Mol. Wt. (KDa)	Relative Front	Adj. Volume (Int)	Volume (Int)	Abs. Quant.	Rel. Quant.	Band %	Lane %
1		N/A	0,370	2 271 800	2 524 160	N/A	N/A	100,0	75,7

Band Detection	Automatically detected bands with sensitivity: Low
Lane Background	Lane background subtracted with disk size: 59.7
Lane Width	4.57 mm

Lane 8

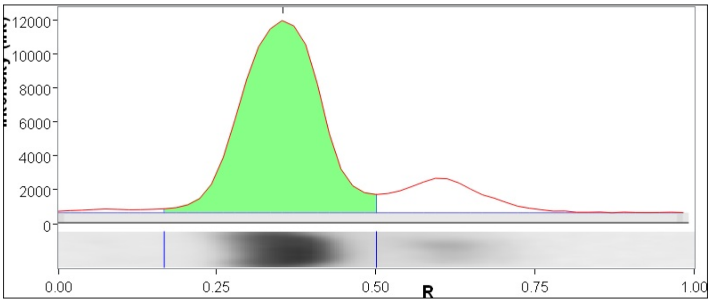

Band No.	Band Label	Mol. Wt. (KDa)	Relative Front	Adj. Volume (Int)	Volume (Int)	Abs. Quant.	Rel. Quant.	Band %	Lane %
1		N/A	0,370	2 246 412	2 519 727	N/A	N/A	100,0	83,3

Band Detection	Automatically detected bands with sensitivity: Low
Lane Background	Lane background subtracted with disk size: 59.7
Lane Width	4.80 mm

Lane 9

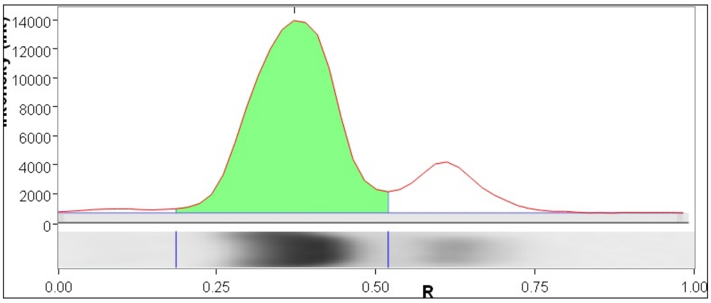

Band No.	Band Label	Mol. Wt. (KDa)	Relative Front	Adj. Volume (Int)	Volume (Int)	Abs. Quant.	Rel. Quant.	Band %	Lane %
1		N/A	0,389	2 466 135	2 739 849	N/A	N/A	100,0	81,4

Band Detection	Automatically detected bands with sensitivity: Low
Lane Background	Lane background subtracted with disk size: 59.7
Lane Width	4.80 mm

Lane 10

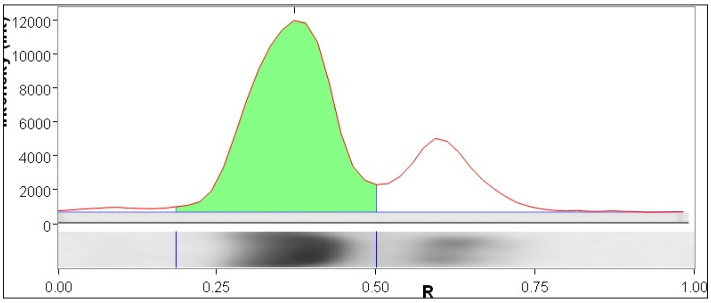

Band No.	Band Label	Mol. Wt. (KDa)	Relative Front	Adj. Volume (Int)	Volume (Int)	Abs. Quant.	Rel. Quant.	Band %	Lane %
1		N/A	0,389	2 274 825	2 539 047	N/A	N/A	100,0	74,4

Band Detection	Automatically detected bands with sensitivity: Low
Lane Background	Lane background subtracted with disk size: 59.7
Lane Width	4.80 mm

Lane 11

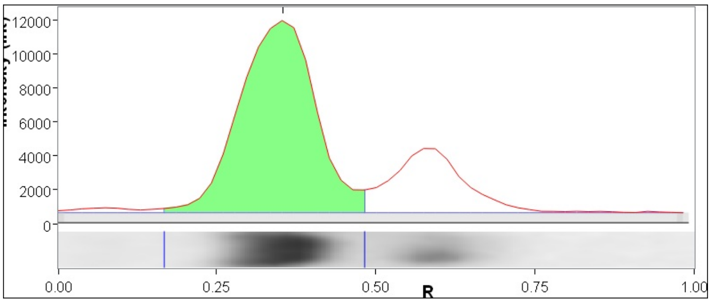

Band No.	Band Label	Mol. Wt. (KDa)	Relative Front	Adj. Volume (Int)	Volume (Int)	Abs. Quant.	Rel. Quant.	Band %	Lane %
1		N/A	0,370	2 146 826	2 411 354	N/A	N/A	100,0	75,0

Band Detection	Automatically detected bands with sensitivity: Low
Lane Background	Lane background subtracted with disk size: 59.7
Lane Width	5.03 mm

Lane 12

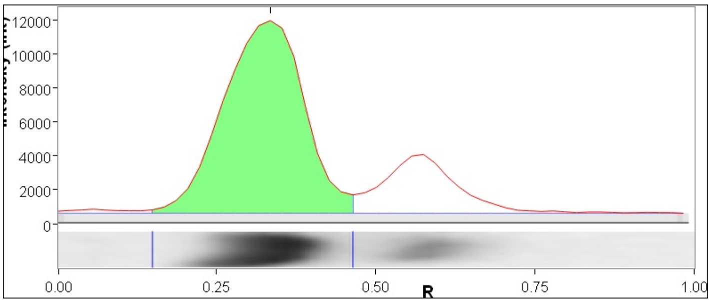

Band No.	Band Label	Mol. Wt. (KDa)	Relative Front	Adj. Volume (Int)	Volume (Int)	Abs. Quant.	Rel. Quant.	Band %	Lane %
1		N/A	0,352	2 476 939	2 747 695	N/A	N/A	100,0	77,8

Band Detection	Automatically detected bands with sensitivity: Low

Lane Background	Lane background subtracted with disk size: 59.7
Lane Width	5.26 mm

Image Report: Fig. 4g, Hmox1 panel, males

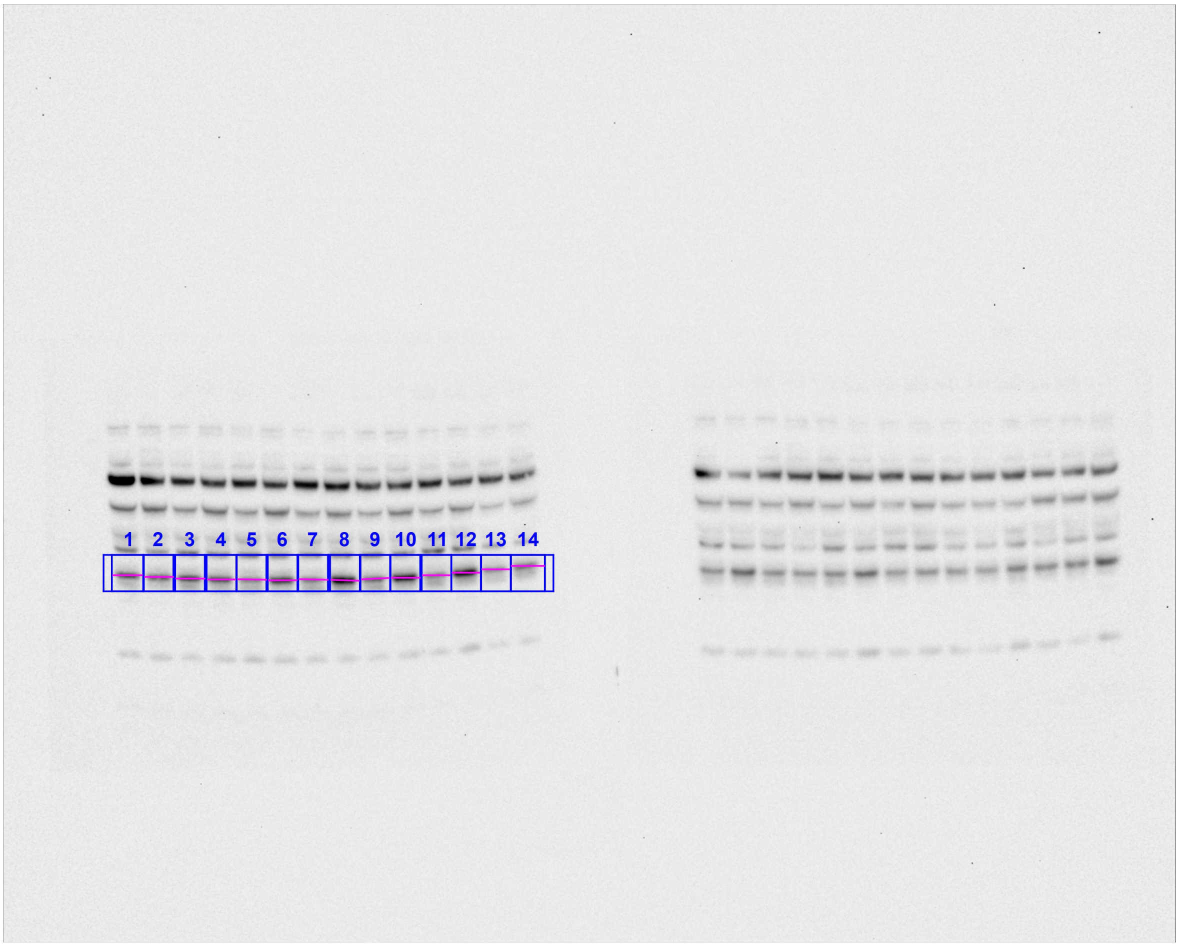

Acquisition Information

Imager	ChemiDoc Touch
Exposure Time (sec)	308.380 (Signal Accumulation)
Serial Number	732BR0263
Software Version	2.3.0.07
Application	Chemiluminescence
Excitation Source	No Illumination
Emission Filter	No Filter
Binning	2x2

Image Information

Acquisition Date	10/6/2022 9:49:57
User Name	m
Image Area (mm)	X: 185.6 Y: 148.6
Pixel Size (µm)	X: 134.6 Y: 134.6

Data Range (Int)	500 - 50404

Analysis Settings

Detection	Lane detection:<br>Manually created lanes (Copied)<br><br>Band detection:<br>Automatically detected bands with sensitivity: Low<br>Manually adjusted bands<br><br>Lane Background Subtraction:<br>Lane background subtracted with disk size: 24.1<br><br>Lane width: Variable

Lane Statistics

Lane No.	Adj. Total Band Vol. (Int)	Total Band Vol. (Int)	Adj. Total Lane Vol. (Int)	Total Lane Vol. (Int)	Bkgd. Vol. (Int)	Norm. Factor
1	2 570 982	3 258 960	3 144 926	4 788 429	1 643 503	N/A
2	2 449 825	3 276 035	2 791 775	4 406 640	1 614 865	N/A
3	2 945 950	3 650 850	3 721 760	5 317 060	1 595 300	N/A
4	2 941 015	3 779 650	3 585 015	5 302 220	1 717 205	N/A
5	1 581 440	2 170 490	2 763 040	4 451 650	1 688 610	N/A
6	3 355 520	4 122 230	4 226 495	6 058 080	1 831 585	N/A
7	1 626 450	2 186 625	2 799 545	4 405 380	1 605 835	N/A
8	4 972 380	5 666 150	6 092 555	7 847 385	1 754 830	N/A
9	2 099 790	2 846 340	3 065 755	4 849 180	1 783 425	N/A
10	4 459 805	5 316 325	5 079 865	7 018 305	1 938 440	N/A
11	1 601 635	2 181 760	2 444 785	4 107 810	1 663 025	N/A
12	4 605 930	5 177 725	5 475 610	6 921 915	1 446 305	N/A
13	1 419 810	1 839 250	2 088 100	3 376 380	1 288 280	N/A
14	2 187 600	2 771 720	2 769 960	4 247 440	1 477 480	N/A

Lane And Band Analysis

Lane 1

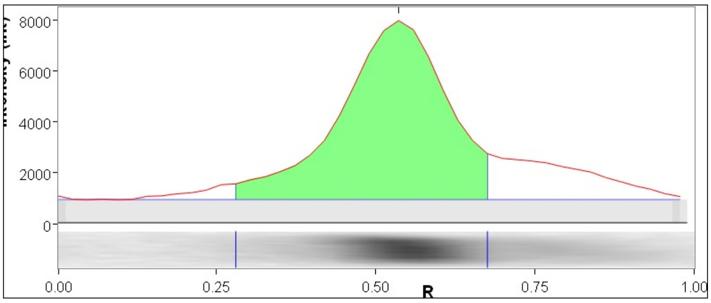

Band No.	Band Label	Mol. Wt. (KDa)	Relative Front	Adj. Volume (Int)	Volume (Int)	Abs. Quant.	Rel. Quant.	Band %	Lane %
1		N/A	0,558	2 570 982	3 258 960	N/A	N/A	100,0	81,8

Band Detection	Automatically detected bands with sensitivity: Low
Lane Background	Lane background subtracted with disk size: 24.1
Lane Width	4.98 mm

Lane 2

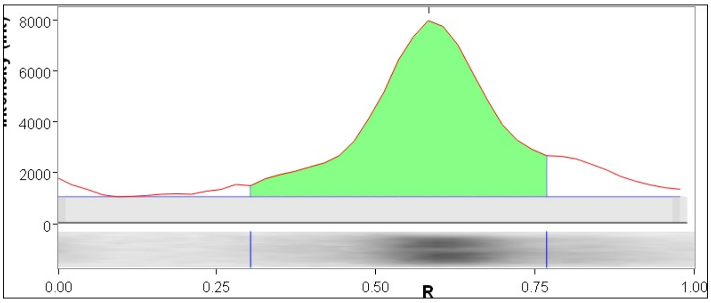

Band No.	Band Label	Mol. Wt. (KDa)	Relative Front	Adj. Volume (Int)	Volume (Int)	Abs. Quant.	Rel. Quant.	Band %	Lane %
1		N/A	0,605	2 449 825	3 276 035	N/A	N/A	100,0	87,8

Band Detection	Automatically detected bands with sensitivity: Low
Lane Background	Lane background subtracted with disk size: 24.1
Lane Width	4.71 mm

Lane 3

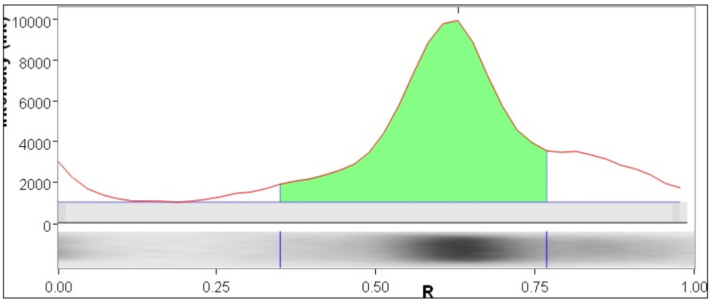

Band No.	Band Label	Mol. Wt. (KDa)	Relative Front	Adj. Volume (Int)	Volume (Int)	Abs. Quant.	Rel. Quant.	Band %	Lane %
1		N/A	0,651	2 945 950	3 650 850	N/A	N/A	100,0	79,2

Band Detection	Automatically detected bands with sensitivity: Low
Lane Background	Lane background subtracted with disk size: 24.1
Lane Width	4.71 mm

Lane 4

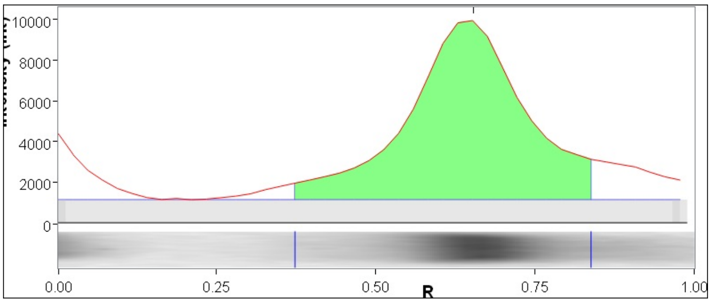

Band No.	Band Label	Mol. Wt. (KDa)	Relative Front	Adj. Volume (Int)	Volume (Int)	Abs. Quant.	Rel. Quant.	Band %	Lane %
1		N/A	0,674	2 941 015	3 779 650	N/A	N/A	100,0	82,0

Band Detection	Automatically detected bands with sensitivity: Low

Lane Background	Lane background subtracted with disk size: 24.1
Lane Width	4.71 mm

Lane 5

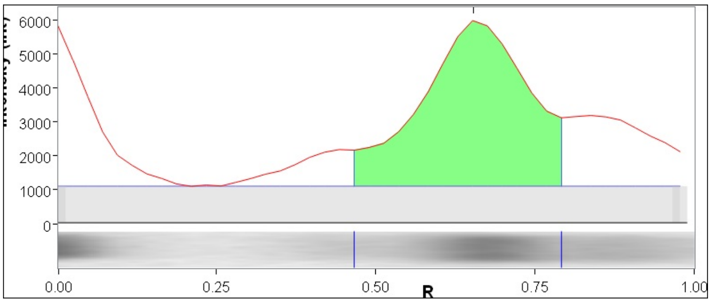

Band No.	Band Label	Mol. Wt. (KDa)	Relative Front	Adj. Volume (Int)	Volume (Int)	Abs. Quant.	Rel. Quant.	Band %	Lane %
1		N/A	0,674	1 581 440	2 170 490	N/A	N/A	100,0	57,2

Band Detection	Automatically detected bands with sensitivity: Low
Lane Background	Lane background subtracted with disk size: 24.1
Lane Width	4.71 mm

Lane 6

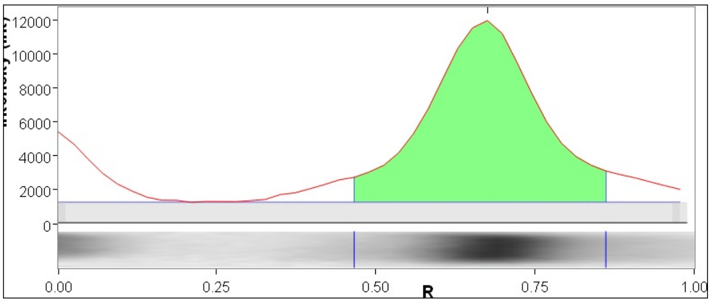

Band No.	Band Label	Mol. Wt. (KDa)	Relative Front	Adj. Volume (Int)	Volume (Int)	Abs. Quant.	Rel. Quant.	Band %	Lane %
1		N/A	0,698	3 355 520	4 122 230	N/A	N/A	100,0	79,4

Band Detection	Automatically detected bands with sensitivity: Low
Lane Background	Lane background subtracted with disk size: 24.1
Lane Width	4.71 mm

Lane 7

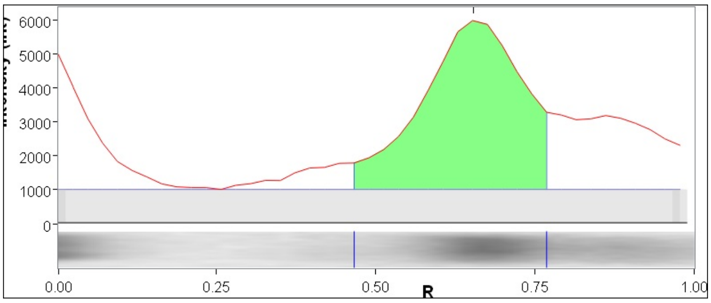

Band No.	Band Label	Mol. Wt. (KDa)	Relative Front	Adj. Volume (Int)	Volume (Int)	Abs. Quant.	Rel. Quant.	Band %	Lane %
1		N/A	0,674	1 626 450	2 186 625	N/A	N/A	100,0	58,1

Band Detection	Automatically detected bands with sensitivity: Low
Lane Background	Lane background subtracted with disk size: 24.1
Lane Width	4.71 mm

Lane 8

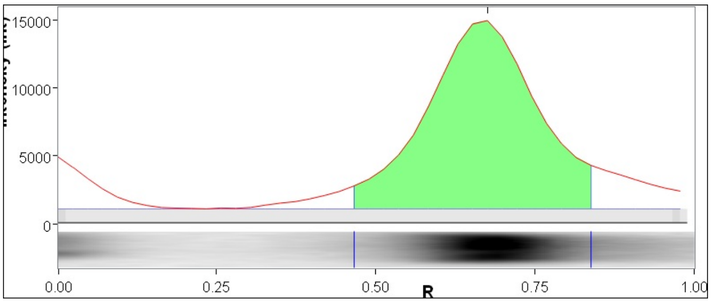

Band No.	Band Label	Mol. Wt. (KDa)	Relative Front	Adj. Volume (Int)	Volume (Int)	Abs. Quant.	Rel. Quant.	Band %	Lane %
1		N/A	0,698	4 972 380	5 666 150	N/A	N/A	100,0	81,6

Band Detection	Automatically detected bands with sensitivity: Low
Lane Background	Lane background subtracted with disk size: 24.1
Lane Width	4.71 mm

Lane 9

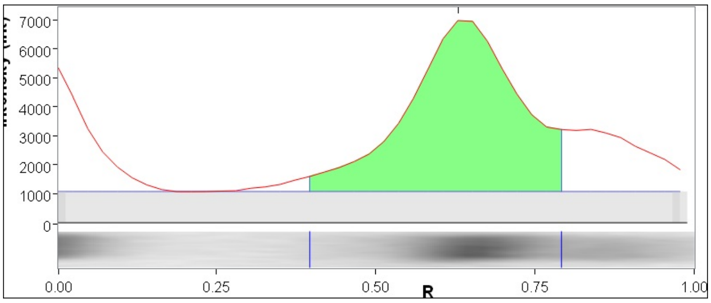

Band No.	Band Label	Mol. Wt. (KDa)	Relative Front	Adj. Volume (Int)	Volume (Int)	Abs. Quant.	Rel. Quant.	Band %	Lane %
1		N/A	0,651	2 099 790	2 846 340	N/A	N/A	100,0	68,5

Band Detection	Automatically detected bands with sensitivity: Low
Lane Background	Lane background subtracted with disk size: 24.1
Lane Width	4.71 mm

Lane 10

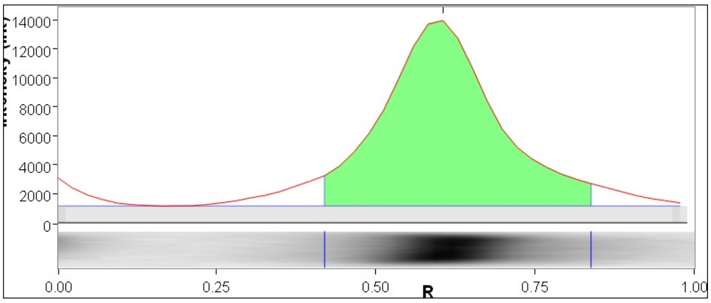

Band No.	Band Label	Mol. Wt. (KDa)	Relative Front	Adj. Volume (Int)	Volume (Int)	Abs. Quant.	Rel. Quant.	Band %	Lane %
1		N/A	0,628	4 459 805	5 316 325	N/A	N/A	100,0	87,8

Band Detection	Automatically detected bands with sensitivity: Low
Lane Background	Lane background subtracted with disk size: 24.1
Lane Width	4.71 mm

Lane 11

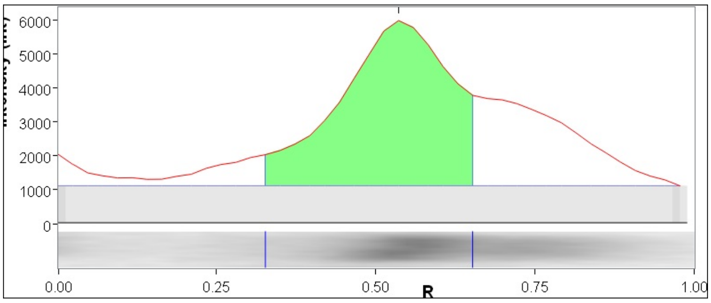

Band No.	Band Label	Mol. Wt. (KDa)	Relative Front	Adj. Volume (Int)	Volume (Int)	Abs. Quant.	Rel. Quant.	Band %	Lane %
1		N/A	0,558	1 601 635	2 181 760	N/A	N/A	100,0	65,5

Band Detection	Automatically detected bands with sensitivity: Low
Lane Background	Lane background subtracted with disk size: 24.1
Lane Width	4.71 mm

Lane 12

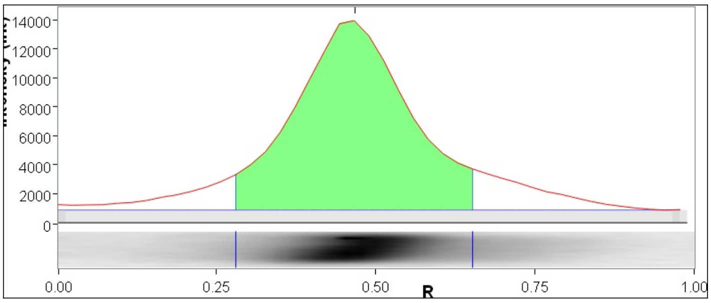

Band No.	Band Label	Mol. Wt. (KDa)	Relative Front	Adj. Volume (Int)	Volume (Int)	Abs. Quant.	Rel. Quant.	Band %	Lane %
1		N/A	0,488	4 605 930	5 177 725	N/A	N/A	100,0	84,1

Band Detection	Automatically detected bands with sensitivity: Low

Lane Background	Lane background subtracted with disk size: 24.1
Lane Width	4.71 mm

Lane 13

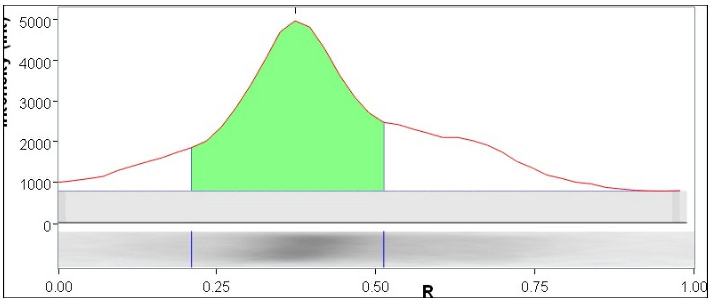

Band No.	Band Label	Mol. Wt. (KDa)	Relative Front	Adj. Volume (Int)	Volume (Int)	Abs. Quant.	Rel. Quant.	Band %	Lane %
1		N/A	0,395	1 419 810	1 839 250	N/A	N/A	100,0	68,0

Band Detection	Automatically detected bands with sensitivity: Low
Lane Background	Lane background subtracted with disk size: 24.1
Lane Width	4.71 mm

Lane 14

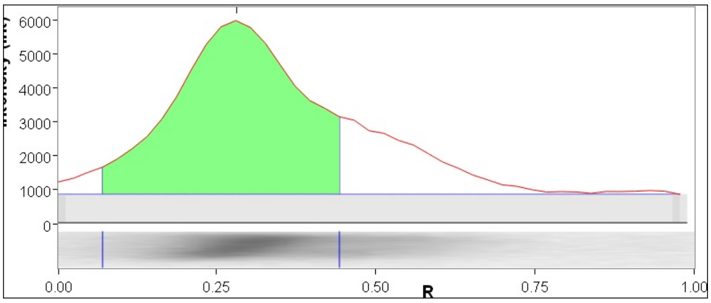

Band No.	Band Label	Mol. Wt. (KDa)	Relative Front	Adj. Volume (Int)	Volume (Int)	Abs. Quant.	Rel. Quant.	Band %	Lane %
1		N/A	0,302	2 187 600	2 771 720	N/A	N/A	100,0	79,0

Band Detection	Automatically detected bands with sensitivity: Low
Lane Background	Lane background subtracted with disk size: 24.1
Lane Width	5.38 mm

Image Report: Fig. 4g, Hmox1 panel, females

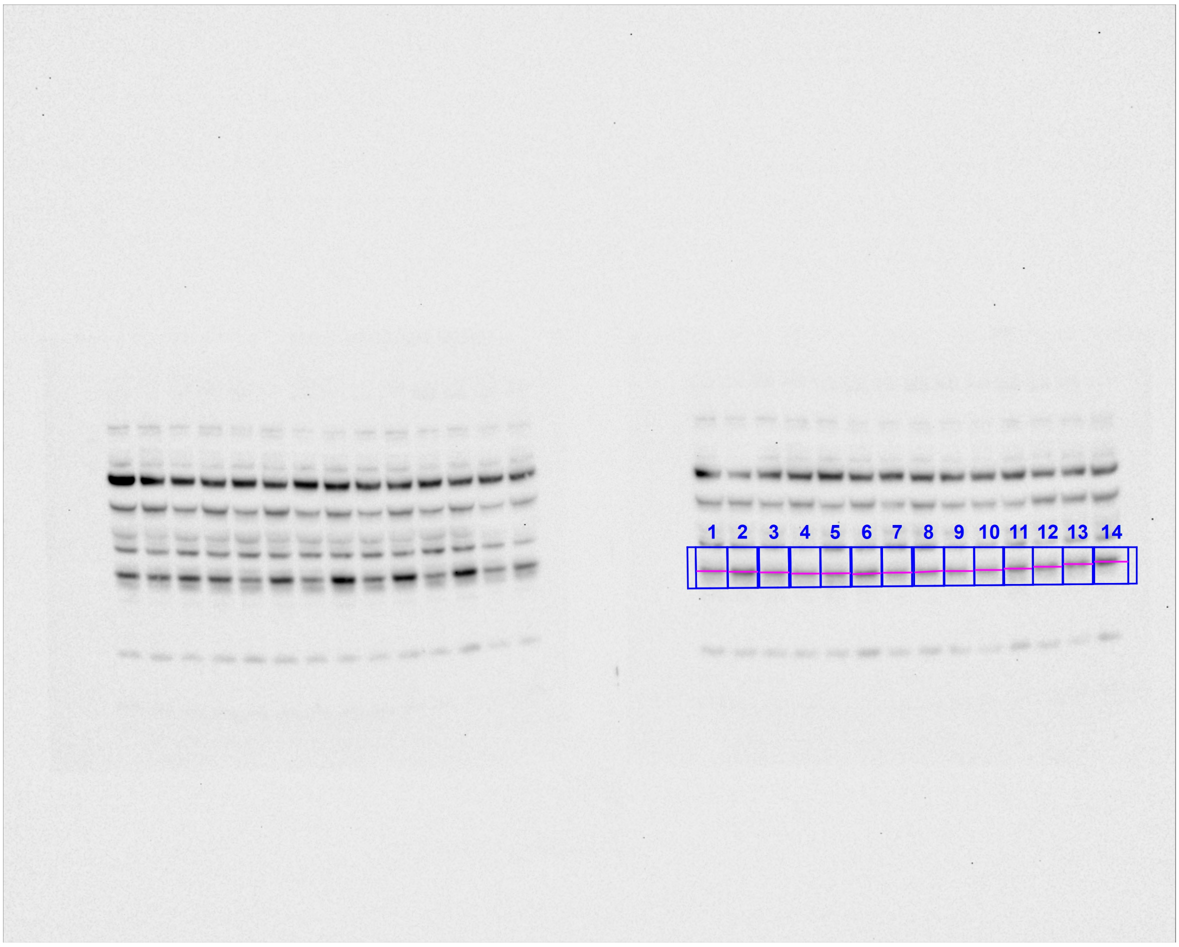

Acquisition Information

Imager	ChemiDoc Touch
Exposure Time (sec)	308.380 (Signal Accumulation)
Serial Number	732BR0263
Software Version	2.3.0.07
Application	Chemiluminescence
Excitation Source	No Illumination
Emission Filter	No Filter
Binning	2x2

Image Information

Acquisition Date	10/6/2022 9:49:57
User Name	m
Image Area (mm)	X: 185.6 Y: 148.6
Pixel Size (µm)	X: 134.6 Y: 134.6

Data Range (Int)	500 - 50404

Analysis Settings

Detection	Lane detection:<br>Manually created lanes (Copied)<br><br>Band detection:<br>Automatically detected bands with sensitivity: Low<br>Manually adjusted bands<br><br>Lane Background Subtraction:<br>Lane background subtracted with disk size: 24.1<br><br>Lane width: Variable

Lane Statistics

Lane No.	Adj. Total Band Vol. (Int)	Total Band Vol. (Int)	Adj. Total Lane Vol. (Int)	Total Lane Vol. (Int)	Bkgd. Vol. (Int)	Norm. Factor
1	1 728 344	2 520 366	2 091 610	3 819 658	1 728 048	N/A
2	2 794 050	3 653 300	3 133 270	4 783 030	1 649 760	N/A
3	1 567 860	2 180 990	2 141 545	3 690 505	1 548 960	N/A
4	1 485 575	2 052 155	1 931 370	3 332 910	1 401 540	N/A
5	1 473 605	2 043 685	2 387 140	4 061 750	1 674 610	N/A
6	2 263 380	2 867 865	2 858 170	4 321 660	1 463 490	N/A
7	1 341 795	2 004 695	1 816 535	3 341 205	1 524 670	N/A
8	1 361 010	1 869 490	1 959 615	3 421 495	1 461 880	N/A
9	1 205 610	1 681 610	1 600 165	2 938 915	1 338 750	N/A
10	1 210 510	1 799 700	1 450 575	2 846 025	1 395 450	N/A
11	1 546 265	2 077 705	1 998 045	3 459 505	1 461 460	N/A
12	1 761 760	2 369 605	1 990 345	3 263 925	1 273 580	N/A
13	1 842 855	2 437 855	2 153 480	3 462 480	1 309 000	N/A
14	2 655 560	3 393 880	2 928 920	4 372 000	1 443 080	N/A

Lane And Band Analysis

Lane 1

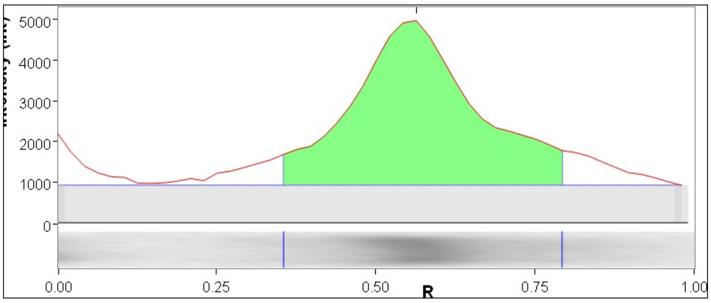

Band No.	Band Label	Mol. Wt. (KDa)	Relative Front	Adj. Volume (Int)	Volume (Int)	Abs. Quant.	Rel. Quant.	Band %	Lane %
1		N/A	0,583	1 728 344	2 520 366	N/A	N/A	100,0	82,6

Band Detection	Automatically detected bands with sensitivity: Low
Lane Background	Lane background subtracted with disk size: 24.1
Lane Width	4.98 mm

Lane 2

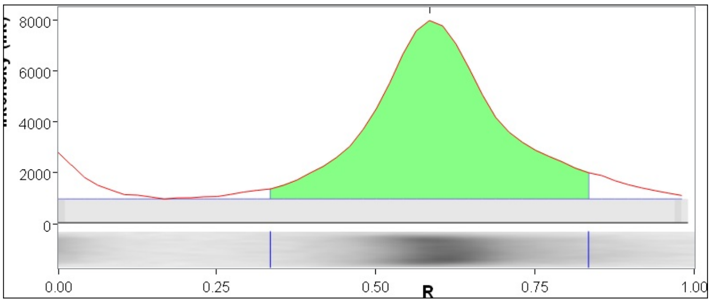

Band No.	Band Label	Mol. Wt. (KDa)	Relative Front	Adj. Volume (Int)	Volume (Int)	Abs. Quant.	Rel. Quant.	Band %	Lane %
1		N/A	0,604	2 794 050	3 653 300	N/A	N/A	100,0	89,2

Band Detection	Automatically detected bands with sensitivity: Low
Lane Background	Lane background subtracted with disk size: 24.1
Lane Width	4.71 mm

Lane 3

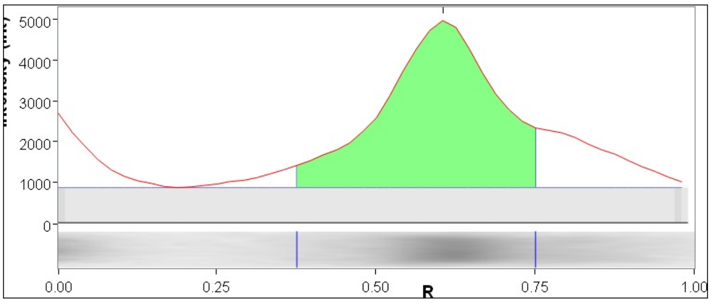

Band No.	Band Label	Mol. Wt. (KDa)	Relative Front	Adj. Volume (Int)	Volume (Int)	Abs. Quant.	Rel. Quant.	Band %	Lane %
1		N/A	0,625	1 567 860	2 180 990	N/A	N/A	100,0	73,2

Band Detection	Automatically detected bands with sensitivity: Low
Lane Background	Lane background subtracted with disk size: 24.1
Lane Width	4.71 mm

Lane 4

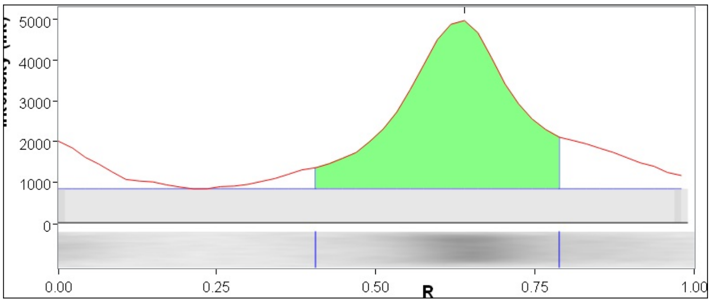

Band No.	Band Label	Mol. Wt. (KDa)	Relative Front	Adj. Volume (Int)	Volume (Int)	Abs. Quant.	Rel. Quant.	Band %	Lane %
1		N/A	0,660	1 485 575	2 052 155	N/A	N/A	100,0	76,9

Band Detection	Automatically detected bands with sensitivity: Low

Lane Background	Lane background subtracted with disk size: 24.1
Lane Width	4.71 mm

Lane 5

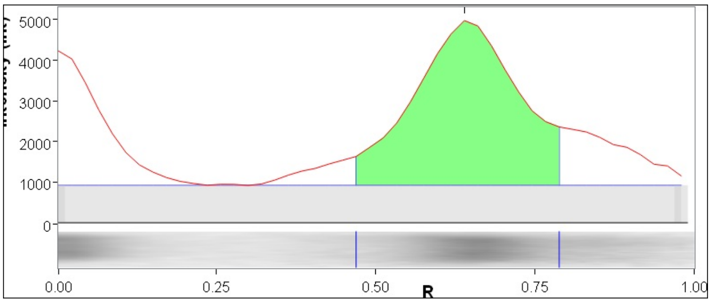

Band No.	Band Label	Mol. Wt. (KDa)	Relative Front	Adj. Volume (Int)	Volume (Int)	Abs. Quant.	Rel. Quant.	Band %	Lane %
1		N/A	0,660	1 473 605	2 043 685	N/A	N/A	100,0	61,7

Band Detection	Automatically detected bands with sensitivity: Low
Lane Background	Lane background subtracted with disk size: 24.1
Lane Width	4.71 mm

Lane 6

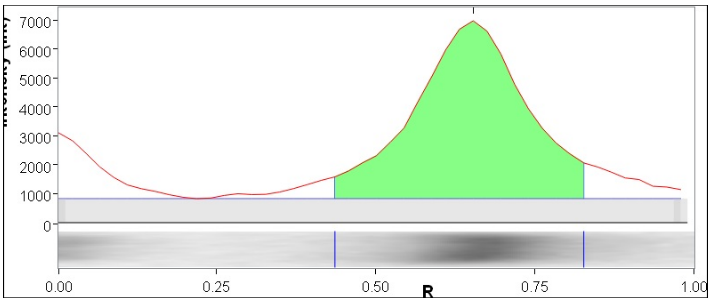

Band No.	Band Label	Mol. Wt. (KDa)	Relative Front	Adj. Volume (Int)	Volume (Int)	Abs. Quant.	Rel. Quant.	Band %	Lane %
1		N/A	0,674	2 263 380	2 867 865	N/A	N/A	100,0	79,2

Band Detection	Automatically detected bands with sensitivity: Low
Lane Background	Lane background subtracted with disk size: 24.1
Lane Width	4.71 mm

Lane 7

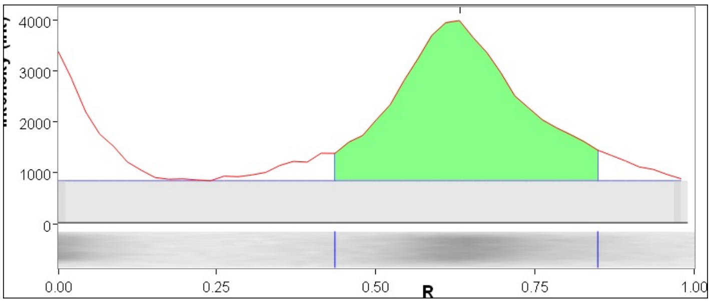

Band No.	Band Label	Mol. Wt. (KDa)	Relative Front	Adj. Volume (Int)	Volume (Int)	Abs. Quant.	Rel. Quant.	Band %	Lane %
1		N/A	0,652	1 341 795	2 004 695	N/A	N/A	100,0	73,9

Band Detection	Automatically detected bands with sensitivity: Low
Lane Background	Lane background subtracted with disk size: 24.1
Lane Width	4.71 mm

Lane 8

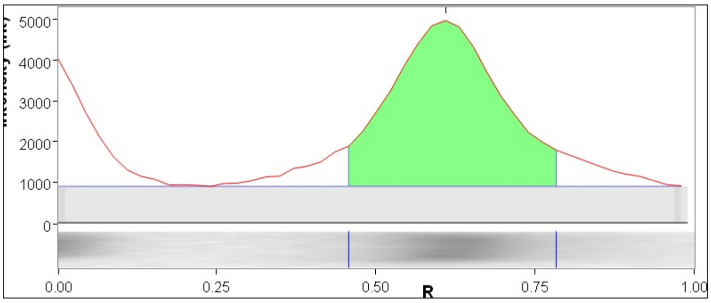

Band No.	Band Label	Mol. Wt. (KDa)	Relative Front	Adj. Volume (Int)	Volume (Int)	Abs. Quant.	Rel. Quant.	Band %	Lane %
1		N/A	0,630	1 361 010	1 869 490	N/A	N/A	100,0	69,5

Band Detection	Automatically detected bands with sensitivity: Low
Lane Background	Lane background subtracted with disk size: 24.1
Lane Width	4.71 mm

Lane 9

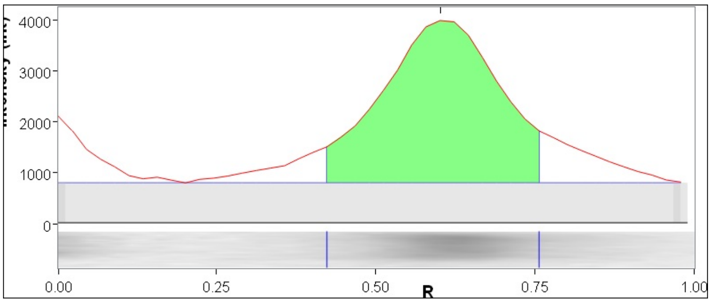

Band No.	Band Label	Mol. Wt. (KDa)	Relative Front	Adj. Volume (Int)	Volume (Int)	Abs. Quant.	Rel. Quant.	Band %	Lane %
1		N/A	0,622	1 205 610	1 681 610	N/A	N/A	100,0	75,3

Band Detection	Automatically detected bands with sensitivity: Low
Lane Background	Lane background subtracted with disk size: 24.1
Lane Width	4.71 mm

Lane 10

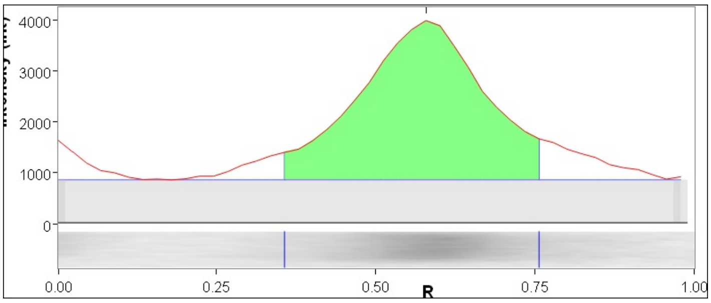

Band No.	Band Label	Mol. Wt. (KDa)	Relative Front	Adj. Volume (Int)	Volume (Int)	Abs. Quant.	Rel. Quant.	Band %	Lane %
1		N/A	0,600	1 210 510	1 799 700	N/A	N/A	100,0	83,5

Band Detection	Automatically detected bands with sensitivity: Low
Lane Background	Lane background subtracted with disk size: 24.1
Lane Width	4.71 mm

Lane 11

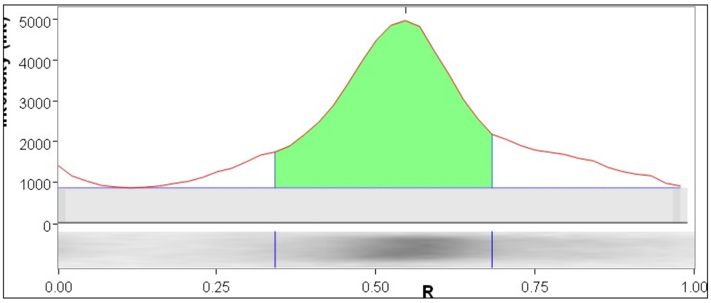

Band No.	Band Label	Mol. Wt. (KDa)	Relative Front	Adj. Volume (Int)	Volume (Int)	Abs. Quant.	Rel. Quant.	Band %	Lane %
1		N/A	0,568	1 546 265	2 077 705	N/A	N/A	100,0	77,4

Band Detection	Automatically detected bands with sensitivity: Low
Lane Background	Lane background subtracted with disk size: 24.1
Lane Width	4.71 mm

Lane 12

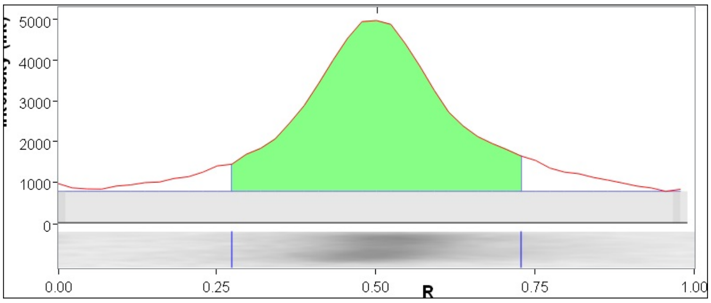

Band No.	Band Label	Mol. Wt. (KDa)	Relative Front	Adj. Volume (Int)	Volume (Int)	Abs. Quant.	Rel. Quant.	Band %	Lane %
1		N/A	0,523	1 761 760	2 369 605	N/A	N/A	100,0	88,5

Band Detection	Automatically detected bands with sensitivity: Low

Lane Background	Lane background subtracted with disk size: 24.1
Lane Width	4.71 mm

Lane 13

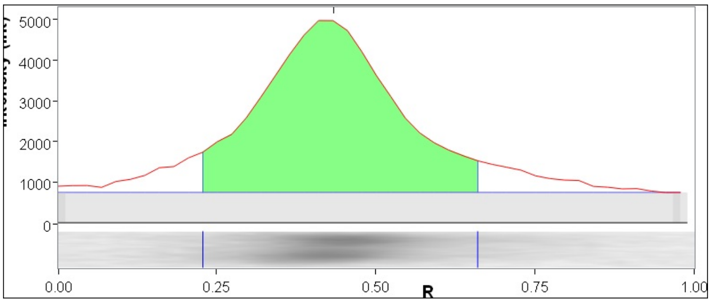

Band No.	Band Label	Mol. Wt. (KDa)	Relative Front	Adj. Volume (Int)	Volume (Int)	Abs. Quant.	Rel. Quant.	Band %	Lane %
1		N/A	0,455	1 842 855	2 437 855	N/A	N/A	100,0	85,6

Band Detection	Automatically detected bands with sensitivity: Low
Lane Background	Lane background subtracted with disk size: 24.1
Lane Width	4.71 mm

Lane 14

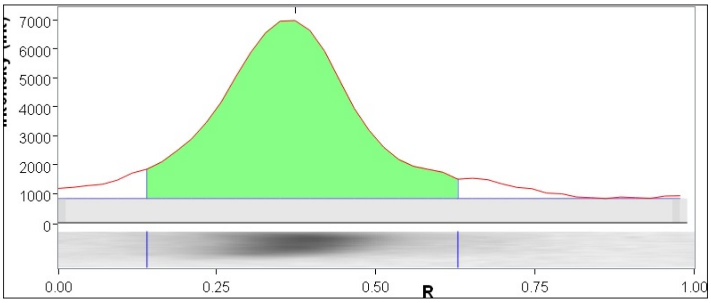

Band No.	Band Label	Mol. Wt. (KDa)	Relative Front	Adj. Volume (Int)	Volume (Int)	Abs. Quant.	Rel. Quant.	Band %	Lane %
1		N/A	0,395	2 655 560	3 393 880	N/A	N/A	100,0	90,7

Band Detection	Automatically detected bands with sensitivity: Low
Lane Background	Lane background subtracted with disk size: 24.1
Lane Width	5.38 mm

Image Report: Fig. 4g, Nqo1 panel, males

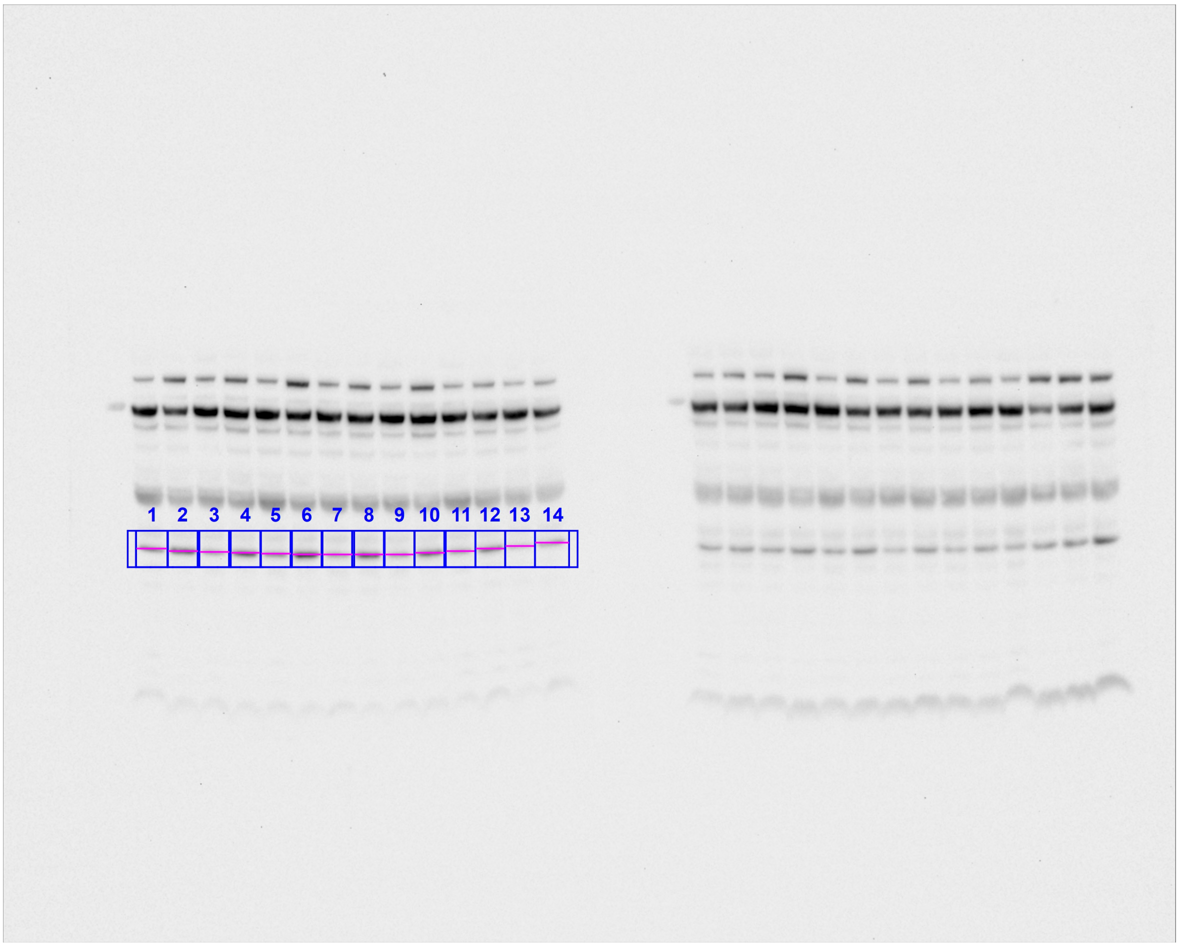

Acquisition Information

Imager	ChemiDoc Touch
Exposure Time (sec)	411.152 (Signal Accumulation)
Serial Number	732BR0263
Software Version	2.3.0.07
Application	Chemiluminescence
Excitation Source	No Illumination
Emission Filter	No Filter
Binning	2x2

Image Information

Acquisition Date	10/5/2022 11:17:24
User Name	m
Image Area (mm)	X: 185.6 Y: 148.6
Pixel Size (µm)	X: 134.6 Y: 134.6

Data Range (Int)	500 - 32012

Analysis Settings

Detection	Lane detection:<br>Manually created lanes<br><br>Band detection:<br>Automatically detected bands with sensitivity: Low<br>Manually adjusted bands<br><br>Lane Background Subtraction:<br>Lane background subtracted with disk size: 24.1<br><br>Lane width: Variable

Lane Statistics

Lane No.	Adj. Total Band Vol. (Int)	Total Band Vol. (Int)	Adj. Total Lane Vol. (Int)	Total Lane Vol. (Int)	Bkgd. Vol. (Int)	Norm. Factor
1	1 125 984	1 622 820	1 337 624	2 524 510	1 186 886	N/A
2	1 851 745	2 371 845	2 020 655	3 138 870	1 118 215	N/A
3	692 195	1 116 115	893 725	2 033 010	1 139 285	N/A
4	1 816 255	2 339 855	2 037 840	3 163 580	1 125 740	N/A
5	969 010	1 435 490	1 242 115	2 422 035	1 179 920	N/A
6	2 501 625	3 118 290	2 735 320	3 998 015	1 262 695	N/A
7	745 045	1 222 235	1 014 860	2 221 870	1 207 010	N/A
8	1 800 330	2 412 130	1 998 745	3 314 115	1 315 370	N/A
9	677 425	1 109 500	882 770	2 121 385	1 238 615	N/A
10	1 652 840	2 272 445	1 813 875	3 082 590	1 268 715	N/A
11	767 480	1 230 390	977 970	2 148 860	1 170 890	N/A
12	1 286 040	1 843 310	1 400 945	2 662 135	1 261 190	N/A
13	464 590	871 710	569 450	1 663 585	1 094 135	N/A
14	889 640	1 398 680	969 920	2 185 960	1 216 040	N/A

Lane And Band Analysis

Lane 1

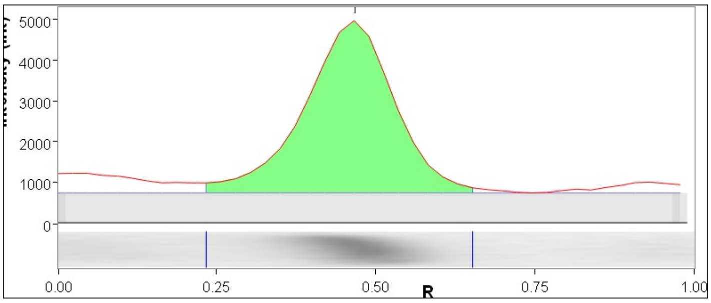

Band No.	Band Label	Mol. Wt. (KDa)	Relative Front	Adj. Volume (Int)	Volume (Int)	Abs. Quant.	Rel. Quant.	Band %	Lane %
1		N/A	0,488	1 125 984	1 622 820	N/A	N/A	100,0	84,2

Band Detection	Automatically detected bands with sensitivity: Low
Lane Background	Lane background subtracted with disk size: 24.1
Lane Width	4.98 mm

Lane 2

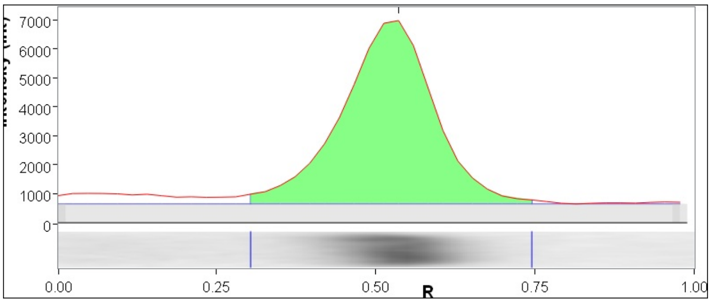

Band No.	Band Label	Mol. Wt. (KDa)	Relative Front	Adj. Volume (Int)	Volume (Int)	Abs. Quant.	Rel. Quant.	Band %	Lane %
1		N/A	0,558	1 851 745	2 371 845	N/A	N/A	100,0	91,6

Band Detection	Automatically detected bands with sensitivity: Low
Lane Background	Lane background subtracted with disk size: 24.1
Lane Width	4.71 mm

Lane 3

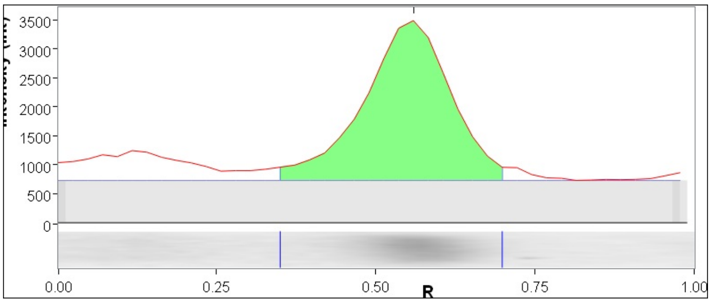

Band No.	Band Label	Mol. Wt. (KDa)	Relative Front	Adj. Volume (Int)	Volume (Int)	Abs. Quant.	Rel. Quant.	Band %	Lane %
1		N/A	0,581	692 195	1 116 115	N/A	N/A	100,0	77,5

Band Detection	Automatically detected bands with sensitivity: Low
Lane Background	Lane background subtracted with disk size: 24.1
Lane Width	4.71 mm

Lane 4

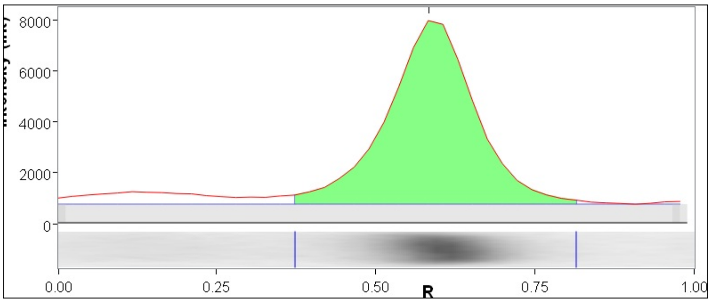

Band No.	Band Label	Mol. Wt. (KDa)	Relative Front	Adj. Volume (Int)	Volume (Int)	Abs. Quant.	Rel. Quant.	Band %	Lane %
1		N/A	0,605	1 816 255	2 339 855	N/A	N/A	100,0	89,1

Band Detection	Automatically detected bands with sensitivity: Low

Lane Background	Lane background subtracted with disk size: 24.1
Lane Width	4.71 mm

Lane 5

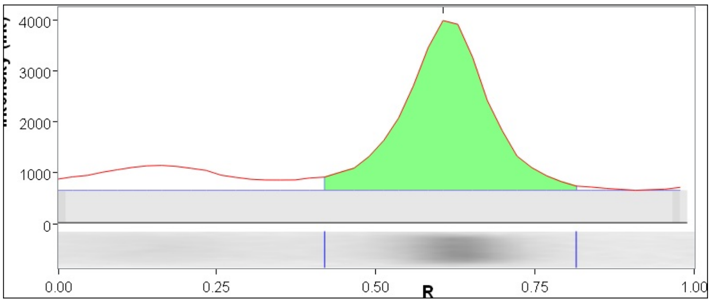

Band No.	Band Label	Mol. Wt. (KDa)	Relative Front	Adj. Volume (Int)	Volume (Int)	Abs. Quant.	Rel. Quant.	Band %	Lane %
1		N/A	0,628	969 010	1 435 490	N/A	N/A	100,0	78,0

Band Detection	Automatically detected bands with sensitivity: Low
Lane Background	Lane background subtracted with disk size: 24.1
Lane Width	4.71 mm

Lane 6

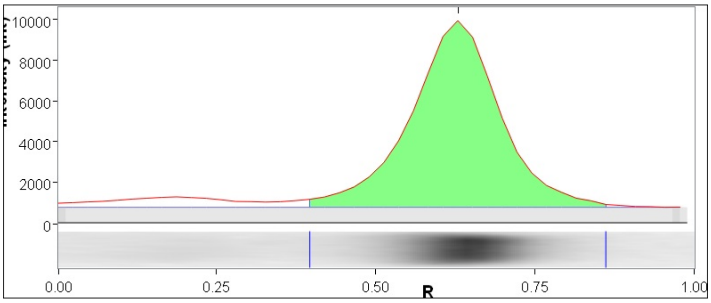

Band No.	Band Label	Mol. Wt. (KDa)	Relative Front	Adj. Volume (Int)	Volume (Int)	Abs. Quant.	Rel. Quant.	Band %	Lane %
1		N/A	0,651	2 501 625	3 118 290	N/A	N/A	100,0	91,5

Band Detection	Automatically detected bands with sensitivity: Low
Lane Background	Lane background subtracted with disk size: 24.1
Lane Width	4.71 mm

Lane 7

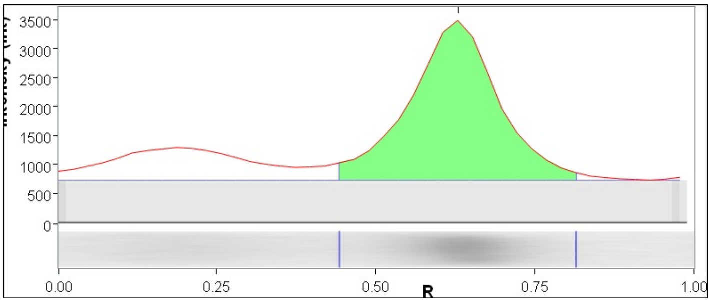

Band No.	Band Label	Mol. Wt. (KDa)	Relative Front	Adj. Volume (Int)	Volume (Int)	Abs. Quant.	Rel. Quant.	Band %	Lane %
1		N/A	0,651	745 045	1 222 235	N/A	N/A	100,0	73,4

Band Detection	Automatically detected bands with sensitivity: Low
Lane Background	Lane background subtracted with disk size: 24.1
Lane Width	4.71 mm

Lane 8

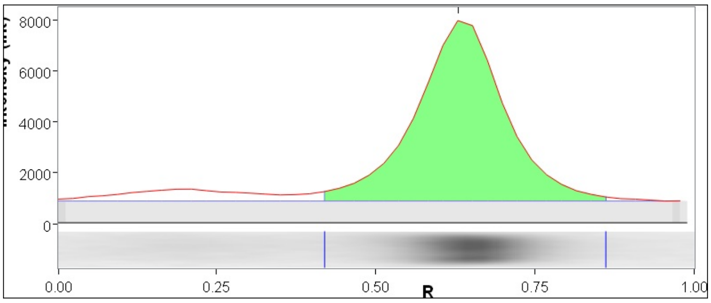

Band No.	Band Label	Mol. Wt. (KDa)	Relative Front	Adj. Volume (Int)	Volume (Int)	Abs. Quant.	Rel. Quant.	Band %	Lane %
1		N/A	0,651	1 800 330	2 412 130	N/A	N/A	100,0	90,1

Band Detection	Automatically detected bands with sensitivity: Low
Lane Background	Lane background subtracted with disk size: 24.1
Lane Width	4.71 mm

Lane 9

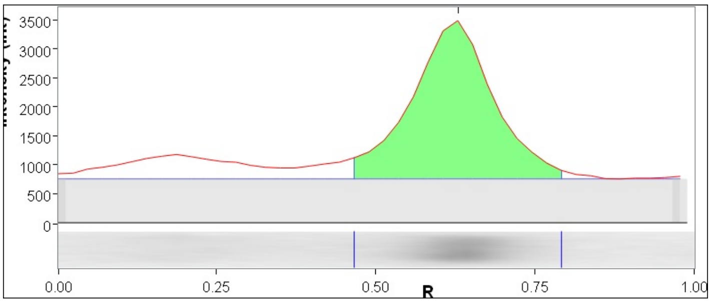

Band No.	Band Label	Mol. Wt. (KDa)	Relative Front	Adj. Volume (Int)	Volume (Int)	Abs. Quant.	Rel. Quant.	Band %	Lane %
1		N/A	0,651	677 425	1 109 500	N/A	N/A	100,0	76,7

Band Detection	Automatically detected bands with sensitivity: Low
Lane Background	Lane background subtracted with disk size: 24.1
Lane Width	4.71 mm

Lane 10

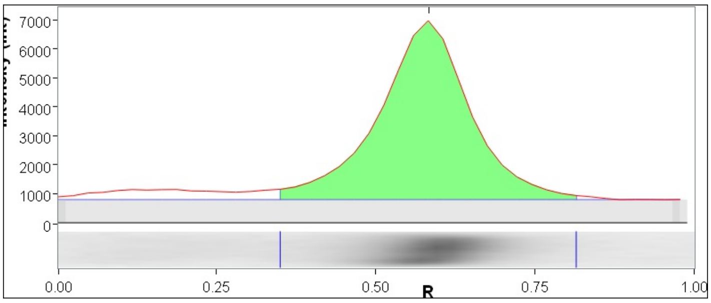

Band No.	Band Label	Mol. Wt. (KDa)	Relative Front	Adj. Volume (Int)	Volume (Int)	Abs. Quant.	Rel. Quant.	Band %	Lane %
1		N/A	0,605	1 652 840	2 272 445	N/A	N/A	100,0	91,1

Band Detection	Automatically detected bands with sensitivity: Low
Lane Background	Lane background subtracted with disk size: 24.1
Lane Width	4.71 mm

Lane 11

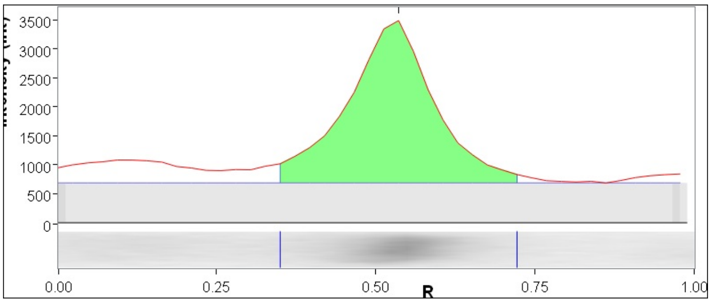

Band No.	Band Label	Mol. Wt. (KDa)	Relative Front	Adj. Volume (Int)	Volume (Int)	Abs. Quant.	Rel. Quant.	Band %	Lane %
1		N/A	0,558	767 480	1 230 390	N/A	N/A	100,0	78,5

Band Detection	Automatically detected bands with sensitivity: Low
Lane Background	Lane background subtracted with disk size: 24.1
Lane Width	4.71 mm

Lane 12

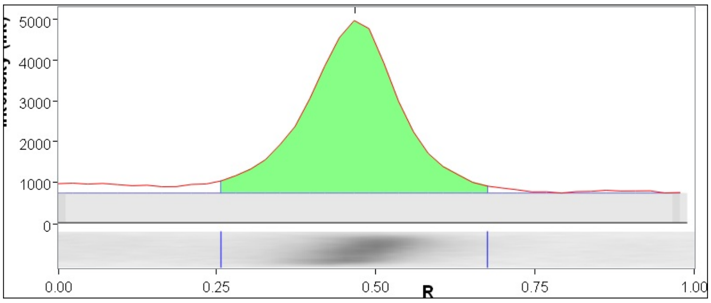

Band No.	Band Label	Mol. Wt. (KDa)	Relative Front	Adj. Volume (Int)	Volume (Int)	Abs. Quant.	Rel. Quant.	Band %	Lane %
1		N/A	0,488	1 286 040	1 843 310	N/A	N/A	100,0	91,8

Band Detection	Automatically detected bands with sensitivity: Low

Lane Background	Lane background subtracted with disk size: 24.1
Lane Width	4.71 mm

Lane 13

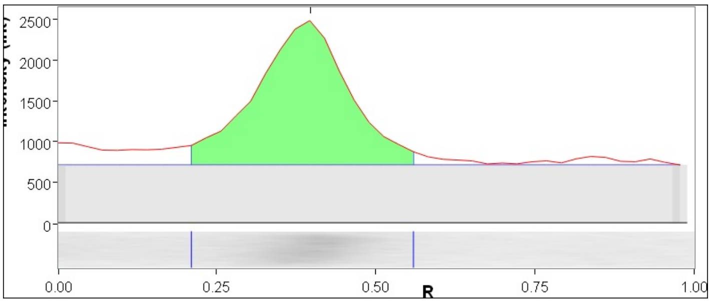

Band No.	Band Label	Mol. Wt. (KDa)	Relative Front	Adj. Volume (Int)	Volume (Int)	Abs. Quant.	Rel. Quant.	Band %	Lane %
1		N/A	0,419	464 590	871 710	N/A	N/A	100,0	81,6

Band Detection	Automatically detected bands with sensitivity: Low
Lane Background	Lane background subtracted with disk size: 24.1
Lane Width	4.71 mm

Lane 14

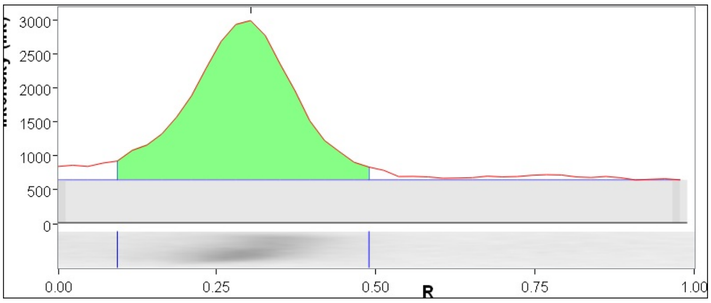

Band No.	Band Label	Mol. Wt. (KDa)	Relative Front	Adj. Volume (Int)	Volume (Int)	Abs. Quant.	Rel. Quant.	Band %	Lane %
1		N/A	0,326	889 640	1 398 680	N/A	N/A	100,0	91,7

Band Detection	Automatically detected bands with sensitivity: Low
Lane Background	Lane background subtracted with disk size: 24.1
Lane Width	5.38 mm

Image Report: Fig. 4g, Nqo1 panel, females

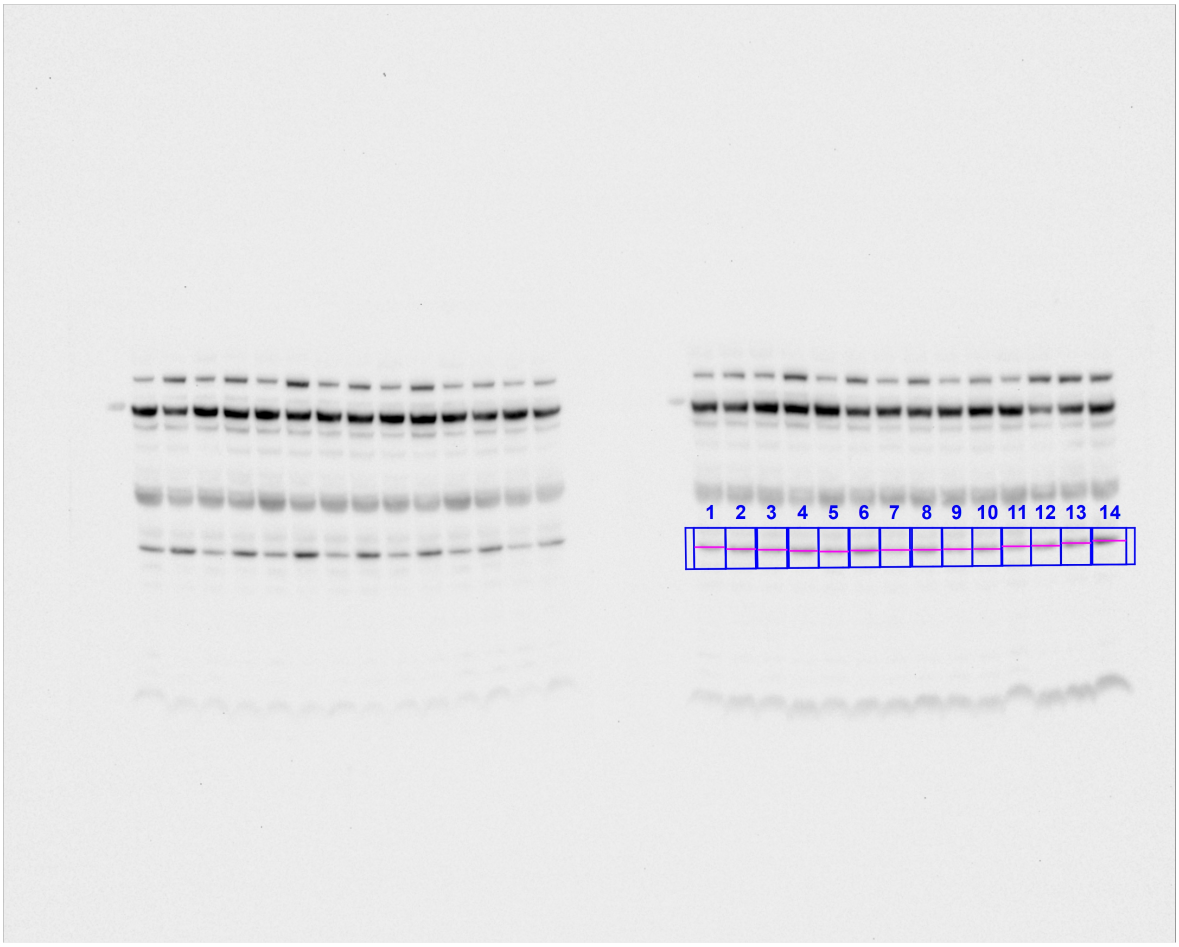

Acquisition Information

Imager	ChemiDoc Touch
Exposure Time (sec)	411.152 (Signal Accumulation)
Serial Number	732BR0263
Software Version	2.3.0.07
Application	Chemiluminescence
Excitation Source	No Illumination
Emission Filter	No Filter
Binning	2x2

Image Information

Acquisition Date	10/5/2022 11:17:24
User Name	m
Image Area (mm)	X: 185.6 Y: 148.6
Pixel Size (μm)	X: 134.6 Y: 134.6

Data Range (Int)	500 - 32012

Analysis Settings

Detection	Lane detection:<br>Manually created lanes<br><br>Band detection:<br>Automatically detected bands with sensitivity: Low<br>Manually adjusted bands<br><br>Lane Background Subtraction:<br>Lane background subtracted with disk size: 24.1<br><br>Lane width: Variable

Lane Statistics

Lane No.	Adj. Total Band Vol. (Int)	Total Band Vol. (Int)	Adj. Total Lane Vol. (Int)	Total Lane Vol. (Int)	Bkgd. Vol. (Int)	Norm. Factor
1	708 550	1 303 510	890 923	2 348 575	1 457 652	N/A
2	993 195	1 605 345	1 191 155	2 526 755	1 335 600	N/A
3	820 750	1 410 955	1 059 485	2 408 525	1 349 040	N/A
4	1 231 895	1 869 455	1 419 670	2 722 510	1 302 840	N/A
5	763 490	1 324 190	1 032 255	2 349 900	1 317 645	N/A
6	1 260 000	1 902 950	1 455 020	2 828 595	1 373 575	N/A
7	496 755	984 375	715 890	1 962 030	1 246 140	N/A
8	957 390	1 551 270	1 141 350	2 442 230	1 300 880	N/A
9	538 055	970 935	839 405	2 056 880	1 217 475	N/A
10	866 460	1 408 960	1 068 830	2 289 455	1 220 625	N/A
11	623 770	1 127 770	911 680	2 171 680	1 260 000	N/A
12	972 475	1 492 120	1 097 670	2 186 450	1 088 780	N/A
13	1 207 920	1 756 230	1 336 510	2 485 350	1 148 840	N/A
14	1 921 880	2 661 880	1 958 520	3 231 320	1 272 800	N/A

Lane And Band Analysis

Lane 1

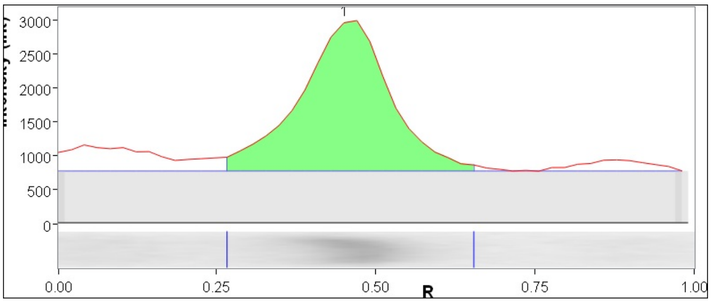

Band No.	Band Label	Mol. Wt. (KDa)	Relative Front	Adj. Volume (Int)	Volume (Int)	Abs. Quant.	Rel. Quant.	Band %	Lane %
1		N/A	0,469	708 550	1 303 510	N/A	N/A	100,0	79,5

Band Detection	Automatically detected bands with sensitivity: Low
Lane Background	Lane background subtracted with disk size: 24.1
Lane Width	4.98 mm

Lane 2

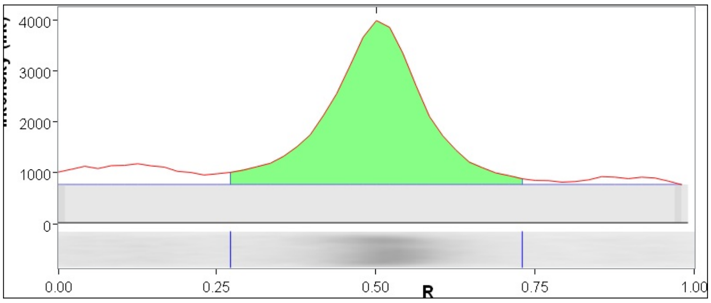

Band No.	Band Label	Mol. Wt. (KDa)	Relative Front	Adj. Volume (Int)	Volume (Int)	Abs. Quant.	Rel. Quant.	Band %	Lane %
1		N/A	0,521	993 195	1 605 345	N/A	N/A	100,0	83,4

Band Detection	Automatically detected bands with sensitivity: Low
Lane Background	Lane background subtracted with disk size: 24.1
Lane Width	4.71 mm

Lane 3

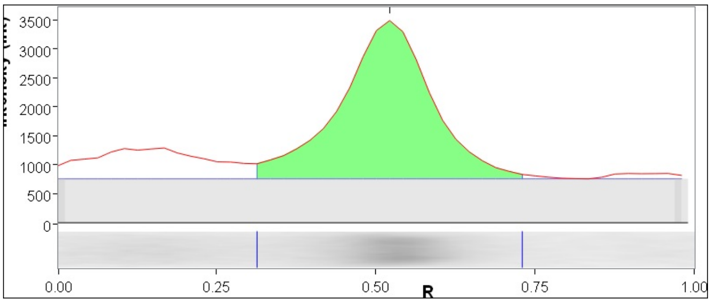

Band No.	Band Label	Mol. Wt. (KDa)	Relative Front	Adj. Volume (Int)	Volume (Int)	Abs. Quant.	Rel. Quant.	Band %	Lane %
1		N/A	0,542	820 750	1 410 955	N/A	N/A	100,0	77,5

Band Detection	Automatically detected bands with sensitivity: Low
Lane Background	Lane background subtracted with disk size: 24.1
Lane Width	4.71 mm

Lane 4

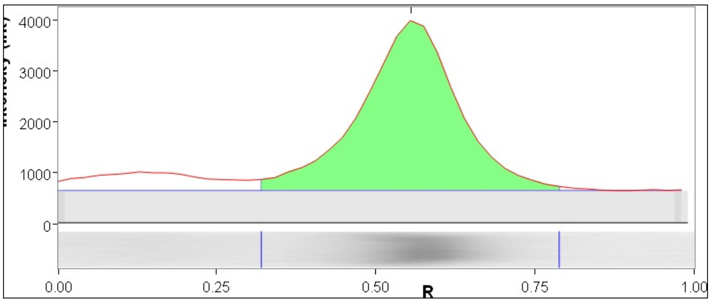

Band No.	Band Label	Mol. Wt. (KDa)	Relative Front	Adj. Volume (Int)	Volume (Int)	Abs. Quant.	Rel. Quant.	Band %	Lane %
1		N/A	0,574	1 231 895	1 869 455	N/A	N/A	100,0	86,8

Band Detection	Automatically detected bands with sensitivity: Low

Lane Background	Lane background subtracted with disk size: 24.1
Lane Width	4.71 mm

Lane 5

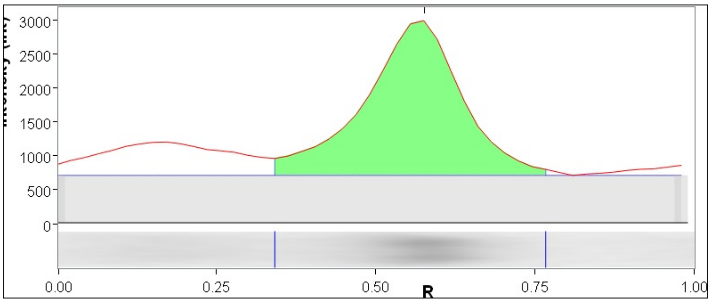

Band No.	Band Label	Mol. Wt. (KDa)	Relative Front	Adj. Volume (Int)	Volume (Int)	Abs. Quant.	Rel. Quant.	Band %	Lane %
1		N/A	0,596	763 490	1 324 190	N/A	N/A	100,0	74,0

Band Detection	Automatically detected bands with sensitivity: Low
Lane Background	Lane background subtracted with disk size: 24.1
Lane Width	4.71 mm

Lane 6

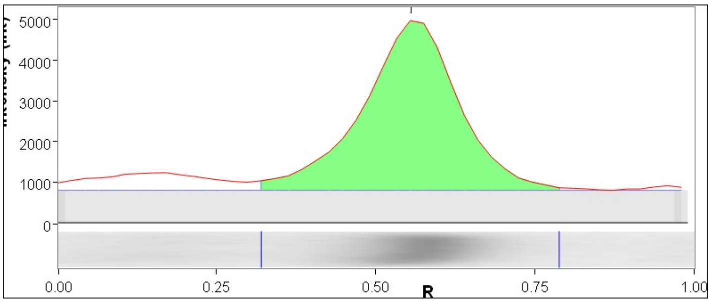

Band No.	Band Label	Mol. Wt. (KDa)	Relative Front	Adj. Volume (Int)	Volume (Int)	Abs. Quant.	Rel. Quant.	Band %	Lane %
1		N/A	0,574	1 260 000	1 902 950	N/A	N/A	100,0	86,6

Band Detection	Automatically detected bands with sensitivity: Low
Lane Background	Lane background subtracted with disk size: 24.1
Lane Width	4.71 mm

Lane 7

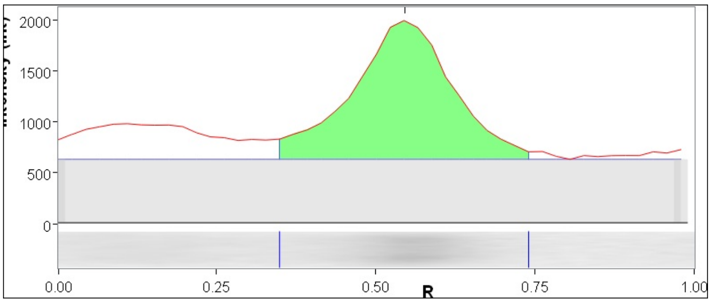

Band No.	Band Label	Mol. Wt. (KDa)	Relative Front	Adj. Volume (Int)	Volume (Int)	Abs. Quant.	Rel. Quant.	Band %	Lane %
1		N/A	0,565	496 755	984 375	N/A	N/A	100,0	69,4

Band Detection	Automatically detected bands with sensitivity: Low
Lane Background	Lane background subtracted with disk size: 24.1
Lane Width	4.71 mm

Lane 8

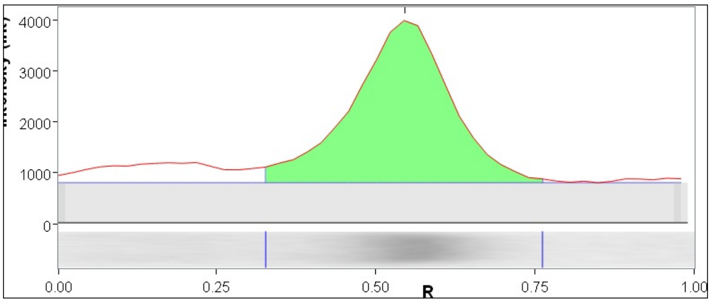

Band No.	Band Label	Mol. Wt. (KDa)	Relative Front	Adj. Volume (Int)	Volume (Int)	Abs. Quant.	Rel. Quant.	Band %	Lane %
1		N/A	0,565	957 390	1 551 270	N/A	N/A	100,0	83,9

Band Detection	Automatically detected bands with sensitivity: Low
Lane Background	Lane background subtracted with disk size: 24.1
Lane Width	4.71 mm

Lane 9

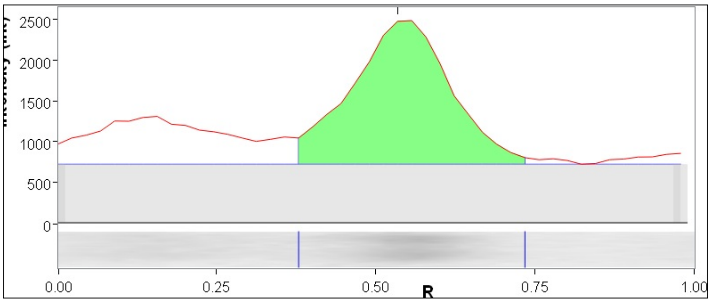

Band No.	Band Label	Mol. Wt. (KDa)	Relative Front	Adj. Volume (Int)	Volume (Int)	Abs. Quant.	Rel. Quant.	Band %	Lane %
1		N/A	0,556	538 055	970 935	N/A	N/A	100,0	64,1

Band Detection	Automatically detected bands with sensitivity: Low
Lane Background	Lane background subtracted with disk size: 24.1
Lane Width	4.71 mm

Lane 10

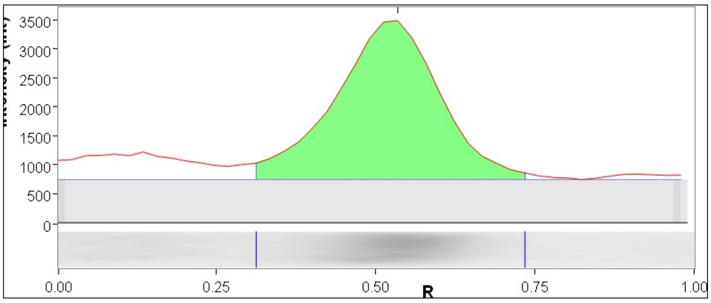

Band No.	Band Label	Mol. Wt. (KDa)	Relative Front	Adj. Volume (Int)	Volume (Int)	Abs. Quant.	Rel. Quant.	Band %	Lane %
1		N/A	0,556	866 460	1 408 960	N/A	N/A	100,0	81,1

Band Detection	Automatically detected bands with sensitivity: Low
Lane Background	Lane background subtracted with disk size: 24.1
Lane Width	4.71 mm

Lane 11

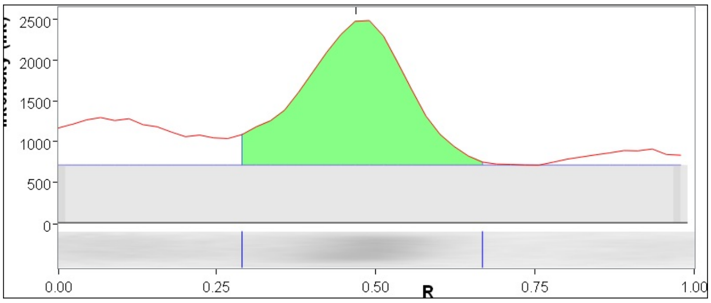

Band No.	Band Label	Mol. Wt. (KDa)	Relative Front	Adj. Volume (Int)	Volume (Int)	Abs. Quant.	Rel. Quant.	Band %	Lane %
1		N/A	0,489	623 770	1 127 770	N/A	N/A	100,0	68,4

Band Detection	Automatically detected bands with sensitivity: Low
Lane Background	Lane background subtracted with disk size: 24.1
Lane Width	4.71 mm

Lane 12

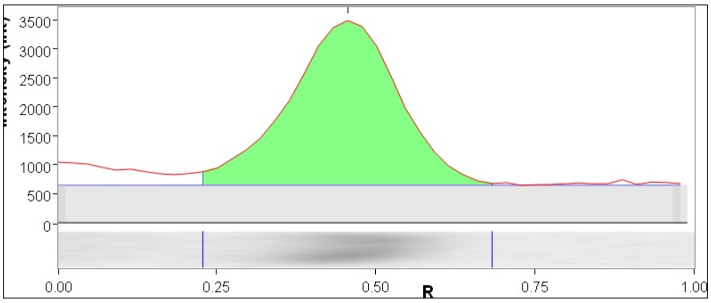

Band No.	Band Label	Mol. Wt. (KDa)	Relative Front	Adj. Volume (Int)	Volume (Int)	Abs. Quant.	Rel. Quant.	Band %	Lane %
1		N/A	0,477	972 475	1 492 120	N/A	N/A	100,0	88,6

Band Detection	Automatically detected bands with sensitivity: Low

Lane Background	Lane background subtracted with disk size: 24.1
Lane Width	4.71 mm

Lane 13

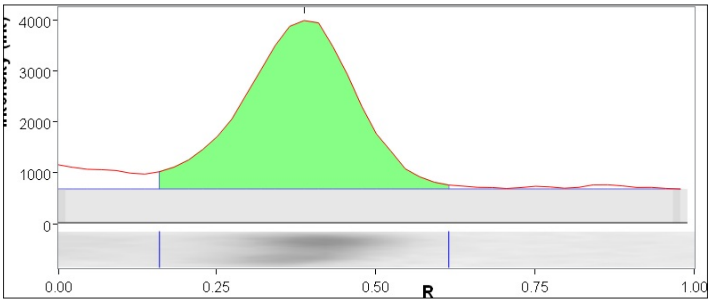

Band No.	Band Label	Mol. Wt. (KDa)	Relative Front	Adj. Volume (Int)	Volume (Int)	Abs. Quant.	Rel. Quant.	Band %	Lane %
1		N/A	0,409	1 207 920	1 756 230	N/A	N/A	100,0	90,4

Band Detection	Automatically detected bands with sensitivity: Low
Lane Background	Lane background subtracted with disk size: 24.1
Lane Width	4.71 mm

Lane 14

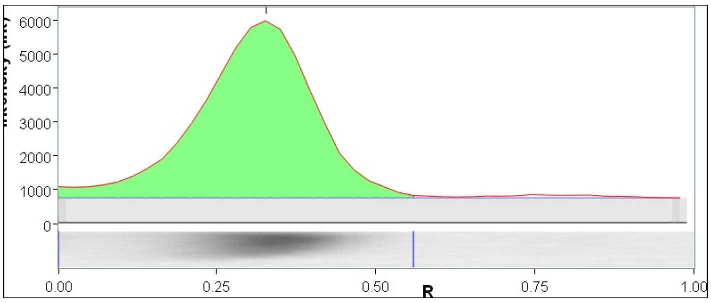

Band No.	Band Label	Mol. Wt. (KDa)	Relative Front	Adj. Volume (Int)	Volume (Int)	Abs. Quant.	Rel. Quant.	Band %	Lane %
1		N/A	0,349	1 921 880	2 661 880	N/A	N/A	100,0	98,1

Band Detection	Automatically detected bands with sensitivity: Low
Lane Background	Lane background subtracted with disk size: 24.1
Lane Width	5.38 mm

Image Report: Fig. 4g, Gapdh panel, males

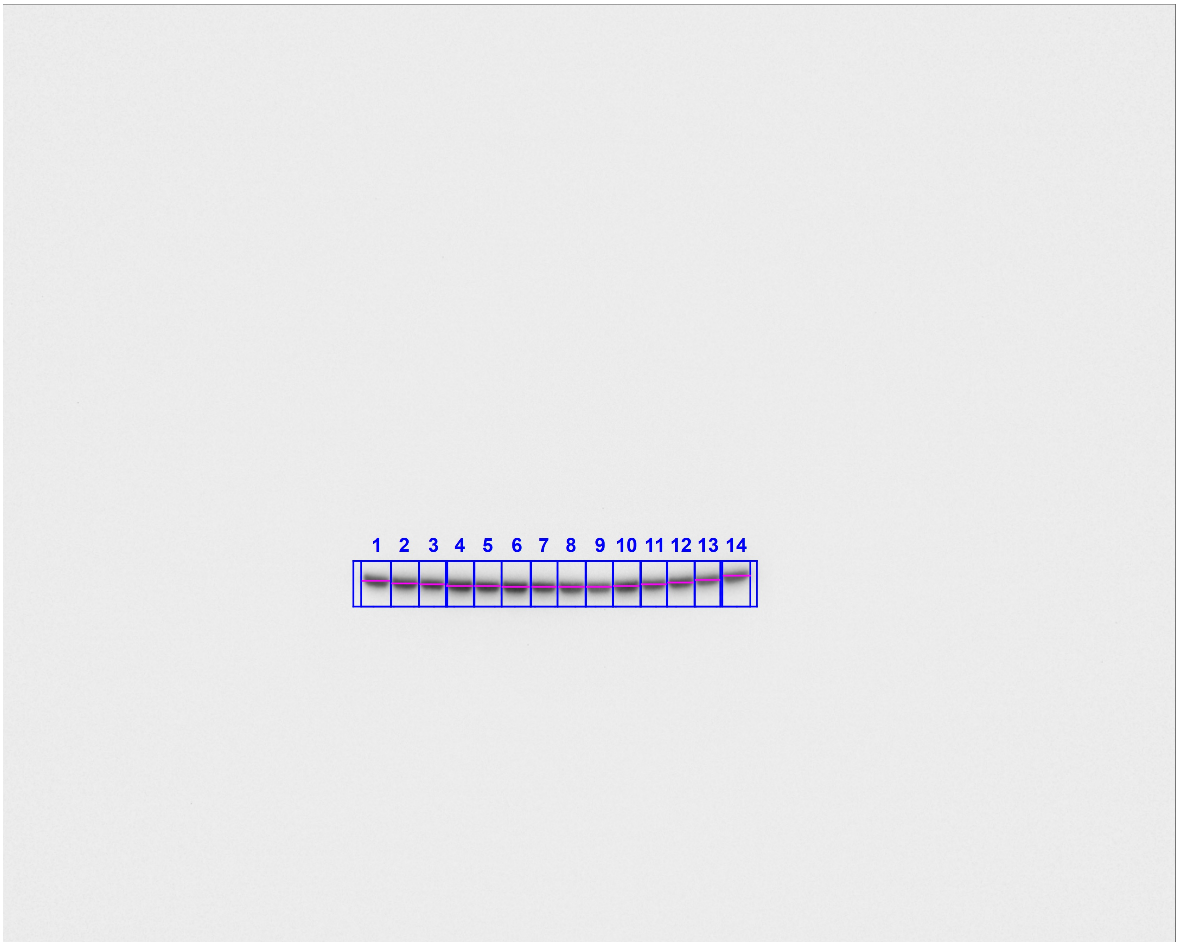

C:\Users\zahelb\OneDrive - KI.SE\Dokument\PhD\IDH2\Manuscript\Rivision\Wetsrn blotting\Ho-1, Nqo, Osgin males females\ChemiDoc Images 2022-10-07_14.13.17 Gapdh 2nd\gapdh males 20221007_1\gapdh males 20221007_4 quantified.scn

Acquisition Information

Imager	ChemiDoc Touch
Exposure Time (sec)	5.500 (Signal Accumulation)
Serial Number	732BR0263
Software Version	2.3.0.07
Application	Chemiluminescence
Excitation Source	No Illumination
Emission Filter	No Filter
Binning	2x2

Image Information

Acquisition Date	10/7/2022 10:18:21
User Name	m
Image Area (mm)	X: 207.1 Y: 165.8
Pixel Size (µm)	X: 150.2 Y: 150.2

Data Range (Int)	500 - 16098

Analysis Settings

Detection	Lane detection:<br>Manually created lanes (Copied)<br><br>Band detection:<br>Automatically detected bands with sensitivity: Low<br><br>Lane Background Subtraction:<br>Lane background subtracted with disk size: 24.1<br><br>Lane width: Variable

Lane Statistics

Lane No.	Adj. Total Band Vol. (Int)	Total Band Vol. (Int)	Adj. Total Lane Vol. (Int)	Total Lane Vol. (Int)	Bkgd. Vol. (Int)	Norm. Factor
1	4 785 690	5 664 645	4 878 195	6 289 850	1 411 655	N/A
2	5 544 858	6 635 211	5 628 975	7 190 832	1 561 857	N/A
3	5 336 958	6 413 286	5 457 705	7 042 299	1 584 594	N/A
4	6 361 245	7 536 276	6 462 984	8 059 821	1 596 837	N/A
5	5 635 008	6 652 224	5 765 920	7 263 488	1 497 568	N/A
6	6 432 188	7 536 712	6 561 218	8 143 374	1 582 156	N/A
7	5 237 264	6 124 794	5 392 140	6 736 114	1 343 974	N/A
8	5 156 250	6 066 390	5 284 983	6 663 195	1 378 212	N/A
9	4 248 448	5 020 384	4 378 624	5 618 400	1 239 776	N/A
10	5 684 316	6 564 426	5 827 371	7 160 109	1 332 738	N/A
11	4 580 870	5 458 852	4 700 220	6 068 839	1 368 619	N/A
12	4 861 536	5 879 616	4 957 344	6 499 008	1 541 664	N/A
13	4 005 420	4 803 360	4 158 180	5 439 720	1 281 540	N/A
14	4 451 403	5 311 977	4 556 442	5 897 925	1 341 483	N/A

Lane And Band Analysis

Lane 1

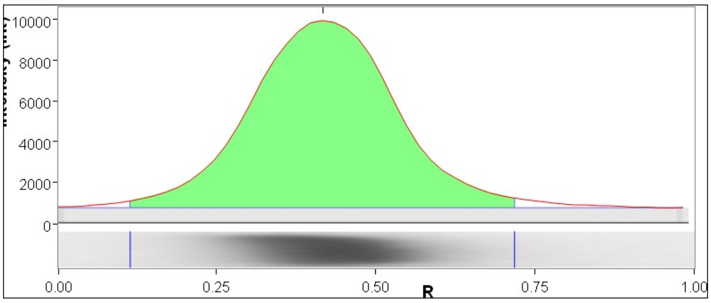

Band No.	Band Label	Mol. Wt. (KDa)	Relative Front	Adj. Volume (Int)	Volume (Int)	Abs. Quant.	Rel. Quant.	Band %	Lane %
1		N/A	0,434	4 785 690	5 664 645	N/A	N/A	100,0	98,1

Band Detection	Automatically detected bands with sensitivity: Low
Lane Background	Lane background subtracted with disk size: 24.1
Lane Width	5.26 mm

Lane 2

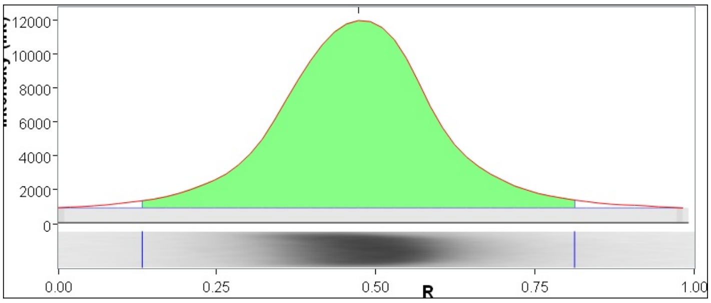

Band No.	Band Label	Mol. Wt. (KDa)	Relative Front	Adj. Volume (Int)	Volume (Int)	Abs. Quant.	Rel. Quant.	Band %	Lane %
1		N/A	0,491	5 544 858	6 635 211	N/A	N/A	100,0	98,5

Band Detection	Automatically detected bands with sensitivity: Low
Lane Background	Lane background subtracted with disk size: 24.1
Lane Width	4.96 mm

Lane 3

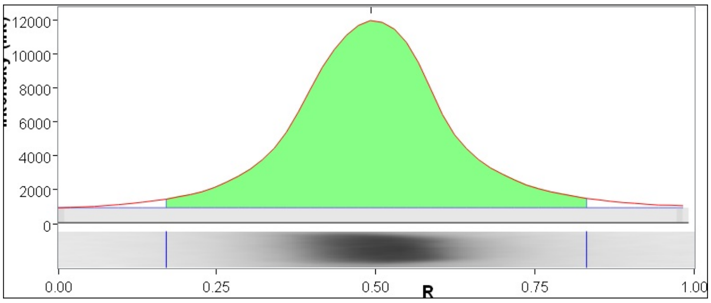

Band No.	Band Label	Mol. Wt. (KDa)	Relative Front	Adj. Volume (Int)	Volume (Int)	Abs. Quant.	Rel. Quant.	Band %	Lane %
1		N/A	0,509	5 336 958	6 413 286	N/A	N/A	100,0	97,8

Band Detection	Automatically detected bands with sensitivity: Low
Lane Background	Lane background subtracted with disk size: 24.1
Lane Width	4.96 mm

Lane 4

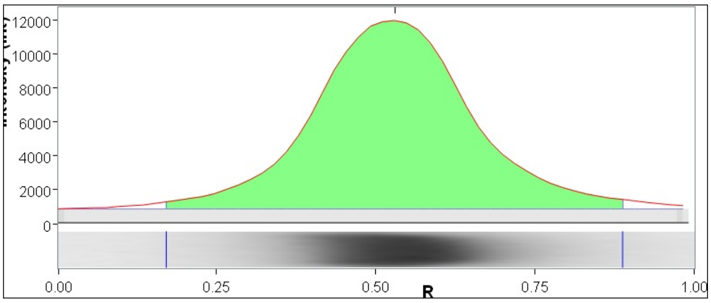

Band No.	Band Label	Mol. Wt. (KDa)	Relative Front	Adj. Volume (Int)	Volume (Int)	Abs. Quant.	Rel. Quant.	Band %	Lane %
1		N/A	0,547	6 361 245	7 536 276	N/A	N/A	100,0	98,4

Band Detection	Automatically detected bands with sensitivity: Low

Lane Background	Lane background subtracted with disk size: 24.1
Lane Width	4.96 mm

Lane 5

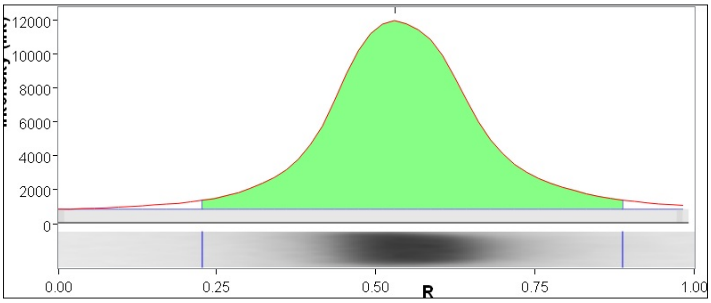

Band No.	Band Label	Mol. Wt. (KDa)	Relative Front	Adj. Volume (Int)	Volume (Int)	Abs. Quant.	Rel. Quant.	Band %	Lane %
1		N/A	0,547	5 635 008	6 652 224	N/A	N/A	100,0	97,7

Band Detection	Automatically detected bands with sensitivity: Low
Lane Background	Lane background subtracted with disk size: 24.1
Lane Width	4.80 mm

Lane 6

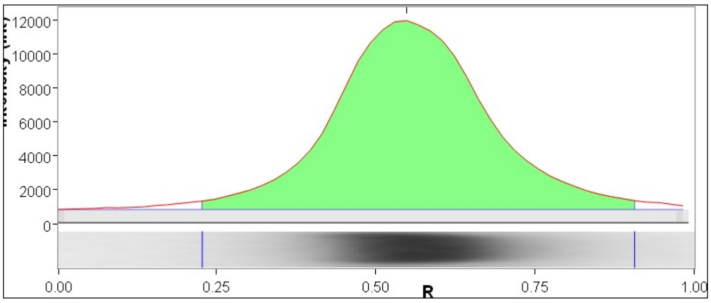

Band No.	Band Label	Mol. Wt. (KDa)	Relative Front	Adj. Volume (Int)	Volume (Int)	Abs. Quant.	Rel. Quant.	Band %	Lane %
1		N/A	0,566	6 432 188	7 536 712	N/A	N/A	100,0	98,0

Band Detection	Automatically detected bands with sensitivity: Low
Lane Background	Lane background subtracted with disk size: 24.1
Lane Width	5.11 mm

Lane 7

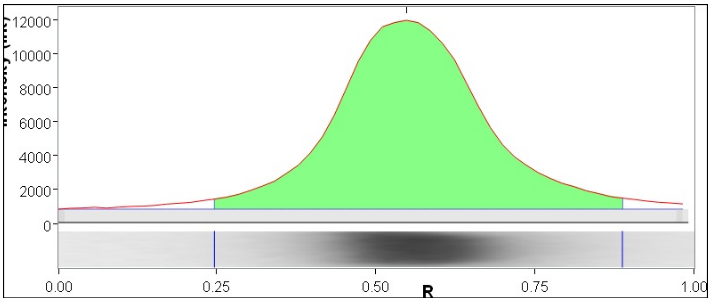

Band No.	Band Label	Mol. Wt. (KDa)	Relative Front	Adj. Volume (Int)	Volume (Int)	Abs. Quant.	Rel. Quant.	Band %	Lane %
1		N/A	0,566	5 237 264	6 124 794	N/A	N/A	100,0	97,1

Band Detection	Automatically detected bands with sensitivity: Low
Lane Background	Lane background subtracted with disk size: 24.1
Lane Width	4.65 mm

Lane 8

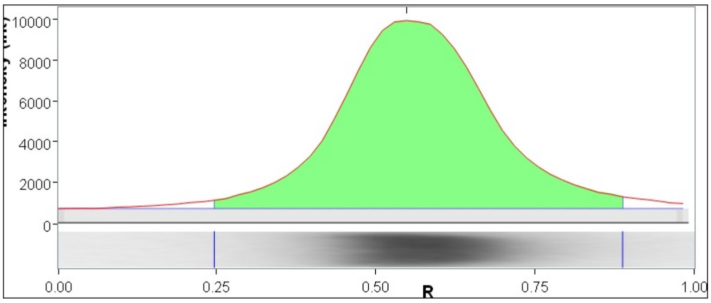

Band No.	Band Label	Mol. Wt. (KDa)	Relative Front	Adj. Volume (Int)	Volume (Int)	Abs. Quant.	Rel. Quant.	Band %	Lane %
1		N/A	0,566	5 156 250	6 066 390	N/A	N/A	100,0	97,6

Band Detection	Automatically detected bands with sensitivity: Low
Lane Background	Lane background subtracted with disk size: 24.1
Lane Width	4.96 mm

Lane 9

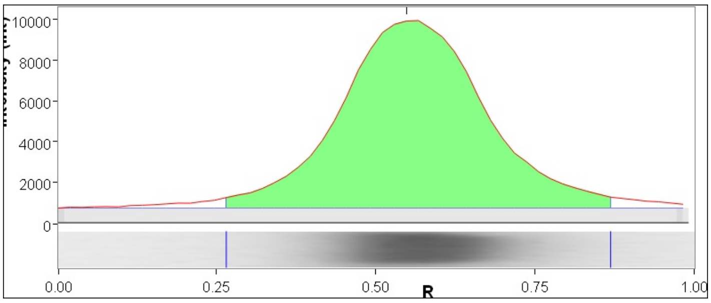

Band No.	Band Label	Mol. Wt. (KDa)	Relative Front	Adj. Volume (Int)	Volume (Int)	Abs. Quant.	Rel. Quant.	Band %	Lane %
1		N/A	0,566	4 248 448	5 020 384	N/A	N/A	100,0	97,0

Band Detection	Automatically detected bands with sensitivity: Low
Lane Background	Lane background subtracted with disk size: 24.1
Lane Width	4.80 mm

Lane 10

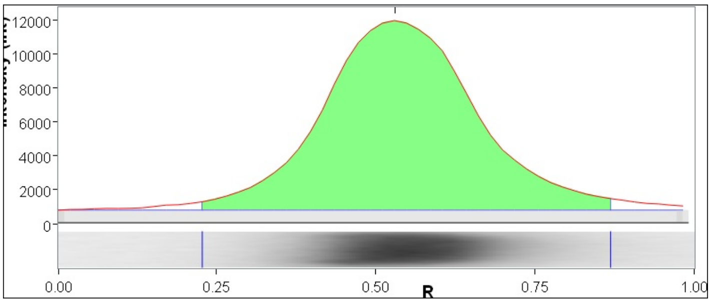

Band No.	Band Label	Mol. Wt. (KDa)	Relative Front	Adj. Volume (Int)	Volume (Int)	Abs. Quant.	Rel. Quant.	Band %	Lane %
1		N/A	0,547	5 684 316	6 564 426	N/A	N/A	100,0	97,5

Band Detection	Automatically detected bands with sensitivity: Low
Lane Background	Lane background subtracted with disk size: 24.1
Lane Width	4.96 mm

Lane 11

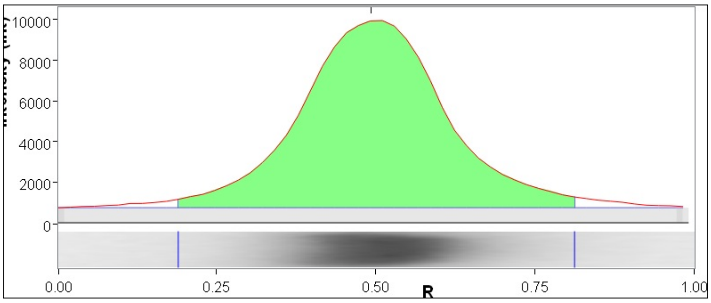

Band No.	Band Label	Mol. Wt. (KDa)	Relative Front	Adj. Volume (Int)	Volume (Int)	Abs. Quant.	Rel. Quant.	Band %	Lane %
1		N/A	0,509	4 580 870	5 458 852	N/A	N/A	100,0	97,5

Band Detection	Automatically detected bands with sensitivity: Low
Lane Background	Lane background subtracted with disk size: 24.1
Lane Width	4.65 mm

Lane 12

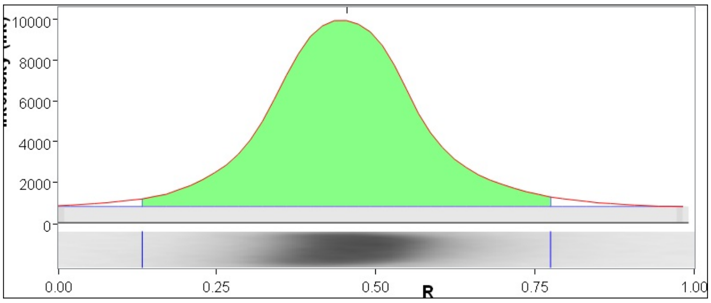

Band No.	Band Label	Mol. Wt. (KDa)	Relative Front	Adj. Volume (Int)	Volume (Int)	Abs. Quant.	Rel. Quant.	Band %	Lane %
1		N/A	0,472	4 861 536	5 879 616	N/A	N/A	100,0	98,1

Band Detection	Automatically detected bands with sensitivity: Low

Lane Background	Lane background subtracted with disk size: 24.1
Lane Width	4.80 mm

Lane 13

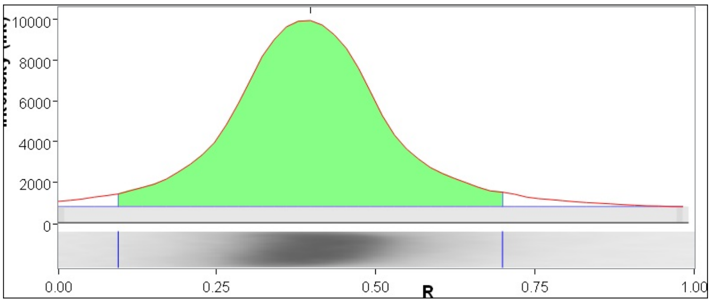

Band No.	Band Label	Mol. Wt. (KDa)	Relative Front	Adj. Volume (Int)	Volume (Int)	Abs. Quant.	Rel. Quant.	Band %	Lane %
1		N/A	0,415	4 005 420	4 803 360	N/A	N/A	100,0	96,3

Band Detection	Automatically detected bands with sensitivity: Low
Lane Background	Lane background subtracted with disk size: 24.1
Lane Width	4.50 mm

Lane 14

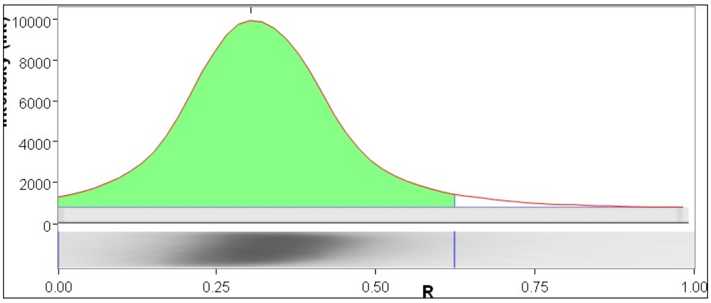

Band No.	Band Label	Mol. Wt. (KDa)	Relative Front	Adj. Volume (Int)	Volume (Int)	Abs. Quant.	Rel. Quant.	Band %	Lane %
1		N/A	0,321	4 451 403	5 311 977	N/A	N/A	100,0	97,7

Band Detection	Automatically detected bands with sensitivity: Low
Lane Background	Lane background subtracted with disk size: 24.1
Lane Width	4.96 mm

Image Report: Fig. 4g, Gapdh panel, females

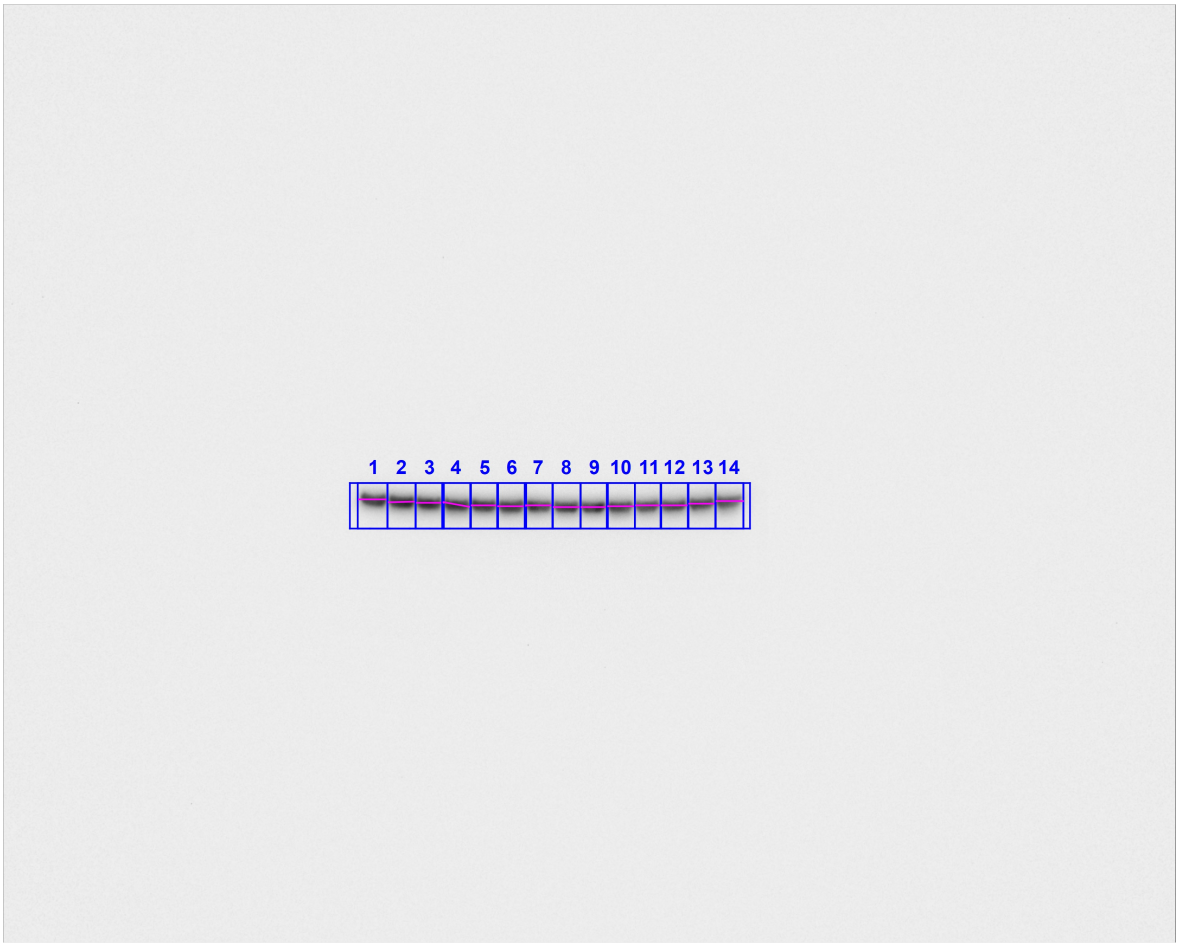

C:\Users\zahelb\OneDrive - KI.SE\Dokument\PhD\IDH2\Manuscript\Rivision\Wetsrn blotting\Ho-1, Nqo, Osgin males females\ChemiDoc Images 2022-10-07_14.13.17 Gapdh 2nd\gapdh females 20221007_1\gapdh females 20221007_5 quantified.scn

Acquisition Information

Imager	ChemiDoc Touch
Exposure Time (sec)	7.750 (Signal Accumulation)
Serial Number	732BR0263
Software Version	2.3.0.07
Application	Chemiluminescence
Excitation Source	No Illumination
Emission Filter	No Filter
Binning	2x2

Image Information

Acquisition Date	10/7/2022 10:02:27
User Name	m
Image Area (mm)	X: 207.1 Y: 165.8
Pixel Size (µm)	X: 150.2 Y: 150.2

Data Range (Int)	500 - 17614

Analysis Settings

Detection	Lane detection:<br>Manually created lanes (Copied)<br><br>Band detection:<br>Automatically detected bands with sensitivity: Low<br><br>Lane Background Subtraction:<br>Lane background subtracted with disk size: 24.1<br><br>Lane width: Variable

Lane Statistics

Lane No.	Adj. Total Band Vol. (Int)	Total Band Vol. (Int)	Adj. Total Lane Vol. (Int)	Total Lane Vol. (Int)	Bkgd. Vol. (Int)	Norm. Factor
1	6 287 050	7 312 690	6 391 595	7 860 755	1 469 160	N/A
2	7 104 801	8 273 001	7 203 075	8 750 940	1 547 865	N/A
3	6 699 924	7 883 700	6 807 933	8 458 989	1 651 056	N/A
4	6 560 268	7 849 380	6 650 061	8 448 033	1 797 972	N/A
5	6 301 696	7 405 184	6 411 744	7 992 416	1 580 672	N/A
6	6 526 334	7 713 682	6 655 942	8 311 980	1 656 038	N/A
7	5 893 107	6 981 018	6 021 708	7 580 067	1 558 359	N/A
8	5 837 337	6 850 701	5 978 808	7 470 705	1 491 897	N/A
9	5 801 472	6 762 240	5 917 824	7 332 288	1 414 464	N/A
10	5 736 423	6 863 769	5 823 114	7 395 465	1 572 351	N/A
11	5 058 983	6 106 907	5 175 574	6 718 351	1 542 777	N/A
12	5 383 008	6 524 640	5 486 656	7 167 392	1 680 736	N/A
13	5 643 744	6 794 080	5 752 736	7 357 152	1 604 416	N/A
14	5 189 283	6 333 723	5 289 042	6 805 425	1 516 383	N/A

Lane And Band Analysis

Lane 1

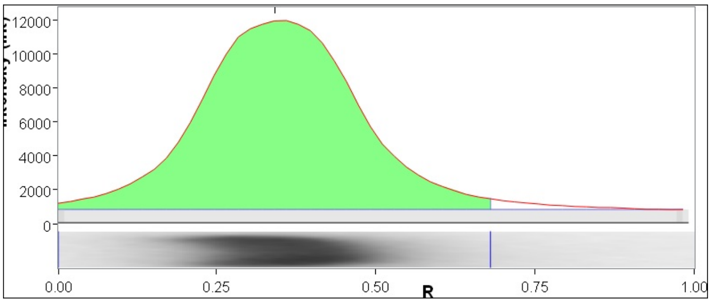

Band No.	Band Label	Mol. Wt. (KDa)	Relative Front	Adj. Volume (Int)	Volume (Int)	Abs. Quant.	Rel. Quant.	Band %	Lane %
1		N/A	0,358	6 287 050	7 312 690	N/A	N/A	100,0	98,4

Band Detection	Automatically detected bands with sensitivity: Low
Lane Background	Lane background subtracted with disk size: 24.1
Lane Width	5.26 mm

Lane 2

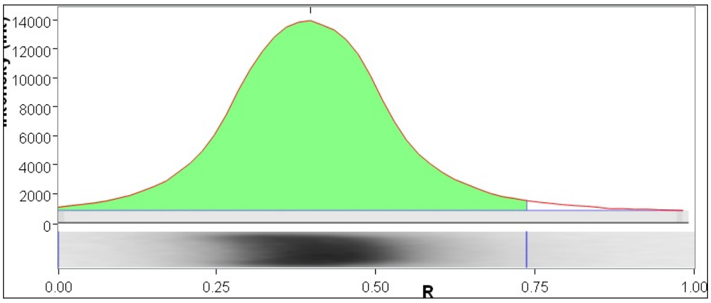

Band No.	Band Label	Mol. Wt. (KDa)	Relative Front	Adj. Volume (Int)	Volume (Int)	Abs. Quant.	Rel. Quant.	Band %	Lane %
1		N/A	0,415	7 104 801	8 273 001	N/A	N/A	100,0	98,6

Band Detection	Automatically detected bands with sensitivity: Low
Lane Background	Lane background subtracted with disk size: 24.1
Lane Width	4.96 mm

Lane 3

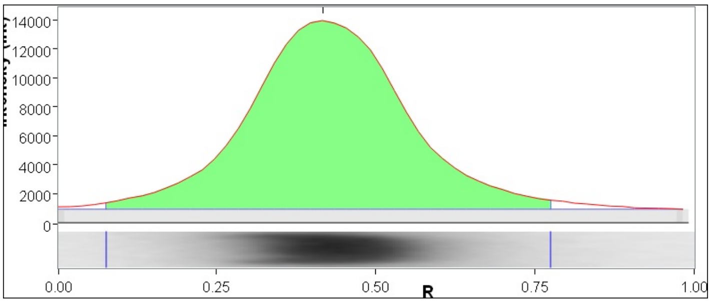

Band No.	Band Label	Mol. Wt. (KDa)	Relative Front	Adj. Volume (Int)	Volume (Int)	Abs. Quant.	Rel. Quant.	Band %	Lane %
1		N/A	0,434	6 699 924	7 883 700	N/A	N/A	100,0	98,4

Band Detection	Automatically detected bands with sensitivity: Low
Lane Background	Lane background subtracted with disk size: 24.1
Lane Width	4.96 mm

Lane 4

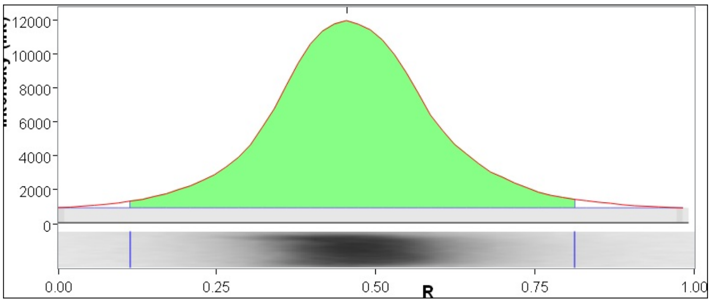

Band No.	Band Label	Mol. Wt. (KDa)	Relative Front	Adj. Volume (Int)	Volume (Int)	Abs. Quant.	Rel. Quant.	Band %	Lane %
1		N/A	0,472	6 560 268	7 849 380	N/A	N/A	100,0	98,6

Band Detection	Automatically detected bands with sensitivity: Low

Lane Background	Lane background subtracted with disk size: 24.1
Lane Width	4.96 mm

Lane 5

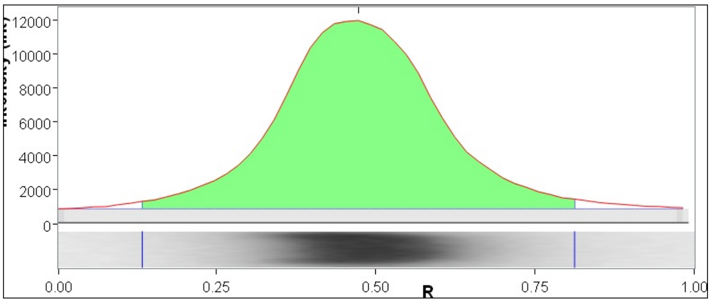

Band No.	Band Label	Mol. Wt. (KDa)	Relative Front	Adj. Volume (Int)	Volume (Int)	Abs. Quant.	Rel. Quant.	Band %	Lane %
1		N/A	0,491	6 301 696	7 405 184	N/A	N/A	100,0	98,3

Band Detection	Automatically detected bands with sensitivity: Low
Lane Background	Lane background subtracted with disk size: 24.1
Lane Width	4.80 mm

Lane 6

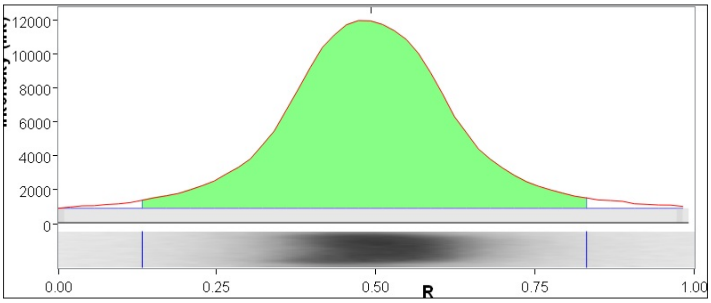

Band No.	Band Label	Mol. Wt. (KDa)	Relative Front	Adj. Volume (Int)	Volume (Int)	Abs. Quant.	Rel. Quant.	Band %	Lane %
1		N/A	0,509	6 526 334	7 713 682	N/A	N/A	100,0	98,1

Band Detection	Automatically detected bands with sensitivity: Low
Lane Background	Lane background subtracted with disk size: 24.1
Lane Width	5.11 mm

Lane 7

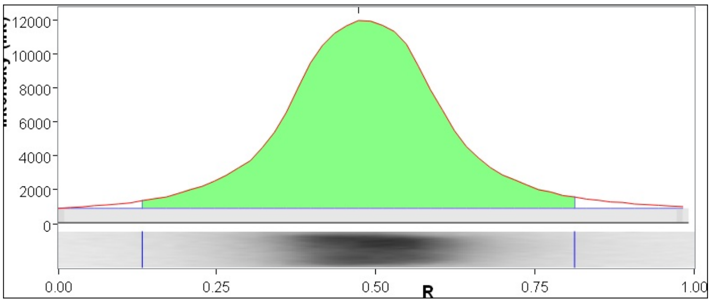

Band No.	Band Label	Mol. Wt. (KDa)	Relative Front	Adj. Volume (Int)	Volume (Int)	Abs. Quant.	Rel. Quant.	Band %	Lane %
1		N/A	0,491	5 893 107	6 981 018	N/A	N/A	100,0	97,9

Band Detection	Automatically detected bands with sensitivity: Low
Lane Background	Lane background subtracted with disk size: 24.1
Lane Width	4.96 mm

Lane 8

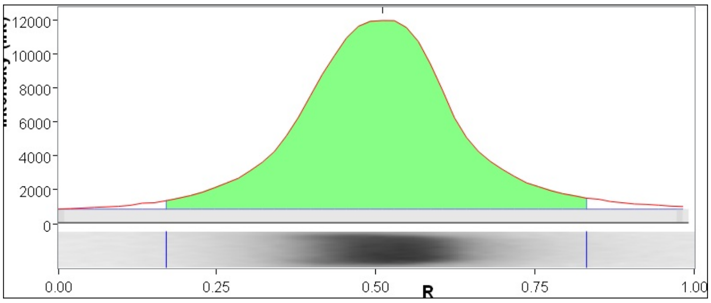

Band No.	Band Label	Mol. Wt. (KDa)	Relative Front	Adj. Volume (Int)	Volume (Int)	Abs. Quant.	Rel. Quant.	Band %	Lane %
1		N/A	0,528	5 837 337	6 850 701	N/A	N/A	100,0	97,6

Band Detection	Automatically detected bands with sensitivity: Low
Lane Background	Lane background subtracted with disk size: 24.1
Lane Width	4.96 mm

Lane 9

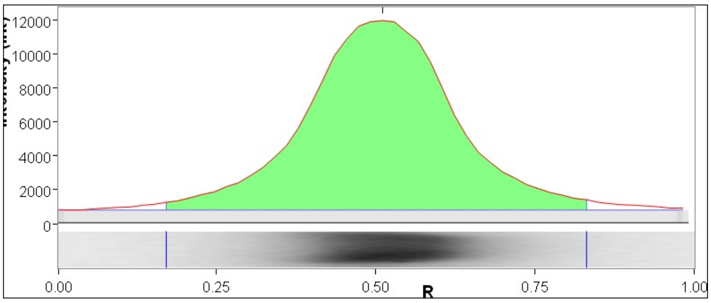

Band No.	Band Label	Mol. Wt. (KDa)	Relative Front	Adj. Volume (Int)	Volume (Int)	Abs. Quant.	Rel. Quant.	Band %	Lane %
1		N/A	0,528	5 801 472	6 762 240	N/A	N/A	100,0	98,0

Band Detection	Automatically detected bands with sensitivity: Low
Lane Background	Lane background subtracted with disk size: 24.1
Lane Width	4.80 mm

Lane 10

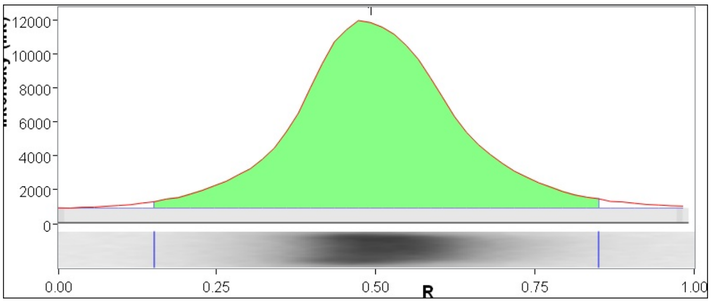

Band No.	Band Label	Mol. Wt. (KDa)	Relative Front	Adj. Volume (Int)	Volume (Int)	Abs. Quant.	Rel. Quant.	Band %	Lane %
1		N/A	0,509	5 736 423	6 863 769	N/A	N/A	100,0	98,5

Band Detection	Automatically detected bands with sensitivity: Low
Lane Background	Lane background subtracted with disk size: 24.1
Lane Width	4.96 mm

Lane 11

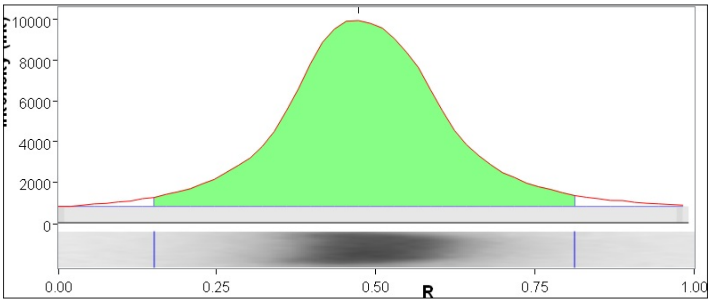

Band No.	Band Label	Mol. Wt. (KDa)	Relative Front	Adj. Volume (Int)	Volume (Int)	Abs. Quant.	Rel. Quant.	Band %	Lane %
1		N/A	0,491	5 058 983	6 106 907	N/A	N/A	100,0	97,7

Band Detection	Automatically detected bands with sensitivity: Low
Lane Background	Lane background subtracted with disk size: 24.1
Lane Width	4.65 mm

Lane 12

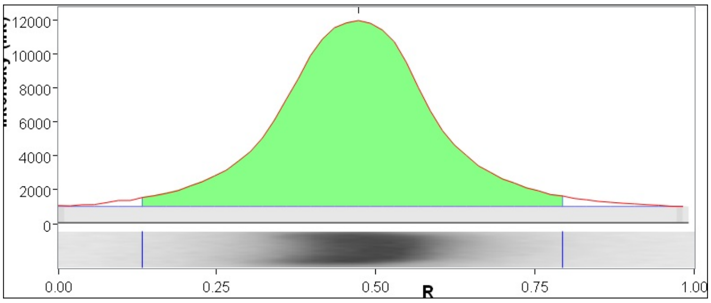

Band No.	Band Label	Mol. Wt. (KDa)	Relative Front	Adj. Volume (Int)	Volume (Int)	Abs. Quant.	Rel. Quant.	Band %	Lane %
1		N/A	0,491	5 383 008	6 524 640	N/A	N/A	100,0	98,1

Band Detection	Automatically detected bands with sensitivity: Low

Lane Background	Lane background subtracted with disk size: 24.1
Lane Width	4.80 mm

Lane 13

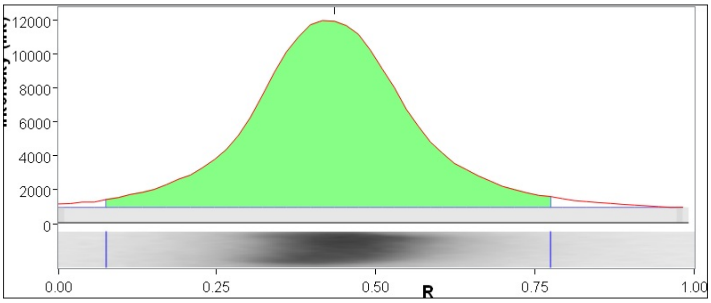

Band No.	Band Label	Mol. Wt. (KDa)	Relative Front	Adj. Volume (Int)	Volume (Int)	Abs. Quant.	Rel. Quant.	Band %	Lane %
1		N/A	0,453	5 643 744	6 794 080	N/A	N/A	100,0	98,1

Band Detection	Automatically detected bands with sensitivity: Low
Lane Background	Lane background subtracted with disk size: 24.1
Lane Width	4.80 mm

Lane 14

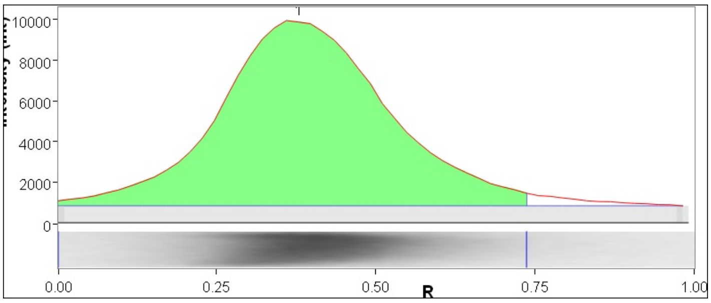

Band No.	Band Label	Mol. Wt. (KDa)	Relative Front	Adj. Volume (Int)	Volume (Int)	Abs. Quant.	Rel. Quant.	Band %	Lane %
1		N/A	0,396	5 189 283	6 333 723	N/A	N/A	100,0	98,1

Band Detection	Automatically detected bands with sensitivity: Low
Lane Background	Lane background subtracted with disk size: 24.1
Lane Width	4.96 mm

Image Report: Intensity analysis of Supplementary Fig. 1e (Oxphos_ individual oxphos panels, males)

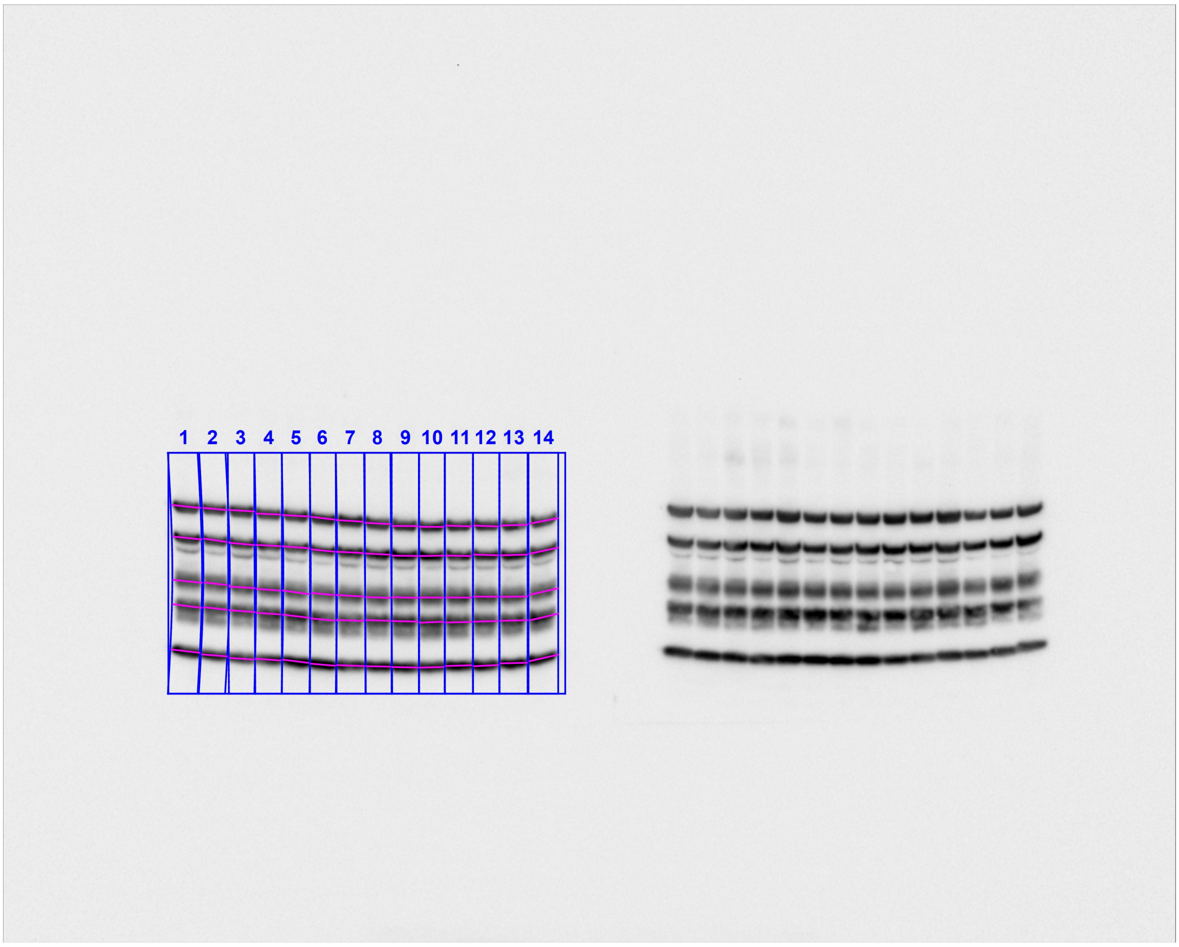

Acquisition Information

Imager	ChemiDoc Touch
Exposure Time (sec)	3.000 (Signal Accumulation)
Serial Number	732BR1240
Software Version	1.1.0.04
Application	Chemiluminescence
Excitation Source	No Illumination
Emission Filter	No Filter
Binning	2x2

Image Information

Acquisition Date	2/7/2021 3:25:24
User Name	
Image Area (mm)	X: 210.0 Y: 168.1
Pixel Size (µm)	X: 152.3 Y: 152.3

Data Range (Int)	500 - 26965

Analysis Settings

Detection	Lane detection:<br>Manually created lanes<br><br>Band detection:<br>Automatically detected bands with sensitivity: Low<br>Manually adjusted bands<br><br>Lane Background Subtraction:<br>Lane background subtracted with disk size: 32.8<br><br>Lane width: Variable

Lane Statistics

Lane No.	Adj. Total Band Vol. (Int)	Total Band Vol. (Int)	Adj. Total Lane Vol. (Int)	Total Lane Vol. (Int)	Bkgd. Vol. (Int)	Norm. Factor
1	34 031 200	37 416 995	36 355 060	43 058 155	6 703 095	N/A
2	29 609 805	32 578 830	31 232 562	37 103 683	5 871 121	N/A
3	29 869 089	32 834 022	32 109 707	37 855 092	5 745 385	N/A
4	30 869 472	34 160 864	32 757 952	39 275 776	6 517 824	N/A
5	34 326 666	37 712 367	36 223 539	42 804 927	6 581 388	N/A
6	33 422 929	36 833 797	34 799 546	41 264 782	6 465 236	N/A
7	35 067 566	38 744 564	37 056 498	44 072 738	7 016 240	N/A
8	34 059 142	37 482 968	35 894 311	42 359 826	6 465 515	N/A
9	36 160 512	39 633 728	38 127 968	44 675 296	6 547 328	N/A
10	35 147 040	38 362 770	36 806 130	42 846 780	6 040 650	N/A
11	36 023 790	39 523 473	37 986 729	44 691 768	6 705 039	N/A
12	33 474 451	36 681 959	35 363 436	41 502 149	6 138 713	N/A
13	35 153 778	38 636 862	37 180 011	43 953 294	6 773 283	N/A
14	33 961 690	37 590 420	35 754 390	42 944 335	7 189 945	N/A

Lane And Band Analysis

Lane 1

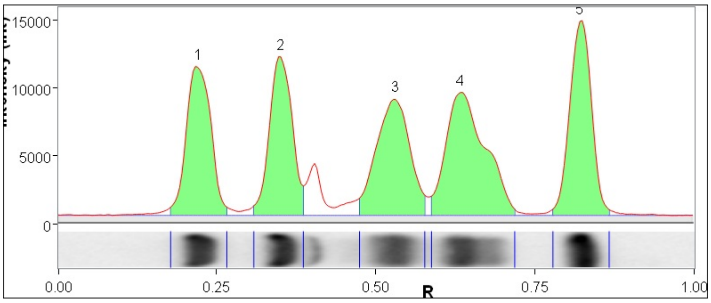

Band No.	Band Label	Mol. Wt. (KDa)	Relative Front	Adj. Volume (Int)	Volume (Int)	Abs. Quant.	Rel. Quant.	Band %	Lane %
1		N/A	0,223	6 148 835	6 767 285	N/A	N/A	18,1	16,9
2		N/A	0,353	6 094 305	6 640 340	N/A	N/A	17,9	16,8
3		N/A	0,534	6 568 800	7 279 125	N/A	N/A	19,3	18,1
4		N/A	0,636	8 117 200	9 015 335	N/A	N/A	23,9	22,3
5		N/A	0,823	7 102 060	7 714 910	N/A	N/A	20,9	19,5

Band Detection	Automatically detected bands with sensitivity: Low
Lane Background	Lane background subtracted with disk size: 32.8

Lane Width	5.33 mm

Lane 2

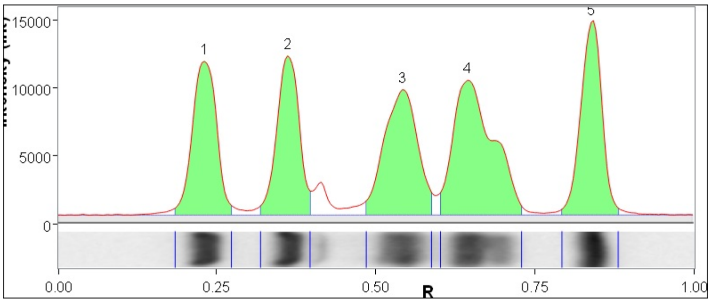

Band No.	Band Label	Mol. Wt. (KDa)	Relative Front	Adj. Volume (Int)	Volume (Int)	Abs. Quant.	Rel. Quant.	Band %	Lane %
1		N/A	0,233	5 250 811	5 787 514	N/A	N/A	17,7	16,8
2		N/A	0,364	5 027 146	5 503 089	N/A	N/A	17,0	16,1
3		N/A	0,544	5 906 089	6 528 879	N/A	N/A	19,9	18,9
4		N/A	0,647	7 643 422	8 434 263	N/A	N/A	25,8	24,5
5		N/A	0,841	5 782 337	6 325 085	N/A	N/A	19,5	18,5

Band Detection	Automatically detected bands with sensitivity: Low
Lane Background	Lane background subtracted with disk size: 32.8
Lane Width	4.72 mm

Lane 3

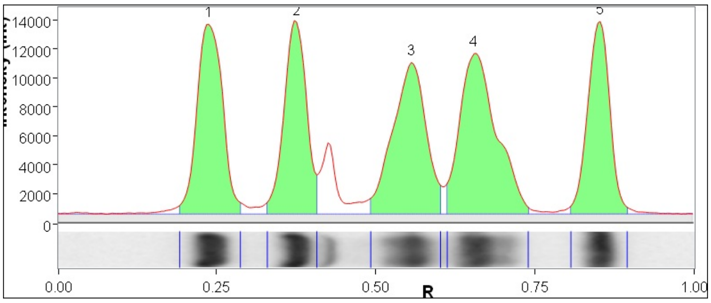

Band No.	Band Label	Mol. Wt. (KDa)	Relative Front	Adj. Volume (Int)	Volume (Int)	Abs. Quant.	Rel. Quant.	Band %	Lane %
1		N/A	0,240	5 954 449	6 522 121	N/A	N/A	19,9	18,5
2		N/A	0,378	5 332 899	5 799 201	N/A	N/A	17,9	16,6
3		N/A	0,558	6 167 884	6 817 644	N/A	N/A	20,6	19,2
4		N/A	0,657	7 234 625	7 986 809	N/A	N/A	24,2	22,5
5		N/A	0,855	5 179 232	5 708 247	N/A	N/A	17,3	16,1

Band Detection	Automatically detected bands with sensitivity: Low
Lane Background	Lane background subtracted with disk size: 32.8
Lane Width	4.72 mm

Lane 4

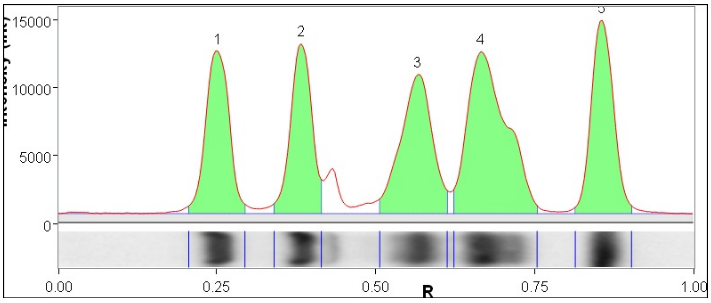

Band No.	Band Label	Mol. Wt. (KDa)	Relative Front	Adj. Volume (Int)	Volume (Int)	Abs. Quant.	Rel. Quant.	Band %	Lane %
1		N/A	0,254	5 246 208	5 846 656	N/A	N/A	17,0	16,0
2		N/A	0,385	4 979 584	5 487 008	N/A	N/A	16,1	15,2
3		N/A	0,569	6 036 736	6 750 240	N/A	N/A	19,6	18,4
4		N/A	0,668	8 709 760	9 583 232	N/A	N/A	28,2	26,6
5		N/A	0,859	5 897 184	6 493 728	N/A	N/A	19,1	18,0

Band Detection	Automatically detected bands with sensitivity: Low
Lane Background	Lane background subtracted with disk size: 32.8
Lane Width	4.87 mm

Lane 5

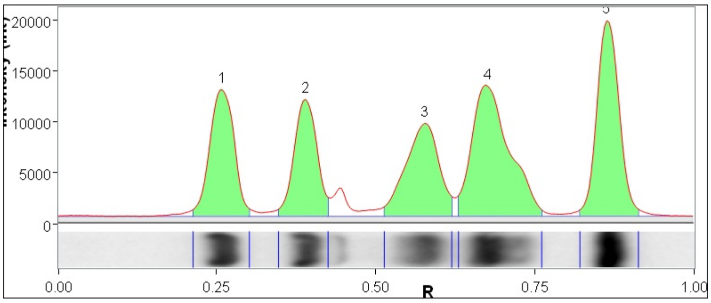

Band No.	Band Label	Mol. Wt. (KDa)	Relative Front	Adj. Volume (Int)	Volume (Int)	Abs. Quant.	Rel. Quant.	Band %	Lane %
1		N/A	0,261	5 871 063	6 466 251	N/A	N/A	17,1	16,2
2		N/A	0,392	5 080 977	5 611 815	N/A	N/A	14,8	14,0
3		N/A	0,580	5 861 229	6 584 787	N/A	N/A	17,1	16,2
4		N/A	0,678	9 309 234	10 203 336	N/A	N/A	27,1	25,7
5		N/A	0,866	8 204 163	8 846 178	N/A	N/A	23,9	22,6

Band Detection	Automatically detected bands with sensitivity: Low
Lane Background	Lane background subtracted with disk size: 32.8
Lane Width	5.03 mm

Lane 6

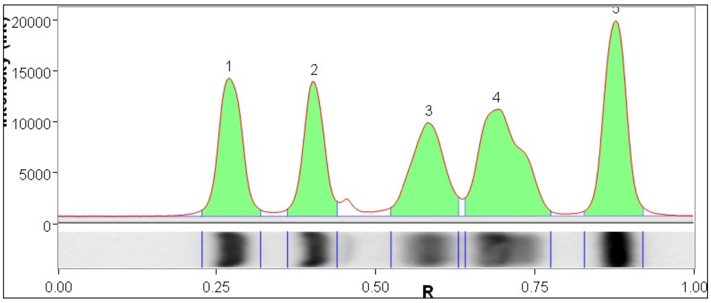

Band No.	Band Label	Mol. Wt. (KDa)	Relative Front	Adj. Volume (Int)	Volume (Int)	Abs. Quant.	Rel. Quant.	Band %	Lane %
1		N/A	0,272	5 985 232	6 586 849	N/A	N/A	17,9	17,2
2		N/A	0,406	5 139 645	5 659 329	N/A	N/A	15,4	14,8
3		N/A	0,587	5 695 723	6 409 560	N/A	N/A	17,0	16,4
4		N/A	0,693	8 460 923	9 371 424	N/A	N/A	25,3	24,3
5		N/A	0,880	8 141 406	8 806 635	N/A	N/A	24,4	23,4

Band Detection	Automatically detected bands with sensitivity: Low
Lane Background	Lane background subtracted with disk size: 32.8
Lane Width	4.72 mm

Lane 7

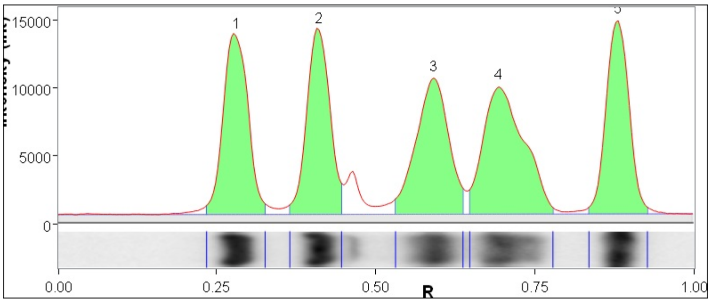

Band No.	Band Label	Mol. Wt. (KDa)	Relative Front	Adj. Volume (Int)	Volume (Int)	Abs. Quant.	Rel. Quant.	Band %	Lane %
1		N/A	0,283	6 890 678	7 546 470	N/A	N/A	19,6	18,6
2		N/A	0,413	6 590 458	7 180 460	N/A	N/A	18,8	17,8
3		N/A	0,594	6 805 678	7 580 878	N/A	N/A	19,4	18,4
4		N/A	0,696	7 989 864	8 951 758	N/A	N/A	22,8	21,6
5		N/A	0,883	6 790 888	7 484 998	N/A	N/A	19,4	18,3

Band Detection	Automatically detected bands with sensitivity: Low
Lane Background	Lane background subtracted with disk size: 32.8
Lane Width	5.18 mm

Lane 8

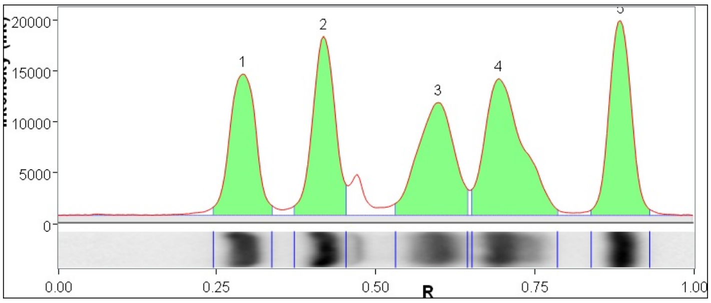

Band No.	Band Label	Mol. Wt. (KDa)	Relative Front	Adj. Volume (Int)	Volume (Int)	Abs. Quant.	Rel. Quant.	Band %	Lane %
1		N/A	0,293	5 893 503	6 515 921	N/A	N/A	17,3	16,4
2		N/A	0,420	6 409 374	6 960 430	N/A	N/A	18,8	17,9
3		N/A	0,601	6 568 373	7 321 766	N/A	N/A	19,3	18,3
4		N/A	0,696	8 317 238	9 203 807	N/A	N/A	24,4	23,2
5		N/A	0,887	6 870 654	7 481 044	N/A	N/A	20,2	19,1

Band Detection	Automatically detected bands with sensitivity: Low
Lane Background	Lane background subtracted with disk size: 32.8
Lane Width	4.72 mm

Lane 9

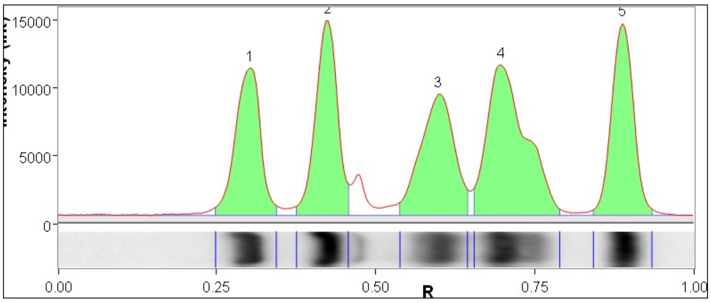

Band No.	Band Label	Mol. Wt. (KDa)	Relative Front	Adj. Volume (Int)	Volume (Int)	Abs. Quant.	Rel. Quant.	Band %	Lane %
1		N/A	0,304	6 034 144	6 677 312	N/A	N/A	16,7	15,8
2		N/A	0,428	6 987 072	7 540 128	N/A	N/A	19,3	18,3
3		N/A	0,601	6 672 736	7 390 400	N/A	N/A	18,5	17,5
4		N/A	0,700	9 615 040	10 544 352	N/A	N/A	26,6	25,2
5		N/A	0,890	6 851 520	7 481 536	N/A	N/A	18,9	18,0

Band Detection	Automatically detected bands with sensitivity: Low
Lane Background	Lane background subtracted with disk size: 32.8
Lane Width	4.87 mm

Lane 10

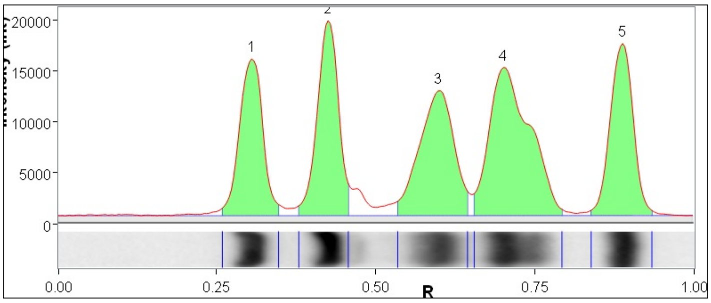

Band No.	Band Label	Mol. Wt. (KDa)	Relative Front	Adj. Volume (Int)	Volume (Int)	Abs. Quant.	Rel. Quant.	Band %	Lane %
1		N/A	0,307	5 802 480	6 352 920	N/A	N/A	16,5	15,8
2		N/A	0,428	6 666 000	7 176 570	N/A	N/A	19,0	18,1
3		N/A	0,601	6 852 810	7 538 430	N/A	N/A	19,5	18,6
4		N/A	0,703	9 568 140	10 429 920	N/A	N/A	27,2	26,0
5		N/A	0,890	6 257 610	6 864 930	N/A	N/A	17,8	17,0

Band Detection	Automatically detected bands with sensitivity: Low
Lane Background	Lane background subtracted with disk size: 32.8
Lane Width	4.57 mm

Lane 11

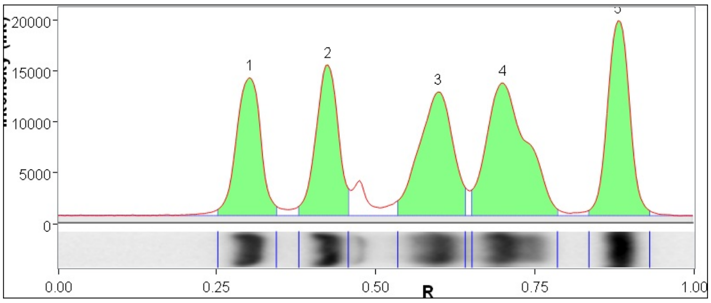

Band No.	Band Label	Mol. Wt. (KDa)	Relative Front	Adj. Volume (Int)	Volume (Int)	Abs. Quant.	Rel. Quant.	Band %	Lane %
1		N/A	0,304	5 883 570	6 525 915	N/A	N/A	16,3	15,5
2		N/A	0,428	5 909 772	6 455 691	N/A	N/A	16,4	15,6
3		N/A	0,601	7 348 671	8 081 832	N/A	N/A	20,4	19,3
4		N/A	0,703	9 344 874	10 264 848	N/A	N/A	25,9	24,6
5		N/A	0,883	7 536 903	8 195 187	N/A	N/A	20,9	19,8

Band Detection	Automatically detected bands with sensitivity: Low
Lane Background	Lane background subtracted with disk size: 32.8
Lane Width	5.03 mm

Lane 12

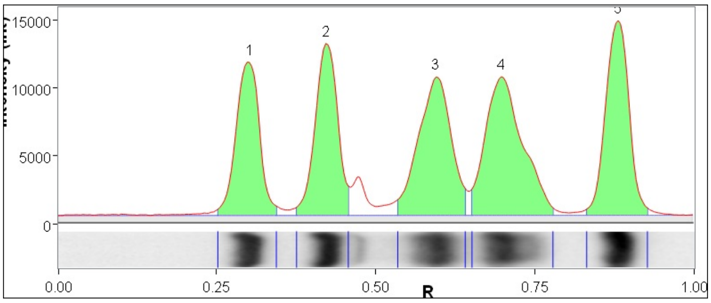

Band No.	Band Label	Mol. Wt. (KDa)	Relative Front	Adj. Volume (Int)	Volume (Int)	Abs. Quant.	Rel. Quant.	Band %	Lane %
1		N/A	0,304	5 549 310	6 127 615	N/A	N/A	16,6	15,7
2		N/A	0,424	5 984 519	6 502 405	N/A	N/A	17,9	16,9
3		N/A	0,597	6 984 920	7 661 092	N/A	N/A	20,9	19,8
4		N/A	0,700	7 966 907	8 780 316	N/A	N/A	23,8	22,5
5		N/A	0,883	6 988 795	7 610 531	N/A	N/A	20,9	19,8

Band Detection	Automatically detected bands with sensitivity: Low
Lane Background	Lane background subtracted with disk size: 32.8
Lane Width	4.72 mm

Lane 13

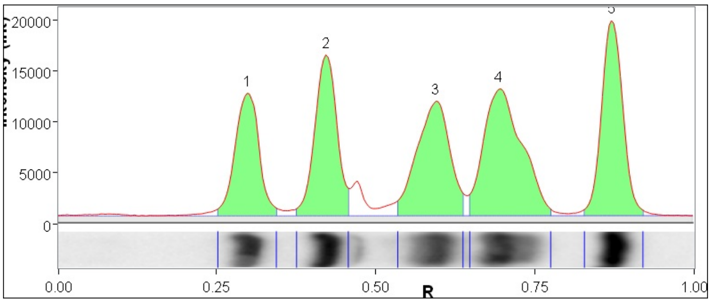

Band No.	Band Label	Mol. Wt. (KDa)	Relative Front	Adj. Volume (Int)	Volume (Int)	Abs. Quant.	Rel. Quant.	Band %	Lane %
1		N/A	0,300	5 361 939	6 002 106	N/A	N/A	15,3	14,4
2		N/A	0,424	6 505 752	7 077 807	N/A	N/A	18,5	17,5
3		N/A	0,597	6 871 062	7 591 551	N/A	N/A	19,5	18,5
4		N/A	0,696	8 877 000	9 770 607	N/A	N/A	25,3	23,9
5		N/A	0,873	7 538 025	8 194 791	N/A	N/A	21,4	20,3

Band Detection	Automatically detected bands with sensitivity: Low
Lane Background	Lane background subtracted with disk size: 32.8
Lane Width	5.03 mm

Lane 14

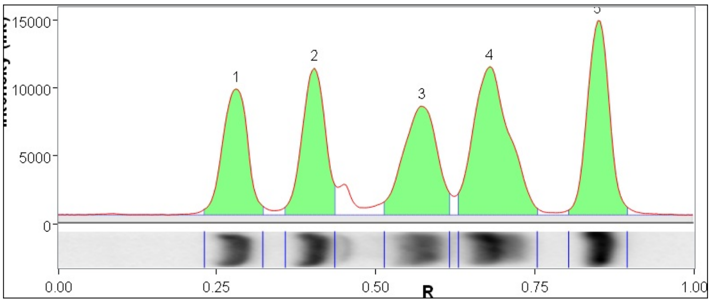

Band No.	Band Label	Mol. Wt. (KDa)	Relative Front	Adj. Volume (Int)	Volume (Int)	Abs. Quant.	Rel. Quant.	Band %	Lane %
1		N/A	0,283	5 399 415	6 086 570	N/A	N/A	15,9	15,1
2		N/A	0,406	5 817 210	6 401 850	N/A	N/A	17,1	16,3
3		N/A	0,576	6 285 510	7 046 760	N/A	N/A	18,5	17,6
4		N/A	0,682	9 136 785	10 049 235	N/A	N/A	26,9	25,6
5		N/A	0,852	7 322 770	8 006 005	N/A	N/A	21,6	20,5

Band Detection	Automatically detected bands with sensitivity: Low
Lane Background	Lane background subtracted with disk size: 32.8
Lane Width	5.33 mm

Image Report: Intensity analysis of Supplementary Fig.1e (Oxphos_ Total oxphos panel, males)

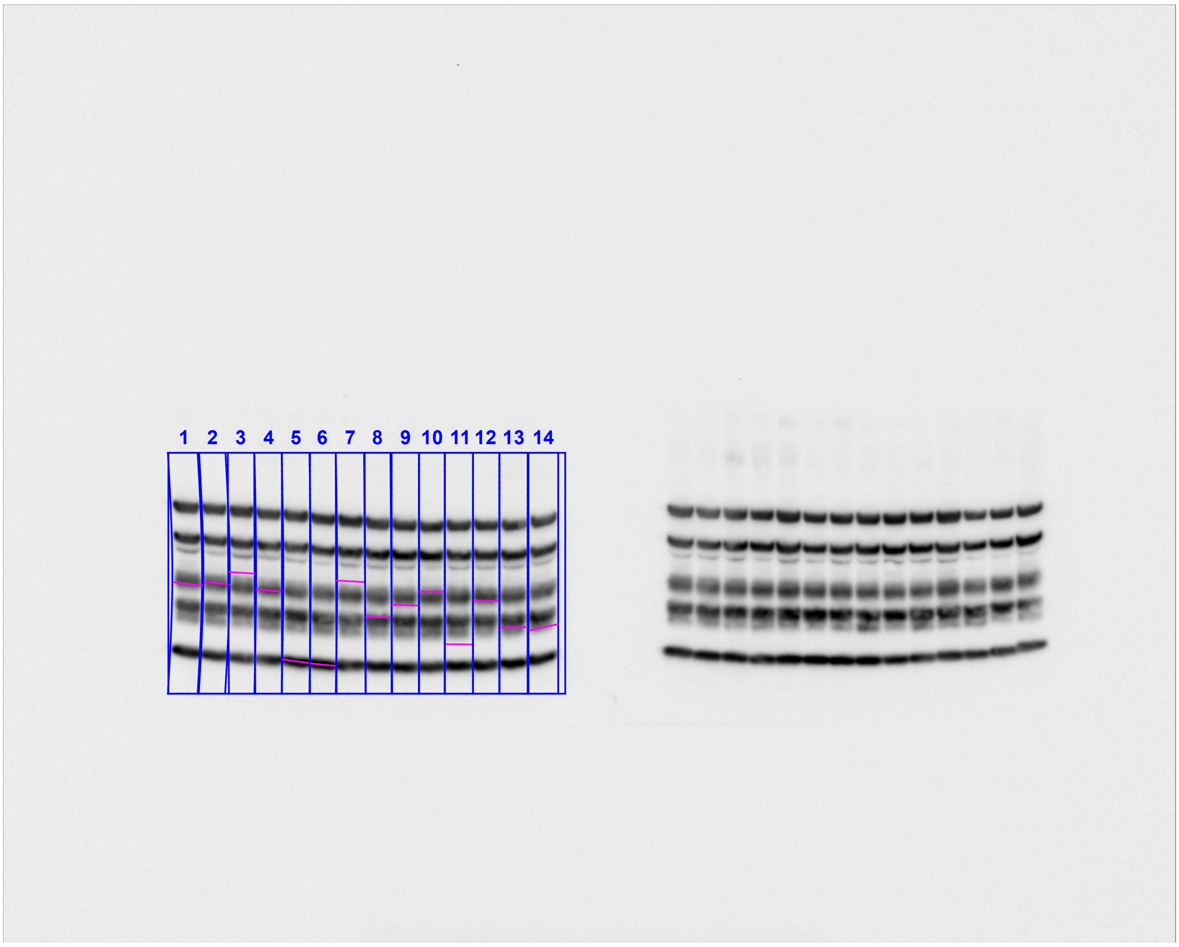

Acquisition Information

Imager	ChemiDoc Touch
Exposure Time (sec)	3.000 (Signal Accumulation)
Serial Number	732BR1240
Software Version	1.1.0.04
Application	Chemiluminescence
Excitation Source	No Illumination
Emission Filter	No Filter
Binning	2x2

Image Information

Acquisition Date	2/7/2021 3:25:24
User Name	
Image Area (mm)	X: 210.0 Y: 168.1
Pixel Size (µm)	X: 152.3 Y: 152.3

Data Range (Int)	500 - 26965

Analysis Settings

Detection	Lane detection:<br>Manually created lanes<br><br>Band detection:<br>Automatically detected bands with sensitivity: Low<br>Manually adjusted bands<br><br>Lane Background Subtraction:<br>Lane background subtracted with disk size: 25.1<br><br>Lane width: Variable

Lane Statistics

Lane No.	Adj. Total Band Vol. (Int)	Total Band Vol. (Int)	Adj. Total Lane Vol. (Int)	Total Lane Vol. (Int)	Bkgd. Vol. (Int)	Norm. Factor
1	35 661 920	41 145 860	35 793 730	43 048 320	7 254 590	N/A
2	30 547 989	35 452 840	30 649 638	37 101 234	6 451 596	N/A
3	31 362 390	36 273 007	31 502 572	37 850 101	6 347 529	N/A
4	32 051 040	37 524 224	32 165 152	39 272 512	7 107 360	N/A
5	35 240 007	41 136 018	35 373 525	42 797 172	7 423 647	N/A
6	32 882 196	39 731 832	32 945 374	41 270 455	8 325 081	N/A
7	36 219 384	42 397 354	36 312 714	44 077 396	7 764 682	N/A
8	35 813 339	40 590 811	35 946 577	42 357 191	6 410 614	N/A
9	37 155 904	42 868 480	37 289 024	44 675 392	7 386 368	N/A
10	36 581 850	41 143 170	36 731 040	42 857 340	6 126 300	N/A
11	37 903 437	42 829 182	38 030 487	44 705 628	6 675 141	N/A
12	34 352 309	39 731 894	34 491 468	41 513 092	7 021 624	N/A
13	36 971 022	41 943 429	37 211 493	43 953 294	6 741 801	N/A
14	35 589 855	40 847 205	35 723 835	42 912 660	7 188 825	N/A

Lane And Band Analysis

Lane 1

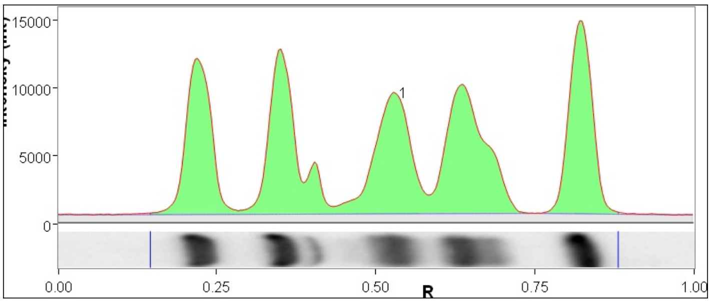

Band No.	Band Label	Mol. Wt. (KDa)	Relative Front	Adj. Volume (Int)	Volume (Int)	Abs. Quant.	Rel. Quant.	Band %	Lane %
1		N/A	0,544	35 661 920	41 145 860	N/A	N/A	100,0	99,6

Band Detection	Automatically detected bands with sensitivity: Low
Lane Background	Lane background subtracted with disk size: 25.1
Lane Width	5.33 mm

Lane 2

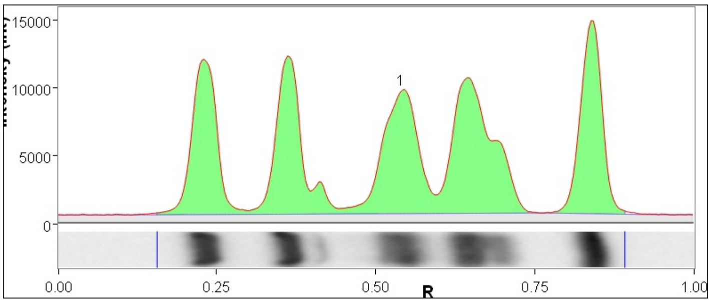

Band No.	Band Label	Mol. Wt. (KDa)	Relative Front	Adj. Volume (Int)	Volume (Int)	Abs. Quant.	Rel. Quant.	Band %	Lane %
1		N/A	0,541	30 547 989	35 452 840	N/A	N/A	100,0	99,7

Band Detection	Automatically detected bands with sensitivity: Low
Lane Background	Lane background subtracted with disk size: 25.1
Lane Width	4.72 mm

Lane 3

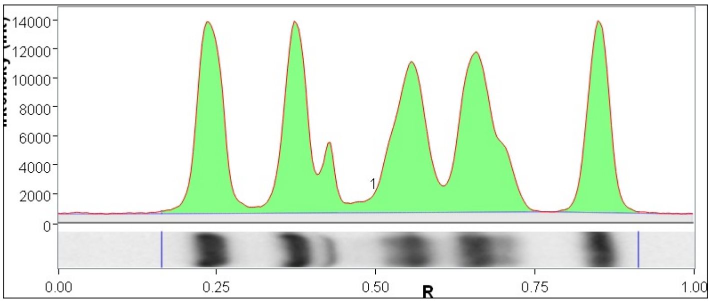

Band No.	Band Label	Mol. Wt. (KDa)	Relative Front	Adj. Volume (Int)	Volume (Int)	Abs. Quant.	Rel. Quant.	Band %	Lane %
1		N/A	0,498	31 362 390	36 273 007	N/A	N/A	100,0	99,6

Band Detection	Automatically detected bands with sensitivity: Low
Lane Background	Lane background subtracted with disk size: 25.1
Lane Width	4.72 mm

Lane 4

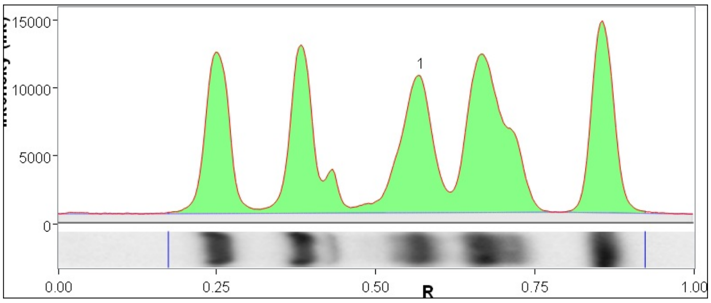

Band No.	Band Label	Mol. Wt. (KDa)	Relative Front	Adj. Volume (Int)	Volume (Int)	Abs. Quant.	Rel. Quant.	Band %	Lane %
1		N/A	0,572	32 051 040	37 524 224	N/A	N/A	100,0	99,6

Band Detection	Automatically detected bands with sensitivity: Low

Lane Background	Lane background subtracted with disk size: 25.1
Lane Width	4.87 mm

Lane 5

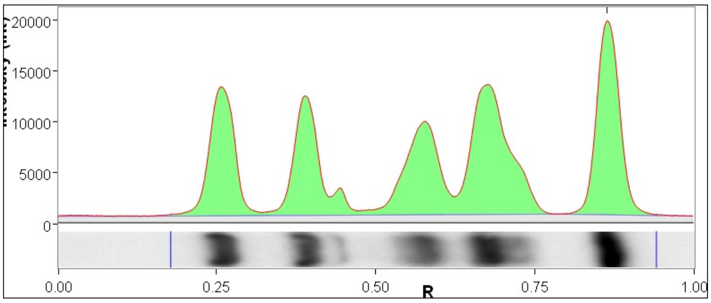

Band No.	Band Label	Mol. Wt. (KDa)	Relative Front	Adj. Volume (Int)	Volume (Int)	Abs. Quant.	Rel. Quant.	Band %	Lane %
1		N/A	0,866	35 240 007	41 136 018	N/A	N/A	100,0	99,6

Band Detection	Automatically detected bands with sensitivity: Low
Lane Background	Lane background subtracted with disk size: 25.1
Lane Width	5.03 mm

Lane 6

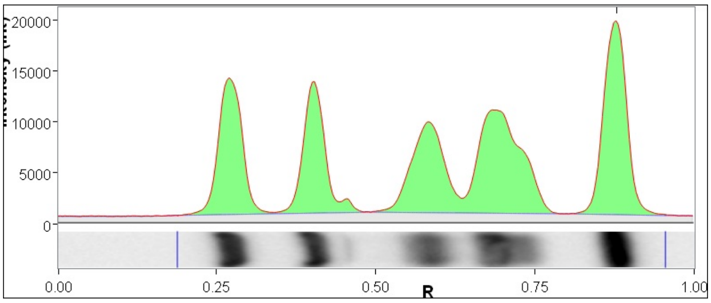

Band No.	Band Label	Mol. Wt. (KDa)	Relative Front	Adj. Volume (Int)	Volume (Int)	Abs. Quant.	Rel. Quant.	Band %	Lane %
1		N/A	0,880	32 882 196	39 731 832	N/A	N/A	100,0	99,8

Band Detection	Automatically detected bands with sensitivity: Low
Lane Background	Lane background subtracted with disk size: 25.1
Lane Width	4.72 mm

Lane 7

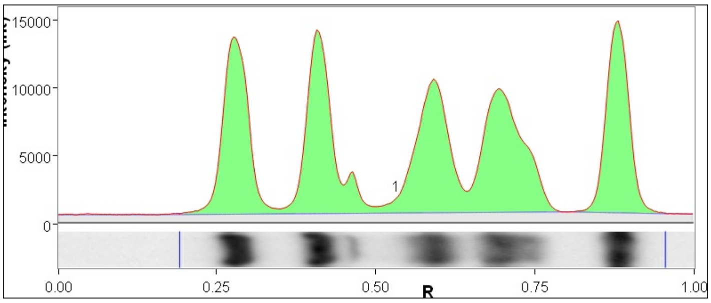

Band No.	Band Label	Mol. Wt. (KDa)	Relative Front	Adj. Volume (Int)	Volume (Int)	Abs. Quant.	Rel. Quant.	Band %	Lane %
1		N/A	0,534	36 219 384	42 397 354	N/A	N/A	100,0	99,7

Band Detection	Automatically detected bands with sensitivity: Low
Lane Background	Lane background subtracted with disk size: 25.1
Lane Width	5.18 mm

Lane 8

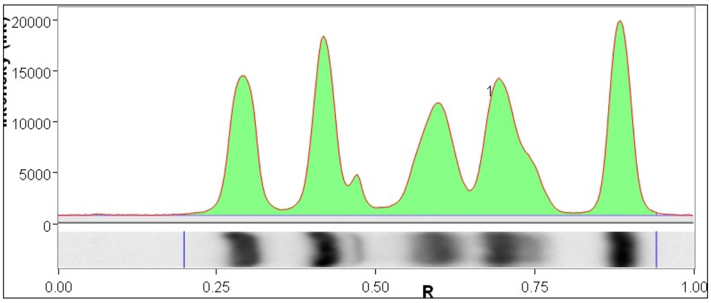

Band No.	Band Label	Mol. Wt. (KDa)	Relative Front	Adj. Volume (Int)	Volume (Int)	Abs. Quant.	Rel. Quant.	Band %	Lane %
1		N/A	0,682	35 813 339	40 590 811	N/A	N/A	100,0	99,6

Band Detection	Automatically detected bands with sensitivity: Low
Lane Background	Lane background subtracted with disk size: 25.1
Lane Width	4.72 mm

Lane 9

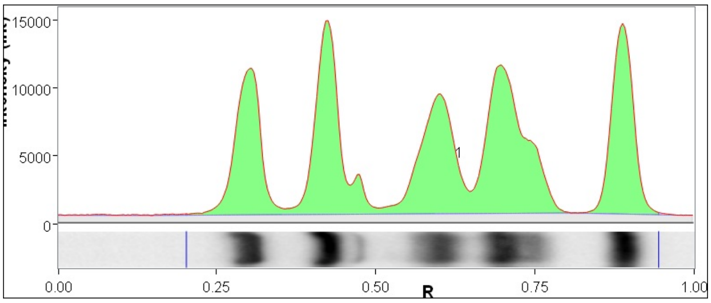

Band No.	Band Label	Mol. Wt. (KDa)	Relative Front	Adj. Volume (Int)	Volume (Int)	Abs. Quant.	Rel. Quant.	Band %	Lane %
1		N/A	0,633	37 155 904	42 868 480	N/A	N/A	100,0	99,6

Band Detection	Automatically detected bands with sensitivity: Low
Lane Background	Lane background subtracted with disk size: 25.1
Lane Width	4.87 mm

Lane 10

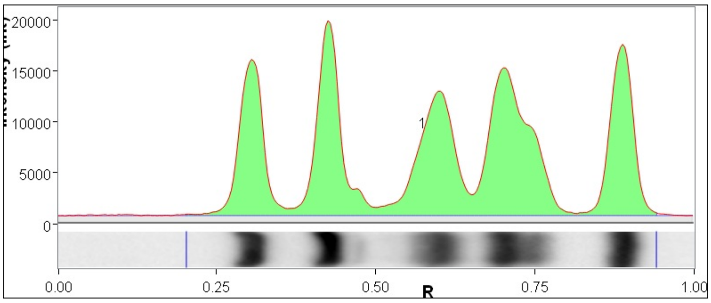

Band No.	Band Label	Mol. Wt. (KDa)	Relative Front	Adj. Volume (Int)	Volume (Int)	Abs. Quant.	Rel. Quant.	Band %	Lane %
1		N/A	0,576	36 581 850	41 143 170	N/A	N/A	100,0	99,6

Band Detection	Automatically detected bands with sensitivity: Low
Lane Background	Lane background subtracted with disk size: 25.1
Lane Width	4.57 mm

Lane 11

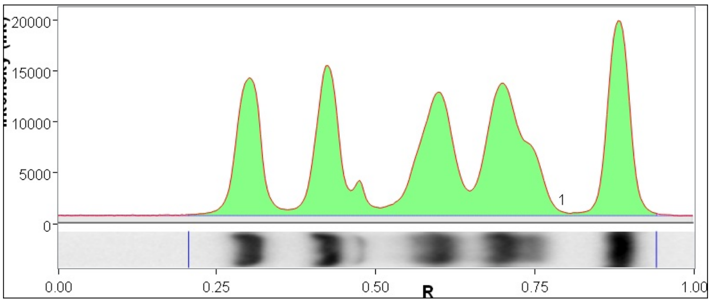

Band No.	Band Label	Mol. Wt. (KDa)	Relative Front	Adj. Volume (Int)	Volume (Int)	Abs. Quant.	Rel. Quant.	Band %	Lane %
1		N/A	0,795	37 903 437	42 829 182	N/A	N/A	100,0	99,7

Band Detection	Automatically detected bands with sensitivity: Low
Lane Background	Lane background subtracted with disk size: 25.1
Lane Width	5.03 mm

Lane 12

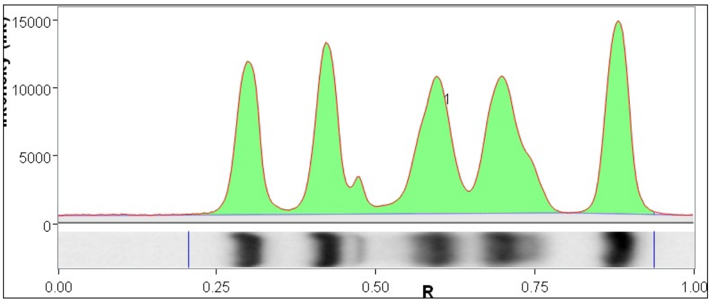

Band No.	Band Label	Mol. Wt. (KDa)	Relative Front	Adj. Volume (Int)	Volume (Int)	Abs. Quant.	Rel. Quant.	Band %	Lane %
1		N/A	0,615	34 352 309	39 731 894	N/A	N/A	100,0	99,6

Band Detection	Automatically detected bands with sensitivity: Low

Lane Background	Lane background subtracted with disk size: 25.1
Lane Width	4.72 mm

Lane 13

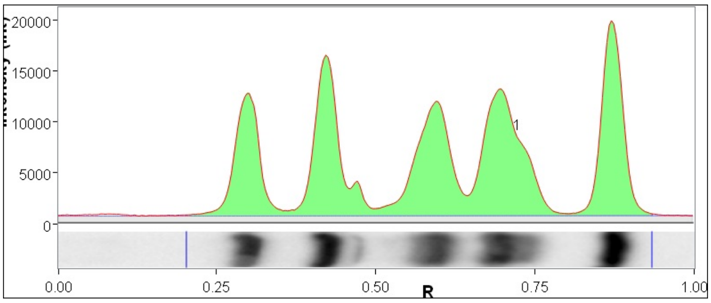

Band No.	Band Label	Mol. Wt. (KDa)	Relative Front	Adj. Volume (Int)	Volume (Int)	Abs. Quant.	Rel. Quant.	Band %	Lane %
1		N/A	0,724	36 971 022	41 943 429	N/A	N/A	100,0	99,4

Band Detection	Automatically detected bands with sensitivity: Low
Lane Background	Lane background subtracted with disk size: 25.1
Lane Width	5.03 mm

Lane 14

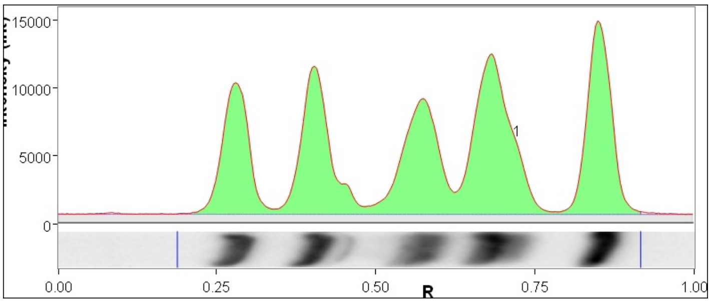

Band No.	Band Label	Mol. Wt. (KDa)	Relative Front	Adj. Volume (Int)	Volume (Int)	Abs. Quant.	Rel. Quant.	Band %	Lane %
1		N/A	0,724	35 589 855	40 847 205	N/A	N/A	100,0	99,6

Band Detection	Automatically detected bands with sensitivity: Low
Lane Background	Lane background subtracted with disk size: 25.1
Lane Width	5.33 mm

Image Report: Intensity analysis of Supplementary Fig. 1e (Oxphos_ individual oxphos panels, females)

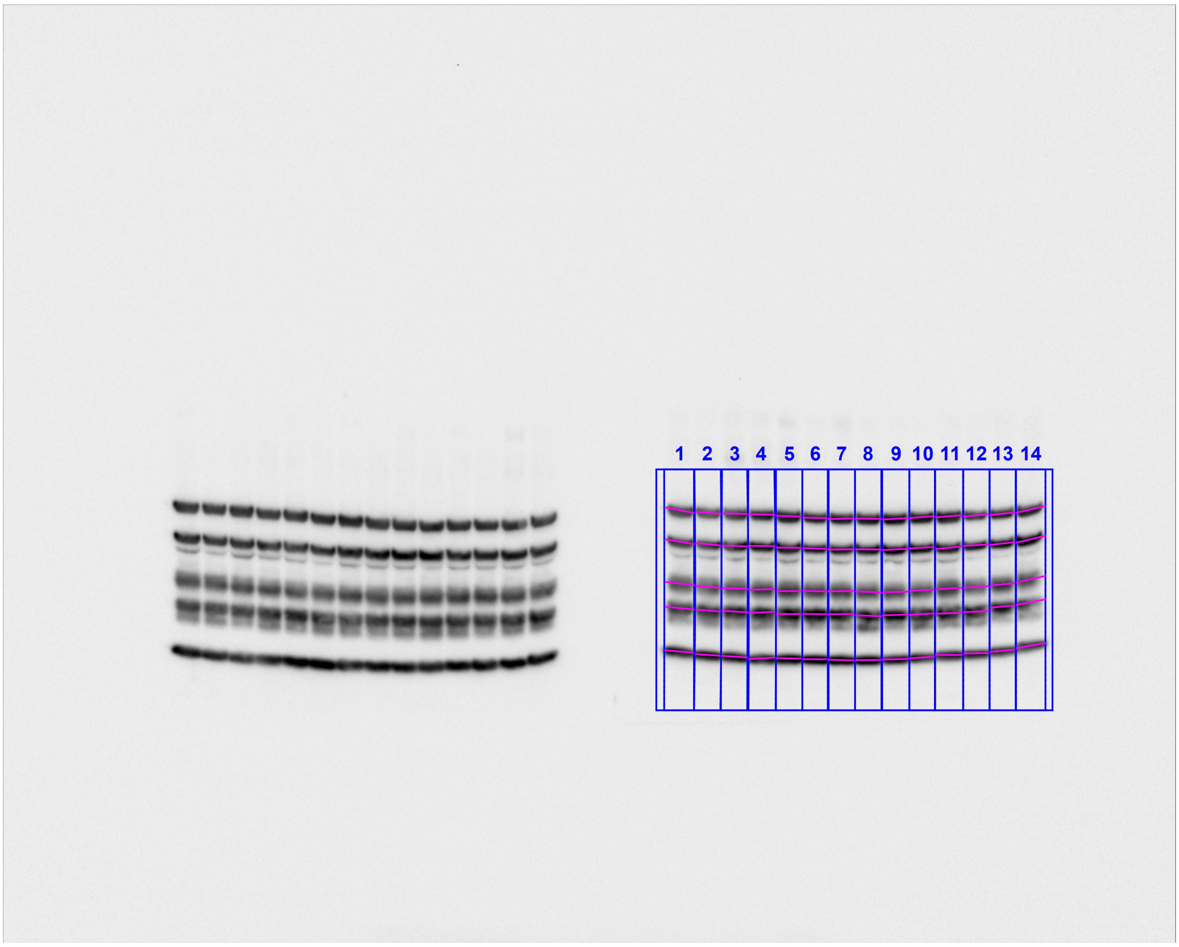

Acquisition Information

Imager	ChemiDoc Touch
Exposure Time (sec)	3.000 (Signal Accumulation)
Serial Number	732BR1240
Software Version	1.1.0.04
Application	Chemiluminescence
Excitation Source	No Illumination
Emission Filter	No Filter
Binning	2x2

Image Information

Acquisition Date	2/7/2021 3:25:24
User Name	
Image Area (mm)	X: 210.0 Y: 168.1
Pixel Size (µm)	X: 152.3 Y: 152.3

Data Range (Int)	500 - 26965

Analysis Settings

Detection	Lane detection:<br>Manually created lanes<br><br>Band detection:<br>Automatically detected bands with sensitivity: Low<br>Manually adjusted bands<br><br>Lane Background Subtraction:<br>Lane background subtracted with disk size: 23.2<br><br>Lane width: Variable

Lane Statistics

Lane No.	Adj. Total Band Vol. (Int)	Total Band Vol. (Int)	Adj. Total Lane Vol. (Int)	Total Lane Vol. (Int)	Bkgd. Vol. (Int)	Norm. Factor
1	28 904 750	34 216 630	29 809 955	39 621 365	9 811 410	N/A
2	27 421 794	32 097 431	28 477 437	37 153 965	8 676 528	N/A
3	30 351 077	35 731 933	31 468 503	41 034 111	9 565 608	N/A
4	32 989 248	38 120 736	33 911 968	43 129 888	9 217 920	N/A
5	35 812 440	41 667 131	36 952 744	47 037 013	10 084 269	N/A
6	33 799 703	38 609 539	34 739 530	43 584 140	8 844 610	N/A
7	33 505 216	38 566 336	34 675 552	43 985 504	9 309 952	N/A
8	34 024 329	39 028 287	35 027 892	44 032 710	9 004 818	N/A
9	33 470 784	38 335 168	34 640 832	43 698 464	9 057 632	N/A
10	31 451 190	36 159 480	32 464 380	40 953 240	8 488 860	N/A
11	35 690 853	38 999 070	37 868 061	44 397 936	6 529 875	N/A
12	26 749 404	30 599 325	27 517 429	35 127 433	7 610 004	N/A
13	28 722 399	32 862 976	29 339 175	37 158 770	7 819 595	N/A
14	30 100 490	33 612 460	31 640 595	38 655 995	7 015 400	N/A

Lane And Band Analysis

Lane 1

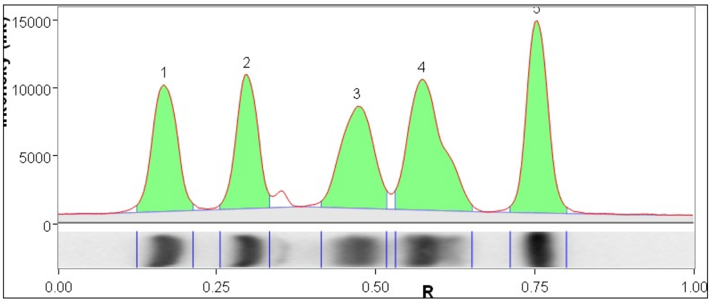

Band No.	Band Label	Mol. Wt. (KDa)	Relative Front	Adj. Volume (Int)	Volume (Int)	Abs. Quant.	Rel. Quant.	Band %	Lane %
1		N/A	0,170	5 034 120	5 928 475	N/A	N/A	17,4	16,9
2		N/A	0,300	4 637 080	5 627 755	N/A	N/A	16,0	15,6
3		N/A	0,473	5 366 445	6 702 815	N/A	N/A	18,6	18,0
4		N/A	0,576	7 274 785	8 589 280	N/A	N/A	25,2	24,4
5		N/A	0,756	6 592 320	7 368 305	N/A	N/A	22,8	22,1

Band Detection	Automatically detected bands with sensitivity: Low
Lane Background	Lane background subtracted with disk size: 23.2

Lane Width	5.33 mm

Lane 2

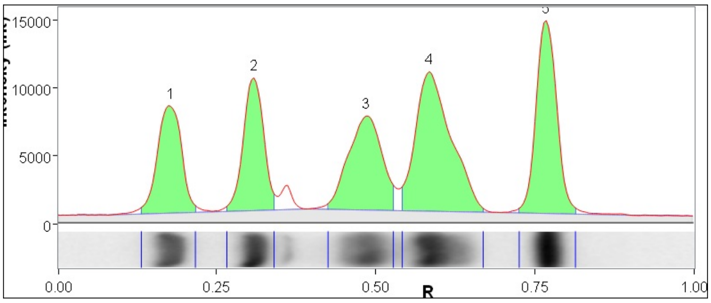

Band No.	Band Label	Mol. Wt. (KDa)	Relative Front	Adj. Volume (Int)	Volume (Int)	Abs. Quant.	Rel. Quant.	Band %	Lane %
1		N/A	0,180	4 085 645	4 807 015	N/A	N/A	14,9	14,3
2		N/A	0,311	4 271 490	5 072 995	N/A	N/A	15,6	15,0
3		N/A	0,488	4 971 811	6 134 621	N/A	N/A	18,1	17,5
4		N/A	0,587	7 819 192	9 083 031	N/A	N/A	28,5	27,5
5		N/A	0,770	6 273 656	6 999 769	N/A	N/A	22,9	22,0

Band Detection	Automatically detected bands with sensitivity: Low
Lane Background	Lane background subtracted with disk size: 23.2
Lane Width	4.72 mm

Lane 3

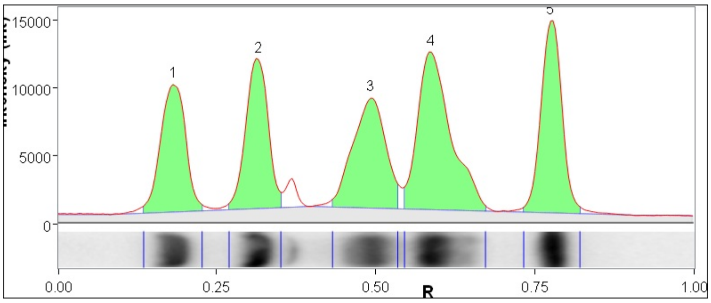

Band No.	Band Label	Mol. Wt. (KDa)	Relative Front	Adj. Volume (Int)	Volume (Int)	Abs. Quant.	Rel. Quant.	Band %	Lane %
1		N/A	0,184	5 002 377	5 866 006	N/A	N/A	16,5	15,9
2		N/A	0,318	5 255 957	6 265 875	N/A	N/A	17,3	16,7
3		N/A	0,495	5 622 718	6 955 842	N/A	N/A	18,5	17,9
4		N/A	0,590	8 203 189	9 610 372	N/A	N/A	27,0	26,1
5		N/A	0,777	6 266 836	7 033 838	N/A	N/A	20,6	19,9

Band Detection	Automatically detected bands with sensitivity: Low
Lane Background	Lane background subtracted with disk size: 23.2
Lane Width	4.72 mm

Lane 4

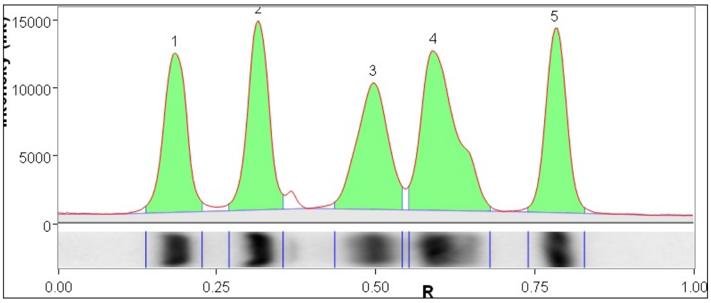

Band No.	Band Label	Mol. Wt. (KDa)	Relative Front	Adj. Volume (Int)	Volume (Int)	Abs. Quant.	Rel. Quant.	Band %	Lane %
1		N/A	0,187	5 744 992	6 551 616	N/A	N/A	17,4	16,9
2		N/A	0,318	6 212 064	7 168 512	N/A	N/A	18,8	18,3
3		N/A	0,498	6 168 512	7 410 272	N/A	N/A	18,7	18,2
4		N/A	0,594	8 852 928	10 198 944	N/A	N/A	26,8	26,1
5		N/A	0,784	6 010 752	6 791 392	N/A	N/A	18,2	17,7

Band Detection	Automatically detected bands with sensitivity: Low
Lane Background	Lane background subtracted with disk size: 23.2
Lane Width	4.87 mm

Lane 5

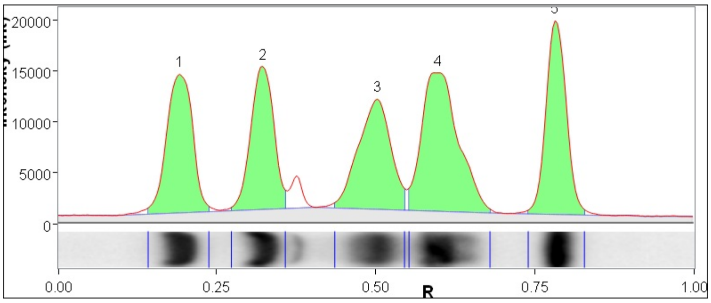

Band No.	Band Label	Mol. Wt. (KDa)	Relative Front	Adj. Volume (Int)	Volume (Int)	Abs. Quant.	Rel. Quant.	Band %	Lane %
1		N/A	0,194	6 602 597	7 568 681	N/A	N/A	18,4	17,9
2		N/A	0,325	6 075 287	7 227 991	N/A	N/A	17,0	16,4
3		N/A	0,505	6 562 638	8 099 711	N/A	N/A	18,3	17,8
4		N/A	0,601	9 386 180	10 828 455	N/A	N/A	26,2	25,4
5		N/A	0,784	7 185 738	7 942 293	N/A	N/A	20,1	19,4

Band Detection	Automatically detected bands with sensitivity: Low
Lane Background	Lane background subtracted with disk size: 23.2
Lane Width	4.72 mm

Lane 6

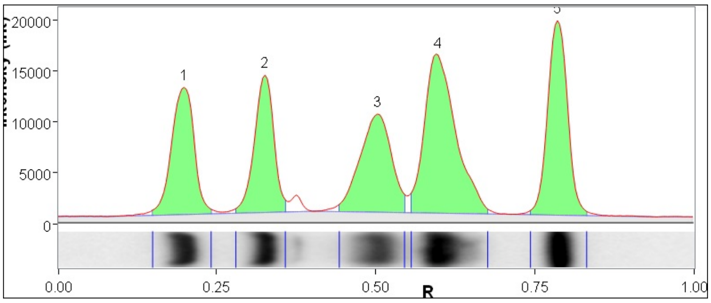

Band No.	Band Label	Mol. Wt. (KDa)	Relative Front	Adj. Volume (Int)	Volume (Int)	Abs. Quant.	Rel. Quant.	Band %	Lane %
1		N/A	0,201	5 560 191	6 372 081	N/A	N/A	16,5	16,0
2		N/A	0,329	5 163 050	6 029 345	N/A	N/A	15,3	14,9
3		N/A	0,505	5 972 708	7 156 133	N/A	N/A	17,7	17,2
4		N/A	0,601	9 645 340	10 863 795	N/A	N/A	28,5	27,8
5		N/A	0,788	7 458 414	8 188 185	N/A	N/A	22,1	21,5

Band Detection	Automatically detected bands with sensitivity: Low
Lane Background	Lane background subtracted with disk size: 23.2
Lane Width	4.72 mm

Lane 7

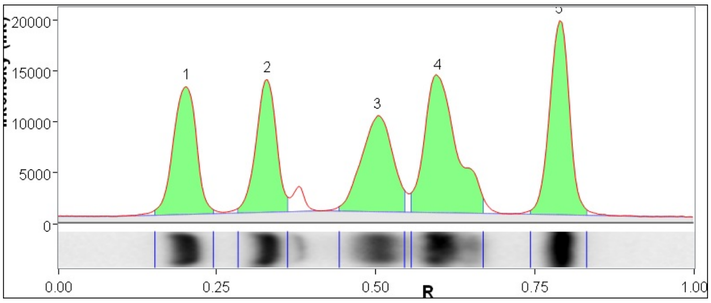

Band No.	Band Label	Mol. Wt. (KDa)	Relative Front	Adj. Volume (Int)	Volume (Int)	Abs. Quant.	Rel. Quant.	Band %	Lane %
1		N/A	0,205	5 833 920	6 673 728	N/A	N/A	17,4	16,8
2		N/A	0,332	5 324 608	6 225 344	N/A	N/A	15,9	15,4
3		N/A	0,505	5 924 672	7 187 200	N/A	N/A	17,7	17,1
4		N/A	0,601	8 858 816	10 132 000	N/A	N/A	26,4	25,5
5		N/A	0,792	7 563 200	8 348 064	N/A	N/A	22,6	21,8

Band Detection	Automatically detected bands with sensitivity: Low
Lane Background	Lane background subtracted with disk size: 23.2
Lane Width	4.87 mm

Lane 8

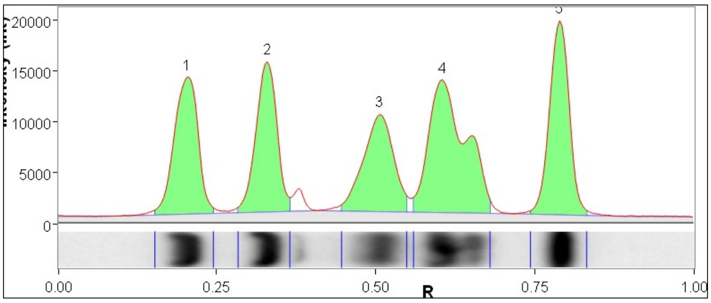

Band No.	Band Label	Mol. Wt. (KDa)	Relative Front	Adj. Volume (Int)	Volume (Int)	Abs. Quant.	Rel. Quant.	Band %	Lane %
1		N/A	0,205	6 143 146	6 972 086	N/A	N/A	18,1	17,5
2		N/A	0,332	5 930 641	6 854 906	N/A	N/A	17,4	16,9
3		N/A	0,509	5 504 453	6 717 266	N/A	N/A	16,2	15,7
4		N/A	0,608	9 292 467	10 587 058	N/A	N/A	27,3	26,5
5		N/A	0,792	7 153 622	7 896 971	N/A	N/A	21,0	20,4

Band Detection	Automatically detected bands with sensitivity: Low
Lane Background	Lane background subtracted with disk size: 23.2
Lane Width	4.72 mm

Lane 9

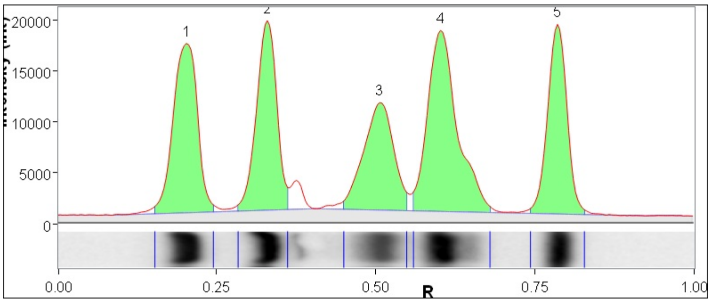

Band No.	Band Label	Mol. Wt. (KDa)	Relative Front	Adj. Volume (Int)	Volume (Int)	Abs. Quant.	Rel. Quant.	Band %	Lane %
1		N/A	0,205	6 818 912	7 668 576	N/A	N/A	20,4	19,7
2		N/A	0,332	6 374 368	7 282 784	N/A	N/A	19,0	18,4
3		N/A	0,509	5 358 432	6 534 368	N/A	N/A	16,0	15,5
4		N/A	0,604	8 876 320	10 109 504	N/A	N/A	26,5	25,6
5		N/A	0,788	6 042 752	6 739 936	N/A	N/A	18,1	17,4

Band Detection	Automatically detected bands with sensitivity: Low
Lane Background	Lane background subtracted with disk size: 23.2
Lane Width	4.87 mm

Lane 10

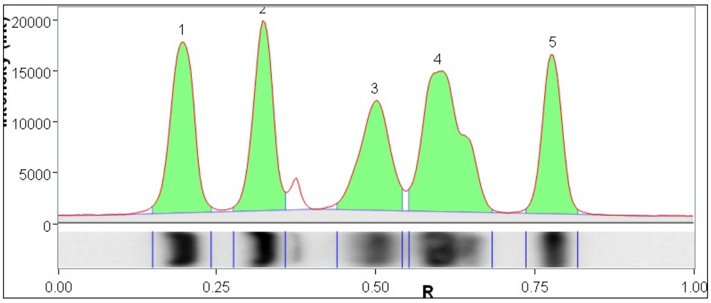

Band No.	Band Label	Mol. Wt. (KDa)	Relative Front	Adj. Volume (Int)	Volume (Int)	Abs. Quant.	Rel. Quant.	Band %	Lane %
1		N/A	0,198	6 358 620	7 158 420	N/A	N/A	20,2	19,6
2		N/A	0,325	5 955 210	6 825 120	N/A	N/A	18,9	18,3
3		N/A	0,502	5 241 810	6 378 720	N/A	N/A	16,7	16,1
4		N/A	0,601	8 921 040	10 175 430	N/A	N/A	28,4	27,5
5		N/A	0,781	4 974 510	5 621 790	N/A	N/A	15,8	15,3

Band Detection	Automatically detected bands with sensitivity: Low
Lane Background	Lane background subtracted with disk size: 23.2
Lane Width	4.57 mm

Lane 11

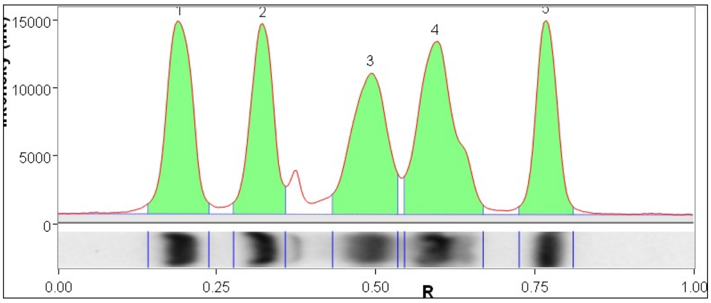

Band No.	Band Label	Mol. Wt. (KDa)	Relative Front	Adj. Volume (Int)	Volume (Int)	Abs. Quant.	Rel. Quant.	Band %	Lane %
1		N/A	0,194	7 158 063	7 830 372	N/A	N/A	20,1	18,9
2		N/A	0,325	6 284 190	6 851 163	N/A	N/A	17,6	16,6
3		N/A	0,495	7 176 609	7 870 236	N/A	N/A	20,1	19,0
4		N/A	0,597	9 168 060	9 986 922	N/A	N/A	25,7	24,2
5		N/A	0,770	5 903 931	6 460 377	N/A	N/A	16,5	15,6

Band Detection	Automatically detected bands with sensitivity: Low
Lane Background	Lane background subtracted with disk size: 23.2
Lane Width	5.03 mm

Lane 12

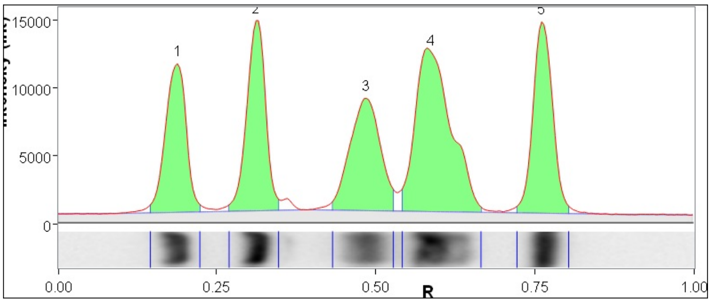

Band No.	Band Label	Mol. Wt. (KDa)	Relative Front	Adj. Volume (Int)	Volume (Int)	Abs. Quant.	Rel. Quant.	Band %	Lane %
1		N/A	0,191	4 202 143	4 822 608	N/A	N/A	15,7	15,3
2		N/A	0,314	4 866 349	5 580 186	N/A	N/A	18,2	17,7
3		N/A	0,488	4 637 414	5 525 192	N/A	N/A	17,3	16,9
4		N/A	0,590	7 958 475	8 991 116	N/A	N/A	29,8	28,9
5		N/A	0,763	5 085 023	5 680 223	N/A	N/A	19,0	18,5

Band Detection	Automatically detected bands with sensitivity: Low
Lane Background	Lane background subtracted with disk size: 23.2
Lane Width	4.72 mm

Lane 13

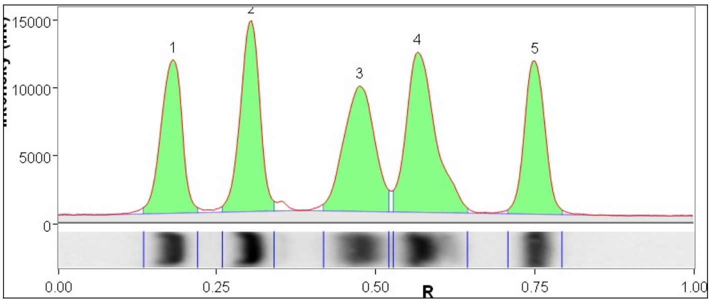

Band No.	Band Label	Mol. Wt. (KDa)	Relative Front	Adj. Volume (Int)	Volume (Int)	Abs. Quant.	Rel. Quant.	Band %	Lane %
1		N/A	0,184	4 980 894	5 673 155	N/A	N/A	17,3	17,0
2		N/A	0,307	5 712 029	6 502 591	N/A	N/A	19,9	19,5
3		N/A	0,477	5 951 163	6 959 779	N/A	N/A	20,7	20,3
4		N/A	0,569	7 243 150	8 265 561	N/A	N/A	25,2	24,7
5		N/A	0,753	4 835 163	5 461 890	N/A	N/A	16,8	16,5

Band Detection	Automatically detected bands with sensitivity: Low
Lane Background	Lane background subtracted with disk size: 23.2
Lane Width	4.72 mm

Lane 14

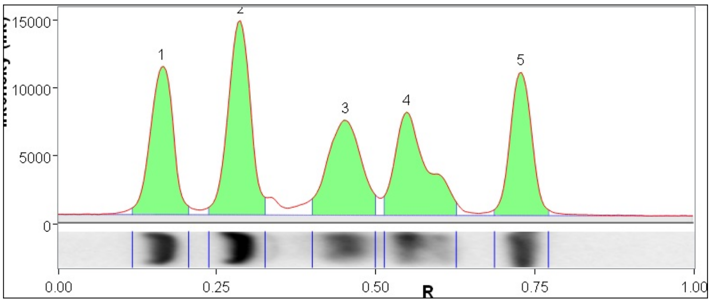

Band No.	Band Label	Mol. Wt. (KDa)	Relative Front	Adj. Volume (Int)	Volume (Int)	Abs. Quant.	Rel. Quant.	Band %	Lane %
1		N/A	0,166	5 859 840	6 552 385	N/A	N/A	19,5	18,5
2		N/A	0,290	7 502 635	8 178 100	N/A	N/A	24,9	23,7
3		N/A	0,456	5 514 635	6 241 270	N/A	N/A	18,3	17,4
4		N/A	0,551	5 887 980	6 717 200	N/A	N/A	19,6	18,6
5		N/A	0,731	5 335 400	5 923 505	N/A	N/A	17,7	16,9

Band Detection	Automatically detected bands with sensitivity: Low
Lane Background	Lane background subtracted with disk size: 23.2
Lane Width	5.33 mm

Image Report: Intensity analysis of Supplementary Fig.1e (Oxphos_ Total oxphos panel, females)

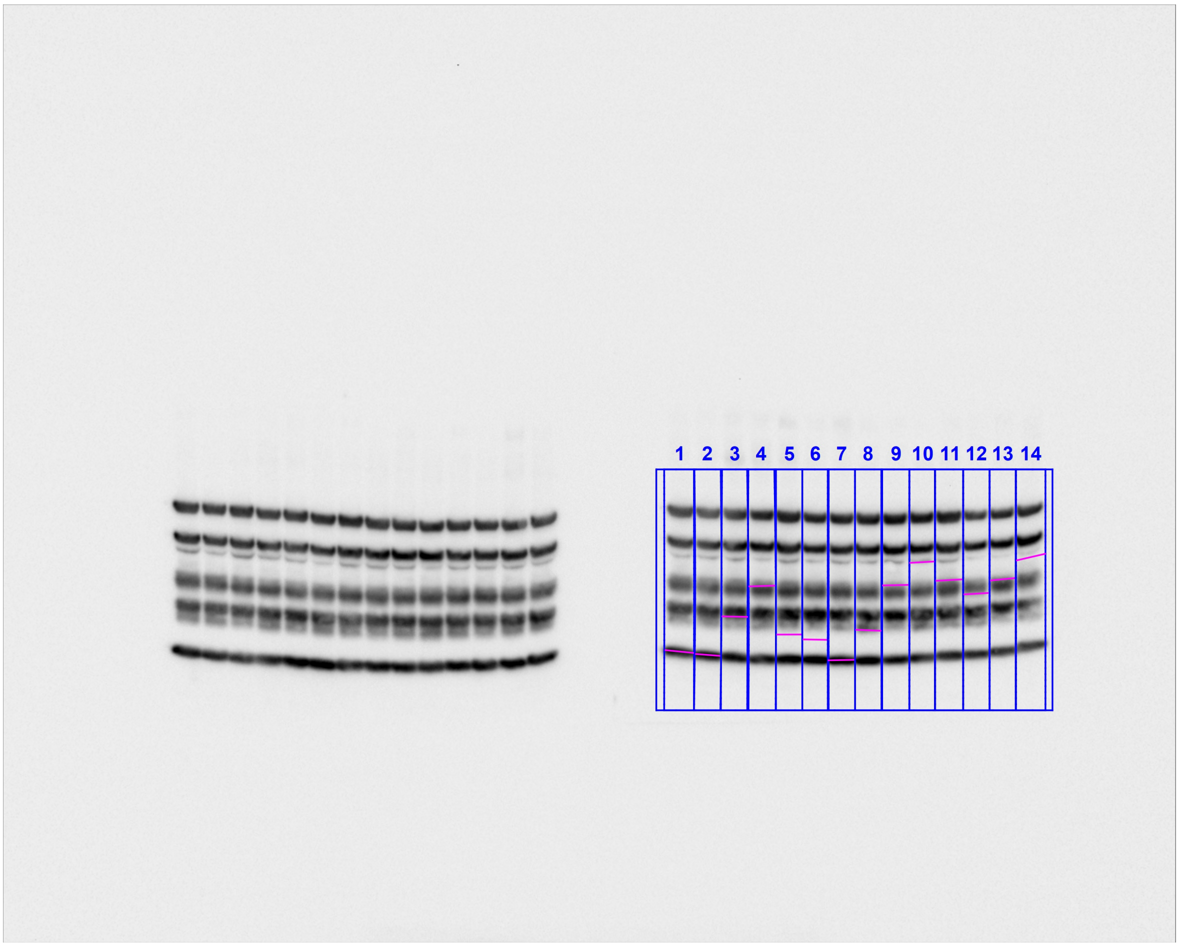

Acquisition Information

Imager	ChemiDoc Touch
Exposure Time (sec)	3.000 (Signal Accumulation)
Serial Number	732BR1240
Software Version	1.1.0.04
Application	Chemiluminescence
Excitation Source	No Illumination
Emission Filter	No Filter
Binning	2x2

Image Information

Acquisition Date	2/7/2021 3:25:24
User Name	
Image Area (mm)	X: 210.0 Y: 168.1
Pixel Size (µm)	X: 152.3 Y: 152.3

Data Range (Int)	500 - 26965

Analysis Settings

Detection	Lane detection:<br>Manually created lanes<br><br>Band detection:<br>Bands detected with different sensitivity per lane<br>Manually adjusted bands<br><br>Lane Background Subtraction:<br>Lane background subtracted with disk size: 30.1<br><br>Lane width: Variable

Lane Statistics

Lane No.	Adj. Total Band Vol. (Int)	Total Band Vol. (Int)	Adj. Total Lane Vol. (Int)	Total Lane Vol. (Int)	Bkgd. Vol. (Int)	Norm. Factor
1	32 612 265	38 060 155	32 724 825	39 628 435	6 903 610	N/A
2	30 916 641	35 781 409	31 007 998	37 152 477	6 144 479	N/A
3	34 512 052	39 574 197	34 622 598	41 042 946	6 420 348	N/A
4	34 061 984	41 603 808	34 162 112	43 139 360	8 977 248	N/A
5	40 290 824	45 535 373	40 386 707	47 041 880	6 655 173	N/A
6	37 191 661	42 187 807	37 276 508	43 587 488	6 310 980	N/A
7	37 385 760	42 563 872	37 470 976	43 980 832	6 509 856	N/A
8	37 680 469	42 687 558	37 766 680	44 035 593	6 268 913	N/A
9	37 177 504	42 350 816	37 247 360	43 697 440	6 450 080	N/A
10	34 944 630	39 624 300	35 042 400	40 954 050	5 911 650	N/A
11	37 750 152	42 918 480	37 868 292	44 393 580	6 525 288	N/A
12	27 398 420	33 795 208	27 438 100	35 122 783	7 684 683	N/A
13	29 278 663	35 818 423	29 341 593	37 159 607	7 818 014	N/A
14	31 629 500	37 134 615	31 713 290	38 651 095	6 937 805	N/A

Lane And Band Analysis

Lane 1

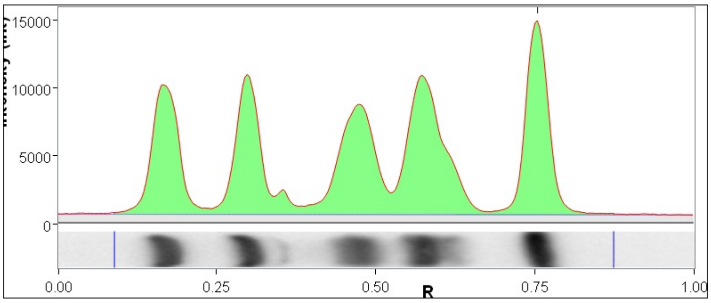

Band No.	Band Label	Mol. Wt. (KDa)	Relative Front	Adj. Volume (Int)	Volume (Int)	Abs. Quant.	Rel. Quant.	Band %	Lane %
1		N/A	0,756	32 612 265	38 060 155	N/A	N/A	100,0	99,7

Band Detection	Automatically detected bands with sensitivity: Low
Lane Background	Lane background subtracted with disk size: 30.1
Lane Width	5.33 mm

Lane 2

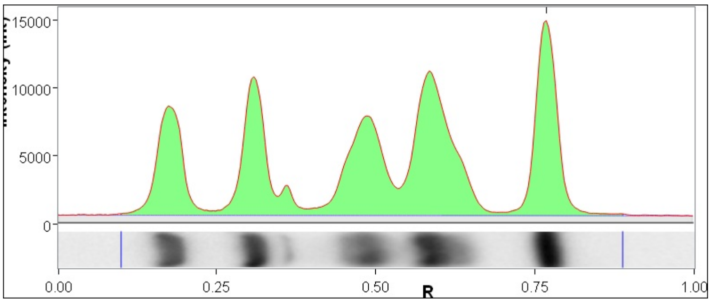

Band No.	Band Label	Mol. Wt. (KDa)	Relative Front	Adj. Volume (Int)	Volume (Int)	Abs. Quant.	Rel. Quant.	Band %	Lane %
1		N/A	0,770	30 916 641	35 781 409	N/A	N/A	100,0	99,7

Band Detection	Automatically detected bands with sensitivity: Low
Lane Background	Lane background subtracted with disk size: 30.1
Lane Width	4.72 mm

Lane 3

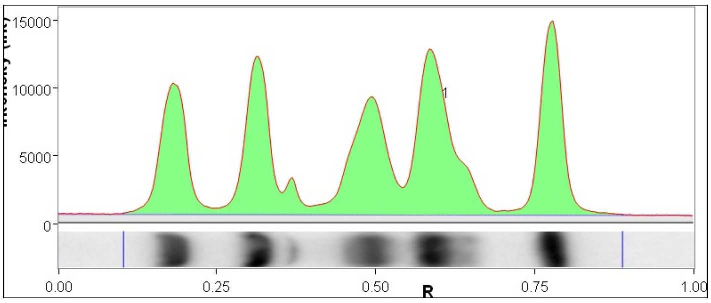

Band No.	Band Label	Mol. Wt. (KDa)	Relative Front	Adj. Volume (Int)	Volume (Int)	Abs. Quant.	Rel. Quant.	Band %	Lane %
1		N/A	0,611	34 512 052	39 574 197	N/A	N/A	100,0	99,7

Band Detection	Automatically detected bands with sensitivity: Low
Lane Background	Lane background subtracted with disk size: 30.1
Lane Width	4.72 mm

Lane 4

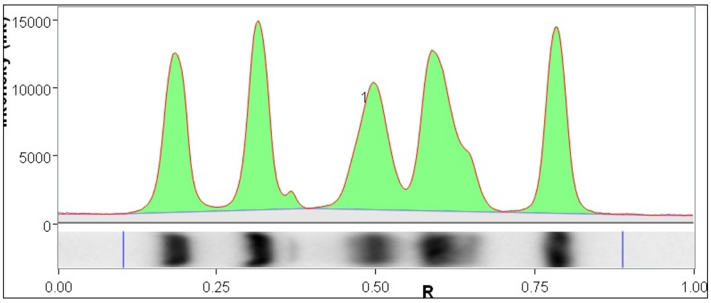

Band No.	Band Label	Mol. Wt. (KDa)	Relative Front	Adj. Volume (Int)	Volume (Int)	Abs. Quant.	Rel. Quant.	Band %	Lane %
1		N/A	0,484	34 061 984	41 603 808	N/A	N/A	100,0	99,7

Lane Background	Lane background subtracted with disk size: 30.1

Lane Width	4.87 mm

Lane 5

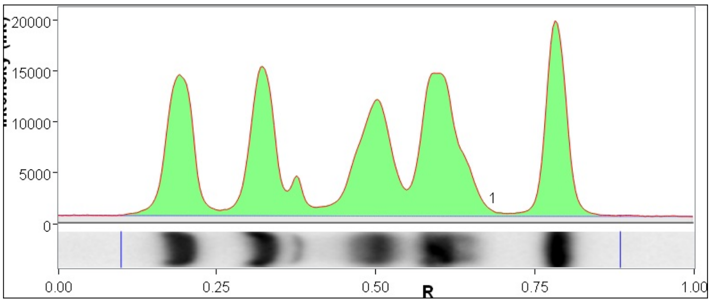

Band No.	Band Label	Mol. Wt. (KDa)	Relative Front	Adj. Volume (Int)	Volume (Int)	Abs. Quant.	Rel. Quant.	Band %	Lane %
1		N/A	0,686	40 290 824	45 535 373	N/A	N/A	100,0	99,8

Band Detection	Automatically detected bands with sensitivity: Low
Lane Background	Lane background subtracted with disk size: 30.1
Lane Width	4.72 mm

Lane 6

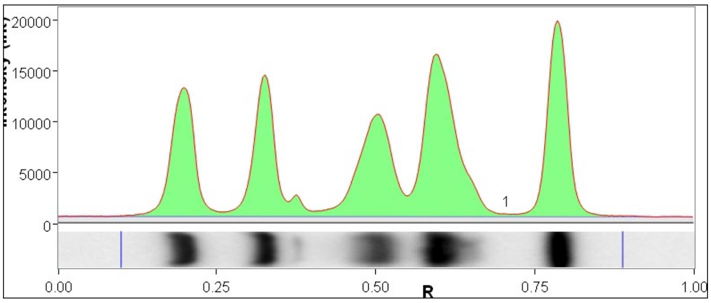

Band No.	Band Label	Mol. Wt. (KDa)	Relative Front	Adj. Volume (Int)	Volume (Int)	Abs. Quant.	Rel. Quant.	Band %	Lane %
1		N/A	0,707	37 191 661	42 187 807	N/A	N/A	100,0	99,8

Band Detection	Automatically detected bands with sensitivity: Low
Lane Background	Lane background subtracted with disk size: 30.1
Lane Width	4.72 mm

Lane 7

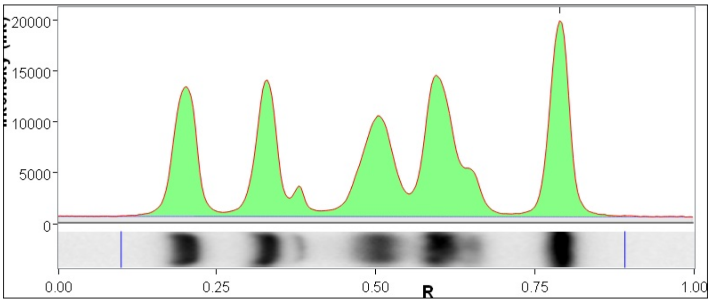

Band No.	Band Label	Mol. Wt. (KDa)	Relative Front	Adj. Volume (Int)	Volume (Int)	Abs. Quant.	Rel. Quant.	Band %	Lane %
1		N/A	0,792	37 385 760	42 563 872	N/A	N/A	100,0	99,8

Band Detection	Automatically detected bands with sensitivity: Low
Lane Background	Lane background subtracted with disk size: 30.1
Lane Width	4.87 mm

Lane 8

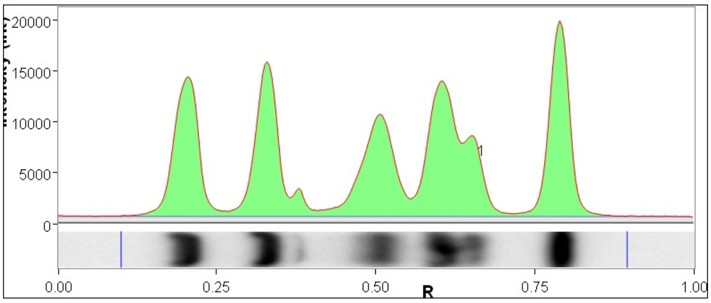

Band No.	Band Label	Mol. Wt. (KDa)	Relative Front	Adj. Volume (Int)	Volume (Int)	Abs. Quant.	Rel. Quant.	Band %	Lane %
1		N/A	0,668	37 680 469	42 687 558	N/A	N/A	100,0	99,8

Band Detection	Automatically detected bands with sensitivity: Low
Lane Background	Lane background subtracted with disk size: 30.1
Lane Width	4.72 mm

Lane 9

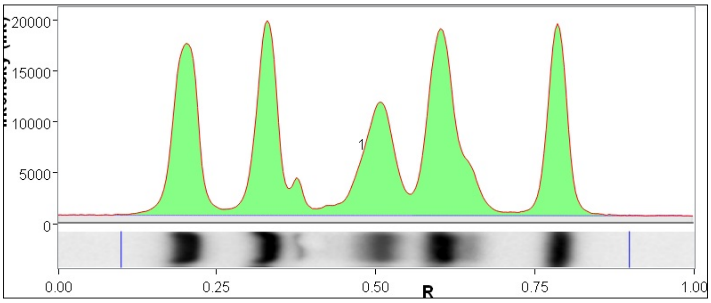

Band No.	Band Label	Mol. Wt. (KDa)	Relative Front	Adj. Volume (Int)	Volume (Int)	Abs. Quant.	Rel. Quant.	Band %	Lane %
1		N/A	0,481	37 177 504	42 350 816	N/A	N/A	100,0	99,8

Band Detection	Automatically detected bands with sensitivity: Low
Lane Background	Lane background subtracted with disk size: 30.1
Lane Width	4.87 mm

Lane 10

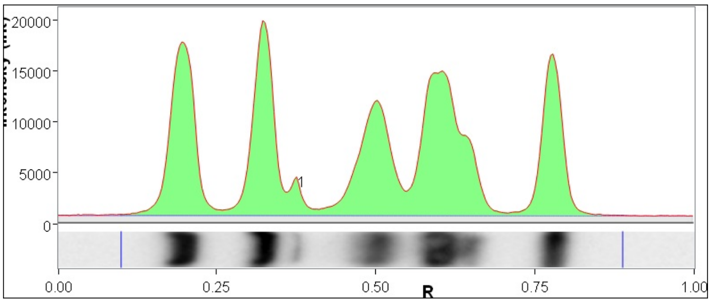

Band No.	Band Label	Mol. Wt. (KDa)	Relative Front	Adj. Volume (Int)	Volume (Int)	Abs. Quant.	Rel. Quant.	Band %	Lane %
1		N/A	0,385	34 944 630	39 624 300	N/A	N/A	100,0	99,7

Band Detection	Automatically detected bands with sensitivity: Low
Lane Background	Lane background subtracted with disk size: 30.1
Lane Width	4.57 mm

Lane 11

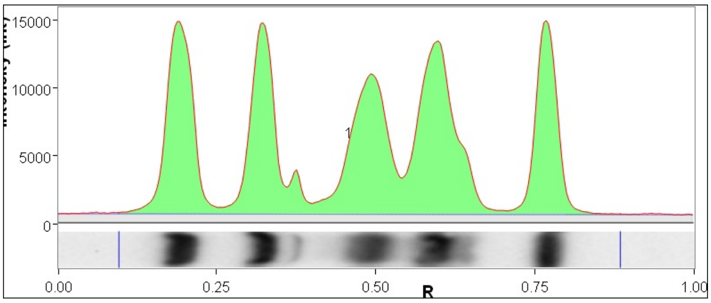

Band No.	Band Label	Mol. Wt. (KDa)	Relative Front	Adj. Volume (Int)	Volume (Int)	Abs. Quant.	Rel. Quant.	Band %	Lane %
1		N/A	0,459	37 750 152	42 918 480	N/A	N/A	100,0	99,7

Band Detection	Automatically detected bands with sensitivity: Low
Lane Background	Lane background subtracted with disk size: 30.1
Lane Width	5.03 mm

Lane 12

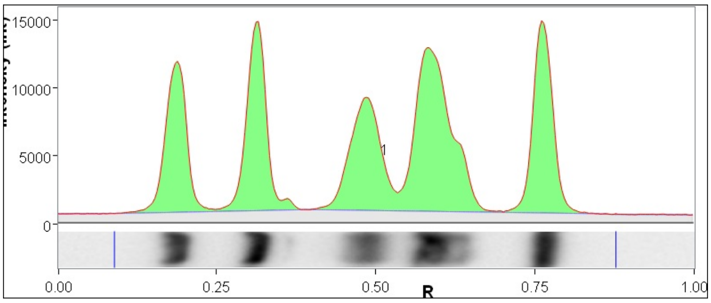

Band No.	Band Label	Mol. Wt. (KDa)	Relative Front	Adj. Volume (Int)	Volume (Int)	Abs. Quant.	Rel. Quant.	Band %	Lane %
1		N/A	0,516	27 398 420	33 795 208	N/A	N/A	100,0	99,9

Band Detection	Automatically detected bands with sensitivity: Low

Lane Background	Lane background subtracted with disk size: 30.1
Lane Width	4.72 mm

Lane 13

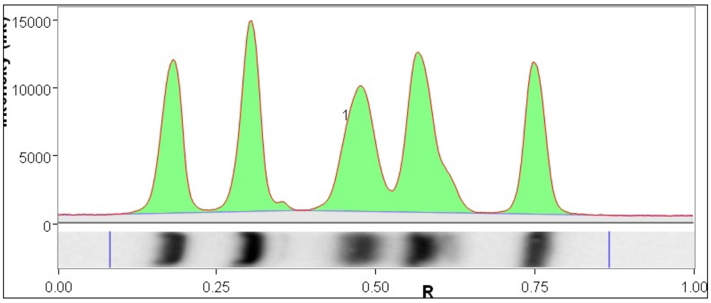

Band No.	Band Label	Mol. Wt. (KDa)	Relative Front	Adj. Volume (Int)	Volume (Int)	Abs. Quant.	Rel. Quant.	Band %	Lane %
1		N/A	0,456	29 278 663	35 818 423	N/A	N/A	100,0	99,8

Band Detection	Automatically detected bands with sensitivity: Low
Lane Background	Lane background subtracted with disk size: 30.1
Lane Width	4.72 mm

Lane 14

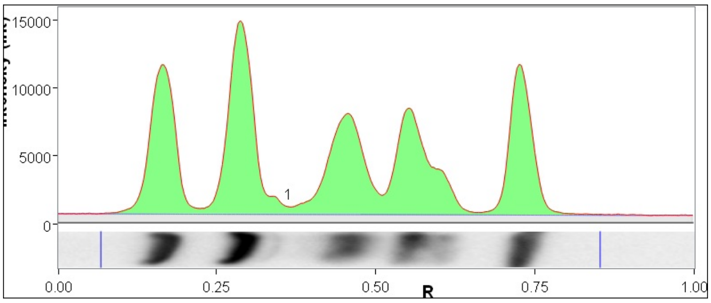

Band No.	Band Label	Mol. Wt. (KDa)	Relative Front	Adj. Volume (Int)	Volume (Int)	Abs. Quant.	Rel. Quant.	Band %	Lane %
1		N/A	0,364	31 629 500	37 134 615	N/A	N/A	100,0	99,7

Band Detection	Automatically detected bands with sensitivity: Low
Lane Background	Lane background subtracted with disk size: 30.1
Lane Width	5.33 mm

Image Report: Intensity analysis of Supplementary Fig.1e (Gapdh, males)

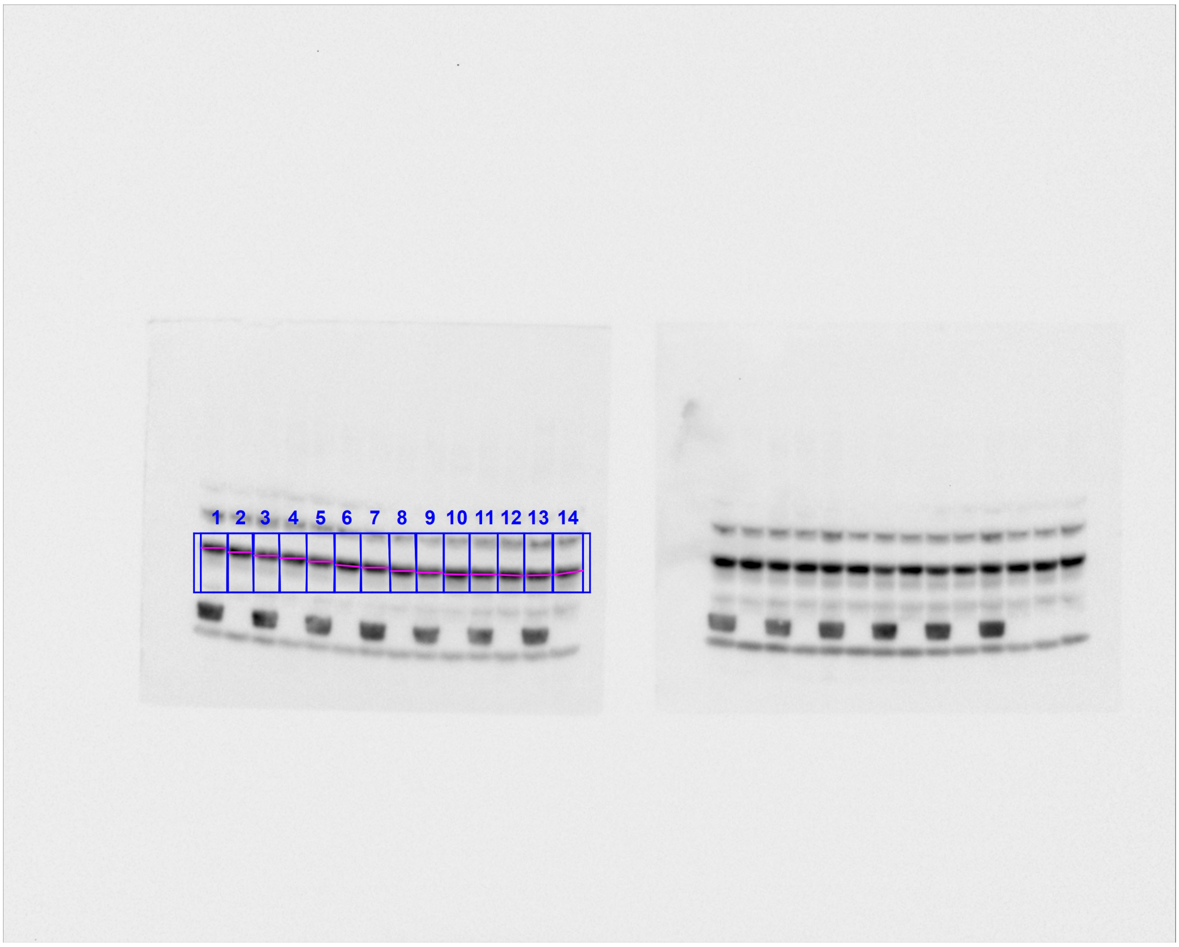

Acquisition Information

Imager	ChemiDoc Touch
Exposure Time (sec)	4.000 (Signal Accumulation)
Serial Number	732BR1240
Software Version	1.1.0.04
Application	Chemiluminescence
Excitation Source	No Illumination
Emission Filter	No Filter
Binning	2x2

Image Information

Acquisition Date	2/15/2021 7:12:59
User Name	
Image Area (mm)	X: 210.0 Y: 168.1
Pixel Size (µm)	X: 152.3 Y: 152.3

Data Range (Int)	500 - 30519

Analysis Settings

Detection	Lane detection:<br>Manually created lanes (Copied)<br><br>Band detection:<br>Automatically detected bands with sensitivity: Low<br>Manually adjusted bands<br><br>Lane Background Subtraction:<br>Lane background subtracted with disk size: 30.1<br><br>Lane width: Variable

Lane Statistics

Lane No.	Adj. Total Band Vol. (Int)	Total Band Vol. (Int)	Adj. Total Lane Vol. (Int)	Total Lane Vol. (Int)	Bkgd. Vol. (Int)	Norm. Factor
1	5 471 712	6 647 712	6 157 344	8 862 144	2 704 800	N/A
2	5 772 727	6 849 915	6 349 079	9 003 578	2 654 499	N/A
3	4 456 901	5 553 061	5 056 007	7 965 047	2 909 040	N/A
4	5 744 864	6 828 128	6 330 112	8 999 584	2 669 472	N/A
5	5 008 641	6 074 277	5 705 865	8 533 899	2 828 034	N/A
6	6 617 756	7 665 432	7 542 579	10 124 352	2 581 773	N/A
7	5 664 944	6 947 390	6 952 864	10 230 226	3 277 362	N/A
8	5 474 383	6 711 283	6 724 086	9 772 161	3 048 075	N/A
9	4 603 200	5 742 208	5 981 536	9 004 288	3 022 752	N/A
10	6 246 990	7 470 870	7 912 650	10 928 640	3 015 990	N/A
11	5 496 513	6 846 378	7 461 828	10 911 483	3 449 655	N/A
12	5 712 153	7 046 331	7 745 381	11 154 947	3 409 566	N/A
13	5 555 880	6 891 555	7 814 202	11 500 665	3 686 463	N/A
14	5 000 170	6 562 290	6 873 125	10 722 635	3 849 510	N/A

Lane And Band Analysis

Lane 1

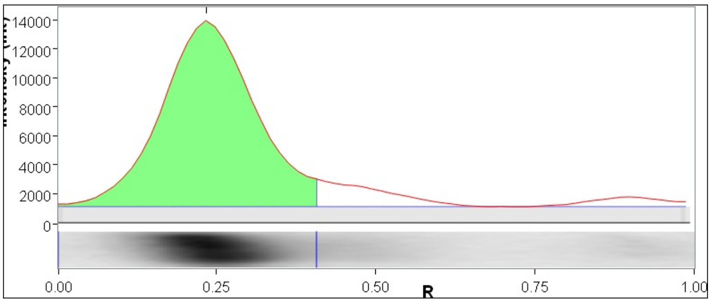

Band No.	Band Label	Mol. Wt. (KDa)	Relative Front	Adj. Volume (Int)	Volume (Int)	Abs. Quant.	Rel. Quant.	Band %	Lane %
1		N/A	0,246	5 471 712	6 647 712	N/A	N/A	100,0	88,9

Band Detection	Automatically detected bands with sensitivity: Low
Lane Background	Lane background subtracted with disk size: 30.1
Lane Width	4.87 mm

Lane 2

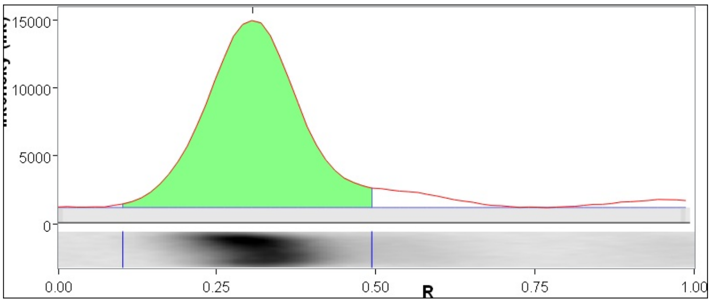

Band No.	Band Label	Mol. Wt. (KDa)	Relative Front	Adj. Volume (Int)	Volume (Int)	Abs. Quant.	Rel. Quant.	Band %	Lane %
1		N/A	0,319	5 772 727	6 849 915	N/A	N/A	100,0	90,9

Band Detection	Automatically detected bands with sensitivity: Low
Lane Background	Lane background subtracted with disk size: 30.1
Lane Width	4.72 mm

Lane 3

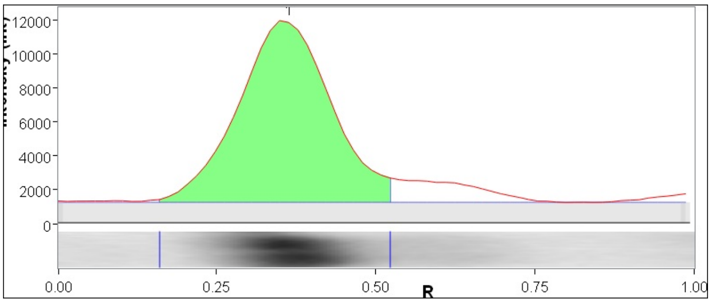

Band No.	Band Label	Mol. Wt. (KDa)	Relative Front	Adj. Volume (Int)	Volume (Int)	Abs. Quant.	Rel. Quant.	Band %	Lane %
1		N/A	0,377	4 456 901	5 553 061	N/A	N/A	100,0	88,2

Band Detection	Automatically detected bands with sensitivity: Low
Lane Background	Lane background subtracted with disk size: 30.1
Lane Width	4.72 mm

Lane 4

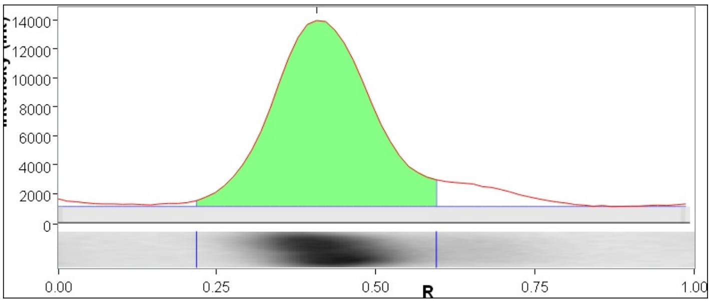

Band No.	Band Label	Mol. Wt. (KDa)	Relative Front	Adj. Volume (Int)	Volume (Int)	Abs. Quant.	Rel. Quant.	Band %	Lane %
1		N/A	0,420	5 744 864	6 828 128	N/A	N/A	100,0	90,8

Band Detection	Automatically detected bands with sensitivity: Low

Lane Background	Lane background subtracted with disk size: 30.1
Lane Width	4.87 mm

Lane 5

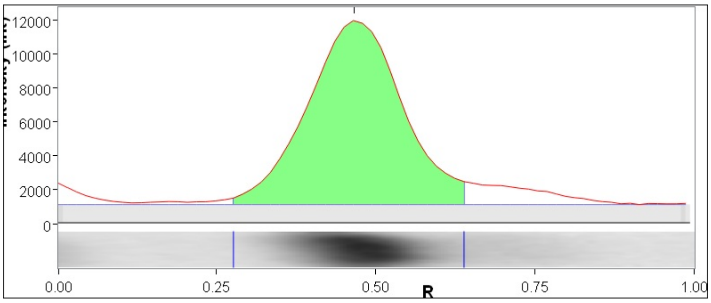

Band No.	Band Label	Mol. Wt. (KDa)	Relative Front	Adj. Volume (Int)	Volume (Int)	Abs. Quant.	Rel. Quant.	Band %	Lane %
1		N/A	0,478	5 008 641	6 074 277	N/A	N/A	100,0	87,8

Band Detection	Automatically detected bands with sensitivity: Low
Lane Background	Lane background subtracted with disk size: 30.1
Lane Width	5.03 mm

Lane 6

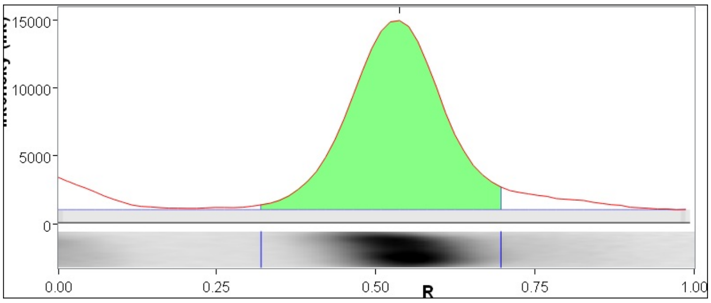

Band No.	Band Label	Mol. Wt. (KDa)	Relative Front	Adj. Volume (Int)	Volume (Int)	Abs. Quant.	Rel. Quant.	Band %	Lane %
1		N/A	0,551	6 617 756	7 665 432	N/A	N/A	100,0	87,7

Band Detection	Automatically detected bands with sensitivity: Low
Lane Background	Lane background subtracted with disk size: 30.1
Lane Width	4.72 mm

Lane 7

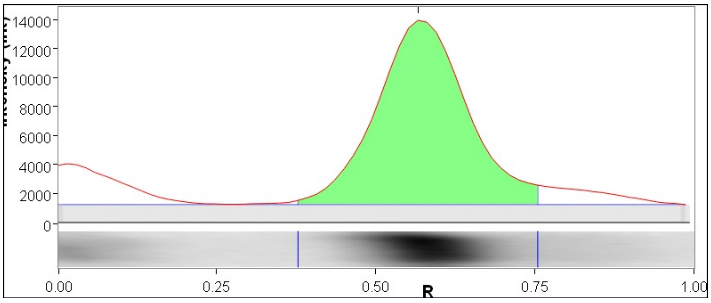

Band No.	Band Label	Mol. Wt. (KDa)	Relative Front	Adj. Volume (Int)	Volume (Int)	Abs. Quant.	Rel. Quant.	Band %	Lane %
1		N/A	0,580	5 664 944	6 947 390	N/A	N/A	100,0	81,5

Band Detection	Automatically detected bands with sensitivity: Low
Lane Background	Lane background subtracted with disk size: 30.1
Lane Width	5.18 mm

Lane 8

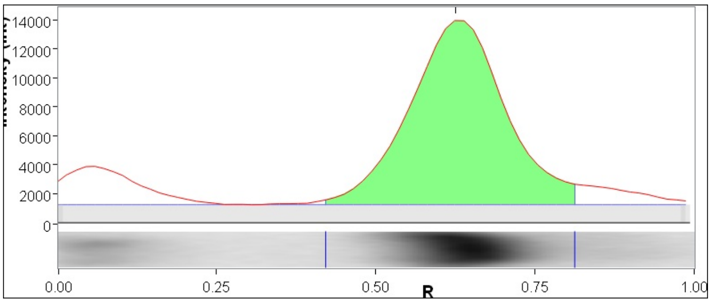

Band No.	Band Label	Mol. Wt. (KDa)	Relative Front	Adj. Volume (Int)	Volume (Int)	Abs. Quant.	Rel. Quant.	Band %	Lane %
1		N/A	0,638	5 474 383	6 711 283	N/A	N/A	100,0	81,4

Band Detection	Automatically detected bands with sensitivity: Low
Lane Background	Lane background subtracted with disk size: 30.1
Lane Width	4.72 mm

Lane 9

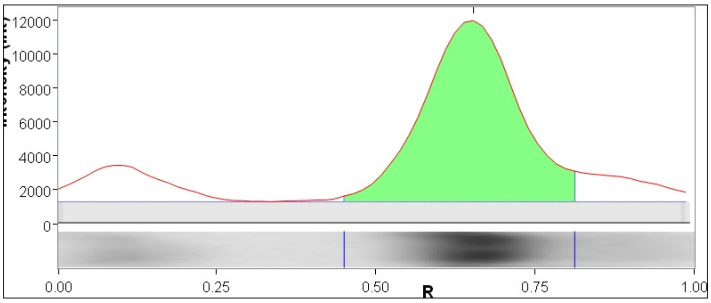

Band No.	Band Label	Mol. Wt. (KDa)	Relative Front	Adj. Volume (Int)	Volume (Int)	Abs. Quant.	Rel. Quant.	Band %	Lane %
1		N/A	0,667	4 603 200	5 742 208	N/A	N/A	100,0	77,0

Band Detection	Automatically detected bands with sensitivity: Low
Lane Background	Lane background subtracted with disk size: 30.1
Lane Width	4.87 mm

Lane 10

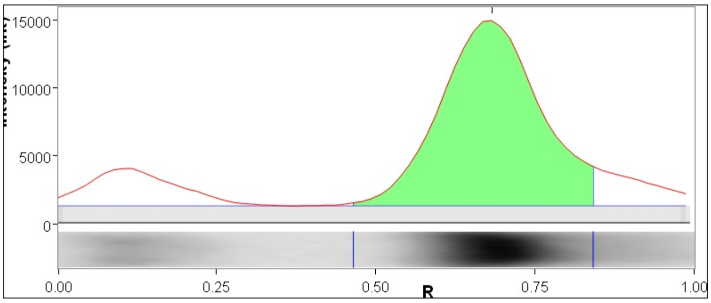

Band No.	Band Label	Mol. Wt. (KDa)	Relative Front	Adj. Volume (Int)	Volume (Int)	Abs. Quant.	Rel. Quant.	Band %	Lane %
1		N/A	0,696	6 246 990	7 470 870	N/A	N/A	100,0	78,9

Band Detection	Automatically detected bands with sensitivity: Low
Lane Background	Lane background subtracted with disk size: 30.1
Lane Width	4.57 mm

Lane 11

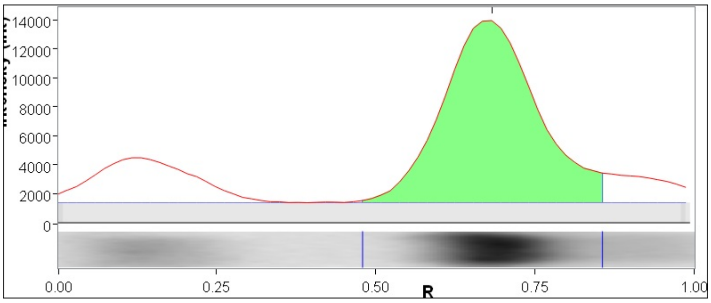

Band No.	Band Label	Mol. Wt. (KDa)	Relative Front	Adj. Volume (Int)	Volume (Int)	Abs. Quant.	Rel. Quant.	Band %	Lane %
1		N/A	0,696	5 496 513	6 846 378	N/A	N/A	100,0	73,7

Band Detection	Automatically detected bands with sensitivity: Low
Lane Background	Lane background subtracted with disk size: 30.1
Lane Width	5.03 mm

Lane 12

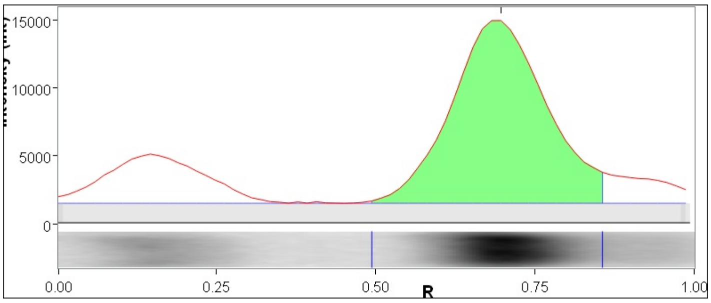

Band No.	Band Label	Mol. Wt. (KDa)	Relative Front	Adj. Volume (Int)	Volume (Int)	Abs. Quant.	Rel. Quant.	Band %	Lane %
1		N/A	0,710	5 712 153	7 046 331	N/A	N/A	100,0	73,7

Band Detection	Automatically detected bands with sensitivity: Low

Lane Background	Lane background subtracted with disk size: 30.1
Lane Width	4.72 mm

Lane 13

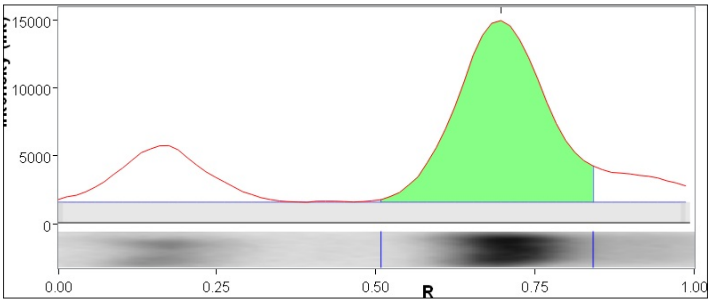

Band No.	Band Label	Mol. Wt. (KDa)	Relative Front	Adj. Volume (Int)	Volume (Int)	Abs. Quant.	Rel. Quant.	Band %	Lane %
1		N/A	0,710	5 555 880	6 891 555	N/A	N/A	100,0	71,1

Band Detection	Automatically detected bands with sensitivity: Low
Lane Background	Lane background subtracted with disk size: 30.1
Lane Width	5.03 mm

Lane 14

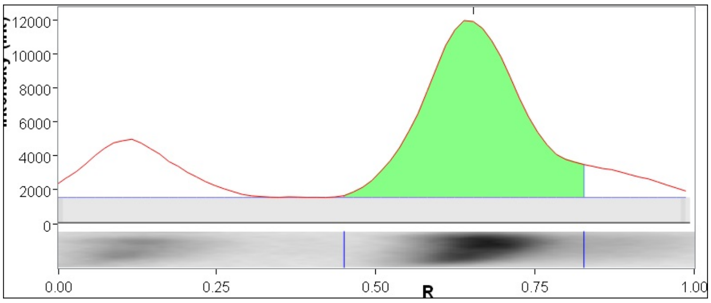

Band No.	Band Label	Mol. Wt. (KDa)	Relative Front	Adj. Volume (Int)	Volume (Int)	Abs. Quant.	Rel. Quant.	Band %	Lane %
1		N/A	0,667	5 000 170	6 562 290	N/A	N/A	100,0	72,7

Band Detection	Automatically detected bands with sensitivity: Low
Lane Background	Lane background subtracted with disk size: 30.1
Lane Width	5.33 mm

Image Report: Intensity analysis of Supplementary Fig. 1e (Gapdh, females)

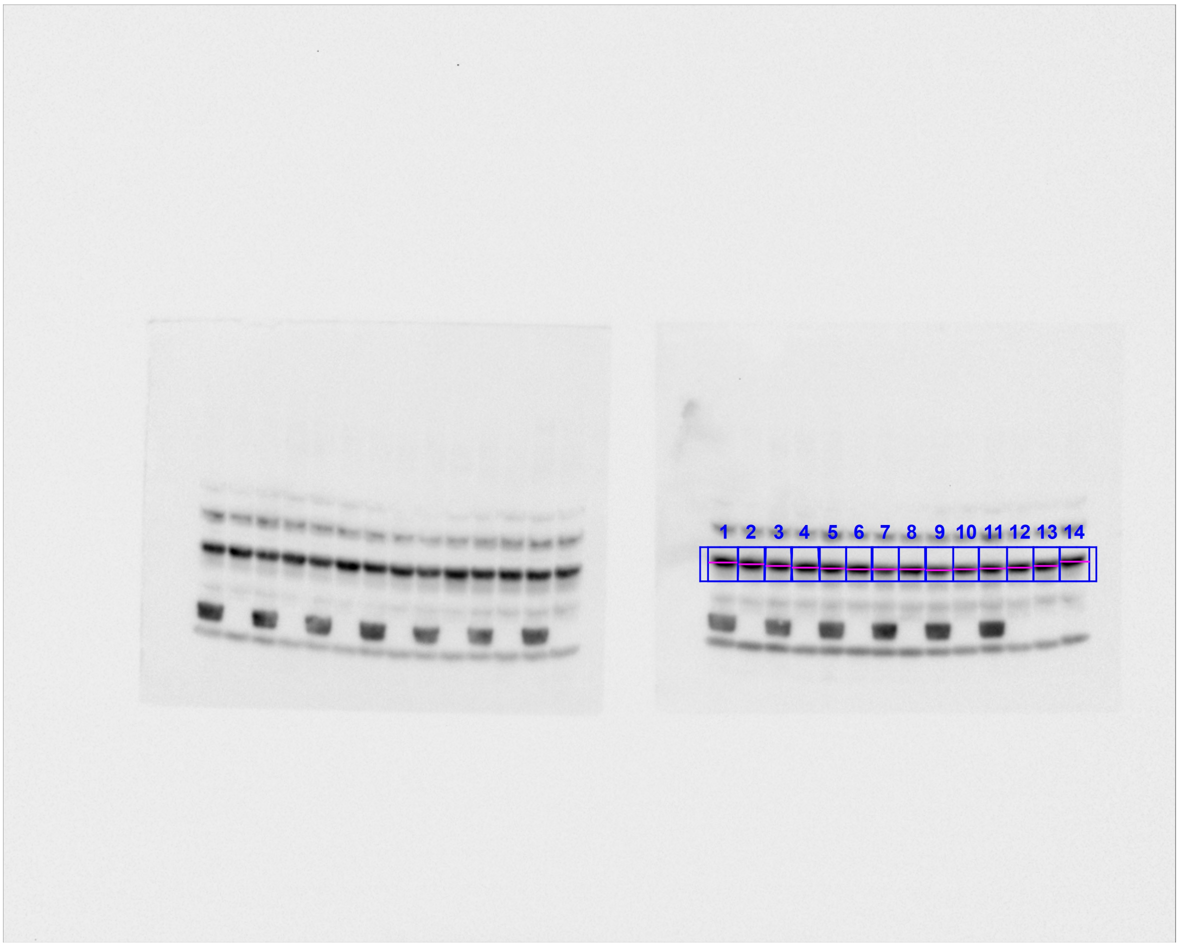

Acquisition Information

Imager	ChemiDoc Touch
Exposure Time (sec)	4.000 (Signal Accumulation)
Serial Number	732BR1240
Software Version	1.1.0.04
Application	Chemiluminescence
Excitation Source	No Illumination
Emission Filter	No Filter
Binning	2x2

Image Information

Acquisition Date	2/15/2021 7:12:59
User Name	
Image Area (mm)	X: 210.0 Y: 168.1
Pixel Size (µm)	X: 152.3 Y: 152.3

Data Range (Int)	500 - 30519

Analysis Settings

Detection	Lane detection:<br>Manually created lanes (Copied)<br><br>Band detection:<br>Automatically detected bands with sensitivity: Low<br><br>Lane Background Subtraction:<br>Lane background subtracted with disk size: 24.1<br><br>Lane width: Variable

Lane Statistics

Lane No.	Adj. Total Band Vol. (Int)	Total Band Vol. (Int)	Adj. Total Lane Vol. (Int)	Total Lane Vol. (Int)	Bkgd. Vol. (Int)	Norm. Factor
1	7 033 600	9 250 080	7 200 830	9 971 430	2 770 600	N/A
2	7 050 764	8 901 464	7 245 072	9 712 672	2 467 600	N/A
3	6 067 909	7 562 791	6 387 829	8 602 469	2 214 640	N/A
4	6 852 800	8 175 584	7 131 744	9 091 424	1 959 680	N/A
5	6 304 904	7 511 486	6 630 342	8 486 622	1 856 280	N/A
6	6 667 635	7 743 955	6 761 441	8 299 041	1 537 600	N/A
7	4 746 624	5 813 248	4 863 168	6 504 128	1 640 960	N/A
8	6 321 365	7 386 866	6 434 856	8 013 376	1 578 520	N/A
9	5 171 360	6 215 520	5 275 936	6 882 336	1 606 400	N/A
10	5 075 280	6 077 250	5 173 860	6 658 260	1 484 400	N/A
11	5 952 441	7 110 741	6 333 426	8 186 706	1 853 280	N/A
12	6 230 411	7 339 436	6 464 926	8 107 926	1 643 000	N/A
13	6 420 379	7 407 729	6 918 270	8 437 270	1 519 000	N/A
14	6 685 000	8 081 500	7 051 940	8 913 940	1 862 000	N/A

Lane And Band Analysis

Lane 1

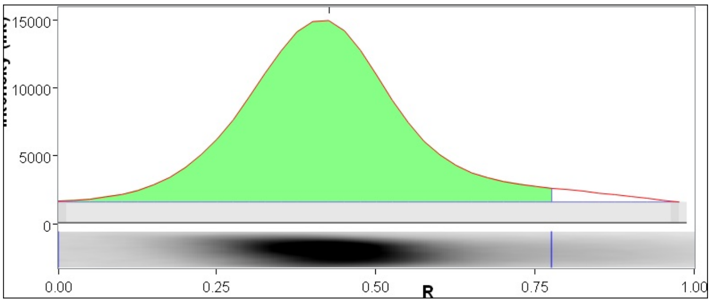

Band No.	Band Label	Mol. Wt. (KDa)	Relative Front	Adj. Volume (Int)	Volume (Int)	Abs. Quant.	Rel. Quant.	Band %	Lane %
1		N/A	0,450	7 033 600	9 250 080	N/A	N/A	100,0	97,7

Band Detection	Automatically detected bands with sensitivity: Low
Lane Background	Lane background subtracted with disk size: 24.1
Lane Width	5.33 mm

Lane 2

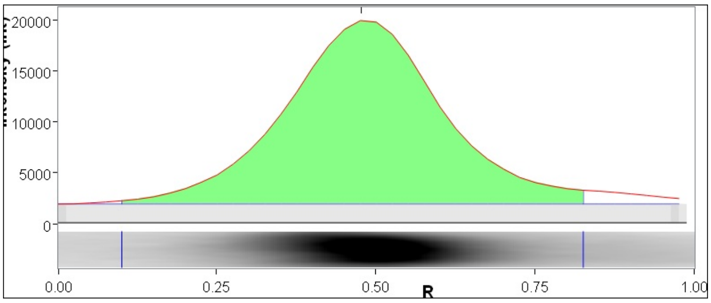

Band No.	Band Label	Mol. Wt. (KDa)	Relative Front	Adj. Volume (Int)	Volume (Int)	Abs. Quant.	Rel. Quant.	Band %	Lane %
1		N/A	0,500	7 050 764	8 901 464	N/A	N/A	100,0	97,3

Band Detection	Automatically detected bands with sensitivity: Low
Lane Background	Lane background subtracted with disk size: 24.1
Lane Width	4.72 mm

Lane 3

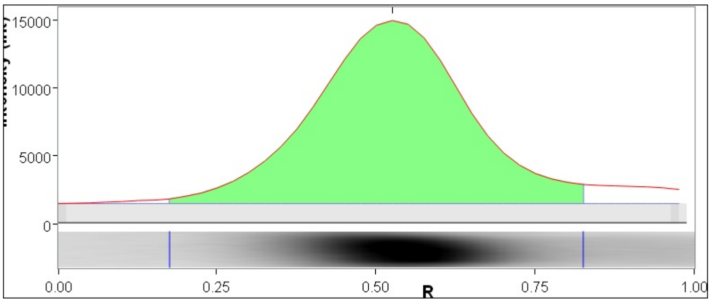

Band No.	Band Label	Mol. Wt. (KDa)	Relative Front	Adj. Volume (Int)	Volume (Int)	Abs. Quant.	Rel. Quant.	Band %	Lane %
1		N/A	0,550	6 067 909	7 562 791	N/A	N/A	100,0	95,0

Band Detection	Automatically detected bands with sensitivity: Low
Lane Background	Lane background subtracted with disk size: 24.1
Lane Width	4.72 mm

Lane 4

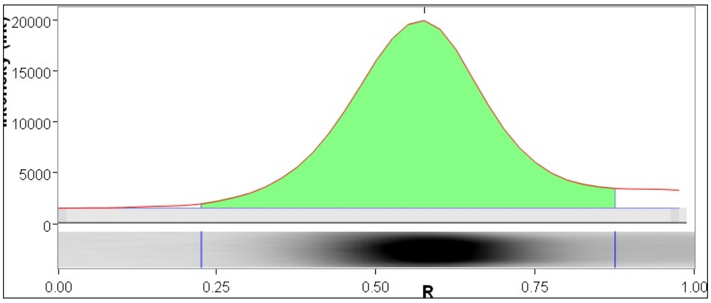

Band No.	Band Label	Mol. Wt. (KDa)	Relative Front	Adj. Volume (Int)	Volume (Int)	Abs. Quant.	Rel. Quant.	Band %	Lane %
1		N/A	0,600	6 852 800	8 175 584	N/A	N/A	100,0	96,1

Band Detection	Automatically detected bands with sensitivity: Low

Lane Background	Lane background subtracted with disk size: 24.1
Lane Width	4.87 mm

Lane 5

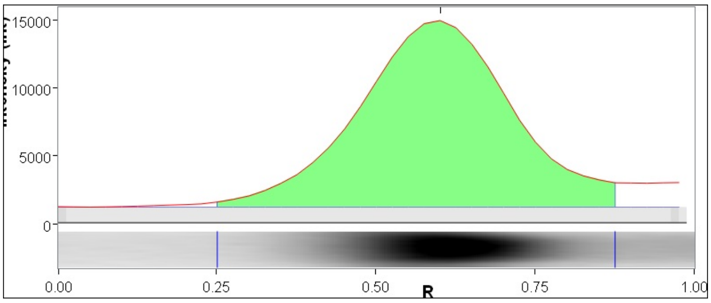

Band No.	Band Label	Mol. Wt. (KDa)	Relative Front	Adj. Volume (Int)	Volume (Int)	Abs. Quant.	Rel. Quant.	Band %	Lane %
1		N/A	0,625	6 304 904	7 511 486	N/A	N/A	100,0	95,1

Band Detection	Automatically detected bands with sensitivity: Low
Lane Background	Lane background subtracted with disk size: 24.1
Lane Width	4.72 mm

Lane 6

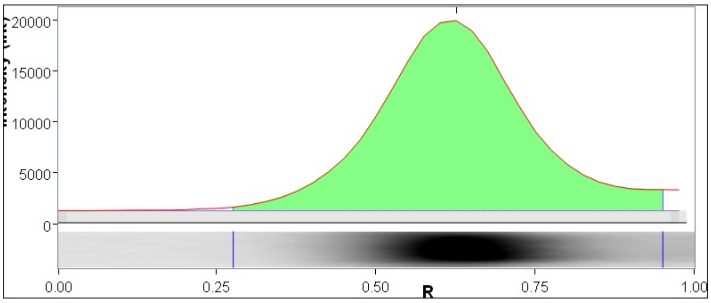

Band No.	Band Label	Mol. Wt. (KDa)	Relative Front	Adj. Volume (Int)	Volume (Int)	Abs. Quant.	Rel. Quant.	Band %	Lane %
1		N/A	0,650	6 667 635	7 743 955	N/A	N/A	100,0	98,6

Band Detection	Automatically detected bands with sensitivity: Low
Lane Background	Lane background subtracted with disk size: 24.1
Lane Width	4.72 mm

Lane 7

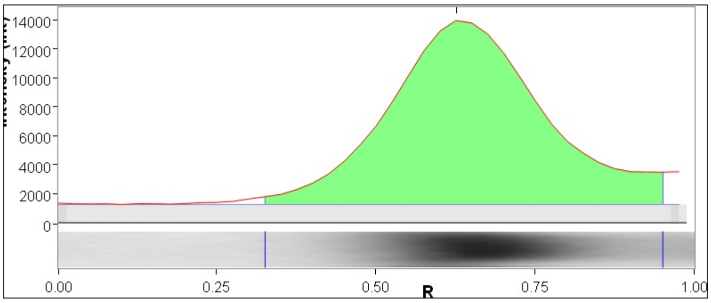

Band No.	Band Label	Mol. Wt. (KDa)	Relative Front	Adj. Volume (Int)	Volume (Int)	Abs. Quant.	Rel. Quant.	Band %	Lane %
1		N/A	0,650	4 746 624	5 813 248	N/A	N/A	100,0	97,6

Band Detection	Automatically detected bands with sensitivity: Low
Lane Background	Lane background subtracted with disk size: 24.1
Lane Width	4.87 mm

Lane 8

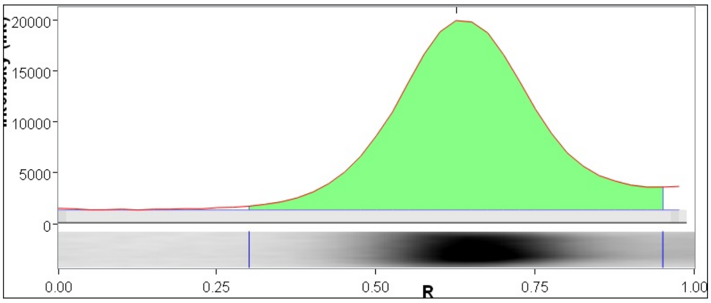

Band No.	Band Label	Mol. Wt. (KDa)	Relative Front	Adj. Volume (Int)	Volume (Int)	Abs. Quant.	Rel. Quant.	Band %	Lane %
1		N/A	0,650	6 321 365	7 386 866	N/A	N/A	100,0	98,2

Band Detection	Automatically detected bands with sensitivity: Low
Lane Background	Lane background subtracted with disk size: 24.1
Lane Width	4.72 mm

Lane 9

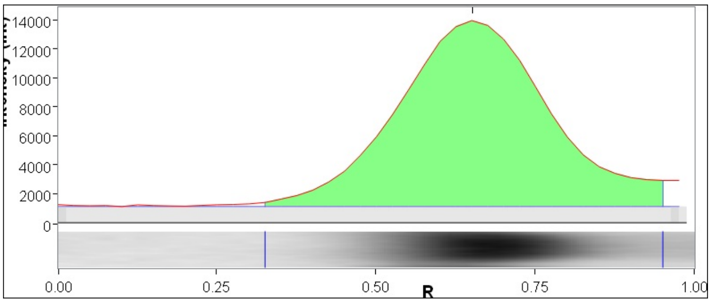

Band No.	Band Label	Mol. Wt. (KDa)	Relative Front	Adj. Volume (Int)	Volume (Int)	Abs. Quant.	Rel. Quant.	Band %	Lane %
1		N/A	0,675	5 171 360	6 215 520	N/A	N/A	100,0	98,0

Band Detection	Automatically detected bands with sensitivity: Low
Lane Background	Lane background subtracted with disk size: 24.1
Lane Width	4.87 mm

Lane 10

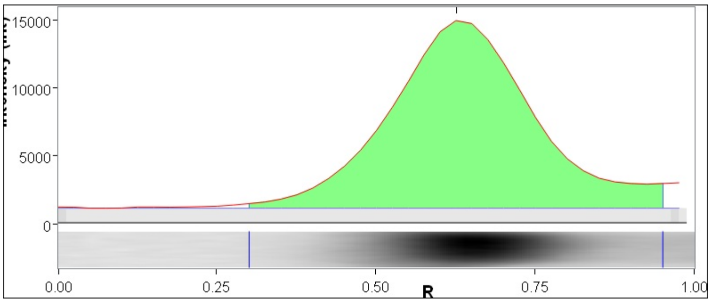

Band No.	Band Label	Mol. Wt. (KDa)	Relative Front	Adj. Volume (Int)	Volume (Int)	Abs. Quant.	Rel. Quant.	Band %	Lane %
1		N/A	0,650	5 075 280	6 077 250	N/A	N/A	100,0	98,1

Band Detection	Automatically detected bands with sensitivity: Low
Lane Background	Lane background subtracted with disk size: 24.1
Lane Width	4.57 mm

Lane 11

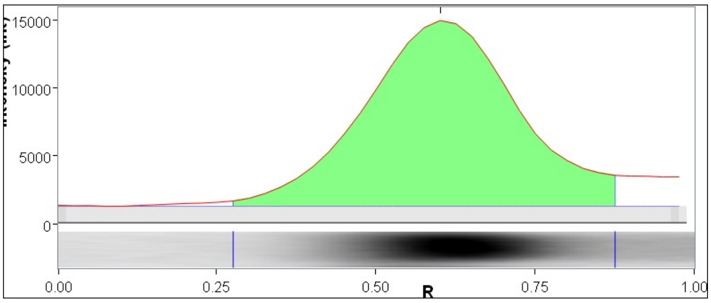

Band No.	Band Label	Mol. Wt. (KDa)	Relative Front	Adj. Volume (Int)	Volume (Int)	Abs. Quant.	Rel. Quant.	Band %	Lane %
1		N/A	0,625	5 952 441	7 110 741	N/A	N/A	100,0	94,0

Band Detection	Automatically detected bands with sensitivity: Low
Lane Background	Lane background subtracted with disk size: 24.1
Lane Width	5.03 mm

Lane 12

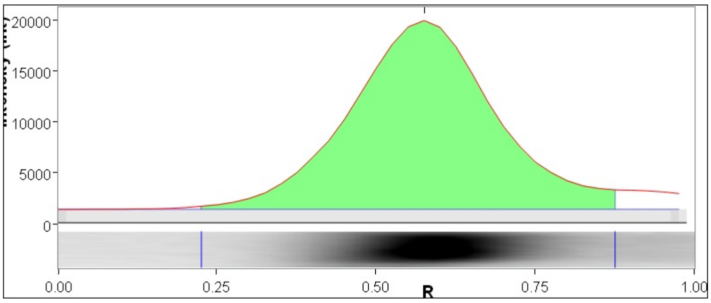

Band No.	Band Label	Mol. Wt. (KDa)	Relative Front	Adj. Volume (Int)	Volume (Int)	Abs. Quant.	Rel. Quant.	Band %	Lane %
1		N/A	0,600	6 230 411	7 339 436	N/A	N/A	100,0	96,4

Band Detection	Automatically detected bands with sensitivity: Low

Lane Background	Lane background subtracted with disk size: 24.1
Lane Width	4.72 mm

Lane 13

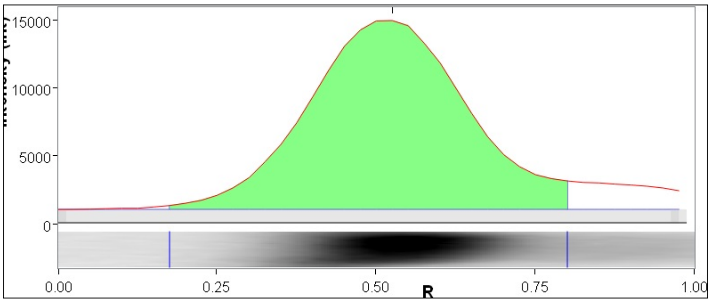

Band No.	Band Label	Mol. Wt. (KDa)	Relative Front	Adj. Volume (Int)	Volume (Int)	Abs. Quant.	Rel. Quant.	Band %	Lane %
1		N/A	0,550	6 420 379	7 407 729	N/A	N/A	100,0	92,8

Band Detection	Automatically detected bands with sensitivity: Low
Lane Background	Lane background subtracted with disk size: 24.1
Lane Width	4.72 mm

Lane 14

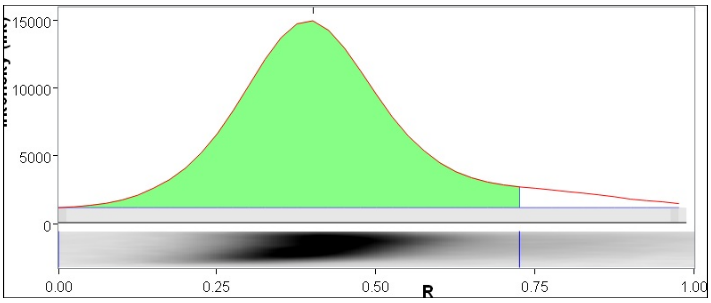

Band No.	Band Label	Mol. Wt. (KDa)	Relative Front	Adj. Volume (Int)	Volume (Int)	Abs. Quant.	Rel. Quant.	Band %	Lane %
1		N/A	0,425	6 685 000	8 081 500	N/A	N/A	100,0	94,8

Band Detection	Automatically detected bands with sensitivity: Low
Lane Background	Lane background subtracted with disk size: 24.1
Lane Width	5.33 mm

Image Report: Intensity analysis of Idh2 panel in Supplementary Fig. 1k, males

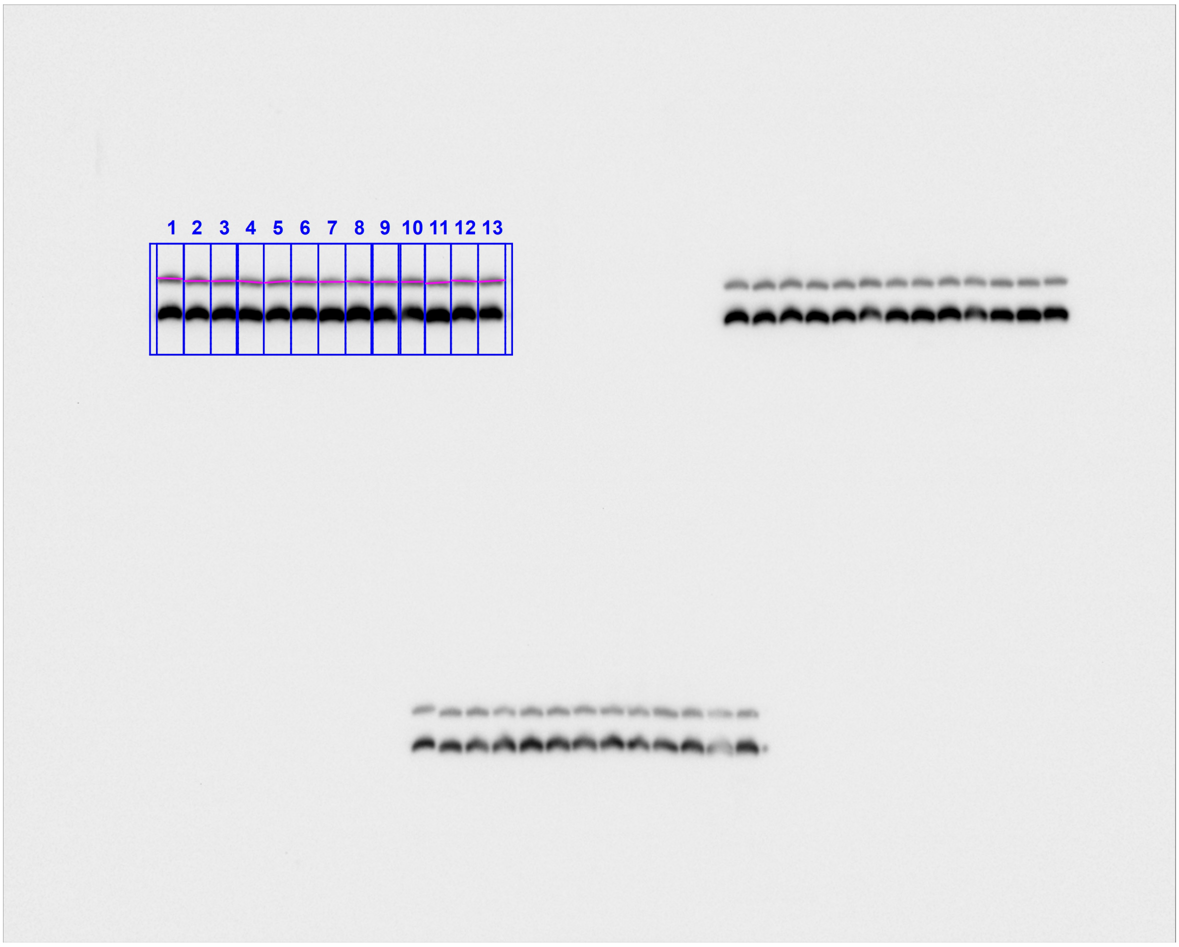

Acquisition Information

Imager	ChemiDoc Touch
Exposure Time (sec)	4.000 (Signal Accumulation)
Serial Number	732BR0263
Software Version	1.0.0.15
Application	Chemiluminescence
Excitation Source	No Illumination
Emission Filter	No Filter
Binning	2x2

Image Information

Acquisition Date	9/12/2019 8:06:21
User Name	
Image Area (mm)	X: 210.0 Y: 168.1
Pixel Size (µm)	X: 152.3 Y: 152.3
Data Range (Int)	500 - 38242

Analysis Settings

Detection	Lane detection:<br>Manually created lanes<br><br>Band detection:<br>Automatically detected bands with sensitivity: Low<br>Manually adjusted bands<br><br>Lane Background Subtraction:<br>Lane background subtracted with disk size: 10<br><br>Lane width: Variable

Lane Statistics

Lane No.	Adj. Total Band Vol. (Int)	Total Band Vol. (Int)	Adj. Total Lane Vol. (Int)	Total Lane Vol. (Int)	Bkgd. Vol. (Int)	Norm. Factor
1	2 780 832	3 344 768	14 883 328	18 269 152	3 385 824	N/A
2	2 571 200	3 146 592	13 983 296	17 507 296	3 524 000	N/A
3	2 635 424	3 249 536	14 486 080	18 075 296	3 589 216	N/A
4	2 567 712	3 163 072	14 445 536	17 975 808	3 530 272	N/A
5	2 599 328	3 216 128	14 409 952	18 020 256	3 610 304	N/A
6	2 586 816	3 191 232	15 325 184	18 867 936	3 542 752	N/A
7	1 923 552	2 451 840	15 867 424	19 377 664	3 510 240	N/A
8	2 188 224	2 766 304	16 490 112	20 097 152	3 607 040	N/A
9	2 210 976	2 785 984	14 539 584	18 090 176	3 550 592	N/A
10	2 722 027	3 315 367	12 581 244	15 901 222	3 319 978	N/A
11	2 255 281	2 794 061	16 486 544	19 884 516	3 397 972	N/A
12	2 336 128	2 942 496	14 452 032	18 029 312	3 577 280	N/A
13	2 562 560	3 138 528	12 944 192	16 369 664	3 425 472	N/A

Lane And Band Analysis

Lane 1

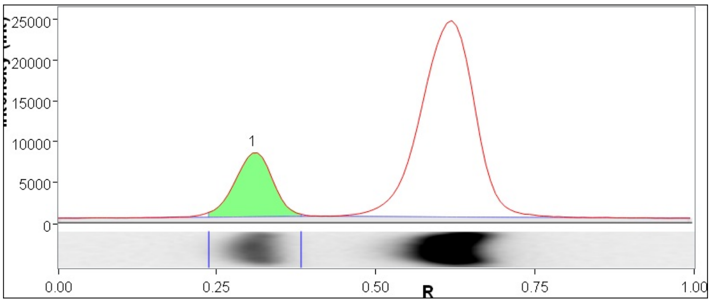

Band No.	Band Label	Mol. Wt. (KDa)	Relative Front	Adj. Volume (Int)	Volume (Int)	Abs. Quant.	Rel. Quant.	Band %	Lane %
1		N/A	0,313	2 780 832	3 344 768	N/A	N/A	100,0	18,7

Band Detection	Automatically detected bands with sensitivity: Low
Lane Background	Lane background subtracted with disk size: 10
Lane Width	4.87 mm

Lane 2

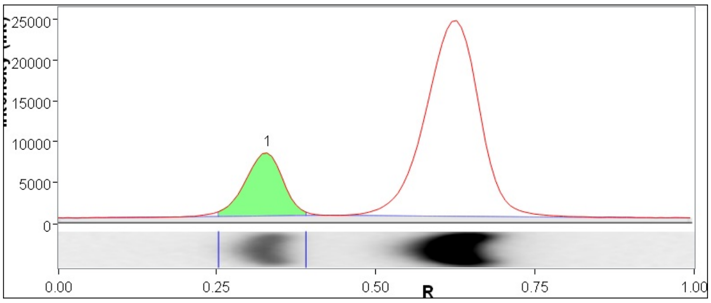

Band No.	Band Label	Mol. Wt. (KDa)	Relative Front	Adj. Volume (Int)	Volume (Int)	Abs. Quant.	Rel. Quant.	Band %	Lane %
1		N/A	0,336	2 571 200	3 146 592	N/A	N/A	100,0	18,4

Band Detection	Automatically detected bands with sensitivity: Low
Lane Background	Lane background subtracted with disk size: 10
Lane Width	4.87 mm

Lane 3

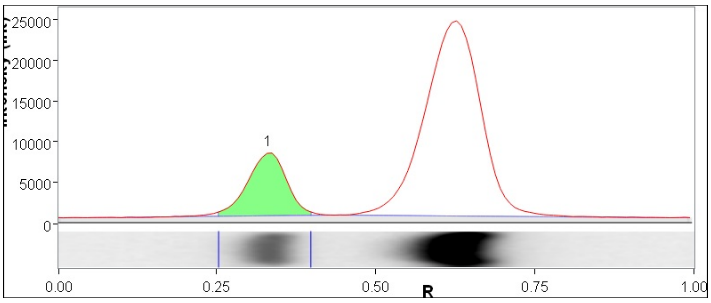

Band No.	Band Label	Mol. Wt. (KDa)	Relative Front	Adj. Volume (Int)	Volume (Int)	Abs. Quant.	Rel. Quant.	Band %	Lane %
1		N/A	0,336	2 635 424	3 249 536	N/A	N/A	100,0	18,2

Band Detection	Automatically detected bands with sensitivity: Low
Lane Background	Lane background subtracted with disk size: 10
Lane Width	4.87 mm

Lane 4

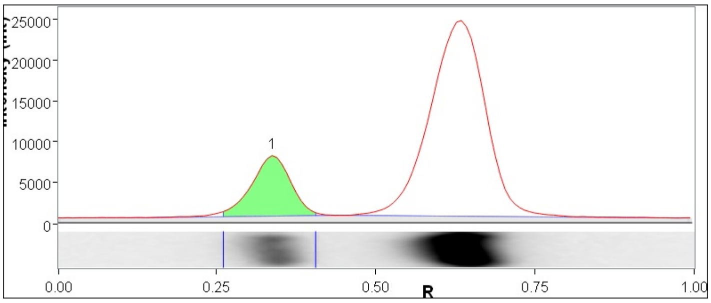

Band No.	Band Label	Mol. Wt. (KDa)	Relative Front	Adj. Volume (Int)	Volume (Int)	Abs. Quant.	Rel. Quant.	Band %	Lane %
1		N/A	0,344	2 567 712	3 163 072	N/A	N/A	100,0	17,8

Band Detection	Automatically detected bands with sensitivity: Low

Lane Background	Lane background subtracted with disk size: 10
Lane Width	4.87 mm

Lane 5

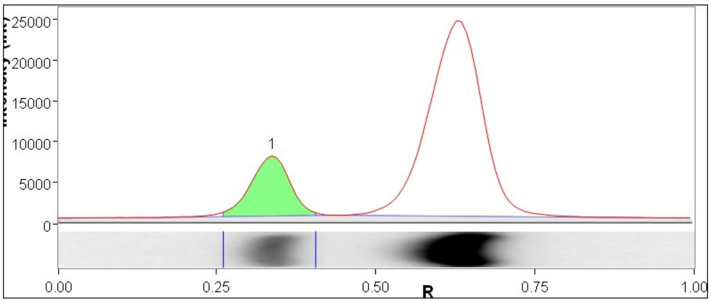

Band No.	Band Label	Mol. Wt. (KDa)	Relative Front	Adj. Volume (Int)	Volume (Int)	Abs. Quant.	Rel. Quant.	Band %	Lane %
1		N/A	0,344	2 599 328	3 216 128	N/A	N/A	100,0	18,0

Band Detection	Automatically detected bands with sensitivity: Low
Lane Background	Lane background subtracted with disk size: 10
Lane Width	4.87 mm

Lane 6

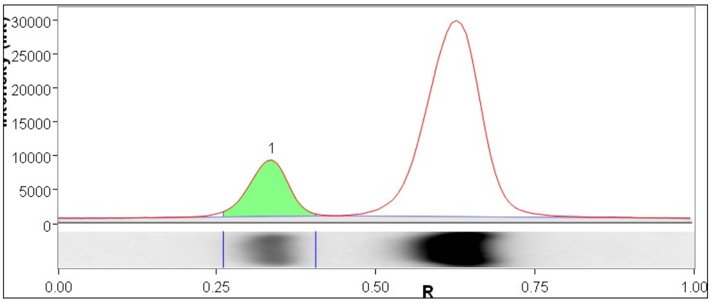

Band No.	Band Label	Mol. Wt. (KDa)	Relative Front	Adj. Volume (Int)	Volume (Int)	Abs. Quant.	Rel. Quant.	Band %	Lane %
1		N/A	0,344	2 586 816	3 191 232	N/A	N/A	100,0	16,9

Band Detection	Automatically detected bands with sensitivity: Low
Lane Background	Lane background subtracted with disk size: 10
Lane Width	4.87 mm

Lane 7

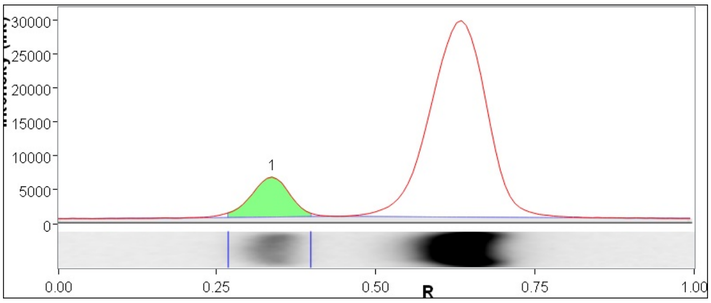

Band No.	Band Label	Mol. Wt. (KDa)	Relative Front	Adj. Volume (Int)	Volume (Int)	Abs. Quant.	Rel. Quant.	Band %	Lane %
1		N/A	0,344	1 923 552	2 451 840	N/A	N/A	100,0	12,1

Band Detection	Automatically detected bands with sensitivity: Low
Lane Background	Lane background subtracted with disk size: 10
Lane Width	4.87 mm

Lane 8

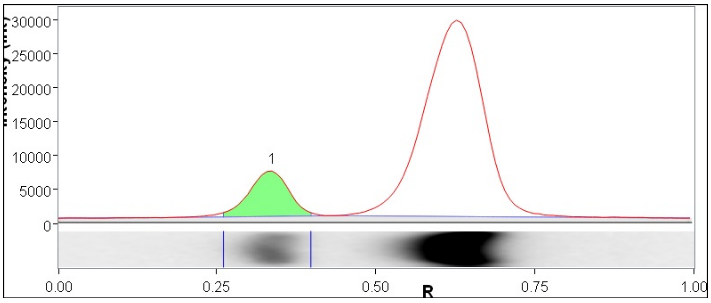

Band No.	Band Label	Mol. Wt. (KDa)	Relative Front	Adj. Volume (Int)	Volume (Int)	Abs. Quant.	Rel. Quant.	Band %	Lane %
1		N/A	0,344	2 188 224	2 766 304	N/A	N/A	100,0	13,3

Band Detection	Automatically detected bands with sensitivity: Low
Lane Background	Lane background subtracted with disk size: 10
Lane Width	4.87 mm

Lane 9

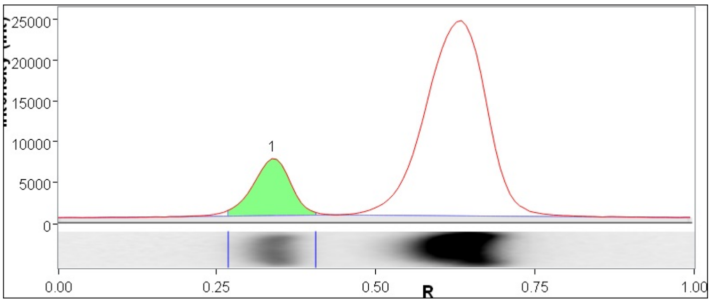

Band No.	Band Label	Mol. Wt. (KDa)	Relative Front	Adj. Volume (Int)	Volume (Int)	Abs. Quant.	Rel. Quant.	Band %	Lane %
1		N/A	0,344	2 210 976	2 785 984	N/A	N/A	100,0	15,2

Band Detection	Automatically detected bands with sensitivity: Low
Lane Background	Lane background subtracted with disk size: 10
Lane Width	4.87 mm

Lane 10

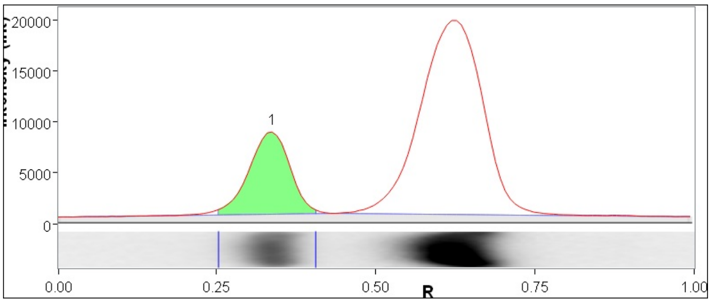

Band No.	Band Label	Mol. Wt. (KDa)	Relative Front	Adj. Volume (Int)	Volume (Int)	Abs. Quant.	Rel. Quant.	Band %	Lane %
1		N/A	0,344	2 722 027	3 315 367	N/A	N/A	100,0	21,6

Band Detection	Automatically detected bands with sensitivity: Low
Lane Background	Lane background subtracted with disk size: 10
Lane Width	4.42 mm

Lane 11

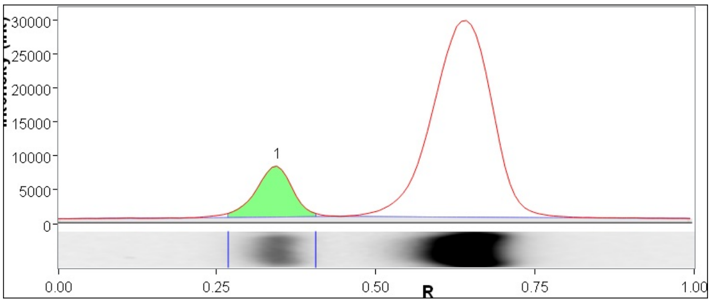

Band No.	Band Label	Mol. Wt. (KDa)	Relative Front	Adj. Volume (Int)	Volume (Int)	Abs. Quant.	Rel. Quant.	Band %	Lane %
1		N/A	0,351	2 255 281	2 794 061	N/A	N/A	100,0	13,7

Band Detection	Automatically detected bands with sensitivity: Low
Lane Background	Lane background subtracted with disk size: 10
Lane Width	4.72 mm

Lane 12

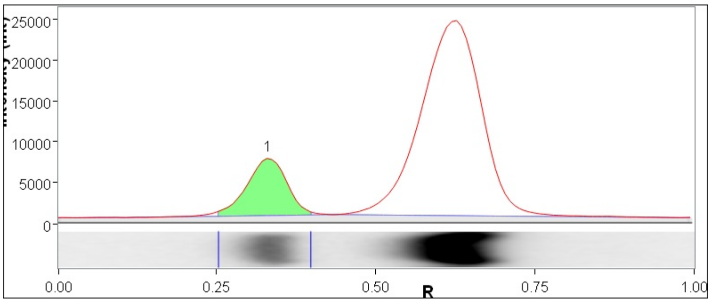

Band No.	Band Label	Mol. Wt. (KDa)	Relative Front	Adj. Volume (Int)	Volume (Int)	Abs. Quant.	Rel. Quant.	Band %	Lane %
1		N/A	0,336	2 336 128	2 942 496	N/A	N/A	100,0	16,2

Band Detection	Automatically detected bands with sensitivity: Low

Lane Background	Lane background subtracted with disk size: 10
Lane Width	4.87 mm

Lane 13

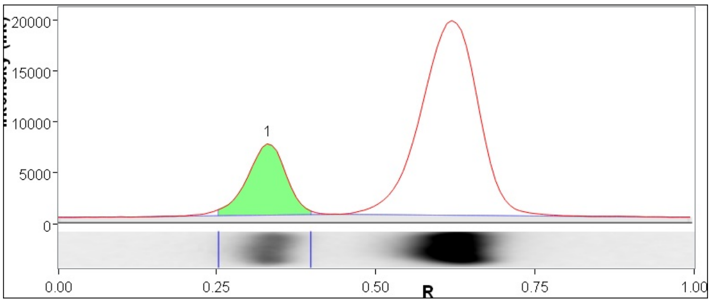

Band No.	Band Label	Mol. Wt. (KDa)	Relative Front	Adj. Volume (Int)	Volume (Int)	Abs. Quant.	Rel. Quant.	Band %	Lane %
1		N/A	0,336	2 562 560	3 138 528	N/A	N/A	100,0	19,8

Band Detection	Automatically detected bands with sensitivity: Low
Lane Background	Lane background subtracted with disk size: 10
Lane Width	4.87 mm

Image Report: Intensity analysis of Gapdh panel in Supplementary Fig. 1k, males

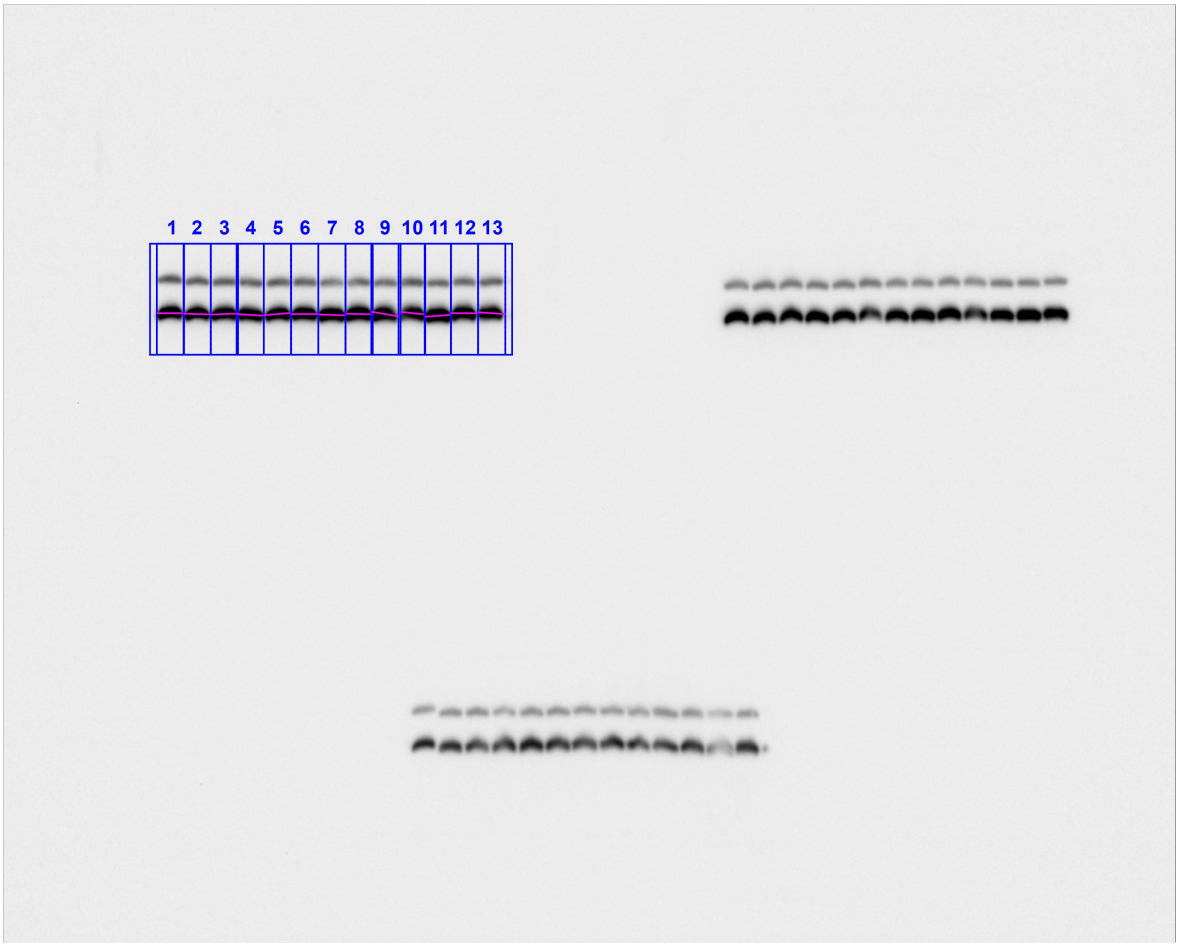

C:\Users\zahelb\OneDrive - KI.SE\Dokument\PhD\IDH2\Manuscript\Raw data sharing\Western blotting\Intensity analysis of Gapdh panel in figure S1K males.scn

Acquisition Information

Imager	ChemiDoc Touch
Exposure Time (sec)	4.000 (Signal Accumulation)
Serial Number	732BR0263
Software Version	1.0.0.15
Application	Chemiluminescence
Excitation Source	No Illumination
Emission Filter	No Filter
Binning	2x2

Image Information

Acquisition Date	9/12/2019 8:06:21
User Name	
Image Area (mm)	X: 210.0 Y: 168.1
Pixel Size (µm)	X: 152.3 Y: 152.3
Data Range (Int)	500 - 38242

Analysis Settings

Detection	Lane detection:<br>Manually created lanes<br><br>Band detection:<br>Automatically detected bands with sensitivity: Low<br>Manually adjusted bands<br><br>Lane Background Subtraction:<br>Lane background subtracted with disk size: 10<br><br>Lane width: Variable

Lane Statistics

Lane No.	Adj. Total Band Vol. (Int)	Total Band Vol. (Int)	Adj. Total Lane Vol. (Int)	Total Lane Vol. (Int)	Bkgd. Vol. (Int)	Norm. Factor
1	11 875 104	12 731 904	14 883 328	18 269 152	3 385 824	N/A
2	11 166 848	12 072 192	13 983 296	17 507 296	3 524 000	N/A
3	11 591 520	12 521 984	14 486 080	18 075 296	3 589 216	N/A
4	11 593 248	12 483 392	14 445 536	17 975 808	3 530 272	N/A
5	11 573 728	12 512 992	14 409 952	18 020 256	3 610 304	N/A
6	12 451 232	13 398 336	15 325 184	18 867 936	3 542 752	N/A
7	13 670 336	14 613 280	15 867 424	19 377 664	3 510 240	N/A
8	14 035 136	15 067 744	16 490 112	20 097 152	3 607 040	N/A
9	12 091 808	13 066 528	14 539 584	18 090 176	3 550 592	N/A
10	9 665 555	10 582 506	12 581 244	15 901 222	3 319 978	N/A
11	13 970 925	14 907 683	16 486 544	19 884 516	3 397 972	N/A
12	11 857 728	12 861 568	14 452 032	18 029 312	3 577 280	N/A
13	10 149 952	11 039 776	12 944 192	16 369 664	3 425 472	N/A

Lane And Band Analysis

Lane 1

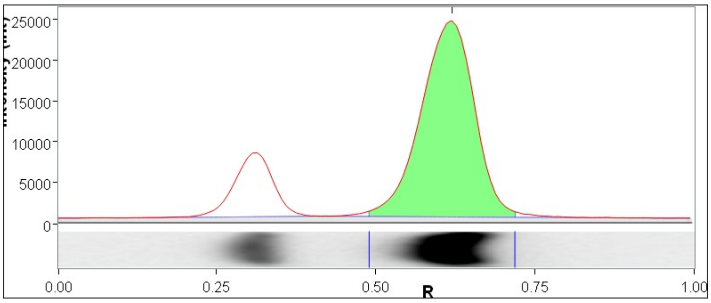

Band No.	Band Label	Mol. Wt. (KDa)	Relative Front	Adj. Volume (Int)	Volume (Int)	Abs. Quant.	Rel. Quant.	Band %	Lane %
1		N/A	0,626	11 875 104	12 731 904	N/A	N/A	100,0	79,8

Band Detection	Automatically detected bands with sensitivity: Low
Lane Background	Lane background subtracted with disk size: 10
Lane Width	4.87 mm

Lane 2

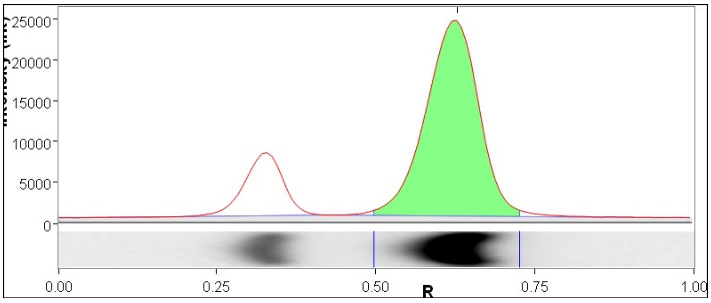

Band No.	Band Label	Mol. Wt. (KDa)	Relative Front	Adj. Volume (Int)	Volume (Int)	Abs. Quant.	Rel. Quant.	Band %	Lane %
1		N/A	0,634	11 166 848	12 072 192	N/A	N/A	100,0	79,9

Band Detection	Automatically detected bands with sensitivity: Low
Lane Background	Lane background subtracted with disk size: 10
Lane Width	4.87 mm

Lane 3

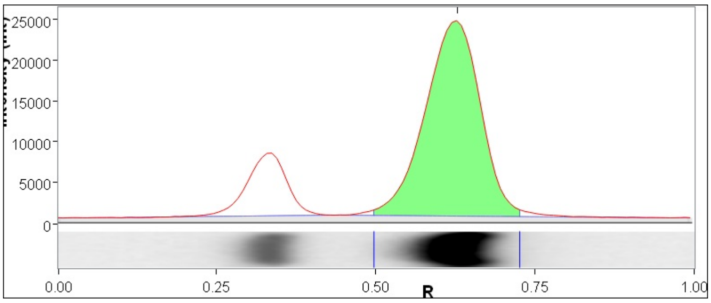

Band No.	Band Label	Mol. Wt. (KDa)	Relative Front	Adj. Volume (Int)	Volume (Int)	Abs. Quant.	Rel. Quant.	Band %	Lane %
1		N/A	0,634	11 591 520	12 521 984	N/A	N/A	100,0	80,0

Band Detection	Automatically detected bands with sensitivity: Low
Lane Background	Lane background subtracted with disk size: 10
Lane Width	4.87 mm

Lane 4

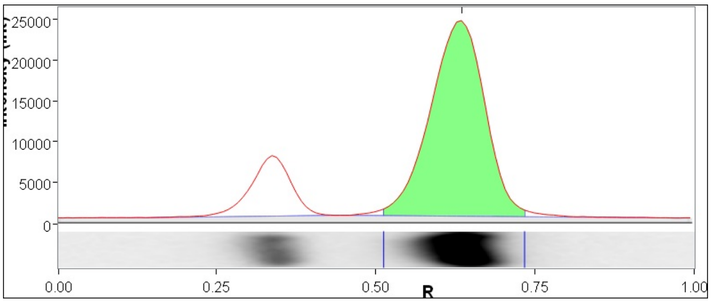

Band No.	Band Label	Mol. Wt. (KDa)	Relative Front	Adj. Volume (Int)	Volume (Int)	Abs. Quant.	Rel. Quant.	Band %	Lane %
1		N/A	0,641	11 593 248	12 483 392	N/A	N/A	100,0	80,3

Band Detection	Automatically detected bands with sensitivity: Low

Lane Background	Lane background subtracted with disk size: 10
Lane Width	4.87 mm

Lane 5

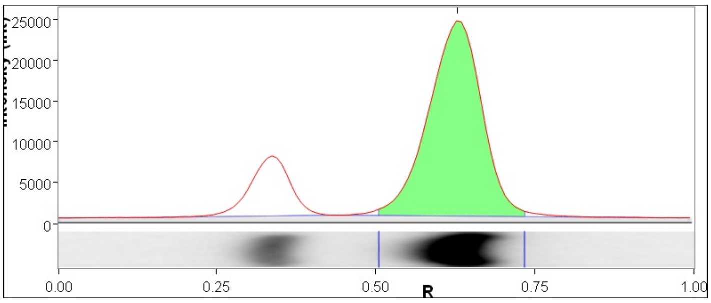

Band No.	Band Label	Mol. Wt. (KDa)	Relative Front	Adj. Volume (Int)	Volume (Int)	Abs. Quant.	Rel. Quant.	Band %	Lane %
1		N/A	0,634	11 573 728	12 512 992	N/A	N/A	100,0	80,3

Band Detection	Automatically detected bands with sensitivity: Low
Lane Background	Lane background subtracted with disk size: 10
Lane Width	4.87 mm

Lane 6

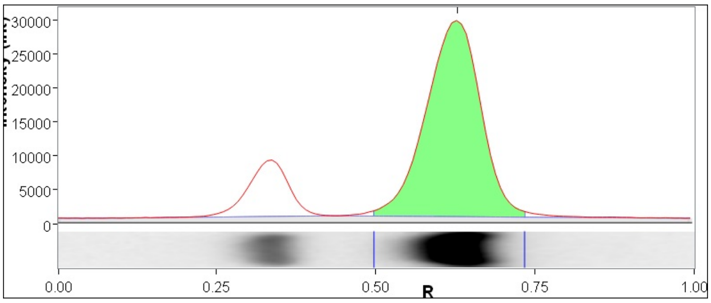

Band No.	Band Label	Mol. Wt. (KDa)	Relative Front	Adj. Volume (Int)	Volume (Int)	Abs. Quant.	Rel. Quant.	Band %	Lane %
1		N/A	0,634	12 451 232	13 398 336	N/A	N/A	100,0	81,2

Band Detection	Automatically detected bands with sensitivity: Low
Lane Background	Lane background subtracted with disk size: 10
Lane Width	4.87 mm

Lane 7

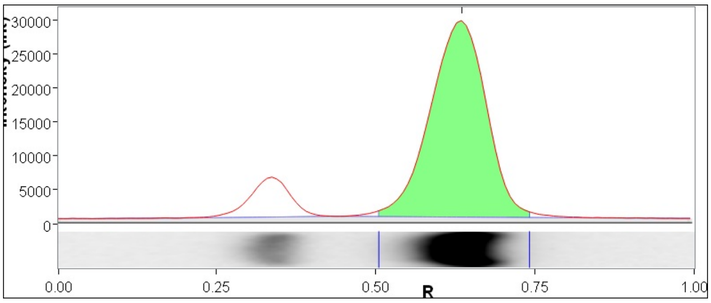

Band No.	Band Label	Mol. Wt. (KDa)	Relative Front	Adj. Volume (Int)	Volume (Int)	Abs. Quant.	Rel. Quant.	Band %	Lane %
1		N/A	0,641	13 670 336	14 613 280	N/A	N/A	100,0	86,2

Band Detection	Automatically detected bands with sensitivity: Low
Lane Background	Lane background subtracted with disk size: 10
Lane Width	4.87 mm

Lane 8

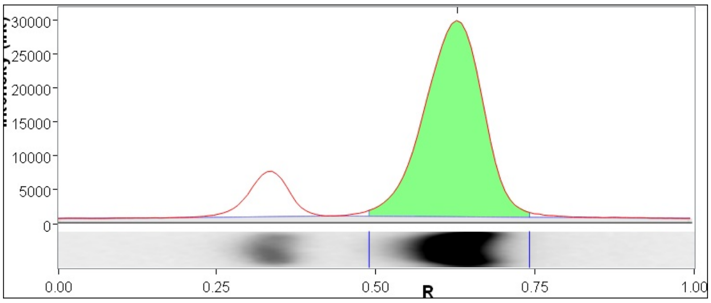

Band No.	Band Label	Mol. Wt. (KDa)	Relative Front	Adj. Volume (Int)	Volume (Int)	Abs. Quant.	Rel. Quant.	Band %	Lane %
1		N/A	0,634	14 035 136	15 067 744	N/A	N/A	100,0	85,1

Band Detection	Automatically detected bands with sensitivity: Low
Lane Background	Lane background subtracted with disk size: 10
Lane Width	4.87 mm

Lane 9

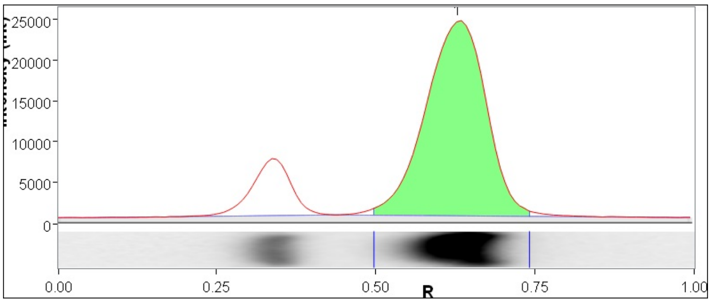

Band No.	Band Label	Mol. Wt. (KDa)	Relative Front	Adj. Volume (Int)	Volume (Int)	Abs. Quant.	Rel. Quant.	Band %	Lane %
1		N/A	0,634	12 091 808	13 066 528	N/A	N/A	100,0	83,2

Band Detection	Automatically detected bands with sensitivity: Low
Lane Background	Lane background subtracted with disk size: 10
Lane Width	4.87 mm

Lane 10

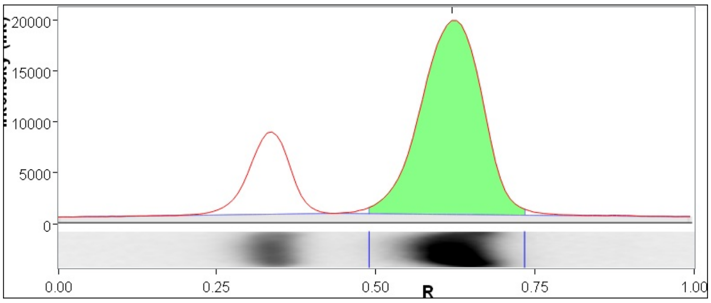

Band No.	Band Label	Mol. Wt. (KDa)	Relative Front	Adj. Volume (Int)	Volume (Int)	Abs. Quant.	Rel. Quant.	Band %	Lane %
1		N/A	0,626	9 665 555	10 582 506	N/A	N/A	100,0	76,8

Band Detection	Automatically detected bands with sensitivity: Low
Lane Background	Lane background subtracted with disk size: 10
Lane Width	4.42 mm

Lane 11

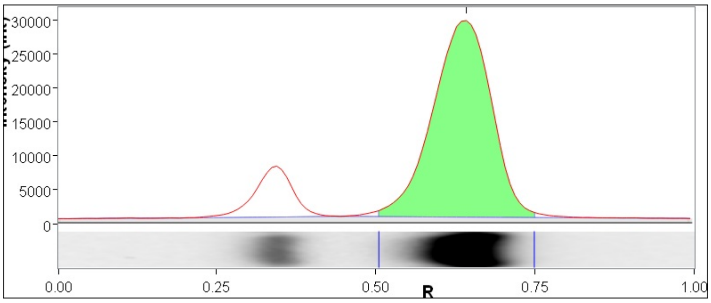

Band No.	Band Label	Mol. Wt. (KDa)	Relative Front	Adj. Volume (Int)	Volume (Int)	Abs. Quant.	Rel. Quant.	Band %	Lane %
1		N/A	0,649	13 970 925	14 907 683	N/A	N/A	100,0	84,7

Band Detection	Automatically detected bands with sensitivity: Low
Lane Background	Lane background subtracted with disk size: 10
Lane Width	4.72 mm

Lane 12

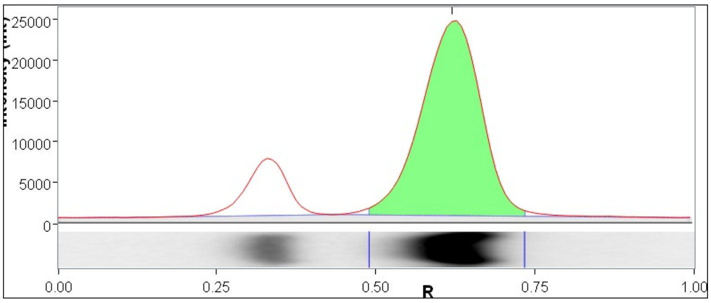

Band No.	Band Label	Mol. Wt. (KDa)	Relative Front	Adj. Volume (Int)	Volume (Int)	Abs. Quant.	Rel. Quant.	Band %	Lane %
1		N/A	0,626	11 857 728	12 861 568	N/A	N/A	100,0	82,0

Band Detection	Automatically detected bands with sensitivity: Low

Lane Background	Lane background subtracted with disk size: 10
Lane Width	4.87 mm

Lane 13

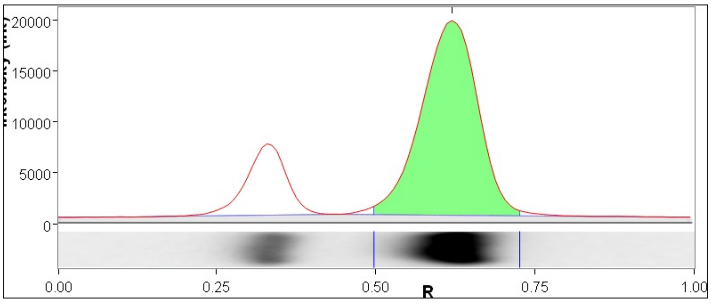

Band No.	Band Label	Mol. Wt. (KDa)	Relative Front	Adj. Volume (Int)	Volume (Int)	Abs. Quant.	Rel. Quant.	Band %	Lane %
1		N/A	0,626	10 149 952	11 039 776	N/A	N/A	100,0	78,4

Band Detection	Automatically detected bands with sensitivity: Low
Lane Background	Lane background subtracted with disk size: 10
Lane Width	4.87 mm

Image Report: Intensity analysis of Idh2 panel in Supplementary Fig. 1k, females

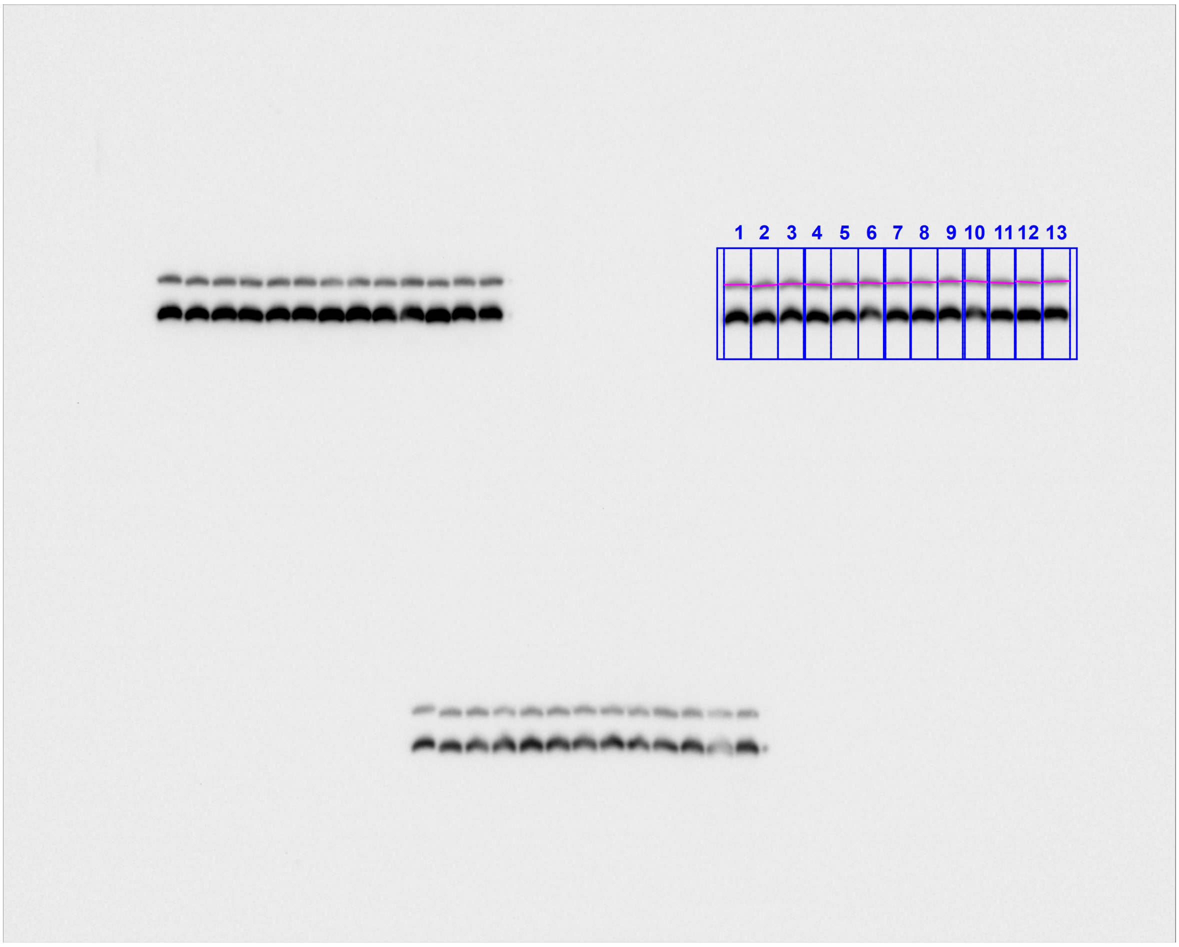

C:\Users\zahelb\OneDrive - KI.SE\Dokument\PhD\IDH2\Manuscript\Raw data sharing\Western blotting\Intensity analysis of Idh2 panel in figure S1K females.scn

Acquisition Information

Imager	ChemiDoc Touch
Exposure Time (sec)	4.000 (Signal Accumulation)
Serial Number	732BR0263
Software Version	1.0.0.15
Application	Chemiluminescence
Excitation Source	No Illumination
Emission Filter	No Filter
Binning	2x2

Image Information

Acquisition Date	9/12/2019 8:06:21
User Name	
Image Area (mm)	X: 210.0 Y: 168.1
Pixel Size (µm)	X: 152.3 Y: 152.3
Data Range (Int)	500 - 38242

Analysis Settings

Detection	Lane detection:<br>Manually created lanes<br><br>Band detection:<br>Automatically detected bands with sensitivity: Low<br>Manually adjusted bands<br><br>Lane Background Subtraction:<br>Lane background subtracted with disk size: 10<br><br>Lane width: Variable

Lane Statistics

Lane No.	Adj. Total Band Vol. (Int)	Total Band Vol. (Int)	Adj. Total Lane Vol. (Int)	Total Lane Vol. (Int)	Bkgd. Vol. (Int)	Norm. Factor
1	1 971 744	2 494 144	11 712 416	14 852 096	3 139 680	N/A
2	2 164 864	2 720 896	10 595 328	13 926 816	3 331 488	N/A
3	2 142 592	2 723 232	10 634 176	13 939 840	3 305 664	N/A
4	1 988 736	2 526 176	11 235 968	14 466 304	3 230 336	N/A
5	2 041 696	2 538 560	10 519 072	13 703 296	3 184 224	N/A
6	2 158 016	2 690 624	8 563 456	11 731 968	3 168 512	N/A
7	1 762 528	2 250 848	10 336 512	13 461 152	3 124 640	N/A
8	1 754 912	2 261 248	11 407 520	14 640 352	3 232 832	N/A
9	1 892 256	2 410 016	11 607 200	14 750 400	3 143 200	N/A
10	1 598 190	2 037 482	7 903 080	10 724 896	2 821 816	N/A
11	1 945 312	2 424 448	10 843 273	13 798 658	2 955 385	N/A
12	1 821 696	2 307 680	12 755 328	15 882 720	3 127 392	N/A
13	1 719 008	2 171 936	11 045 984	14 124 640	3 078 656	N/A

Lane And Band Analysis

Lane 1

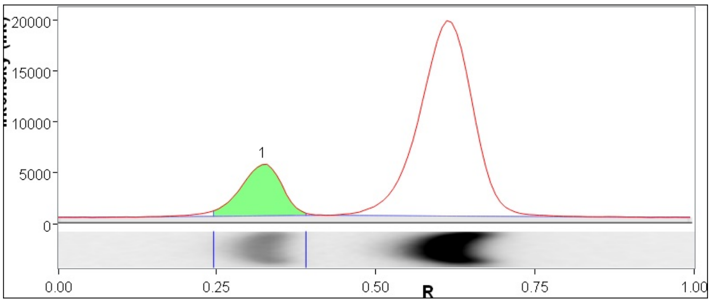

Band No.	Band Label	Mol. Wt. (KDa)	Relative Front	Adj. Volume (Int)	Volume (Int)	Abs. Quant.	Rel. Quant.	Band %	Lane %
1		N/A	0,328	1 971 744	2 494 144	N/A	N/A	100,0	16,8

Band Detection	Automatically detected bands with sensitivity: Low
Lane Background	Lane background subtracted with disk size: 10
Lane Width	4.87 mm

Lane 2

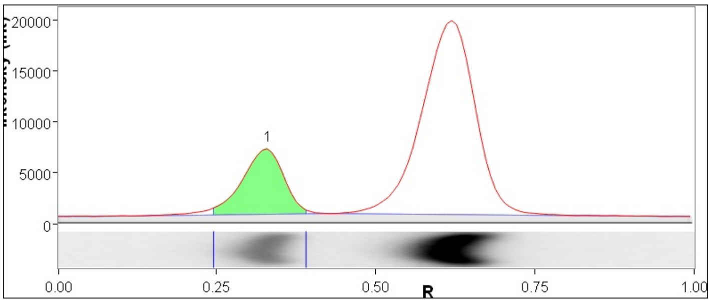

Band No.	Band Label	Mol. Wt. (KDa)	Relative Front	Adj. Volume (Int)	Volume (Int)	Abs. Quant.	Rel. Quant.	Band %	Lane %
1		N/A	0,336	2 164 864	2 720 896	N/A	N/A	100,0	20,4

Band Detection	Automatically detected bands with sensitivity: Low
Lane Background	Lane background subtracted with disk size: 10
Lane Width	4.87 mm

Lane 3

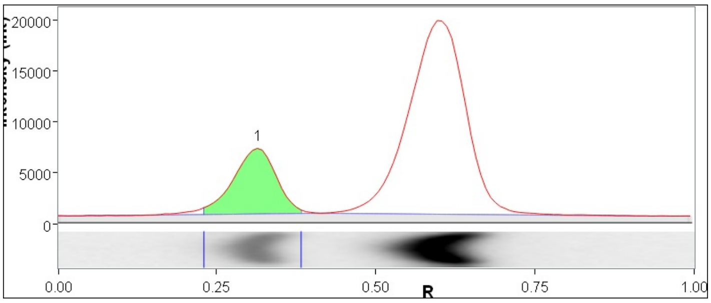

Band No.	Band Label	Mol. Wt. (KDa)	Relative Front	Adj. Volume (Int)	Volume (Int)	Abs. Quant.	Rel. Quant.	Band %	Lane %
1		N/A	0,321	2 142 592	2 723 232	N/A	N/A	100,0	20,1

Band Detection	Automatically detected bands with sensitivity: Low
Lane Background	Lane background subtracted with disk size: 10
Lane Width	4.87 mm

Lane 4

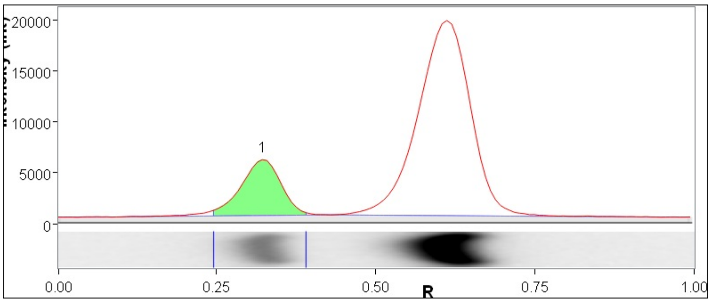

Band No.	Band Label	Mol. Wt. (KDa)	Relative Front	Adj. Volume (Int)	Volume (Int)	Abs. Quant.	Rel. Quant.	Band %	Lane %
1		N/A	0,328	1 988 736	2 526 176	N/A	N/A	100,0	17,7

Band Detection	Automatically detected bands with sensitivity: Low

Lane Background	Lane background subtracted with disk size: 10
Lane Width	4.87 mm

Lane 5

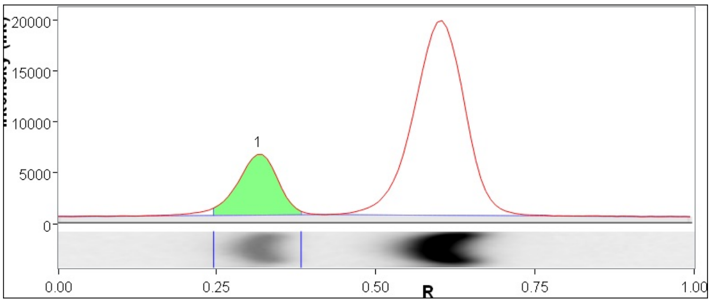

Band No.	Band Label	Mol. Wt. (KDa)	Relative Front	Adj. Volume (Int)	Volume (Int)	Abs. Quant.	Rel. Quant.	Band %	Lane %
1		N/A	0,321	2 041 696	2 538 560	N/A	N/A	100,0	19,4

Band Detection	Automatically detected bands with sensitivity: Low
Lane Background	Lane background subtracted with disk size: 10
Lane Width	4.87 mm

Lane 6

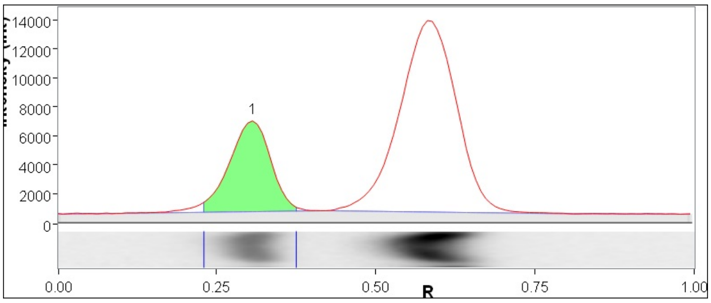

Band No.	Band Label	Mol. Wt. (KDa)	Relative Front	Adj. Volume (Int)	Volume (Int)	Abs. Quant.	Rel. Quant.	Band %	Lane %
1		N/A	0,313	2 158 016	2 690 624	N/A	N/A	100,0	25,2

Band Detection	Automatically detected bands with sensitivity: Low
Lane Background	Lane background subtracted with disk size: 10
Lane Width	4.87 mm

Lane 7

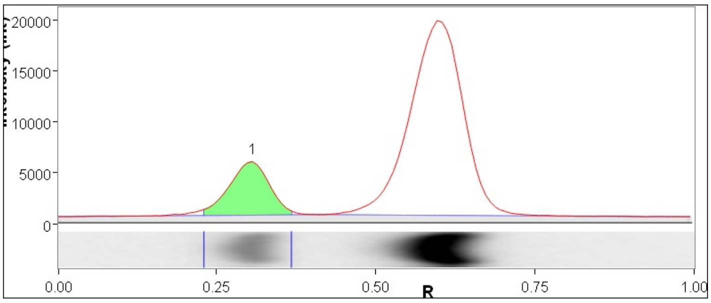

Band No.	Band Label	Mol. Wt. (KDa)	Relative Front	Adj. Volume (Int)	Volume (Int)	Abs. Quant.	Rel. Quant.	Band %	Lane %
1		N/A	0,313	1 762 528	2 250 848	N/A	N/A	100,0	17,1

Band Detection	Automatically detected bands with sensitivity: Low
Lane Background	Lane background subtracted with disk size: 10
Lane Width	4.87 mm

Lane 8

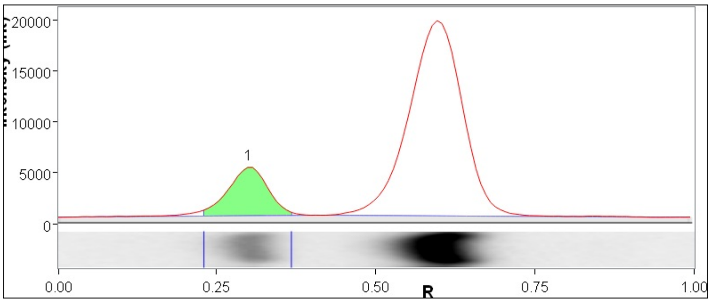

Band No.	Band Label	Mol. Wt. (KDa)	Relative Front	Adj. Volume (Int)	Volume (Int)	Abs. Quant.	Rel. Quant.	Band %	Lane %
1		N/A	0,305	1 754 912	2 261 248	N/A	N/A	100,0	15,4

Band Detection	Automatically detected bands with sensitivity: Low
Lane Background	Lane background subtracted with disk size: 10
Lane Width	4.87 mm

Lane 9

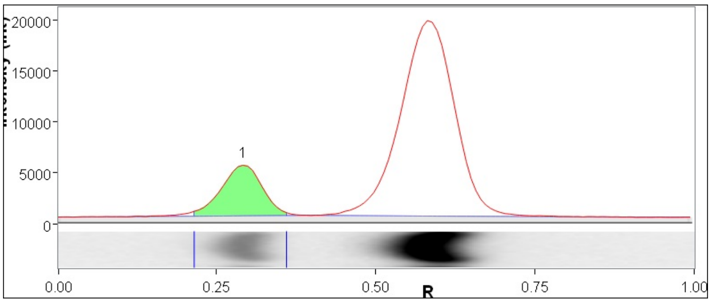

Band No.	Band Label	Mol. Wt. (KDa)	Relative Front	Adj. Volume (Int)	Volume (Int)	Abs. Quant.	Rel. Quant.	Band %	Lane %
1		N/A	0,298	1 892 256	2 410 016	N/A	N/A	100,0	16,3

Band Detection	Automatically detected bands with sensitivity: Low
Lane Background	Lane background subtracted with disk size: 10
Lane Width	4.87 mm

Lane 10

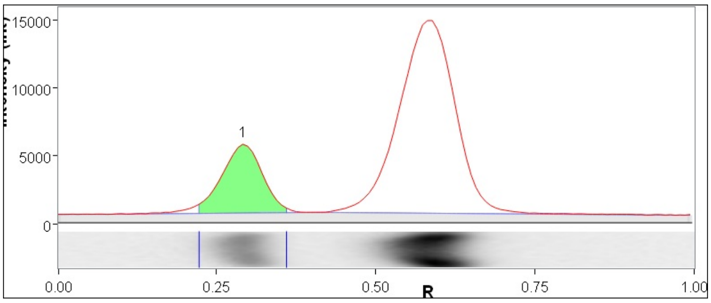

Band No.	Band Label	Mol. Wt. (KDa)	Relative Front	Adj. Volume (Int)	Volume (Int)	Abs. Quant.	Rel. Quant.	Band %	Lane %
1		N/A	0,298	1 598 190	2 037 482	N/A	N/A	100,0	20,2

Band Detection	Automatically detected bands with sensitivity: Low
Lane Background	Lane background subtracted with disk size: 10
Lane Width	4.42 mm

Lane 11

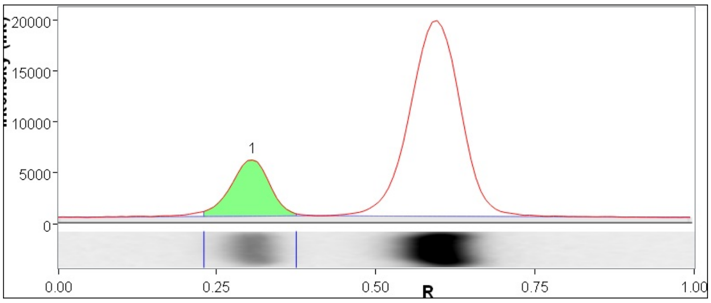

Band No.	Band Label	Mol. Wt. (KDa)	Relative Front	Adj. Volume (Int)	Volume (Int)	Abs. Quant.	Rel. Quant.	Band %	Lane %
1		N/A	0,313	1 945 312	2 424 448	N/A	N/A	100,0	17,9

Band Detection	Automatically detected bands with sensitivity: Low
Lane Background	Lane background subtracted with disk size: 10
Lane Width	4.72 mm

Lane 12

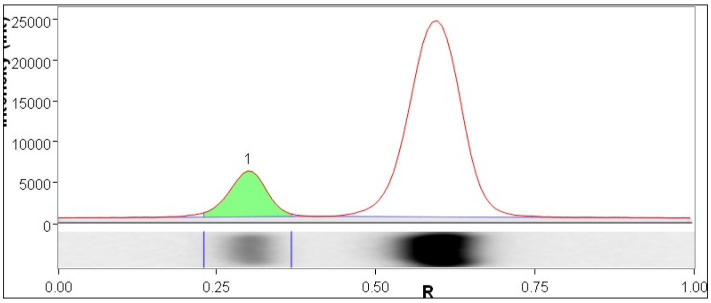

Band No.	Band Label	Mol. Wt. (KDa)	Relative Front	Adj. Volume (Int)	Volume (Int)	Abs. Quant.	Rel. Quant.	Band %	Lane %
1		N/A	0,305	1 821 696	2 307 680	N/A	N/A	100,0	14,3

Band Detection	Automatically detected bands with sensitivity: Low

Lane Background	Lane background subtracted with disk size: 10
Lane Width	4.87 mm

Lane 13

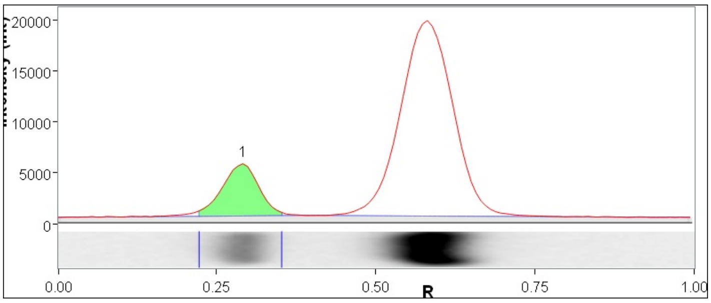

Band No.	Band Label	Mol. Wt. (KDa)	Relative Front	Adj. Volume (Int)	Volume (Int)	Abs. Quant.	Rel. Quant.	Band %	Lane %
1		N/A	0,298	1 719 008	2 171 936	N/A	N/A	100,0	15,6

Band Detection	Automatically detected bands with sensitivity: Low
Lane Background	Lane background subtracted with disk size: 10
Lane Width	4.87 mm

Image Report: Intensity analysis of Gapdh panel in Supplementary Fig.1k females

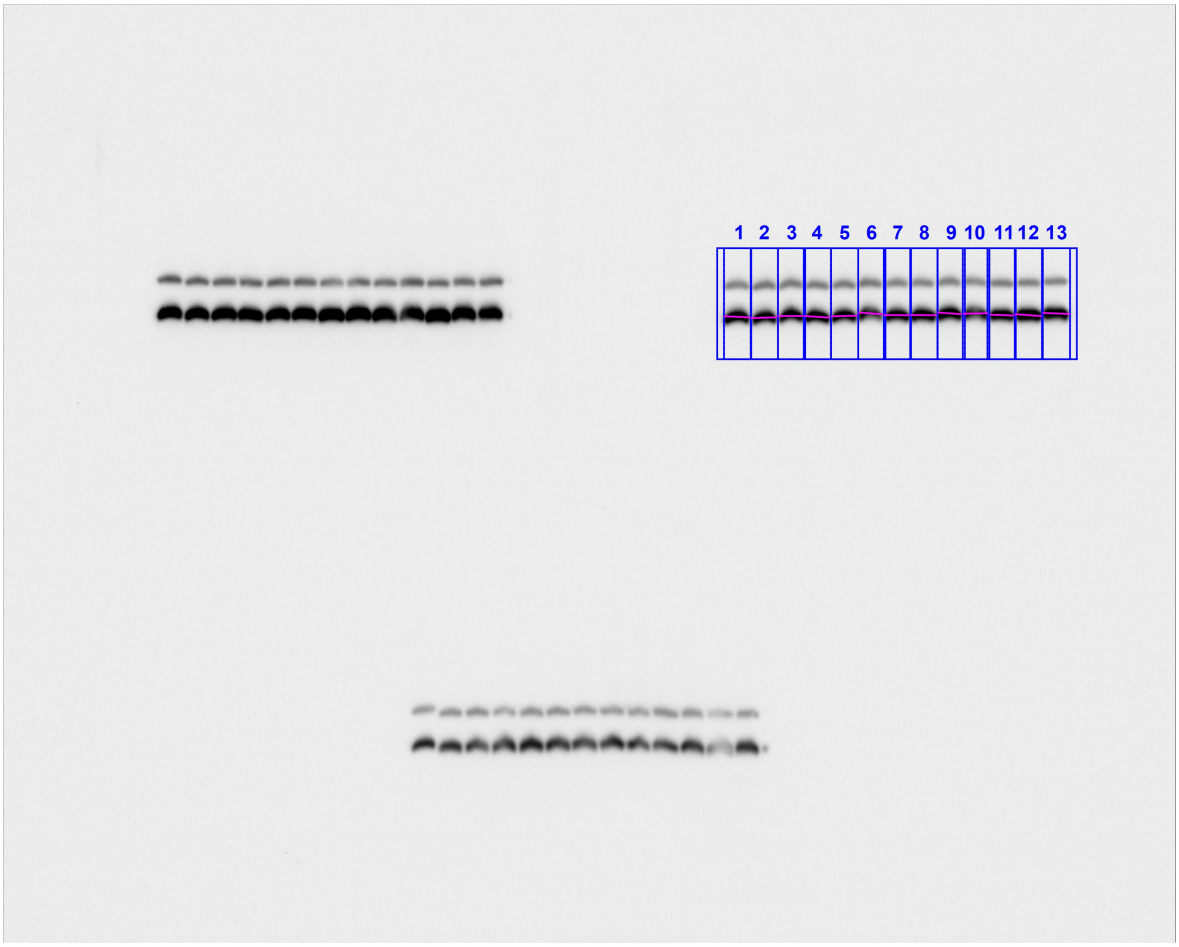

Acquisition Information

Imager	ChemiDoc Touch
Exposure Time (sec)	4.000 (Signal Accumulation)
Serial Number	732BR0263
Software Version	1.0.0.15
Application	Chemiluminescence
Excitation Source	No Illumination
Emission Filter	No Filter
Binning	2x2

Image Information

Acquisition Date	9/12/2019 8:06:21
User Name	
Image Area (mm)	X: 210.0 Y: 168.1
Pixel Size (µm)	X: 152.3 Y: 152.3

Data Range (Int)	500 - 38242

Analysis Settings

Detection	Lane detection:<br>Manually created lanes<br><br>Band detection:<br>Automatically detected bands with sensitivity: Low<br>Manually adjusted bands<br><br>Lane Background Subtraction:<br>Lane background subtracted with disk size: 10<br><br>Lane width: Variable

Lane Statistics

Lane No.	Adj. Total Band Vol. (Int)	Total Band Vol. (Int)	Adj. Total Lane Vol. (Int)	Total Lane Vol. (Int)	Bkgd. Vol. (Int)	Norm. Factor
1	9 497 088	10 293 184	11 712 416	14 852 096	3 139 680	N/A
2	8 192 800	9 016 640	10 595 328	13 926 816	3 331 488	N/A
3	8 271 616	9 111 136	10 634 176	13 939 840	3 305 664	N/A
4	9 022 016	9 818 144	11 235 968	14 466 304	3 230 336	N/A
5	8 240 832	8 986 496	10 519 072	13 703 296	3 184 224	N/A
6	6 189 152	6 938 976	8 563 456	11 731 968	3 168 512	N/A
7	8 366 816	9 123 040	10 336 512	13 461 152	3 124 640	N/A
8	9 427 936	10 239 584	11 407 520	14 640 352	3 232 832	N/A
9	9 518 688	10 328 160	11 607 200	14 750 400	3 143 200	N/A
10	6 091 189	6 753 056	7 903 080	10 724 896	2 821 816	N/A
11	8 658 300	9 347 585	10 843 273	13 798 658	2 955 385	N/A
12	10 723 200	11 508 672	12 755 328	15 882 720	3 127 392	N/A
13	9 133 248	9 882 944	11 045 984	14 124 640	3 078 656	N/A

Lane And Band Analysis

Lane 1

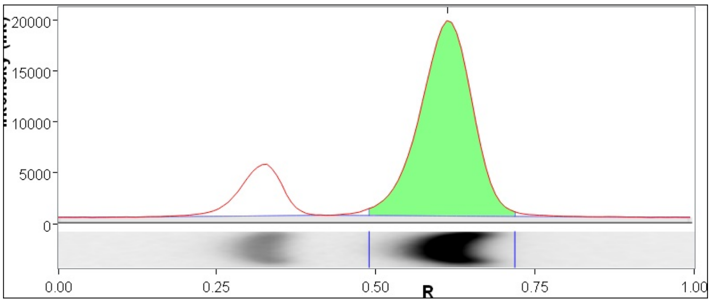

Band No.	Band Label	Mol. Wt. (KDa)	Relative Front	Adj. Volume (Int)	Volume (Int)	Abs. Quant.	Rel. Quant.	Band %	Lane %
1		N/A	0,618	9 497 088	10 293 184	N/A	N/A	100,0	81,1

Band Detection	Automatically detected bands with sensitivity: Low
Lane Background	Lane background subtracted with disk size: 10
Lane Width	4.87 mm

Lane 2

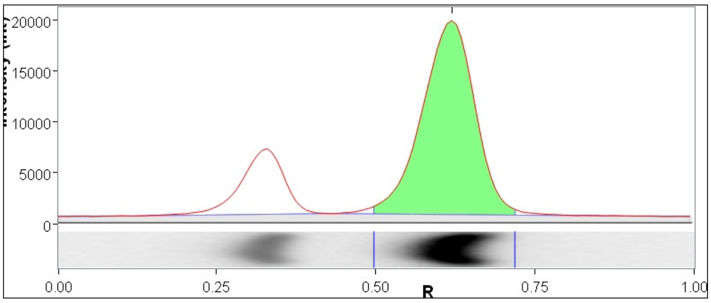

Band No.	Band Label	Mol. Wt. (KDa)	Relative Front	Adj. Volume (Int)	Volume (Int)	Abs. Quant.	Rel. Quant.	Band %	Lane %
1		N/A	0,626	8 192 800	9 016 640	N/A	N/A	100,0	77,3

Band Detection	Automatically detected bands with sensitivity: Low
Lane Background	Lane background subtracted with disk size: 10
Lane Width	4.87 mm

Lane 3

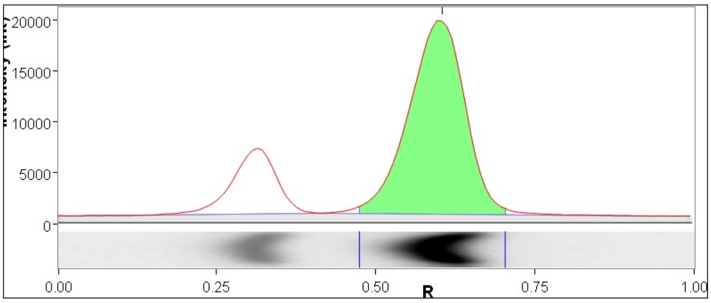

Band No.	Band Label	Mol. Wt. (KDa)	Relative Front	Adj. Volume (Int)	Volume (Int)	Abs. Quant.	Rel. Quant.	Band %	Lane %
1		N/A	0,611	8 271 616	9 111 136	N/A	N/A	100,0	77,8

Band Detection	Automatically detected bands with sensitivity: Low
Lane Background	Lane background subtracted with disk size: 10
Lane Width	4.87 mm

Lane 4

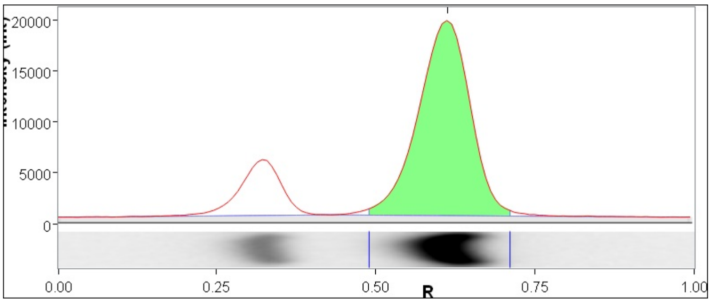

Band No.	Band Label	Mol. Wt. (KDa)	Relative Front	Adj. Volume (Int)	Volume (Int)	Abs. Quant.	Rel. Quant.	Band %	Lane %
1		N/A	0,618	9 022 016	9 818 144	N/A	N/A	100,0	80,3

Band Detection	Automatically detected bands with sensitivity: Low

Lane Background	Lane background subtracted with disk size: 10
Lane Width	4.87 mm

Lane 5

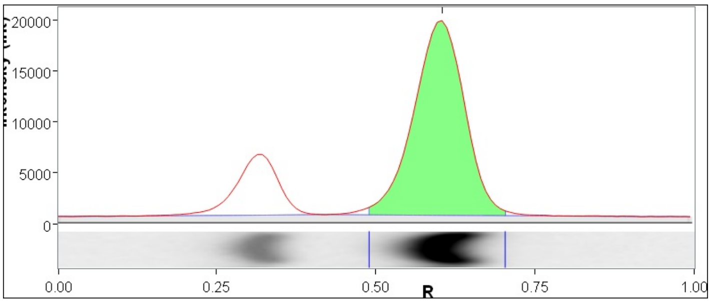

Band No.	Band Label	Mol. Wt. (KDa)	Relative Front	Adj. Volume (Int)	Volume (Int)	Abs. Quant.	Rel. Quant.	Band %	Lane %
1		N/A	0,611	8 240 832	8 986 496	N/A	N/A	100,0	78,3

Band Detection	Automatically detected bands with sensitivity: Low
Lane Background	Lane background subtracted with disk size: 10
Lane Width	4.87 mm

Lane 6

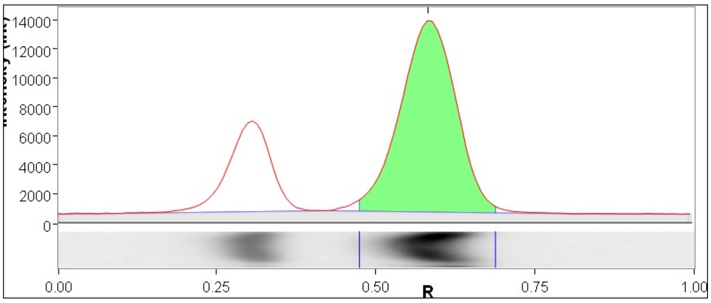

Band No.	Band Label	Mol. Wt. (KDa)	Relative Front	Adj. Volume (Int)	Volume (Int)	Abs. Quant.	Rel. Quant.	Band %	Lane %
1		N/A	0,588	6 189 152	6 938 976	N/A	N/A	100,0	72,3

Band Detection	Automatically detected bands with sensitivity: Low
Lane Background	Lane background subtracted with disk size: 10
Lane Width	4.87 mm

Lane 7

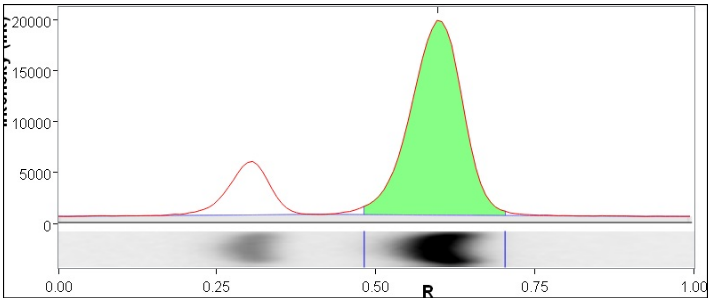

Band No.	Band Label	Mol. Wt. (KDa)	Relative Front	Adj. Volume (Int)	Volume (Int)	Abs. Quant.	Rel. Quant.	Band %	Lane %
1		N/A	0,603	8 366 816	9 123 040	N/A	N/A	100,0	80,9

Band Detection	Automatically detected bands with sensitivity: Low
Lane Background	Lane background subtracted with disk size: 10
Lane Width	4.87 mm

Lane 8

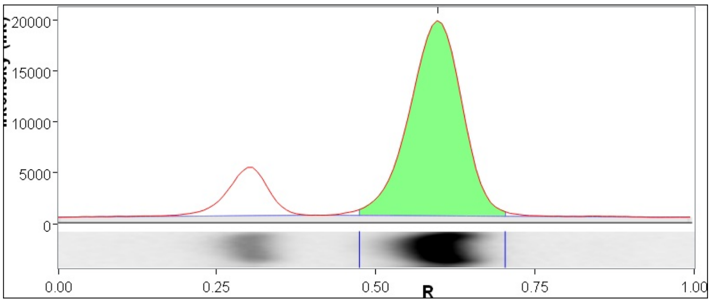

Band No.	Band Label	Mol. Wt. (KDa)	Relative Front	Adj. Volume (Int)	Volume (Int)	Abs. Quant.	Rel. Quant.	Band %	Lane %
1		N/A	0,603	9 427 936	10 239 584	N/A	N/A	100,0	82,6

Band Detection	Automatically detected bands with sensitivity: Low
Lane Background	Lane background subtracted with disk size: 10
Lane Width	4.87 mm

Lane 9

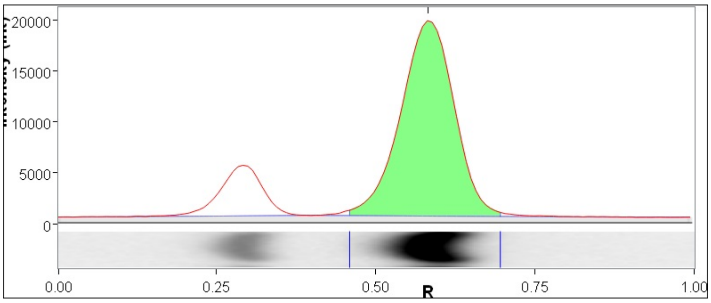

Band No.	Band Label	Mol. Wt. (KDa)	Relative Front	Adj. Volume (Int)	Volume (Int)	Abs. Quant.	Rel. Quant.	Band %	Lane %
1		N/A	0,588	9 518 688	10 328 160	N/A	N/A	100,0	82,0

Band Detection	Automatically detected bands with sensitivity: Low
Lane Background	Lane background subtracted with disk size: 10
Lane Width	4.87 mm

Lane 10

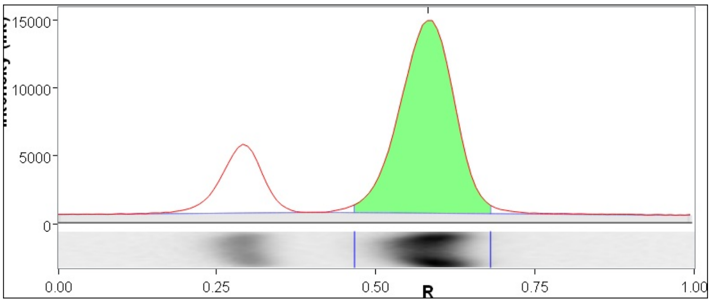

Band No.	Band Label	Mol. Wt. (KDa)	Relative Front	Adj. Volume (Int)	Volume (Int)	Abs. Quant.	Rel. Quant.	Band %	Lane %
1		N/A	0,588	6 091 189	6 753 056	N/A	N/A	100,0	77,1

Band Detection	Automatically detected bands with sensitivity: Low
Lane Background	Lane background subtracted with disk size: 10
Lane Width	4.42 mm

Lane 11

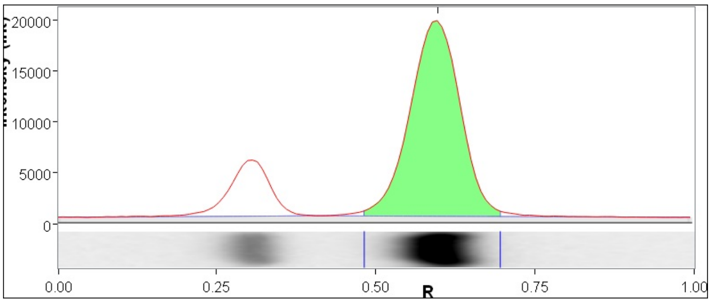

Band No.	Band Label	Mol. Wt. (KDa)	Relative Front	Adj. Volume (Int)	Volume (Int)	Abs. Quant.	Rel. Quant.	Band %	Lane %
1		N/A	0,603	8 658 300	9 347 585	N/A	N/A	100,0	79,8

Band Detection	Automatically detected bands with sensitivity: Low
Lane Background	Lane background subtracted with disk size: 10
Lane Width	4.72 mm

Lane 12

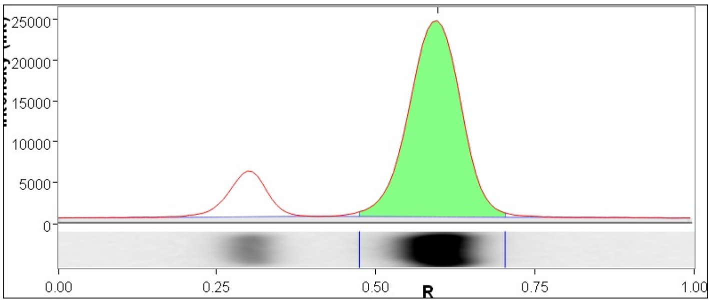

Band No.	Band Label	Mol. Wt. (KDa)	Relative Front	Adj. Volume (Int)	Volume (Int)	Abs. Quant.	Rel. Quant.	Band %	Lane %
1		N/A	0,603	10 723 200	11 508 672	N/A	N/A	100,0	84,1

Band Detection	Automatically detected bands with sensitivity: Low

Lane Background	Lane background subtracted with disk size: 10
Lane Width	4.87 mm

Lane 13

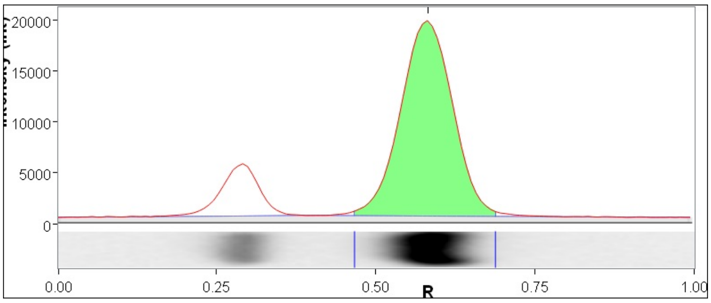

Band No.	Band Label	Mol. Wt. (KDa)	Relative Front	Adj. Volume (Int)	Volume (Int)	Abs. Quant.	Rel. Quant.	Band %	Lane %
1		N/A	0,588	9 133 248	9 882 944	N/A	N/A	100,0	82,7

Band Detection	Automatically detected bands with sensitivity: Low
Lane Background	Lane background subtracted with disk size: 10
Lane Width	4.87 mm

Image Report: Intensity analysis of succinyllysine panel in Supplementary Fig.2a, males

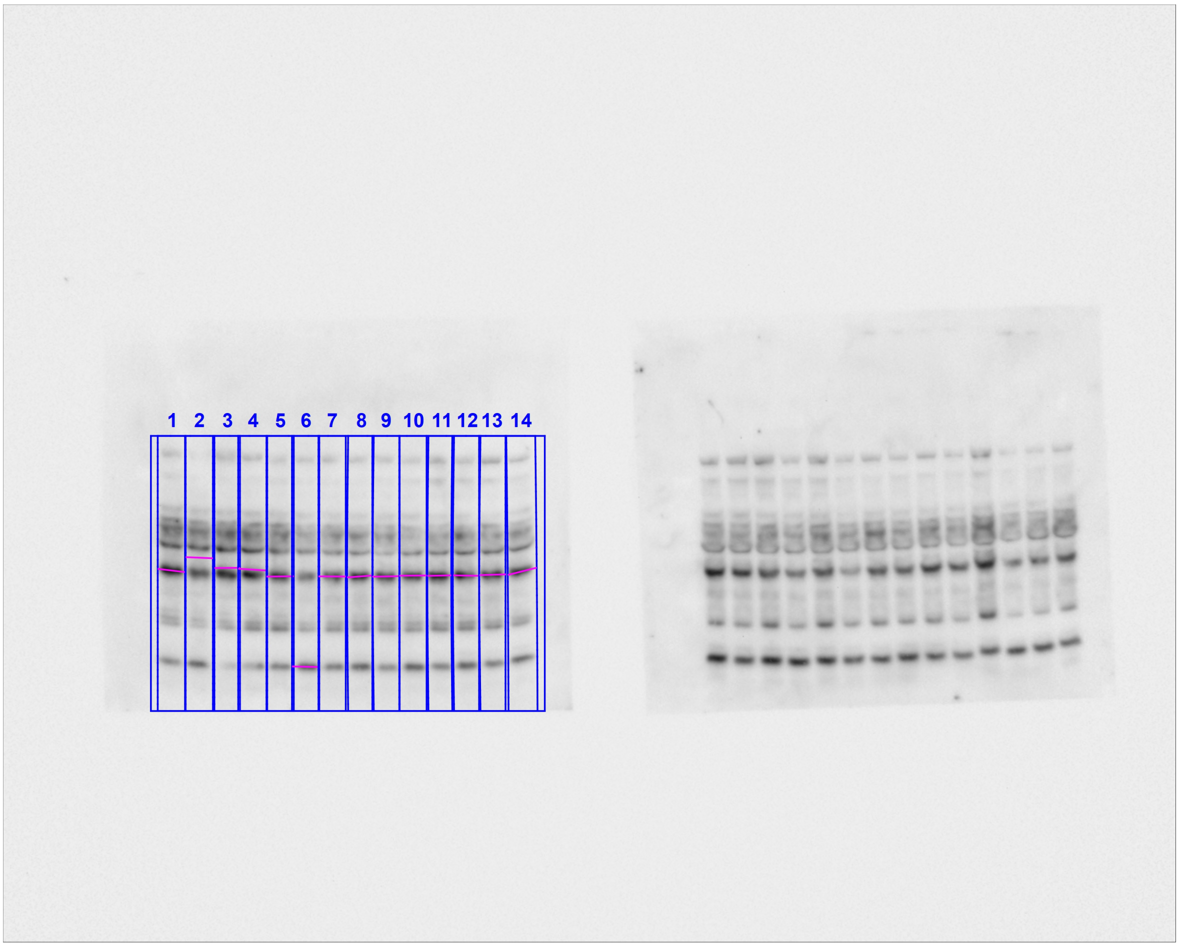

Acquisition Information

Imager	ChemiDoc MP
Exposure Time (sec)	67.110 (Signal Accumulation)
Serial Number	734BR-2876
Software Version	2.3.0.07
Application	Chemiluminescence
Excitation Source	No Illumination
Emission Filter	647SP Filter
Binning	2x2

Image Information

Acquisition Date	1/15/2021 1:42:08
User Name	Lipidlab
Image Area (mm)	X: 210.0 Y: 168.0
Pixel Size (μm)	X: 152.7 Y: 152.7

Data Range (Int)	500 - 19063

Analysis Settings

Detection	Lane detection:<br>Manually created lanes<br><br>Band detection:<br>Automatically detected bands with sensitivity: High<br>Manually adjusted bands<br><br>Lane Background Subtraction:<br>Background subtracted with different disk sizes<br><br>Lane width: Variable

Lane Statistics

Lane No.	Adj. Total Band Vol. (Int)	Total Band Vol. (Int)	Adj. Total Lane Vol. (Int)	Total Lane Vol. (Int)	Bkgd. Vol. (Int)	Norm. Factor
1	22 124 288	32 949 952	22 124 288	32 949 952	10 825 664	N/A
2	15 894 464	28 992 032	15 894 464	28 992 032	13 097 568	N/A
3	17 728 380	29 338 440	17 728 380	29 338 440	11 610 060	N/A
4	19 310 640	32 122 520	19 310 640	32 122 520	12 811 880	N/A
5	18 316 672	29 640 832	18 316 672	29 640 832	11 324 160	N/A
6	12 307 072	24 104 352	12 307 072	24 104 352	11 797 280	N/A
7	14 768 864	25 842 656	14 768 864	25 842 656	11 073 792	N/A
8	14 068 110	25 698 690	14 068 110	25 698 690	11 630 580	N/A
9	14 599 904	25 647 040	14 599 904	25 647 040	11 047 136	N/A
10	14 692 288	26 143 808	14 692 288	26 143 808	11 451 520	N/A
11	16 340 352	25 774 728	16 340 352	25 774 728	9 434 376	N/A
12	16 769 636	28 283 098	16 769 636	28 283 098	11 513 462	N/A
13	16 834 519	26 031 165	16 834 519	26 031 165	9 196 646	N/A
14	15 264 164	24 865 118	15 264 164	24 865 118	9 600 954	N/A

Lane And Band Analysis

Lane 1

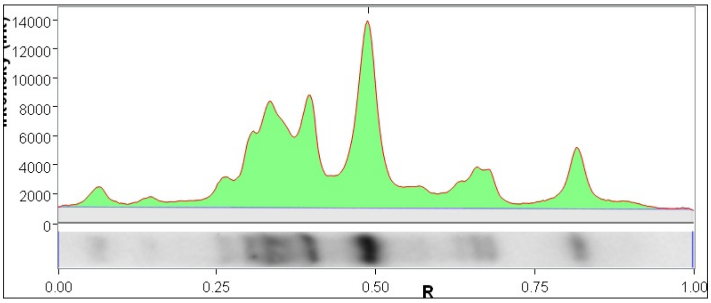

Band No.	Band Label	Mol. Wt. (KDa)	Relative Front	Adj. Volume (Int)	Volume (Int)	Abs. Quant.	Rel. Quant.	Band %	Lane %
1		N/A	0,489	22 124 288	32 949 952	N/A	N/A	100,0	100,0

Band Detection	Automatically detected bands with sensitivity: High
Lane Background	Lane background subtracted with disk size: 44.3
Lane Width	4.89 mm

Lane 2

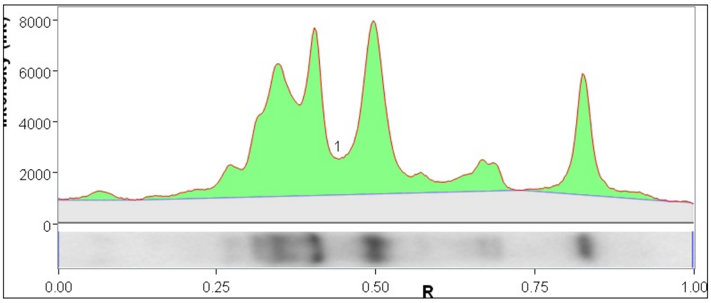

Band No.	Band Label	Mol. Wt. (KDa)	Relative Front	Adj. Volume (Int)	Volume (Int)	Abs. Quant.	Rel. Quant.	Band %	Lane %
1		N/A	0,443	15 894 464	28 992 032	N/A	N/A	100,0	100,0

Band Detection	Automatically detected bands with sensitivity: High
Lane Background	Lane background subtracted with disk size: 30.9
Lane Width	4.89 mm

Lane 3

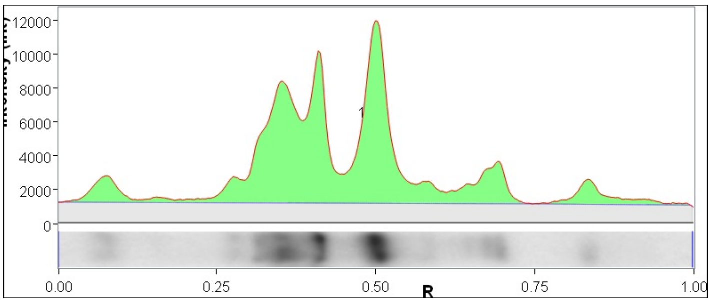

Band No.	Band Label	Mol. Wt. (KDa)	Relative Front	Adj. Volume (Int)	Volume (Int)	Abs. Quant.	Rel. Quant.	Band %	Lane %
1		N/A	0,480	17 728 380	29 338 440	N/A	N/A	100,0	100,0

Band Detection	Automatically detected bands with sensitivity: High
Lane Background	Lane background subtracted with disk size: 49.1
Lane Width	4.58 mm

Lane 4

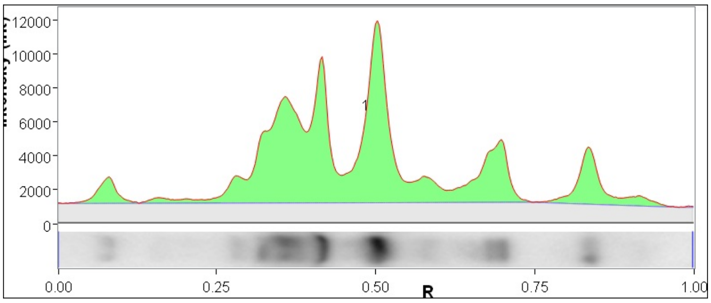

Band No.	Band Label	Mol. Wt. (KDa)	Relative Front	Adj. Volume (Int)	Volume (Int)	Abs. Quant.	Rel. Quant.	Band %	Lane %
1		N/A	0,486	19 310 640	32 122 520	N/A	N/A	100,0	100,0

Band Detection	Automatically detected bands with sensitivity: High

Lane Background	Lane background subtracted with disk size: 28
Lane Width	5.19 mm

Lane 5

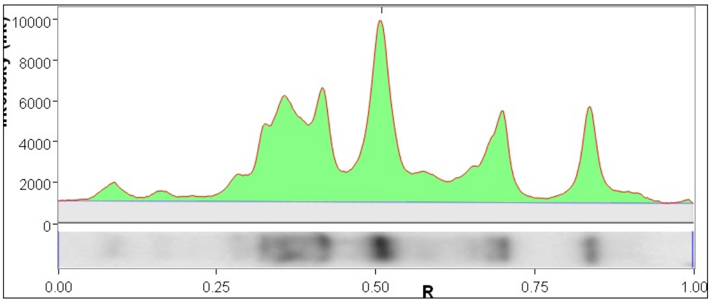

Band No.	Band Label	Mol. Wt. (KDa)	Relative Front	Adj. Volume (Int)	Volume (Int)	Abs. Quant.	Rel. Quant.	Band %	Lane %
1		N/A	0,511	18 316 672	29 640 832	N/A	N/A	100,0	100,0

Band Detection	Automatically detected bands with sensitivity: High
Lane Background	Lane background subtracted with disk size: 38.6
Lane Width	4.89 mm

Lane 6

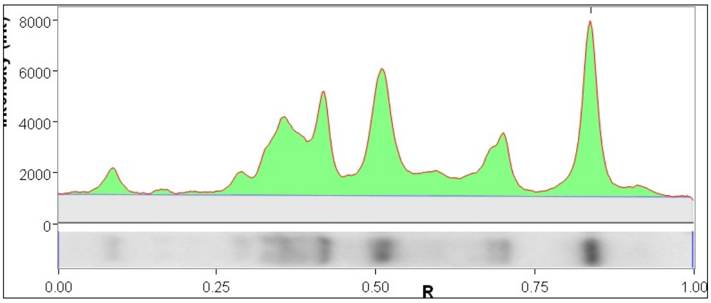

Band No.	Band Label	Mol. Wt. (KDa)	Relative Front	Adj. Volume (Int)	Volume (Int)	Abs. Quant.	Rel. Quant.	Band %	Lane %
1		N/A	0,839	12 307 072	24 104 352	N/A	N/A	100,0	100,0

Band Detection	Automatically detected bands with sensitivity: High
Lane Background	Lane background subtracted with disk size: 43.4
Lane Width	4.89 mm

Lane 7

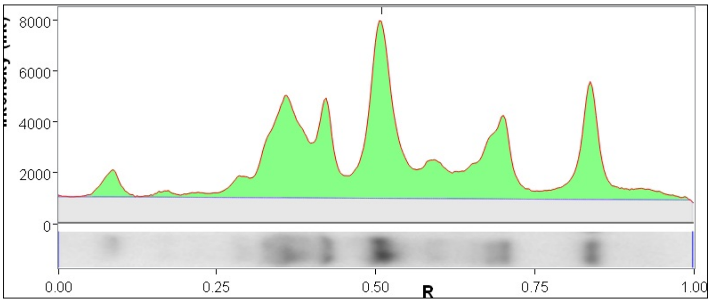

Band No.	Band Label	Mol. Wt. (KDa)	Relative Front	Adj. Volume (Int)	Volume (Int)	Abs. Quant.	Rel. Quant.	Band %	Lane %
1		N/A	0,511	14 768 864	25 842 656	N/A	N/A	100,0	100,0

Band Detection	Automatically detected bands with sensitivity: High
Lane Background	Lane background subtracted with disk size: 47.2
Lane Width	4.89 mm

Lane 8

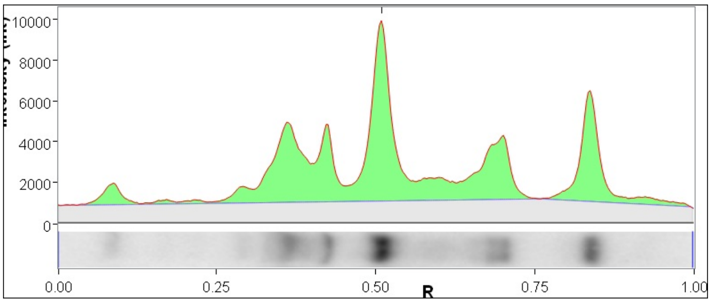

Band No.	Band Label	Mol. Wt. (KDa)	Relative Front	Adj. Volume (Int)	Volume (Int)	Abs. Quant.	Rel. Quant.	Band %	Lane %
1		N/A	0,511	14 068 110	25 698 690	N/A	N/A	100,0	100,0

Band Detection	Automatically detected bands with sensitivity: High
Lane Background	Lane background subtracted with disk size: 29.9
Lane Width	4.58 mm

Lane 9

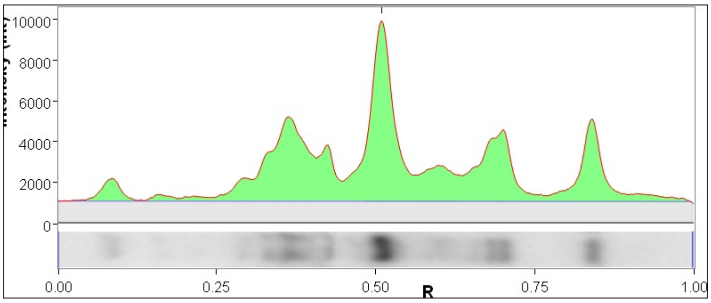

Band No.	Band Label	Mol. Wt. (KDa)	Relative Front	Adj. Volume (Int)	Volume (Int)	Abs. Quant.	Rel. Quant.	Band %	Lane %
1		N/A	0,511	14 599 904	25 647 040	N/A	N/A	100,0	100,0

Band Detection	Automatically detected bands with sensitivity: High
Lane Background	Lane background subtracted with disk size: 42.4
Lane Width	4.89 mm

Lane 10

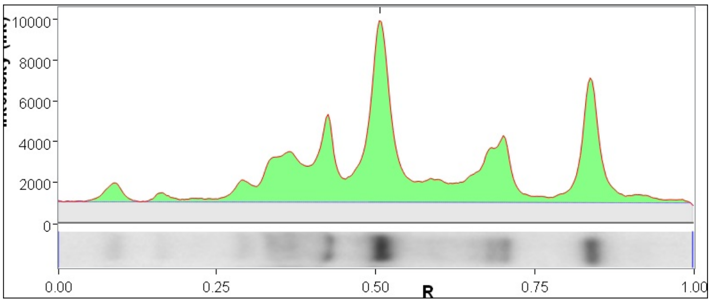

Band No.	Band Label	Mol. Wt. (KDa)	Relative Front	Adj. Volume (Int)	Volume (Int)	Abs. Quant.	Rel. Quant.	Band %	Lane %
1		N/A	0,508	14 692 288	26 143 808	N/A	N/A	100,0	100,0

Band Detection	Automatically detected bands with sensitivity: High
Lane Background	Lane background subtracted with disk size: 41.4
Lane Width	4.89 mm

Lane 11

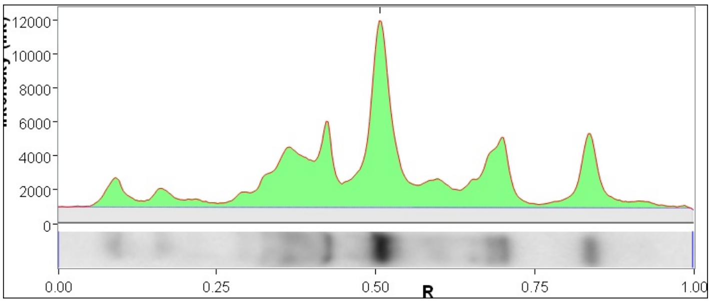

Band No.	Band Label	Mol. Wt. (KDa)	Relative Front	Adj. Volume (Int)	Volume (Int)	Abs. Quant.	Rel. Quant.	Band %	Lane %
1		N/A	0,508	16 340 352	25 774 728	N/A	N/A	100,0	100,0

Band Detection	Automatically detected bands with sensitivity: High
Lane Background	Lane background subtracted with disk size: 66.4
Lane Width	4.28 mm

Lane 12

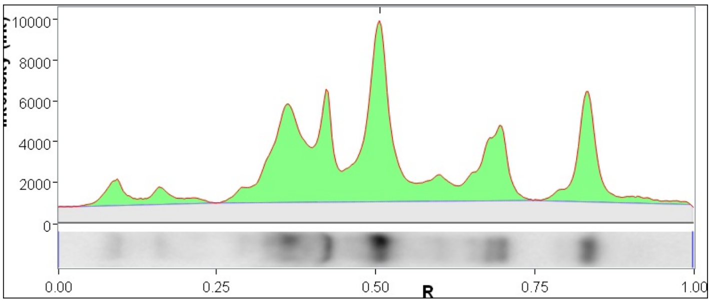

Band No.	Band Label	Mol. Wt. (KDa)	Relative Front	Adj. Volume (Int)	Volume (Int)	Abs. Quant.	Rel. Quant.	Band %	Lane %
1		N/A	0,508	16 769 636	28 283 098	N/A	N/A	100,0	100,0

Band Detection	Automatically detected bands with sensitivity: High

Lane Background	Lane background subtracted with disk size: 32.8
Lane Width	4.73 mm

Lane 13

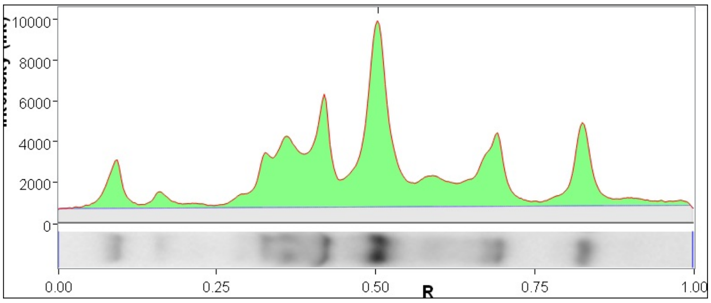

Band No.	Band Label	Mol. Wt. (KDa)	Relative Front	Adj. Volume (Int)	Volume (Int)	Abs. Quant.	Rel. Quant.	Band %	Lane %
1		N/A	0,505	16 834 519	26 031 165	N/A	N/A	100,0	100,0

Band Detection	Automatically detected bands with sensitivity: High
Lane Background	Lane background subtracted with disk size: 53.9
Lane Width	4.73 mm

Lane 14

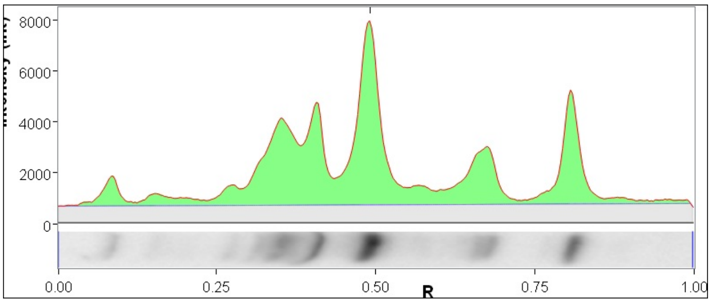

Band No.	Band Label	Mol. Wt. (KDa)	Relative Front	Adj. Volume (Int)	Volume (Int)	Abs. Quant.	Rel. Quant.	Band %	Lane %
1		N/A	0,492	15 264 164	24 865 118	N/A	N/A	100,0	100,0

Band Detection	Automatically detected bands with sensitivity: High
Lane Background	Lane background subtracted with disk size: 53.9
Lane Width	5.19 mm

Image Report: Intensity analysis of Gapdh panel in Supplementary Fig. 2a, males

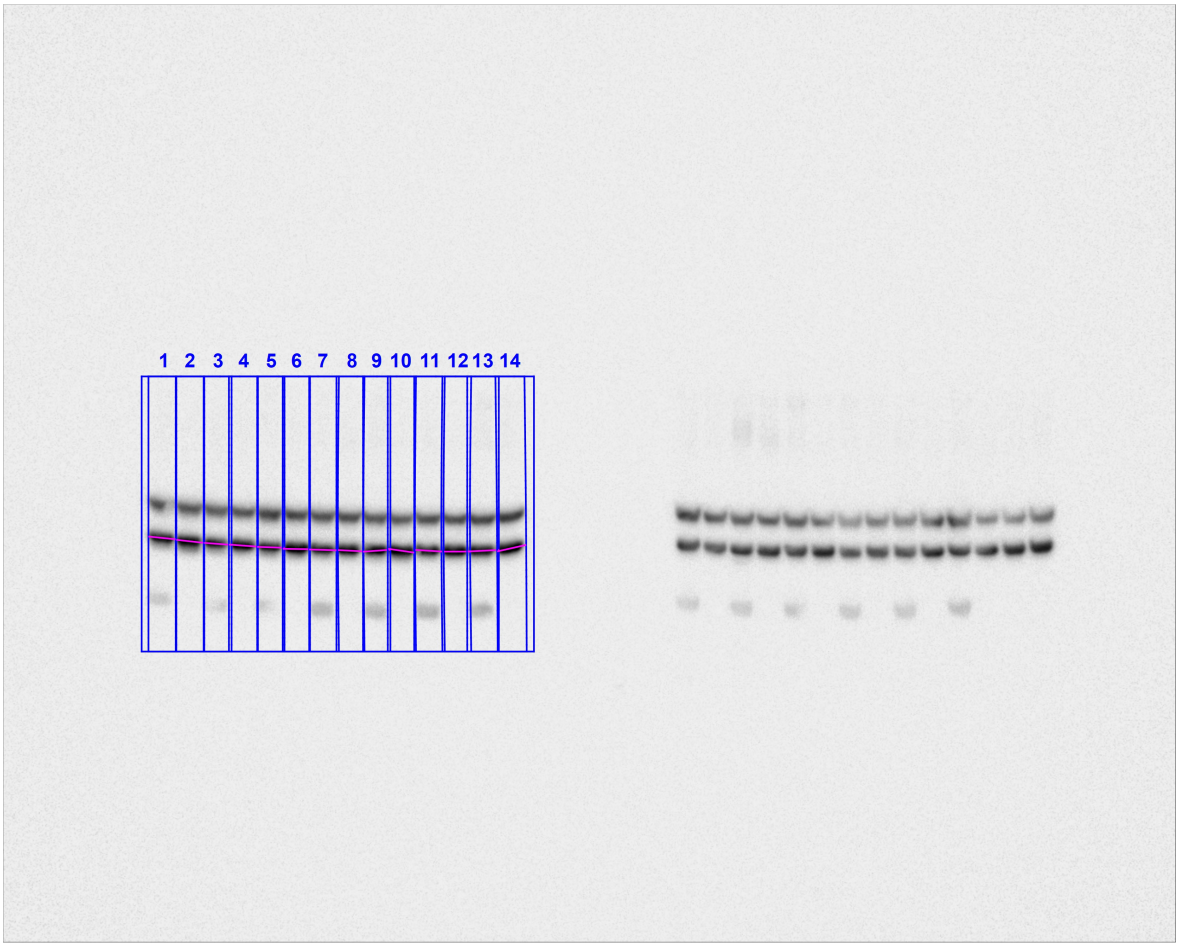

Acquisition Information

Imager	ChemiDoc MP
Exposure Time (sec)	9.987 (Signal Accumulation)
Serial Number	734BR-2876
Software Version	2.3.0.07
Application	Chemiluminescence
Excitation Source	No Illumination
Emission Filter	647SP Filter
Binning	2x2

Image Information

Acquisition Date	1/16/2021 6:18:28
User Name	Lipidlab
Image Area (mm)	X: 210.0 Y: 168.0
Pixel Size (µm)	X: 152.7 Y: 152.7
Data Range (Int)	500 - 25287

Analysis Settings

Detection	Lane detection:<br>Manually created lanes (Copied)<br><br>Band detection:<br>Automatically detected bands with sensitivity: Low<br>Manually adjusted bands<br><br>Lane Background Subtraction:<br>Lane background subtracted with disk size: 10.1<br><br>Lane width: Variable

Lane Statistics

Lane No.	Adj. Total Band Vol. (Int)	Total Band Vol. (Int)	Adj. Total Lane Vol. (Int)	Total Lane Vol. (Int)	Bkgd. Vol. (Int)	Norm. Factor
1	6 712 832	7 803 552	11 838 592	19 415 424	7 576 832	N/A
2	7 866 560	9 076 352	13 141 120	20 928 384	7 787 264	N/A
3	5 865 280	6 893 760	11 317 920	19 029 312	7 711 392	N/A
4	7 342 674	8 387 120	12 610 566	20 548 886	7 938 320	N/A
5	5 953 856	6 995 392	12 514 304	20 132 256	7 617 952	N/A
6	7 926 496	9 006 880	13 342 240	20 929 888	7 587 648	N/A
7	6 162 400	7 215 552	12 845 696	20 414 080	7 568 384	N/A
8	6 456 330	7 430 100	11 653 080	18 644 970	6 991 890	N/A
9	6 879 072	8 016 576	13 221 760	21 097 632	7 875 872	N/A
10	7 702 911	8 827 250	12 173 390	19 657 534	7 484 144	N/A
11	5 827 876	6 824 123	12 367 202	19 646 715	7 279 513	N/A
12	7 232 052	8 251 208	12 171 065	19 405 938	7 234 873	N/A
13	7 239 272	8 453 945	14 928 390	23 817 455	8 889 065	N/A
14	7 740 372	8 812 528	13 626 112	21 472 530	7 846 418	N/A

Lane And Band Analysis

Lane 1

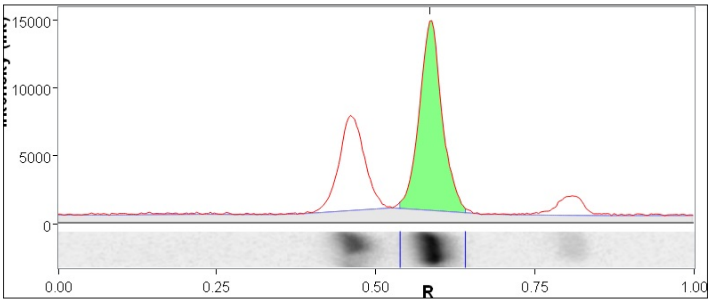

Band No.	Band Label	Mol. Wt. (KDa)	Relative Front	Adj. Volume (Int)	Volume (Int)	Abs. Quant.	Rel. Quant.	Band %	Lane %
1		N/A	0,587	6 712 832	7 803 552	N/A	N/A	100,0	56,7

Band Detection	Automatically detected bands with sensitivity: Low
Lane Background	Lane background subtracted with disk size: 10.1
Lane Width	4.89 mm

Lane 2

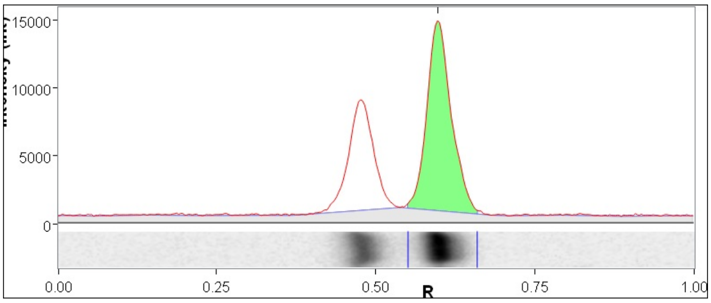

Band No.	Band Label	Mol. Wt. (KDa)	Relative Front	Adj. Volume (Int)	Volume (Int)	Abs. Quant.	Rel. Quant.	Band %	Lane %
1		N/A	0,599	7 866 560	9 076 352	N/A	N/A	100,0	59,9

Band Detection	Automatically detected bands with sensitivity: Low
Lane Background	Lane background subtracted with disk size: 10.1
Lane Width	4.89 mm

Lane 3

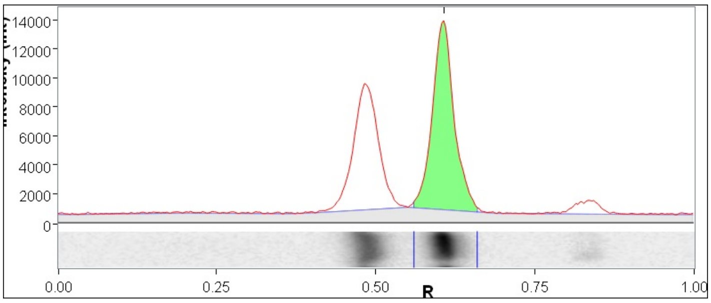

Band No.	Band Label	Mol. Wt. (KDa)	Relative Front	Adj. Volume (Int)	Volume (Int)	Abs. Quant.	Rel. Quant.	Band %	Lane %
1		N/A	0,609	5 865 280	6 893 760	N/A	N/A	100,0	51,8

Band Detection	Automatically detected bands with sensitivity: Low
Lane Background	Lane background subtracted with disk size: 10.1
Lane Width	4.89 mm

Lane 4

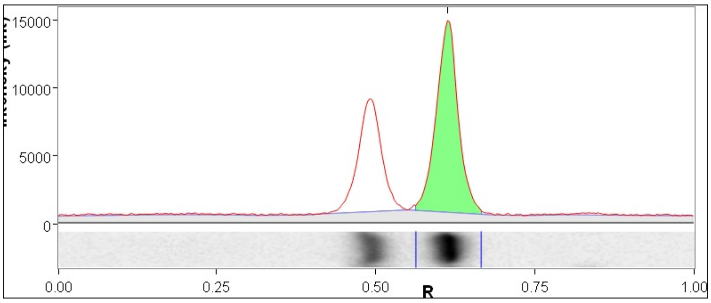

Band No.	Band Label	Mol. Wt. (KDa)	Relative Front	Adj. Volume (Int)	Volume (Int)	Abs. Quant.	Rel. Quant.	Band %	Lane %
1		N/A	0,615	7 342 674	8 387 120	N/A	N/A	100,0	58,2

Band Detection	Automatically detected bands with sensitivity: Low

Lane Background	Lane background subtracted with disk size: 10.1
Lane Width	5.19 mm

Lane 5

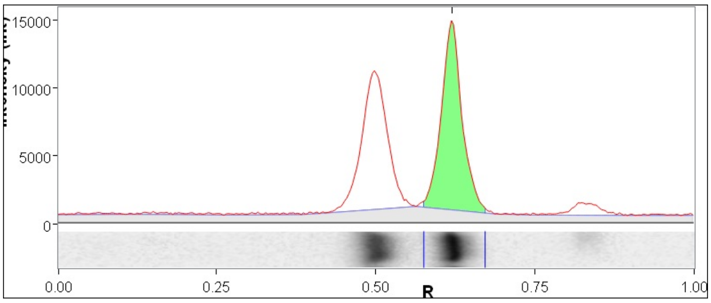

Band No.	Band Label	Mol. Wt. (KDa)	Relative Front	Adj. Volume (Int)	Volume (Int)	Abs. Quant.	Rel. Quant.	Band %	Lane %
1		N/A	0,621	5 953 856	6 995 392	N/A	N/A	100,0	47,6

Band Detection	Automatically detected bands with sensitivity: Low
Lane Background	Lane background subtracted with disk size: 10.1
Lane Width	4.89 mm

Lane 6

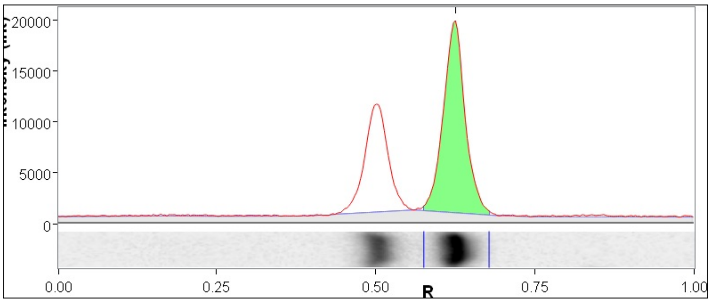

Band No.	Band Label	Mol. Wt. (KDa)	Relative Front	Adj. Volume (Int)	Volume (Int)	Abs. Quant.	Rel. Quant.	Band %	Lane %
1		N/A	0,627	7 926 496	9 006 880	N/A	N/A	100,0	59,4

Band Detection	Automatically detected bands with sensitivity: Low
Lane Background	Lane background subtracted with disk size: 10.1
Lane Width	4.89 mm

Lane 7

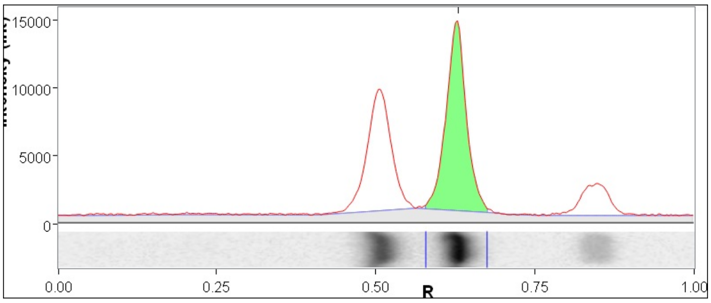

Band No.	Band Label	Mol. Wt. (KDa)	Relative Front	Adj. Volume (Int)	Volume (Int)	Abs. Quant.	Rel. Quant.	Band %	Lane %
1		N/A	0,630	6 162 400	7 215 552	N/A	N/A	100,0	48,0

Band Detection	Automatically detected bands with sensitivity: Low
Lane Background	Lane background subtracted with disk size: 10.1
Lane Width	4.89 mm

Lane 8

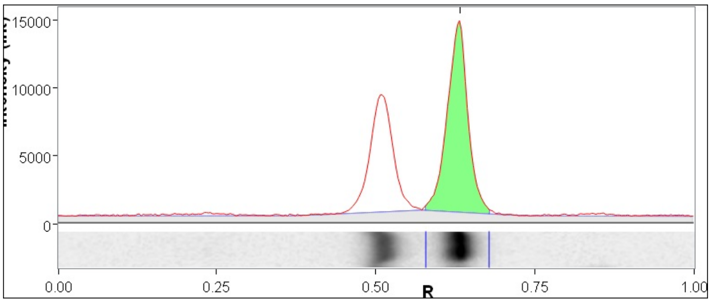

Band No.	Band Label	Mol. Wt. (KDa)	Relative Front	Adj. Volume (Int)	Volume (Int)	Abs. Quant.	Rel. Quant.	Band %	Lane %
1		N/A	0,634	6 456 330	7 430 100	N/A	N/A	100,0	55,4

Band Detection	Automatically detected bands with sensitivity: Low
Lane Background	Lane background subtracted with disk size: 10.1
Lane Width	4.58 mm

Lane 9

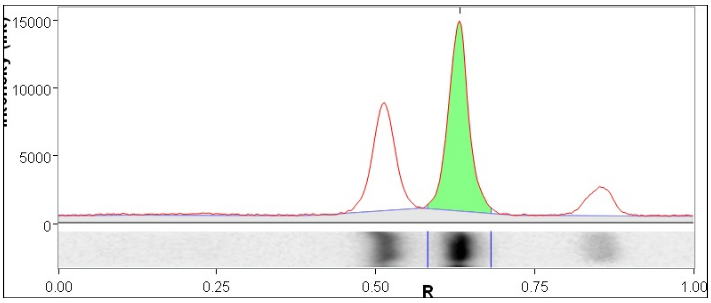

Band No.	Band Label	Mol. Wt. (KDa)	Relative Front	Adj. Volume (Int)	Volume (Int)	Abs. Quant.	Rel. Quant.	Band %	Lane %
1		N/A	0,634	6 879 072	8 016 576	N/A	N/A	100,0	52,0

Band Detection	Automatically detected bands with sensitivity: Low
Lane Background	Lane background subtracted with disk size: 10.1
Lane Width	4.89 mm

Lane 10

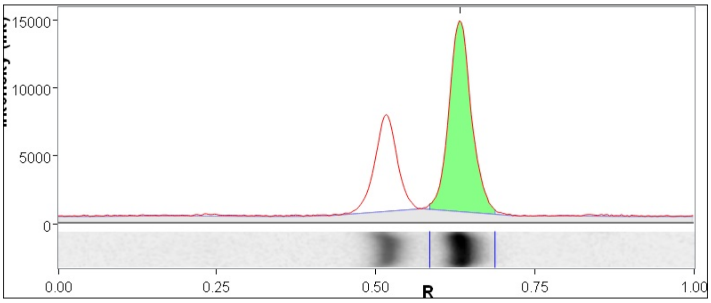

Band No.	Band Label	Mol. Wt. (KDa)	Relative Front	Adj. Volume (Int)	Volume (Int)	Abs. Quant.	Rel. Quant.	Band %	Lane %
1		N/A	0,634	7 702 911	8 827 250	N/A	N/A	100,0	63,3

Band Detection	Automatically detected bands with sensitivity: Low
Lane Background	Lane background subtracted with disk size: 10.1
Lane Width	4.73 mm

Lane 11

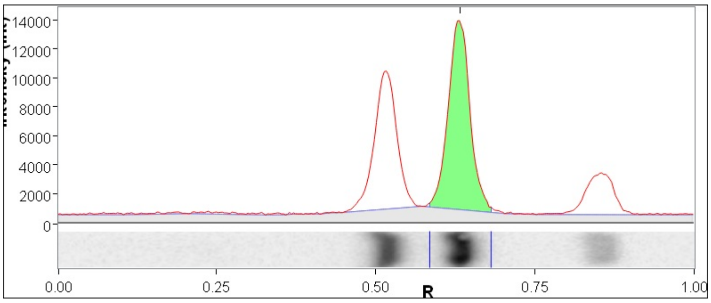

Band No.	Band Label	Mol. Wt. (KDa)	Relative Front	Adj. Volume (Int)	Volume (Int)	Abs. Quant.	Rel. Quant.	Band %	Lane %
1		N/A	0,634	5 827 876	6 824 123	N/A	N/A	100,0	47,1

Band Detection	Automatically detected bands with sensitivity: Low
Lane Background	Lane background subtracted with disk size: 10.1
Lane Width	4.73 mm

Lane 12

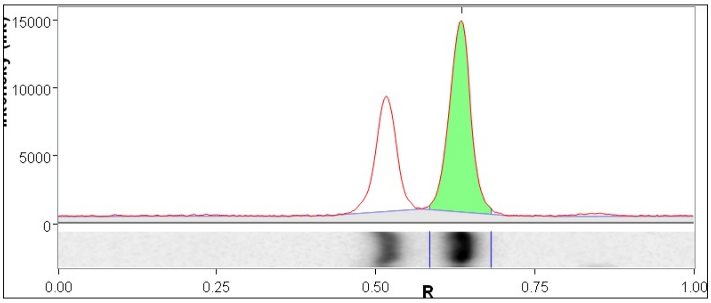

Band No.	Band Label	Mol. Wt. (KDa)	Relative Front	Adj. Volume (Int)	Volume (Int)	Abs. Quant.	Rel. Quant.	Band %	Lane %
1		N/A	0,637	7 232 052	8 251 208	N/A	N/A	100,0	59,4

Band Detection	Automatically detected bands with sensitivity: Low

Lane Background	Lane background subtracted with disk size: 10.1
Lane Width	4.73 mm

Lane 13

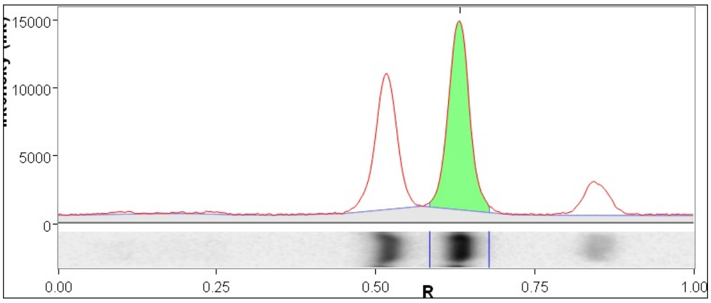

Band No.	Band Label	Mol. Wt. (KDa)	Relative Front	Adj. Volume (Int)	Volume (Int)	Abs. Quant.	Rel. Quant.	Band %	Lane %
1		N/A	0,634	7 239 272	8 453 945	N/A	N/A	100,0	48,5

Band Detection	Automatically detected bands with sensitivity: Low
Lane Background	Lane background subtracted with disk size: 10.1
Lane Width	5.65 mm

Lane 14

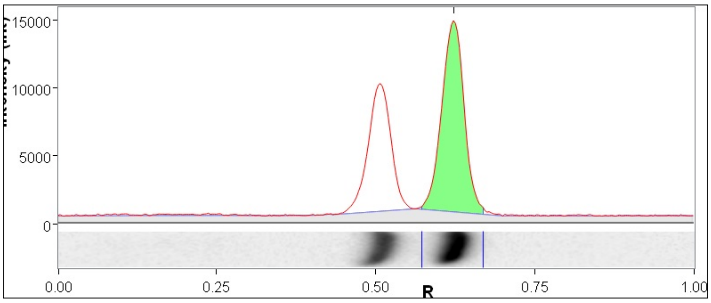

Band No.	Band Label	Mol. Wt. (KDa)	Relative Front	Adj. Volume (Int)	Volume (Int)	Abs. Quant.	Rel. Quant.	Band %	Lane %
1		N/A	0,624	7 740 372	8 812 528	N/A	N/A	100,0	56,8

Band Detection	Automatically detected bands with sensitivity: Low
Lane Background	Lane background subtracted with disk size: 10.1
Lane Width	5.19 mm

Image Report: Intensity analysis of succinyllysine panel in Supplementary Fig. 2a, females

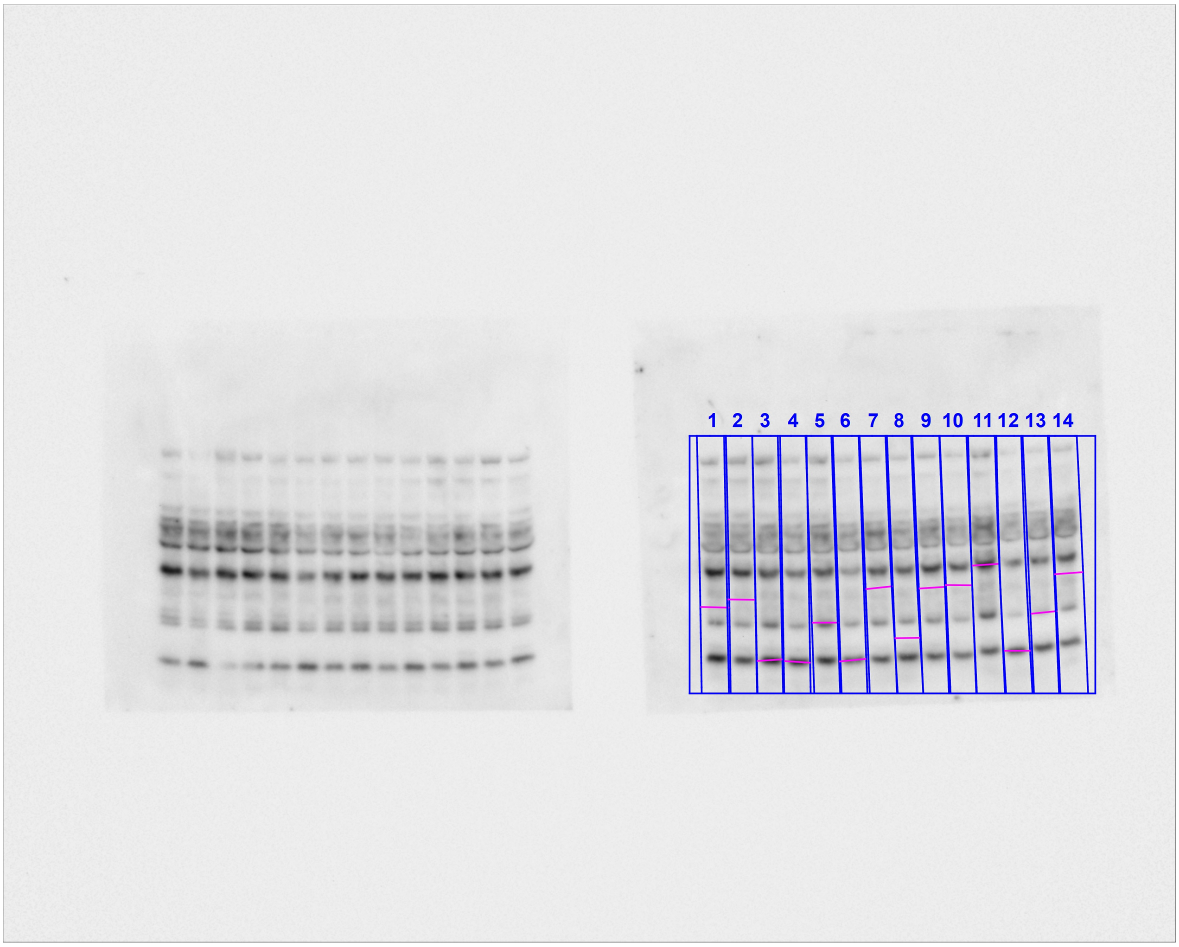

Acquisition Information

Imager	ChemiDoc MP
Exposure Time (sec)	67.110 (Signal Accumulation)
Serial Number	734BR-2876
Software Version	2.3.0.07
Application	Chemiluminescence
Excitation Source	No Illumination
Emission Filter	647SP Filter
Binning	2x2

Image Information

Acquisition Date	1/15/2021 1:42:08
User Name	Lipidlab
Image Area (mm)	X: 210.0 Y: 168.0
Pixel Size (µm)	X: 152.7 Y: 152.7

Data Range (Int)	500 - 19063

Analysis Settings

Detection	Lane detection:<br>Manually created lanes<br><br>Band detection:<br>Automatically detected bands with sensitivity: Low<br>Manually adjusted bands<br><br>Lane Background Subtraction:<br>Background subtracted with different disk sizes<br><br>Lane width: Variable

Lane Statistics

Lane No.	Adj. Total Band Vol. (Int)	Total Band Vol. (Int)	Adj. Total Lane Vol. (Int)	Total Lane Vol. (Int)	Bkgd. Vol. (Int)	Norm. Factor
1	18 793 602	32 444 024	18 793 602	32 444 024	13 650 422	N/A
2	15 673 944	30 038 415	15 673 944	30 038 415	14 364 471	N/A
3	17 727 990	30 942 180	17 727 990	30 942 180	13 214 190	N/A
4	12 405 797	25 969 537	12 405 797	25 969 537	13 563 740	N/A
5	17 064 320	31 484 032	17 064 320	31 484 032	14 419 712	N/A
6	11 669 152	23 568 800	11 669 152	23 568 800	11 899 648	N/A
7	15 401 600	27 324 288	15 401 600	27 324 288	11 922 688	N/A
8	14 448 030	25 271 040	14 448 030	25 271 040	10 823 010	N/A
9	16 005 760	26 816 224	16 005 760	26 816 224	10 810 464	N/A
10	12 115 136	23 053 696	12 115 136	23 053 696	10 938 560	N/A
11	21 532 236	33 596 904	21 532 236	33 596 904	12 064 668	N/A
12	10 339 833	20 372 456	10 339 833	20 372 456	10 032 623	N/A
13	12 912 120	22 540 193	12 912 120	22 540 193	9 628 073	N/A
14	15 705 790	26 095 646	15 705 790	26 095 646	10 389 856	N/A

Lane And Band Analysis

Lane 1

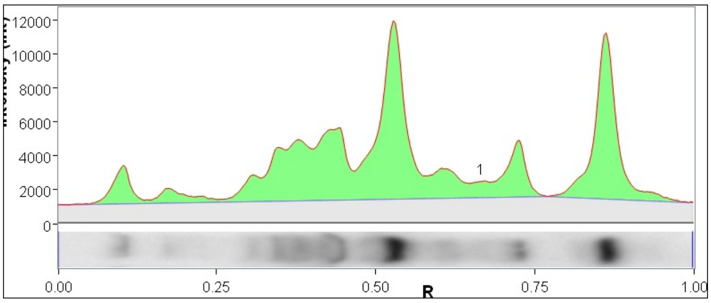

Band No.	Band Label	Mol. Wt. (KDa)	Relative Front	Adj. Volume (Int)	Volume (Int)	Abs. Quant.	Rel. Quant.	Band %	Lane %
1		N/A	0,666	18 793 602	32 444 024	N/A	N/A	100,0	100,0

Band Detection	Automatically detected bands with sensitivity: Low
Lane Background	Lane background subtracted with disk size: 34.7
Lane Width	5.19 mm

Lane 2

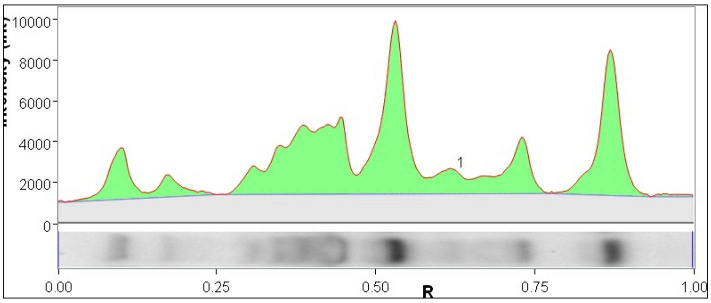

Band No.	Band Label	Mol. Wt. (KDa)	Relative Front	Adj. Volume (Int)	Volume (Int)	Abs. Quant.	Rel. Quant.	Band %	Lane %
1		N/A	0,636	15 673 944	30 038 415	N/A	N/A	100,0	100,0

Band Detection	Automatically detected bands with sensitivity: Low
Lane Background	Lane background subtracted with disk size: 25.1
Lane Width	5.04 mm

Lane 3

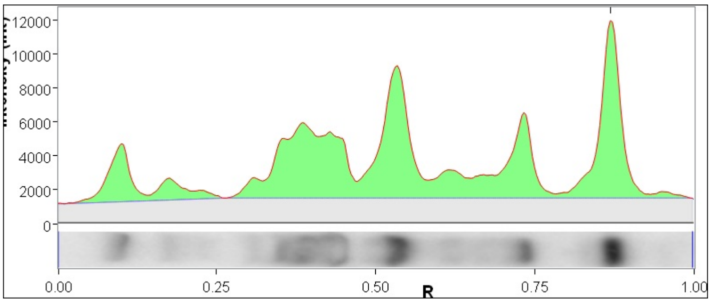

Band No.	Band Label	Mol. Wt. (KDa)	Relative Front	Adj. Volume (Int)	Volume (Int)	Abs. Quant.	Rel. Quant.	Band %	Lane %
1		N/A	0,871	17 727 990	30 942 180	N/A	N/A	100,0	100,0

Band Detection	Automatically detected bands with sensitivity: Low
Lane Background	Lane background subtracted with disk size: 34.7
Lane Width	4.58 mm

Lane 4

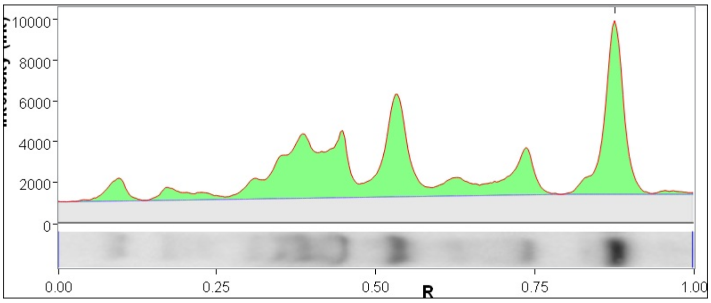

Band No.	Band Label	Mol. Wt. (KDa)	Relative Front	Adj. Volume (Int)	Volume (Int)	Abs. Quant.	Rel. Quant.	Band %	Lane %
1		N/A	0,877	12 405 797	25 969 537	N/A	N/A	100,0	100,0

Band Detection	Automatically detected bands with sensitivity: Low

Lane Background	Lane background subtracted with disk size: 38.6
Lane Width	4.73 mm

Lane 5

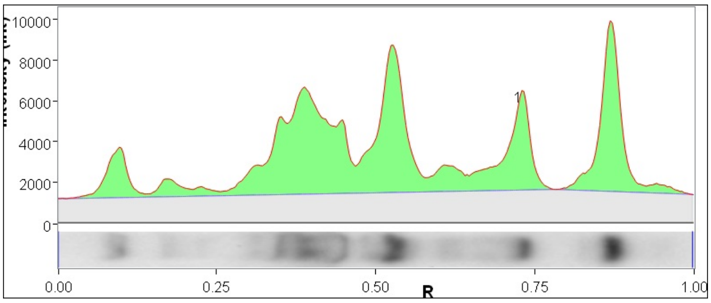

Band No.	Band Label	Mol. Wt. (KDa)	Relative Front	Adj. Volume (Int)	Volume (Int)	Abs. Quant.	Rel. Quant.	Band %	Lane %
1		N/A	0,725	17 064 320	31 484 032	N/A	N/A	100,0	100,0

Band Detection	Automatically detected bands with sensitivity: Low
Lane Background	Lane background subtracted with disk size: 35.7
Lane Width	4.89 mm

Lane 6

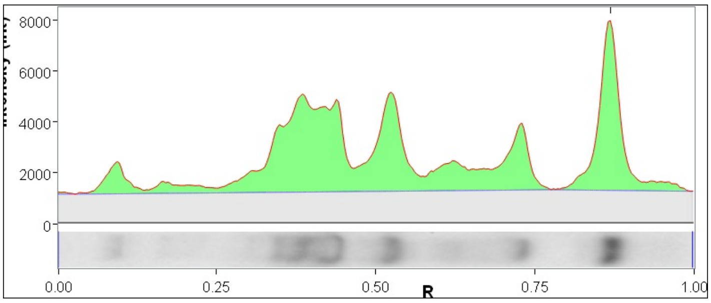

Band No.	Band Label	Mol. Wt. (KDa)	Relative Front	Adj. Volume (Int)	Volume (Int)	Abs. Quant.	Rel. Quant.	Band %	Lane %
1		N/A	0,871	11 669 152	23 568 800	N/A	N/A	100,0	100,0

Band Detection	Automatically detected bands with sensitivity: Low
Lane Background	Lane background subtracted with disk size: 34.7
Lane Width	4.89 mm

Lane 7

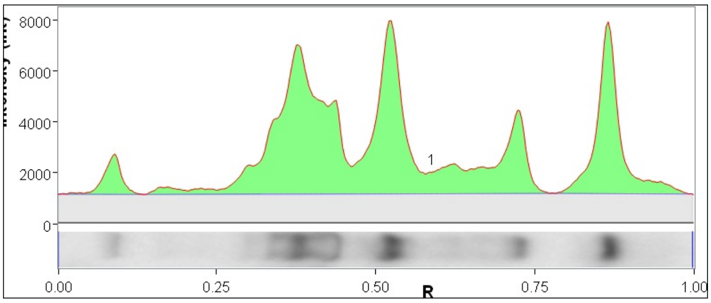

Band No.	Band Label	Mol. Wt. (KDa)	Relative Front	Adj. Volume (Int)	Volume (Int)	Abs. Quant.	Rel. Quant.	Band %	Lane %
1		N/A	0,589	15 401 600	27 324 288	N/A	N/A	100,0	100,0

Band Detection	Automatically detected bands with sensitivity: Low
Lane Background	Lane background subtracted with disk size: 39.5
Lane Width	4.89 mm

Lane 8

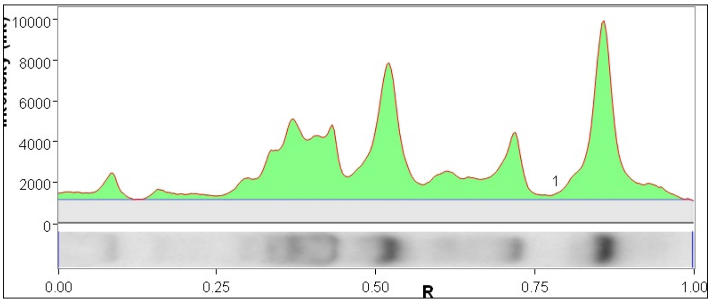

Band No.	Band Label	Mol. Wt. (KDa)	Relative Front	Adj. Volume (Int)	Volume (Int)	Abs. Quant.	Rel. Quant.	Band %	Lane %
1		N/A	0,785	14 448 030	25 271 040	N/A	N/A	100,0	100,0

Band Detection	Automatically detected bands with sensitivity: Low
Lane Background	Lane background subtracted with disk size: 38.6
Lane Width	4.58 mm

Lane 9

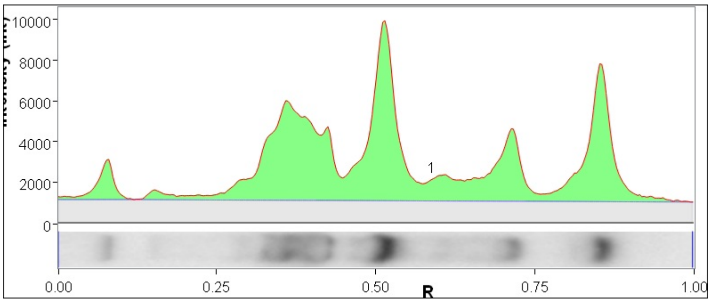

Band No.	Band Label	Mol. Wt. (KDa)	Relative Front	Adj. Volume (Int)	Volume (Int)	Abs. Quant.	Rel. Quant.	Band %	Lane %
1		N/A	0,589	16 005 760	26 816 224	N/A	N/A	100,0	100,0

Band Detection	Automatically detected bands with sensitivity: Low
Lane Background	Lane background subtracted with disk size: 40.5
Lane Width	4.89 mm

Lane 10

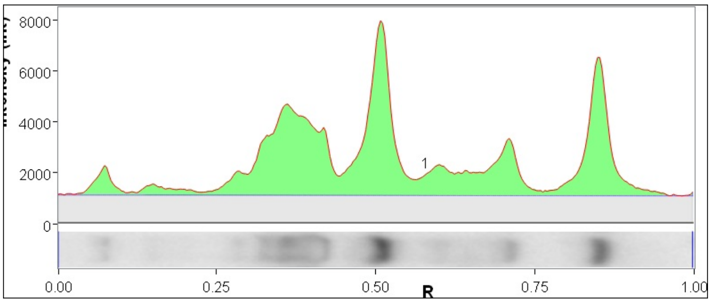

Band No.	Band Label	Mol. Wt. (KDa)	Relative Front	Adj. Volume (Int)	Volume (Int)	Abs. Quant.	Rel. Quant.	Band %	Lane %
1		N/A	0,579	12 115 136	23 053 696	N/A	N/A	100,0	100,0

Band Detection	Automatically detected bands with sensitivity: Low
Lane Background	Lane background subtracted with disk size: 36.6
Lane Width	4.89 mm

Lane 11

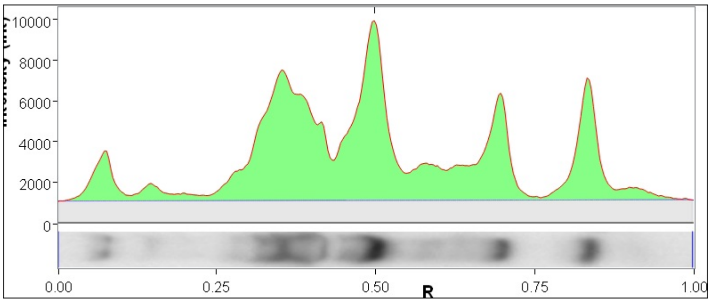

Band No.	Band Label	Mol. Wt. (KDa)	Relative Front	Adj. Volume (Int)	Volume (Int)	Abs. Quant.	Rel. Quant.	Band %	Lane %
1		N/A	0,500	21 532 236	33 596 904	N/A	N/A	100,0	100,0

Band Detection	Automatically detected bands with sensitivity: Low
Lane Background	Lane background subtracted with disk size: 41.4
Lane Width	5.04 mm

Lane 12

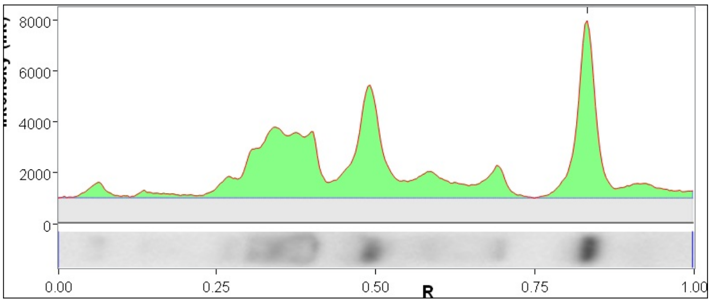

Band No.	Band Label	Mol. Wt. (KDa)	Relative Front	Adj. Volume (Int)	Volume (Int)	Abs. Quant.	Rel. Quant.	Band %	Lane %
1		N/A	0,834	10 339 833	20 372 456	N/A	N/A	100,0	100,0

Band Detection	Automatically detected bands with sensitivity: Low

Lane Background	Lane background subtracted with disk size: 54.9
Lane Width	4.73 mm

Lane 13

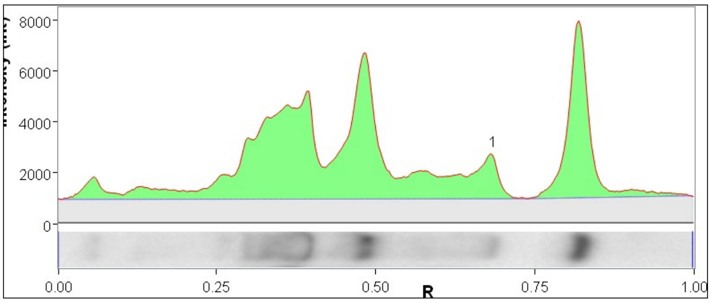

Band No.	Band Label	Mol. Wt. (KDa)	Relative Front	Adj. Volume (Int)	Volume (Int)	Abs. Quant.	Rel. Quant.	Band %	Lane %
1		N/A	0,685	12 912 120	22 540 193	N/A	N/A	100,0	100,0

Band Detection	Automatically detected bands with sensitivity: Low
Lane Background	Lane background subtracted with disk size: 36.6
Lane Width	4.73 mm

Lane 14

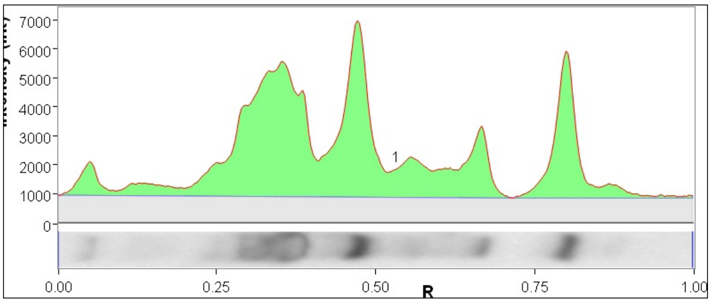

Band No.	Band Label	Mol. Wt. (KDa)	Relative Front	Adj. Volume (Int)	Volume (Int)	Abs. Quant.	Rel. Quant.	Band %	Lane %
1		N/A	0,533	15 705 790	26 095 646	N/A	N/A	100,0	100,0

Band Detection	Automatically detected bands with sensitivity: Low
Lane Background	Lane background subtracted with disk size: 66.4
Lane Width	5.19 mm

Image Report: Intensity analysis of Gapdh panel in Supplementary Fig. 2a, females

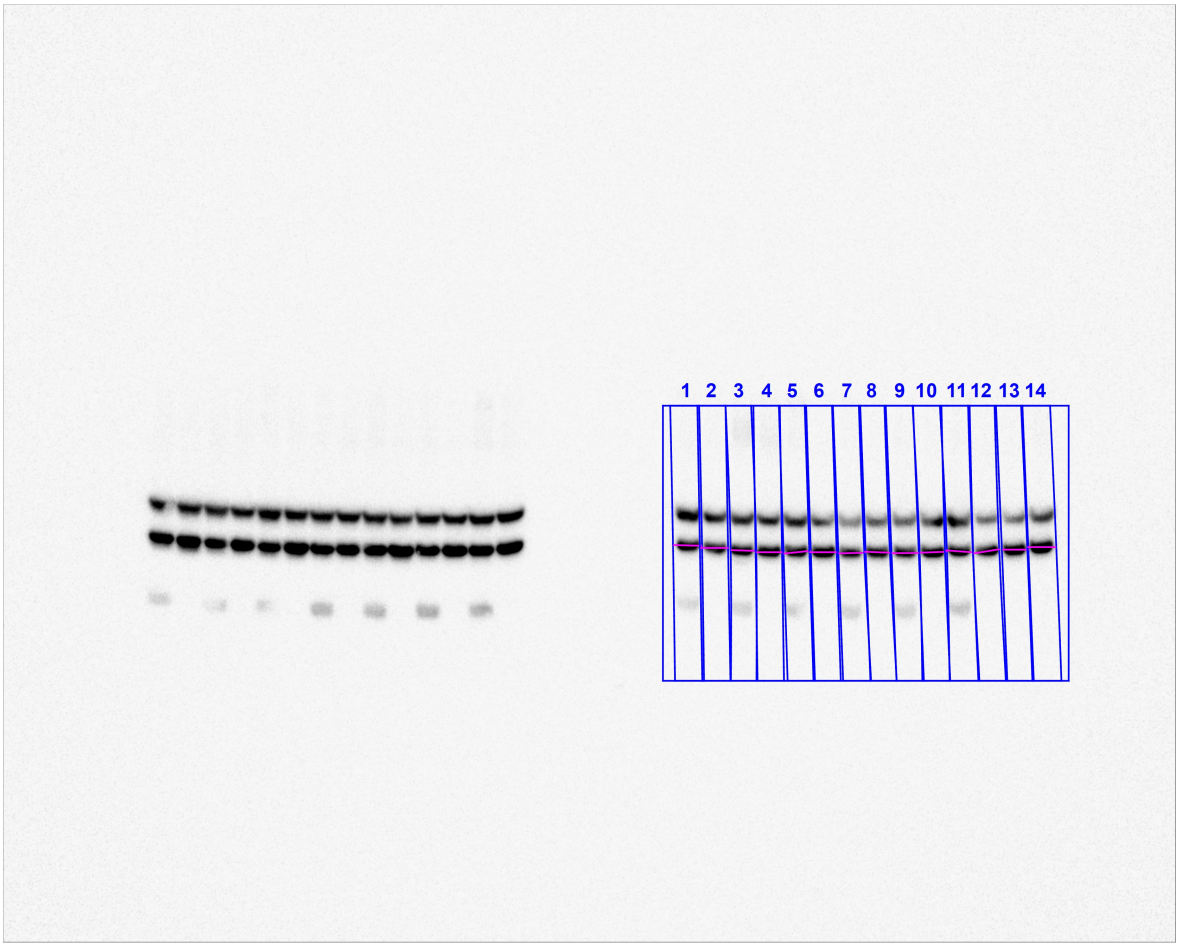

Acquisition Information

Imager	ChemiDoc MP
Exposure Time (sec)	9.987 (Signal Accumulation)
Serial Number	734BR-2876
Software Version	2.3.0.07
Application	Chemiluminescence
Excitation Source	No Illumination
Emission Filter	647SP Filter
Binning	2x2

Image Information

Acquisition Date	1/16/2021 6:18:28
User Name	Lipidlab
Image Area (mm)	X: 210.0 Y: 168.0
Pixel Size (µm)	X: 152.7 Y: 152.7

Data Range (Int)	500 - 25287

Analysis Settings

Detection	Lane detection:<br>Manually created lanes (Copied)<br><br>Band detection:<br>Automatically detected bands with sensitivity: Low<br>Manually adjusted bands<br><br>Lane Background Subtraction:<br>Lane background subtracted with disk size: 10.1<br><br>Lane width: Variable

Lane Statistics

Lane No.	Adj. Total Band Vol. (Int)	Total Band Vol. (Int)	Adj. Total Lane Vol. (Int)	Total Lane Vol. (Int)	Bkgd. Vol. (Int)	Norm. Factor
1	4 362 880	5 372 408	10 391 522	18 153 348	7 761 826	N/A
2	3 775 233	4 702 896	8 121 366	15 568 641	7 447 275	N/A
3	4 373 280	5 367 270	9 603 030	16 659 060	7 056 030	N/A
4	4 838 418	5 762 683	9 071 592	16 211 977	7 140 385	N/A
5	4 258 592	5 249 248	9 251 104	16 823 296	7 572 192	N/A
6	5 238 080	6 265 728	8 537 952	15 865 984	7 328 032	N/A
7	3 702 944	4 456 352	7 343 264	14 264 064	6 920 800	N/A
8	4 095 300	4 917 900	7 264 800	13 954 800	6 690 000	N/A
9	3 815 328	4 744 512	7 940 896	15 296 256	7 355 360	N/A
10	4 756 320	5 807 008	8 955 616	16 487 072	7 531 456	N/A
11	4 210 833	5 304 024	10 050 051	18 234 183	8 184 132	N/A
12	4 116 180	5 008 515	6 817 272	13 956 293	7 139 021	N/A
13	5 249 881	6 124 391	8 077 980	14 918 285	6 840 305	N/A
14	5 902 434	7 051 260	9 970 874	17 977 942	8 007 068	N/A

Lane And Band Analysis

Lane 1

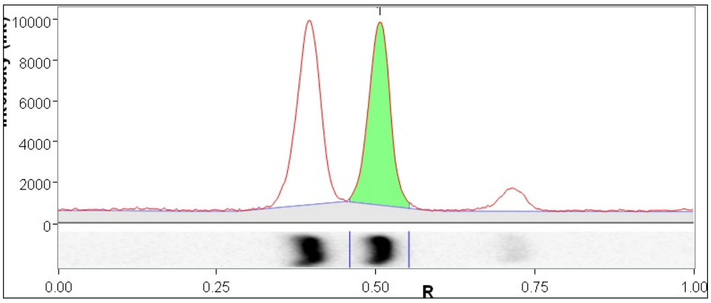

Band No.	Band Label	Mol. Wt. (KDa)	Relative Front	Adj. Volume (Int)	Volume (Int)	Abs. Quant.	Rel. Quant.	Band %	Lane %
1		N/A	0,508	4 362 880	5 372 408	N/A	N/A	100,0	42,0

Band Detection	Automatically detected bands with sensitivity: Low
Lane Background	Lane background subtracted with disk size: 10.1
Lane Width	5.19 mm

Lane 2

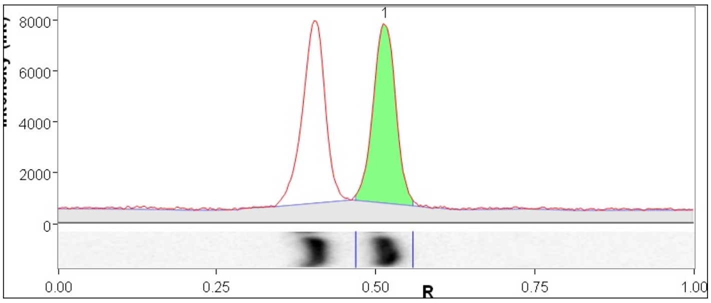

Band No.	Band Label	Mol. Wt. (KDa)	Relative Front	Adj. Volume (Int)	Volume (Int)	Abs. Quant.	Rel. Quant.	Band %	Lane %
1		N/A	0,517	3 775 233	4 702 896	N/A	N/A	100,0	46,5

Band Detection	Automatically detected bands with sensitivity: Low
Lane Background	Lane background subtracted with disk size: 10.1
Lane Width	5.04 mm

Lane 3

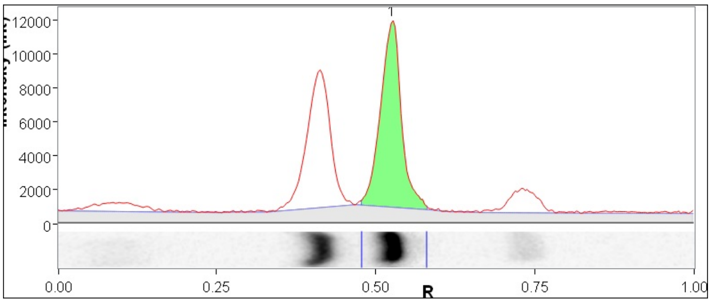

Band No.	Band Label	Mol. Wt. (KDa)	Relative Front	Adj. Volume (Int)	Volume (Int)	Abs. Quant.	Rel. Quant.	Band %	Lane %
1		N/A	0,526	4 373 280	5 367 270	N/A	N/A	100,0	45,5

Band Detection	Automatically detected bands with sensitivity: Low
Lane Background	Lane background subtracted with disk size: 10.1
Lane Width	4.58 mm

Lane 4

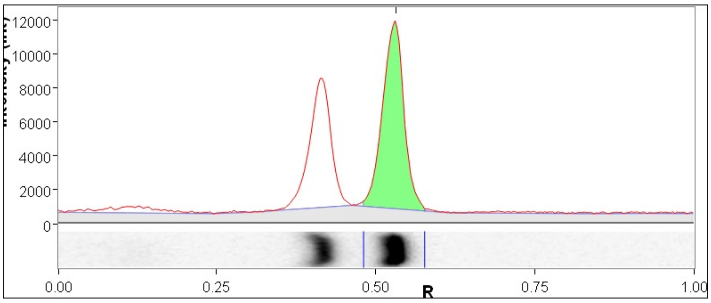

Band No.	Band Label	Mol. Wt. (KDa)	Relative Front	Adj. Volume (Int)	Volume (Int)	Abs. Quant.	Rel. Quant.	Band %	Lane %
1		N/A	0,533	4 838 418	5 762 683	N/A	N/A	100,0	53,3

Band Detection	Automatically detected bands with sensitivity: Low

Lane Background	Lane background subtracted with disk size: 10.1
Lane Width	4.73 mm

Lane 5

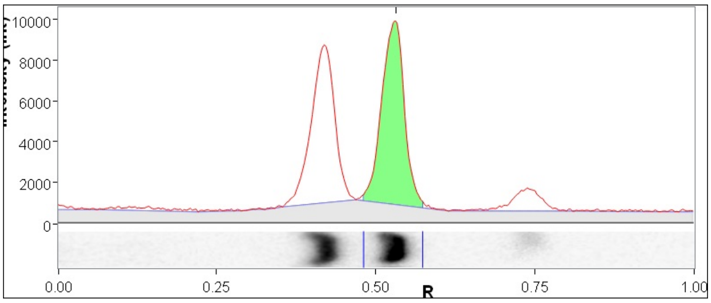

Band No.	Band Label	Mol. Wt. (KDa)	Relative Front	Adj. Volume (Int)	Volume (Int)	Abs. Quant.	Rel. Quant.	Band %	Lane %
1		N/A	0,533	4 258 592	5 249 248	N/A	N/A	100,0	46,0

Band Detection	Automatically detected bands with sensitivity: Low
Lane Background	Lane background subtracted with disk size: 10.1
Lane Width	4.89 mm

Lane 6

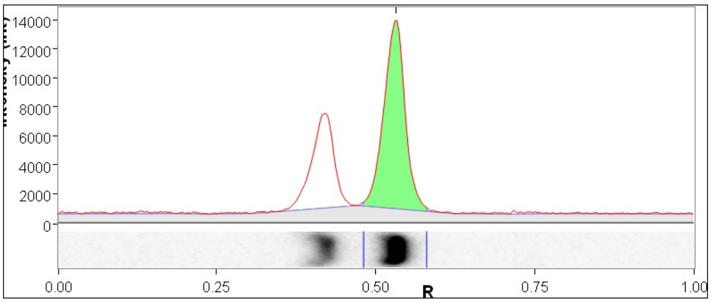

Band No.	Band Label	Mol. Wt. (KDa)	Relative Front	Adj. Volume (Int)	Volume (Int)	Abs. Quant.	Rel. Quant.	Band %	Lane %
1		N/A	0,533	5 238 080	6 265 728	N/A	N/A	100,0	61,4

Band Detection	Automatically detected bands with sensitivity: Low
Lane Background	Lane background subtracted with disk size: 10.1
Lane Width	4.89 mm

Lane 7

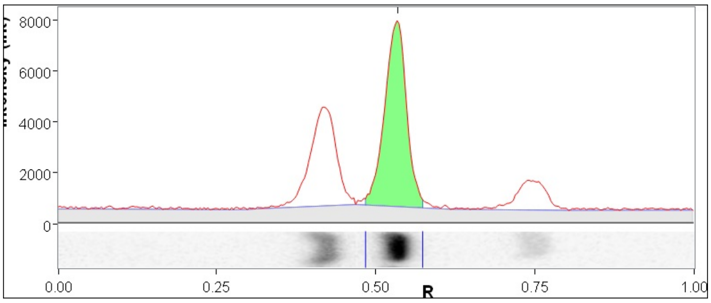

Band No.	Band Label	Mol. Wt. (KDa)	Relative Front	Adj. Volume (Int)	Volume (Int)	Abs. Quant.	Rel. Quant.	Band %	Lane %
1		N/A	0,536	3 702 944	4 456 352	N/A	N/A	100,0	50,4

Band Detection	Automatically detected bands with sensitivity: Low
Lane Background	Lane background subtracted with disk size: 10.1
Lane Width	4.89 mm

Lane 8

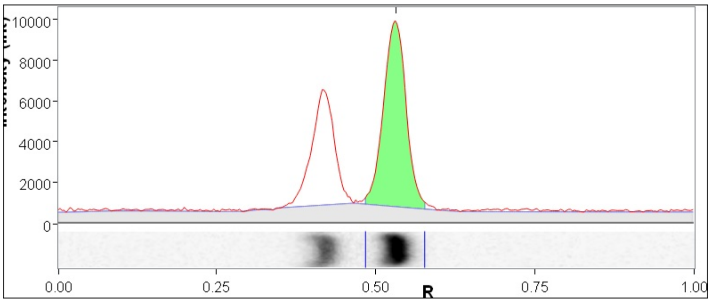

Band No.	Band Label	Mol. Wt. (KDa)	Relative Front	Adj. Volume (Int)	Volume (Int)	Abs. Quant.	Rel. Quant.	Band %	Lane %
1		N/A	0,533	4 095 300	4 917 900	N/A	N/A	100,0	56,4

Band Detection	Automatically detected bands with sensitivity: Low
Lane Background	Lane background subtracted with disk size: 10.1
Lane Width	4.58 mm

Lane 9

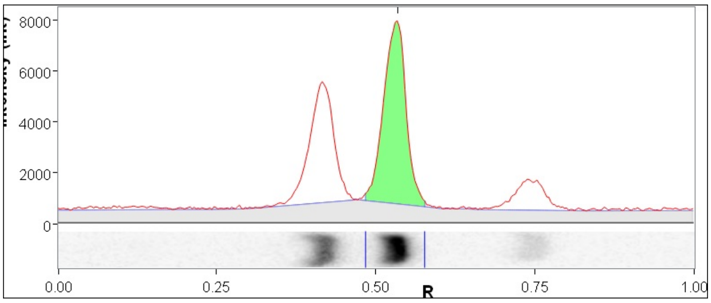

Band No.	Band Label	Mol. Wt. (KDa)	Relative Front	Adj. Volume (Int)	Volume (Int)	Abs. Quant.	Rel. Quant.	Band %	Lane %
1		N/A	0,536	3 815 328	4 744 512	N/A	N/A	100,0	48,0

Band Detection	Automatically detected bands with sensitivity: Low
Lane Background	Lane background subtracted with disk size: 10.1
Lane Width	4.89 mm

Lane 10

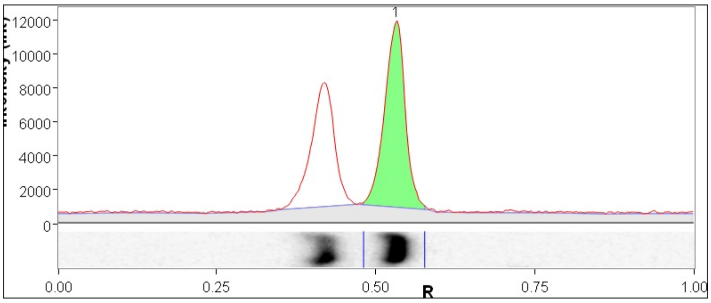

Band No.	Band Label	Mol. Wt. (KDa)	Relative Front	Adj. Volume (Int)	Volume (Int)	Abs. Quant.	Rel. Quant.	Band %	Lane %
1		N/A	0,533	4 756 320	5 807 008	N/A	N/A	100,0	53,1

Band Detection	Automatically detected bands with sensitivity: Low
Lane Background	Lane background subtracted with disk size: 10.1
Lane Width	4.89 mm

Lane 11

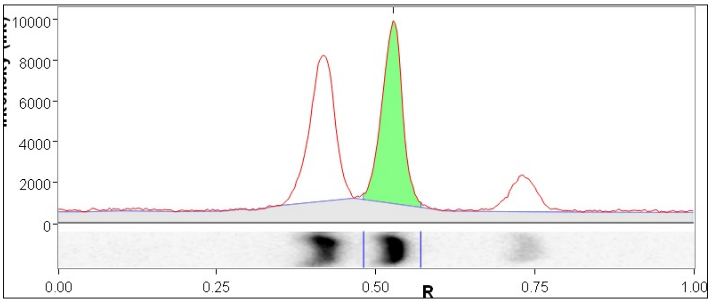

Band No.	Band Label	Mol. Wt. (KDa)	Relative Front	Adj. Volume (Int)	Volume (Int)	Abs. Quant.	Rel. Quant.	Band %	Lane %
1		N/A	0,529	4 210 833	5 304 024	N/A	N/A	100,0	41,9

Band Detection	Automatically detected bands with sensitivity: Low
Lane Background	Lane background subtracted with disk size: 10.1
Lane Width	5.04 mm

Lane 12

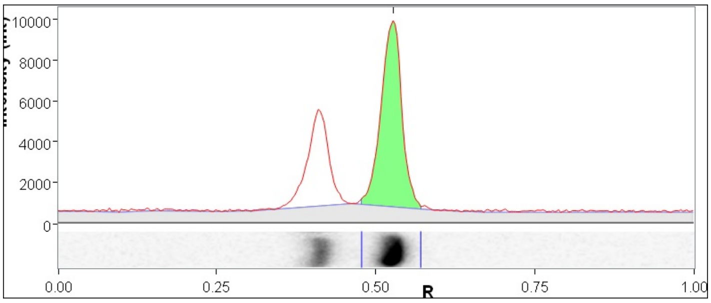

Band No.	Band Label	Mol. Wt. (KDa)	Relative Front	Adj. Volume (Int)	Volume (Int)	Abs. Quant.	Rel. Quant.	Band %	Lane %
1		N/A	0,529	4 116 180	5 008 515	N/A	N/A	100,0	60,4

Band Detection	Automatically detected bands with sensitivity: Low

Lane Background	Lane background subtracted with disk size: 10.1
Lane Width	4.73 mm

Lane 13

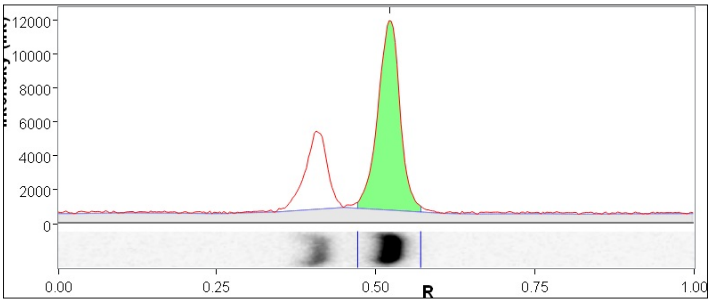

Band No.	Band Label	Mol. Wt. (KDa)	Relative Front	Adj. Volume (Int)	Volume (Int)	Abs. Quant.	Rel. Quant.	Band %	Lane %
1		N/A	0,523	5 249 881	6 124 391	N/A	N/A	100,0	65,0

Band Detection	Automatically detected bands with sensitivity: Low
Lane Background	Lane background subtracted with disk size: 10.1
Lane Width	4.73 mm

Lane 14

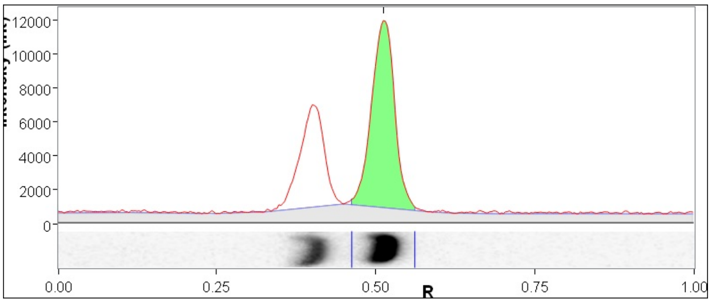

Band No.	Band Label	Mol. Wt. (KDa)	Relative Front	Adj. Volume (Int)	Volume (Int)	Abs. Quant.	Rel. Quant.	Band %	Lane %
1		N/A	0,514	5 902 434	7 051 260	N/A	N/A	100,0	59,2

Band Detection	Automatically detected bands with sensitivity: Low
Lane Background	Lane background subtracted with disk size: 10.1
Lane Width	5.19 mm

Image Report: Supplementary Fig. 4c, NRF2 panel

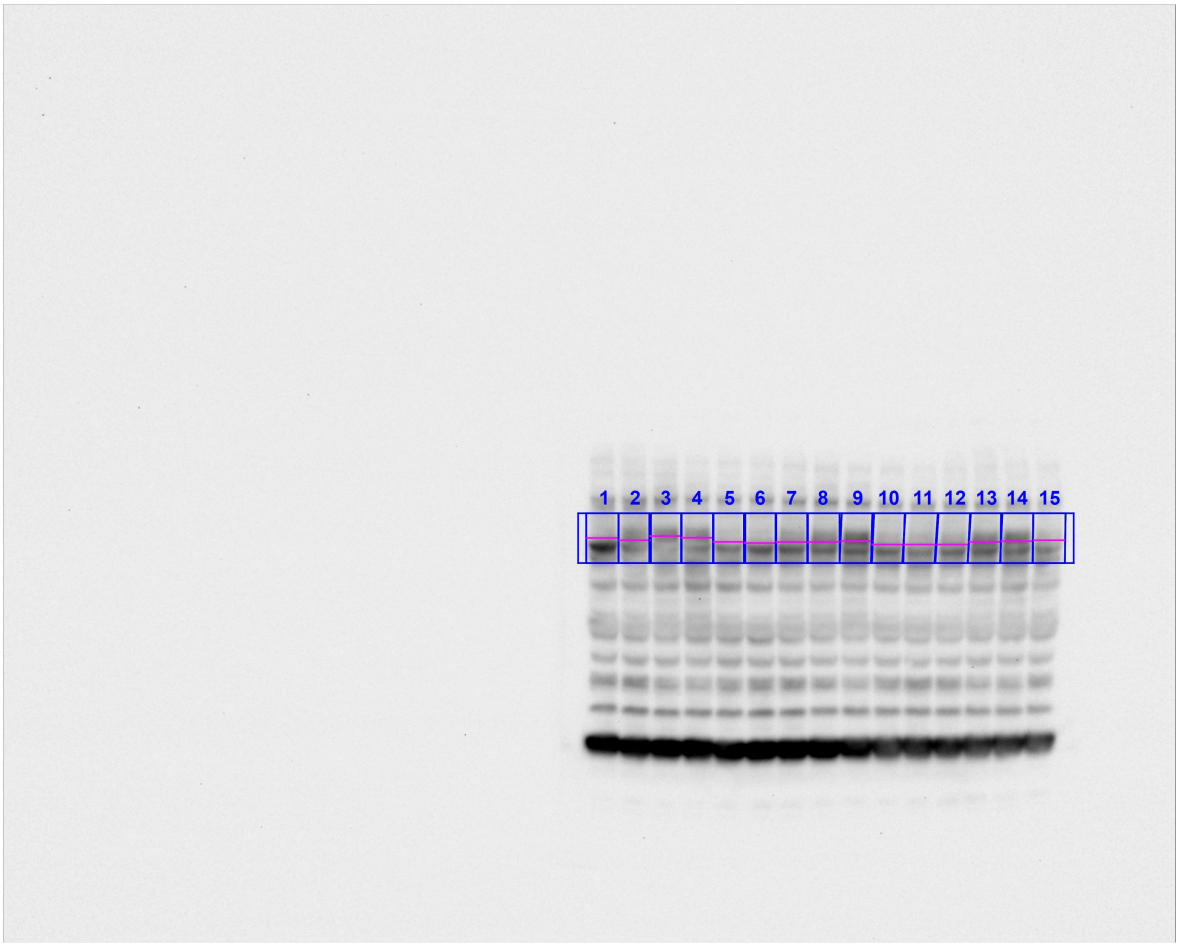

Acquisition Information

Imager	ChemiDoc Touch
Exposure Time (sec)	266.840 (Signal Accumulation)
Serial Number	732BR0263
Software Version	2.3.0.07
Application	Chemiluminescence
Excitation Source	No Illumination
Emission Filter	No Filter
Binning	2x2

Image Information

Acquisition Date	2/19/2023 3:27:30
User Name	m
Image Area (mm)	X: 180.0 Y: 144.1
Pixel Size (µm)	X: 130.5 Y: 130.5

Data Range (Int)	500 - 38169

Analysis Settings

Detection	Lane detection:<br>Manually created lanes (Copied)<br><br>Band detection:<br>Bands detected with different sensitivity per lane<br>Manually adjusted bands<br><br>Lane Background Subtraction:<br>Lane background subtracted with disk size: 24.1<br><br>Lane width: 4.96 mm

Lane Statistics

Lane No.	Adj. Total Band Vol. (Int)	Total Band Vol. (Int)	Adj. Total Lane Vol. (Int)	Total Lane Vol. (Int)	Bkgd. Vol. (Int)	Norm. Factor
1	810 046	2 538 590	8 139 486	14 513 492	6 374 006	N/A
2	2 488 544	5 159 564	4 983 130	12 146 320	7 163 190	N/A
3	3 337 160	5 714 060	5 465 692	11 075 176	5 609 484	N/A
4	2 945 722	4 822 618	6 789 042	12 062 226	5 273 184	N/A
5	1 037 856	2 744 816	5 159 792	10 195 324	5 035 532	N/A
6	1 234 012	3 159 320	6 136 050	11 299 376	5 163 326	N/A
7	2 180 326	4 466 710	7 073 738	12 938 810	5 865 072	N/A
8	3 469 894	5 794 544	7 622 952	13 109 126	5 486 174	N/A
9	5 028 882	7 126 482	9 946 614	15 103 214	5 156 600	N/A
10	1 588 894	3 627 936	5 960 148	11 190 734	5 230 586	N/A
11	1 518 670	3 807 220	5 078 358	10 479 336	5 400 978	N/A
12	1 950 616	4 118 516	5 848 504	10 964 748	5 116 244	N/A
13	3 895 342	6 046 142	8 461 346	13 537 234	5 075 888	N/A
14	4 520 784	6 265 440	9 223 322	13 512 268	4 288 946	N/A
15	1 047 736	2 296 074	4 915 034	8 791 452	3 876 418	N/A

Lane And Band Analysis

Lane 1

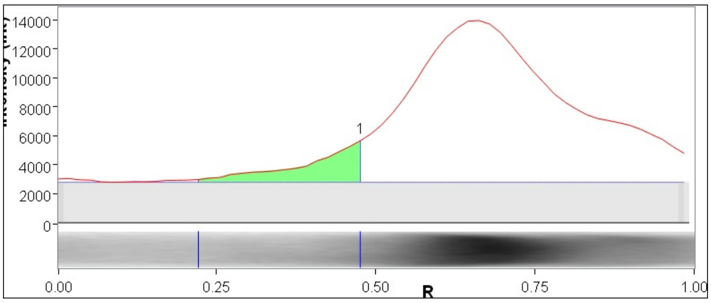

Band No.	Band Label	Mol. Wt. (KDa)	Relative Front	Adj. Volume (Int)	Volume (Int)	Abs. Quant.	Rel. Quant.	Band %	Lane %
1		N/A	0,495	810 046	2 538 590	N/A	N/A	100,0	10,0

Band Detection	Automatically detected bands with sensitivity: Low
Lane Background	Lane background subtracted with disk size: 24.1
Lane Width	4.96 mm

Lane 2

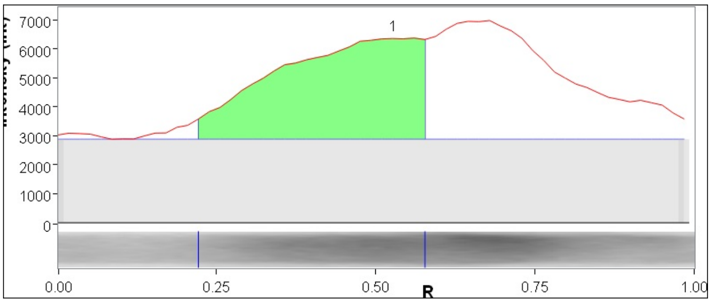

Band No.	Band Label	Mol. Wt. (KDa)	Relative Front	Adj. Volume (Int)	Volume (Int)	Abs. Quant.	Rel. Quant.	Band %	Lane %
1		N/A	0,542	2 488 544	5 159 564	N/A	N/A	100,0	49,9

Band Detection	Automatically detected bands with sensitivity: Low
Lane Background	Lane background subtracted with disk size: 24.1
Lane Width	4.96 mm

Lane 3

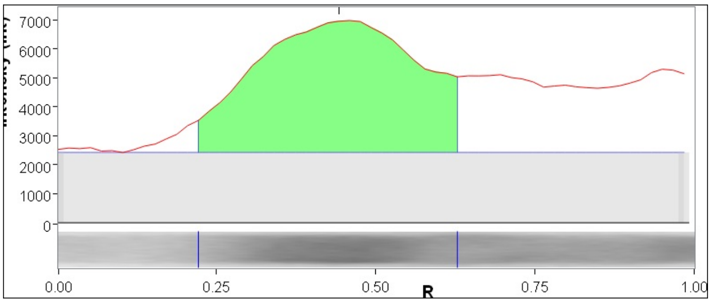

Band No.	Band Label	Mol. Wt. (KDa)	Relative Front	Adj. Volume (Int)	Volume (Int)	Abs. Quant.	Rel. Quant.	Band %	Lane %
1		N/A	0,458	3 337 160	5 714 060	N/A	N/A	100,0	61,1

Band Detection	Automatically detected bands with sensitivity: Low
Lane Background	Lane background subtracted with disk size: 24.1
Lane Width	4.96 mm

Lane 4

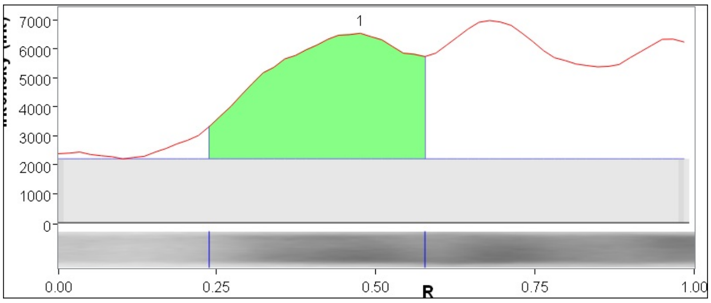

Band No.	Band Label	Mol. Wt. (KDa)	Relative Front	Adj. Volume (Int)	Volume (Int)	Abs. Quant.	Rel. Quant.	Band %	Lane %
1		N/A	0,492	2 945 722	4 822 618	N/A	N/A	100,0	43,4

Band Detection	Automatically detected bands with sensitivity: Low
Lane Background	Lane background subtracted with disk size: 24.1
Lane Width	4.96 mm

Lane 5

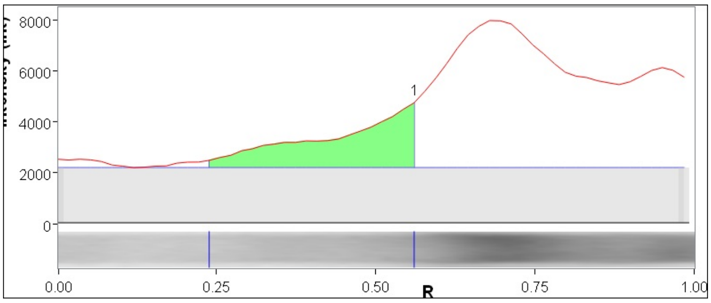

Band No.	Band Label	Mol. Wt. (KDa)	Relative Front	Adj. Volume (Int)	Volume (Int)	Abs. Quant.	Rel. Quant.	Band %	Lane %
1		N/A	0,574	1 037 856	2 744 816	N/A	N/A	100,0	20,1

Band Detection	Automatically detected bands with sensitivity: Low
Lane Background	Lane background subtracted with disk size: 24.1
Lane Width	4.96 mm

Lane 6

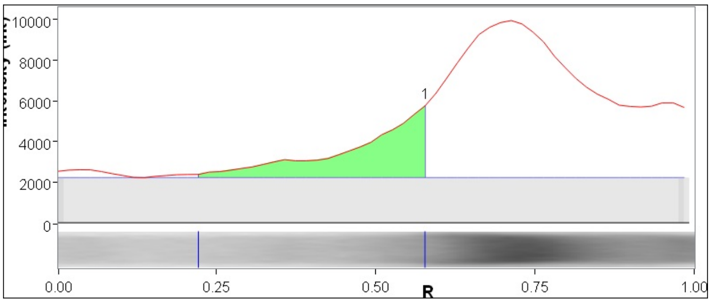

Band No.	Band Label	Mol. Wt. (KDa)	Relative Front	Adj. Volume (Int)	Volume (Int)	Abs. Quant.	Rel. Quant.	Band %	Lane %
1		N/A	0,597	1 234 012	3 159 320	N/A	N/A	100,0	20,1

Band Detection	Automatically detected bands with sensitivity: Low
Lane Background	Lane background subtracted with disk size: 24.1
Lane Width	4.96 mm

Lane 7

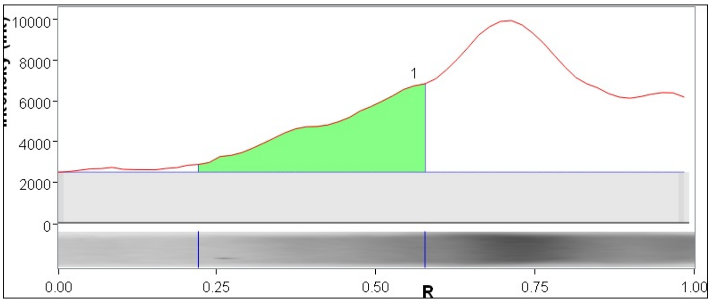

Band No.	Band Label	Mol. Wt. (KDa)	Relative Front	Adj. Volume (Int)	Volume (Int)	Abs. Quant.	Rel. Quant.	Band %	Lane %
1		N/A	0,576	2 180 326	4 466 710	N/A	N/A	100,0	30,8

Band Detection	Automatically detected bands with sensitivity: Low
Lane Background	Lane background subtracted with disk size: 24.1
Lane Width	4.96 mm

Lane 8

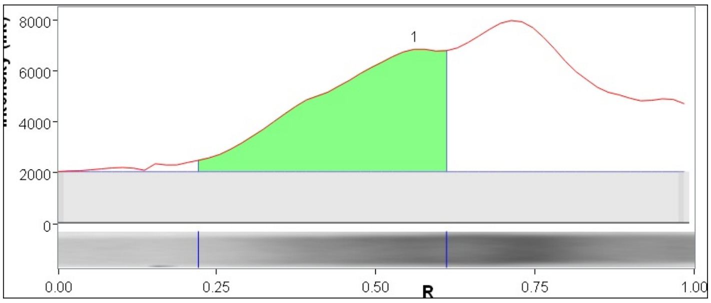

Band No.	Band Label	Mol. Wt. (KDa)	Relative Front	Adj. Volume (Int)	Volume (Int)	Abs. Quant.	Rel. Quant.	Band %	Lane %
1		N/A	0,576	3 469 894	5 794 544	N/A	N/A	100,0	45,5

Band Detection	Automatically detected bands with sensitivity: Low
Lane Background	Lane background subtracted with disk size: 24.1
Lane Width	4.96 mm

Lane 9

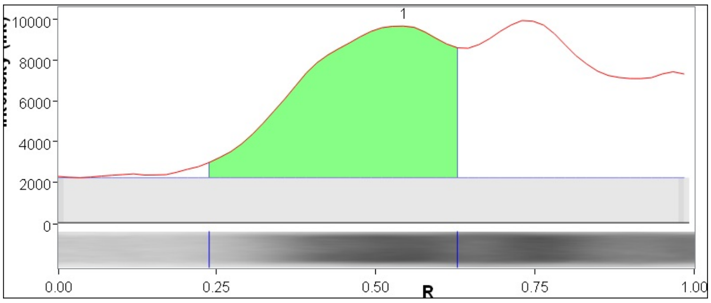

Band No.	Band Label	Mol. Wt. (KDa)	Relative Front	Adj. Volume (Int)	Volume (Int)	Abs. Quant.	Rel. Quant.	Band %	Lane %
1		N/A	0,559	5 028 882	7 126 482	N/A	N/A	100,0	50,6

Band Detection	Automatically detected bands with sensitivity: Low
Lane Background	Lane background subtracted with disk size: 24.1
Lane Width	4.96 mm

Lane 10

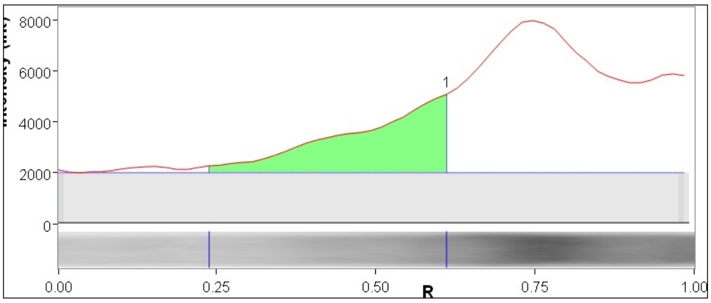

Band No.	Band Label	Mol. Wt. (KDa)	Relative Front	Adj. Volume (Int)	Volume (Int)	Abs. Quant.	Rel. Quant.	Band %	Lane %
1		N/A	0,627	1 588 894	3 627 936	N/A	N/A	100,0	26,7

Band Detection	Automatically detected bands with sensitivity: Low
Lane Background	Lane background subtracted with disk size: 24.1
Lane Width	4.96 mm

Lane 11

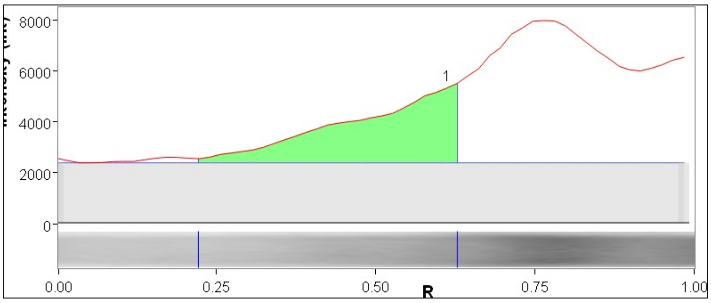

Band No.	Band Label	Mol. Wt. (KDa)	Relative Front	Adj. Volume (Int)	Volume (Int)	Abs. Quant.	Rel. Quant.	Band %	Lane %
1		N/A	0,627	1 518 670	3 807 220	N/A	N/A	100,0	29,9

Band Detection	Automatically detected bands with sensitivity: Low
Lane Background	Lane background subtracted with disk size: 24.1
Lane Width	4.96 mm

Lane 12

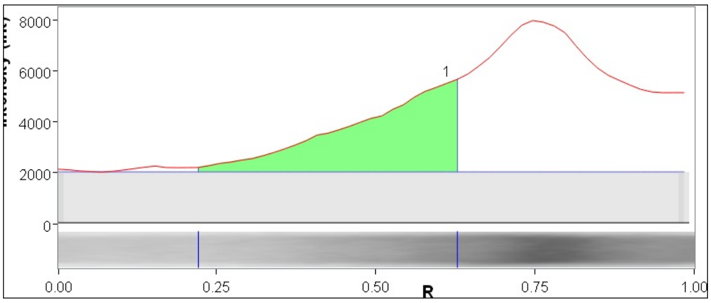

Band No.	Band Label	Mol. Wt. (KDa)	Relative Front	Adj. Volume (Int)	Volume (Int)	Abs. Quant.	Rel. Quant.	Band %	Lane %
1		N/A	0,627	1 950 616	4 118 516	N/A	N/A	100,0	33,4

Band Detection	Automatically detected bands with sensitivity: Low

Lane Background	Lane background subtracted with disk size: 24.1
Lane Width	4.96 mm

Lane 13

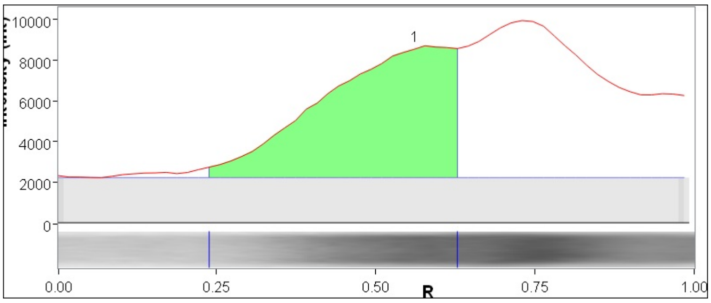

Band No.	Band Label	Mol. Wt. (KDa)	Relative Front	Adj. Volume (Int)	Volume (Int)	Abs. Quant.	Rel. Quant.	Band %	Lane %
1		N/A	0,576	3 895 342	6 046 142	N/A	N/A	100,0	46,0

Band Detection	Automatically detected bands with sensitivity: Low
Lane Background	Lane background subtracted with disk size: 24.1
Lane Width	4.96 mm

Lane 14

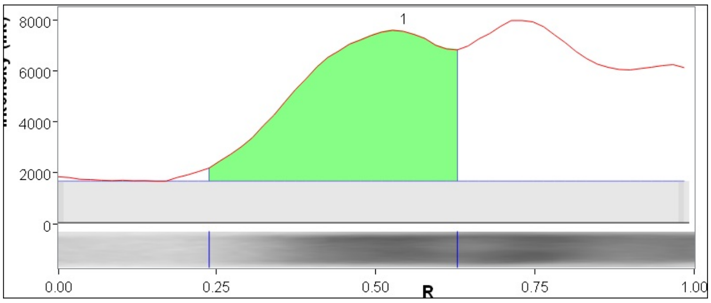

Band No.	Band Label	Mol. Wt. (KDa)	Relative Front	Adj. Volume (Int)	Volume (Int)	Abs. Quant.	Rel. Quant.	Band %	Lane %
1		N/A	0,559	4 520 784	6 265 440	N/A	N/A	100,0	49,0

Band Detection	Automatically detected bands with sensitivity: Low
Lane Background	Lane background subtracted with disk size: 24.1
Lane Width	4.96 mm

Lane 15

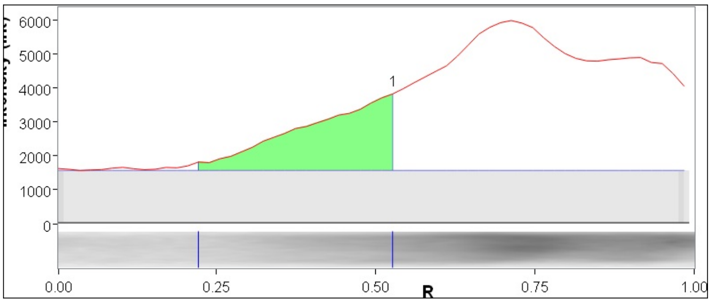

Band No.	Band Label	Mol. Wt. (KDa)	Relative Front	Adj. Volume (Int)	Volume (Int)	Abs. Quant.	Rel. Quant.	Band %	Lane %
1		N/A	0,542	1 047 736	2 296 074	N/A	N/A	100,0	21,3

Lane Background	Lane background subtracted with disk size: 24.1
Lane Width	4.96 mm

Image Report: Supplementary Fig. 4c, HMOX1 panel

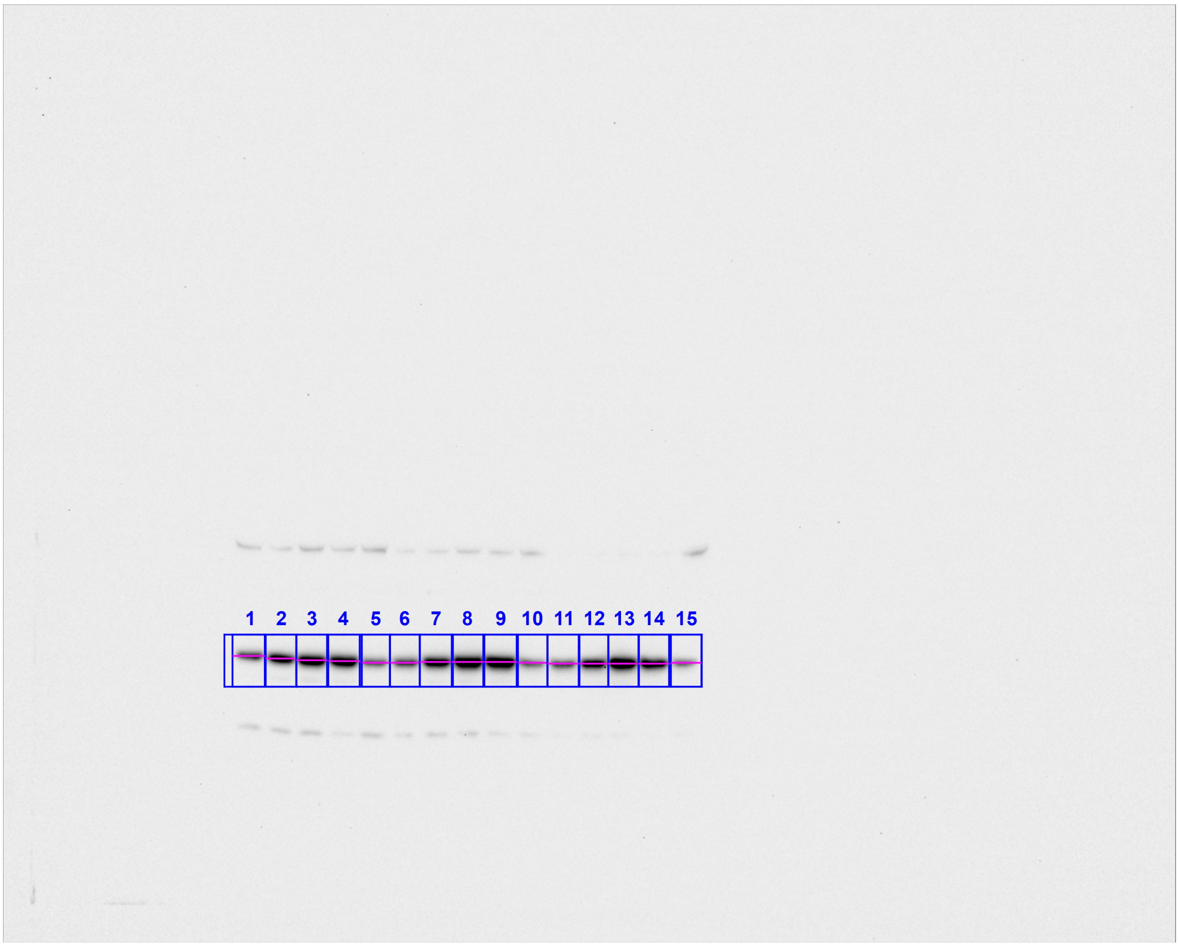

Acquisition Information

Imager	ChemiDoc Touch
Exposure Time (sec)	250.000 (Signal Accumulation)
Serial Number	732BR0263
Software Version	2.3.0.07
Application	Chemiluminescence
Excitation Source	No Illumination
Emission Filter	No Filter
Binning	2x2

Image Information

Acquisition Date	2/19/2023 10:42:43
User Name	m
Image Area (mm)	X: 180.0 Y: 144.1
Pixel Size (µm)	X: 130.5 Y: 130.5

Data Range (Int)	500 - 51232

Analysis Settings

Detection	Lane detection:<br>Manually created lanes<br><br>Band detection:<br>Automatically detected bands with sensitivity: Low<br><br>Lane Background Subtraction:<br>Lane background subtracted with disk size: 24.1<br><br>Lane width: Variable

Lane Statistics

Lane No.	Adj. Total Band Vol. (Int)	Total Band Vol. (Int)	Adj. Total Lane Vol. (Int)	Total Lane Vol. (Int)	Bkgd. Vol. (Int)	Norm. Factor
1	3 828 500	4 316 420	4 006 188	5 494 344	1 488 156	N/A
2	7 330 032	7 899 408	7 586 352	9 033 516	1 447 164	N/A
3	10 062 072	10 754 136	10 359 036	11 922 588	1 563 552	N/A
4	9 646 788	10 342 092	9 927 655	11 498 527	1 570 872	N/A
5	2 941 620	3 373 920	3 155 460	4 473 975	1 318 515	N/A
6	3 924 060	4 440 730	4 149 355	5 581 940	1 432 585	N/A
7	8 618 220	9 337 146	8 933 028	10 619 739	1 686 711	N/A
8	13 055 598	13 911 408	13 412 352	15 152 499	1 740 147	N/A
9	13 235 590	14 089 450	13 516 372	15 252 554	1 736 182	N/A
10	2 799 930	3 236 835	3 080 035	4 482 730	1 402 695	N/A
11	3 585 152	4 086 317	3 833 644	5 289 409	1 455 765	N/A
12	7 462 320	8 032 670	7 778 622	9 170 276	1 391 654	N/A
13	13 208 292	13 959 972	13 587 876	15 116 292	1 528 416	N/A
14	8 088 732	8 761 716	8 308 404	9 887 328	1 578 924	N/A
15	1 968 228	2 365 416	2 141 244	3 566 448	1 425 204	N/A

Lane And Band Analysis

Lane 1

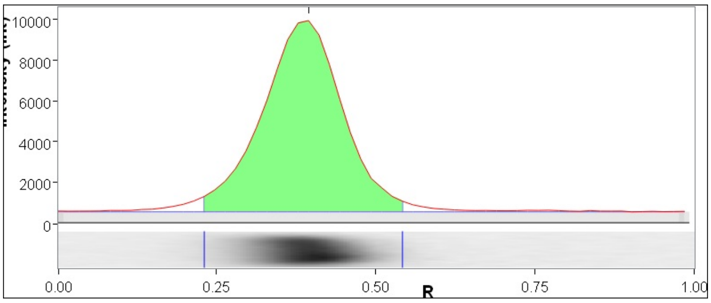

Band No.	Band Label	Mol. Wt. (KDa)	Relative Front	Adj. Volume (Int)	Volume (Int)	Abs. Quant.	Rel. Quant.	Band %	Lane %
1		N/A	0,410	3 828 500	4 316 420	N/A	N/A	100,0	95,6

Band Detection	Automatically detected bands with sensitivity: Low
Lane Background	Lane background subtracted with disk size: 24.1
Lane Width	4.96 mm

Lane 2

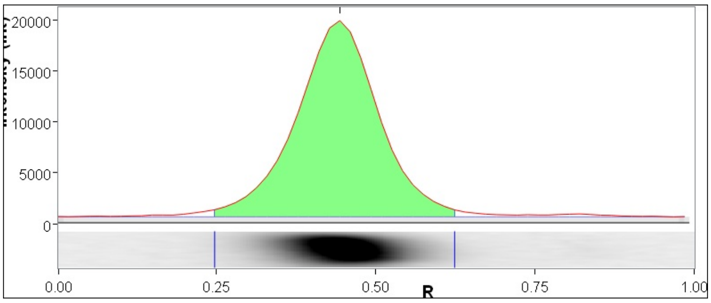

Band No.	Band Label	Mol. Wt. (KDa)	Relative Front	Adj. Volume (Int)	Volume (Int)	Abs. Quant.	Rel. Quant.	Band %	Lane %
1		N/A	0,459	7 330 032	7 899 408	N/A	N/A	100,0	96,6

Band Detection	Automatically detected bands with sensitivity: Low
Lane Background	Lane background subtracted with disk size: 24.1
Lane Width	4.70 mm

Lane 3

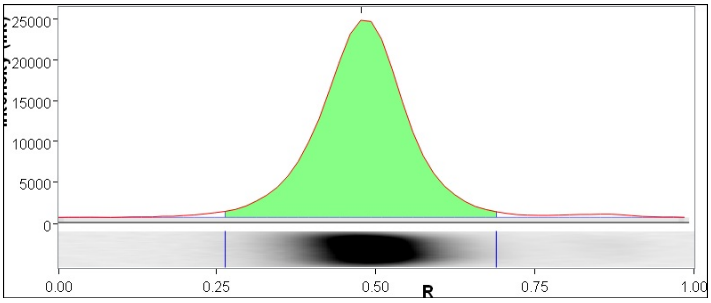

Band No.	Band Label	Mol. Wt. (KDa)	Relative Front	Adj. Volume (Int)	Volume (Int)	Abs. Quant.	Rel. Quant.	Band %	Lane %
1		N/A	0,492	10 062 072	10 754 136	N/A	N/A	100,0	97,1

Band Detection	Automatically detected bands with sensitivity: Low
Lane Background	Lane background subtracted with disk size: 24.1
Lane Width	4.70 mm

Lane 4

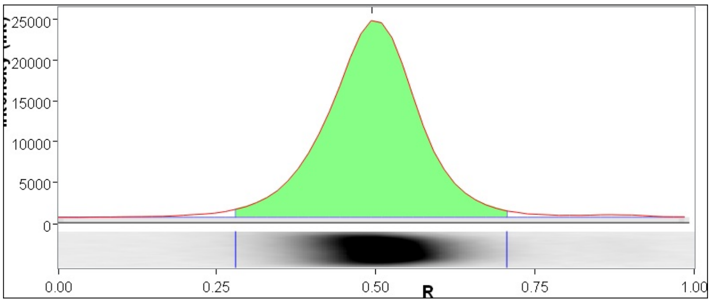

Band No.	Band Label	Mol. Wt. (KDa)	Relative Front	Adj. Volume (Int)	Volume (Int)	Abs. Quant.	Rel. Quant.	Band %	Lane %
1		N/A	0,508	9 646 788	10 342 092	N/A	N/A	100,0	97,2

Band Detection	Automatically detected bands with sensitivity: Low
Lane Background	Lane background subtracted with disk size: 24.1
Lane Width	4.83 mm

Lane 5

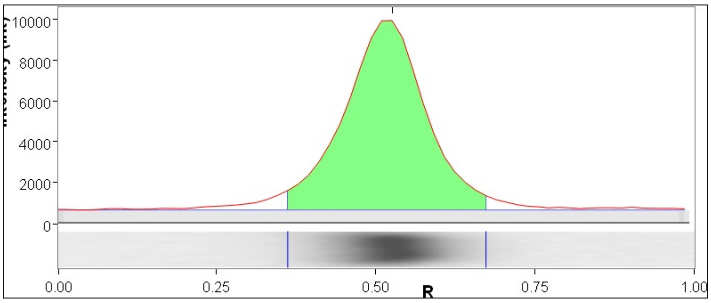

Band No.	Band Label	Mol. Wt. (KDa)	Relative Front	Adj. Volume (Int)	Volume (Int)	Abs. Quant.	Rel. Quant.	Band %	Lane %
1		N/A	0,541	2 941 620	3 373 920	N/A	N/A	100,0	93,2

Band Detection	Automatically detected bands with sensitivity: Low
Lane Background	Lane background subtracted with disk size: 24.1
Lane Width	4.31 mm

Lane 6

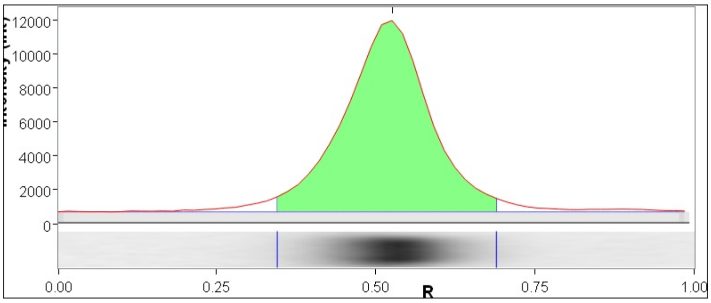

Band No.	Band Label	Mol. Wt. (KDa)	Relative Front	Adj. Volume (Int)	Volume (Int)	Abs. Quant.	Rel. Quant.	Band %	Lane %
1		N/A	0,541	3 924 060	4 440 730	N/A	N/A	100,0	94,6

Band Detection	Automatically detected bands with sensitivity: Low
Lane Background	Lane background subtracted with disk size: 24.1
Lane Width	4.57 mm

Lane 7

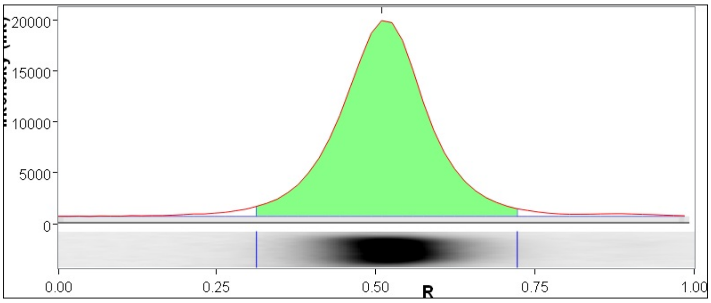

Band No.	Band Label	Mol. Wt. (KDa)	Relative Front	Adj. Volume (Int)	Volume (Int)	Abs. Quant.	Rel. Quant.	Band %	Lane %
1		N/A	0,525	8 618 220	9 337 146	N/A	N/A	100,0	96,5

Band Detection	Automatically detected bands with sensitivity: Low
Lane Background	Lane background subtracted with disk size: 24.1
Lane Width	5.09 mm

Lane 8

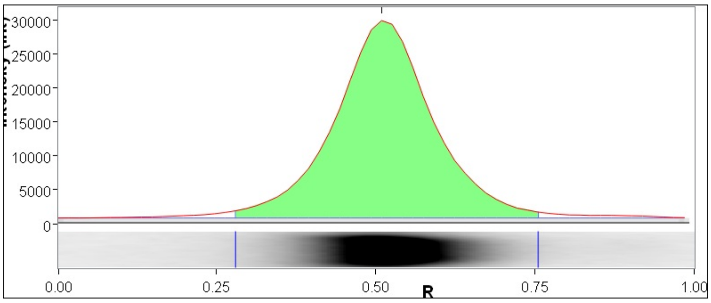

Band No.	Band Label	Mol. Wt. (KDa)	Relative Front	Adj. Volume (Int)	Volume (Int)	Abs. Quant.	Rel. Quant.	Band %	Lane %
1		N/A	0,525	13 055 598	13 911 408	N/A	N/A	100,0	97,3

Band Detection	Automatically detected bands with sensitivity: Low
Lane Background	Lane background subtracted with disk size: 24.1
Lane Width	4.83 mm

Lane 9

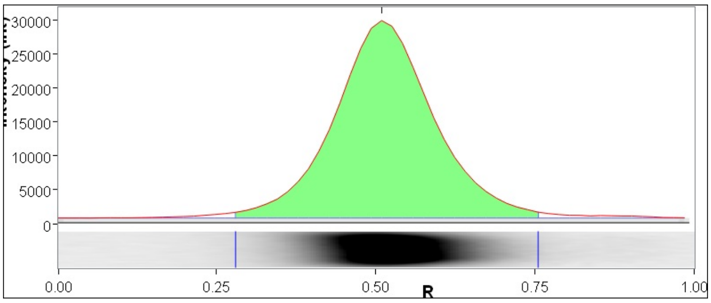

Band No.	Band Label	Mol. Wt. (KDa)	Relative Front	Adj. Volume (Int)	Volume (Int)	Abs. Quant.	Rel. Quant.	Band %	Lane %
1		N/A	0,525	13 235 590	14 089 450	N/A	N/A	100,0	97,9

Band Detection	Automatically detected bands with sensitivity: Low
Lane Background	Lane background subtracted with disk size: 24.1
Lane Width	4.96 mm

Lane 10

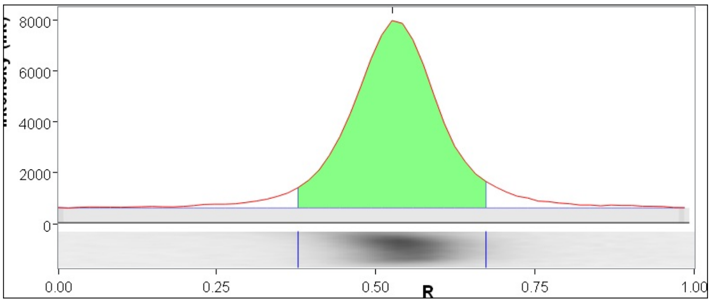

Band No.	Band Label	Mol. Wt. (KDa)	Relative Front	Adj. Volume (Int)	Volume (Int)	Abs. Quant.	Rel. Quant.	Band %	Lane %
1		N/A	0,541	2 799 930	3 236 835	N/A	N/A	100,0	90,9

Band Detection	Automatically detected bands with sensitivity: Low
Lane Background	Lane background subtracted with disk size: 24.1
Lane Width	4.57 mm

Lane 11

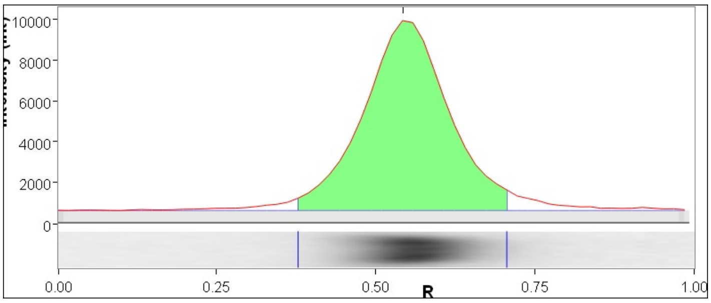

Band No.	Band Label	Mol. Wt. (KDa)	Relative Front	Adj. Volume (Int)	Volume (Int)	Abs. Quant.	Rel. Quant.	Band %	Lane %
1		N/A	0,557	3 585 152	4 086 317	N/A	N/A	100,0	93,5

Band Detection	Automatically detected bands with sensitivity: Low
Lane Background	Lane background subtracted with disk size: 24.1
Lane Width	4.83 mm

Lane 12

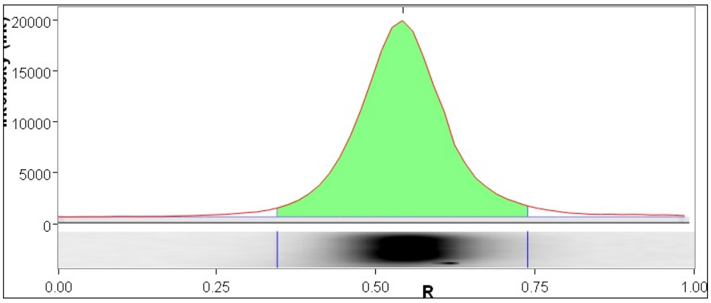

Band No.	Band Label	Mol. Wt. (KDa)	Relative Front	Adj. Volume (Int)	Volume (Int)	Abs. Quant.	Rel. Quant.	Band %	Lane %
1		N/A	0,557	7 462 320	8 032 670	N/A	N/A	100,0	95,9

Band Detection	Automatically detected bands with sensitivity: Low

Lane Background	Lane background subtracted with disk size: 24.1
Lane Width	4.44 mm

Lane 13

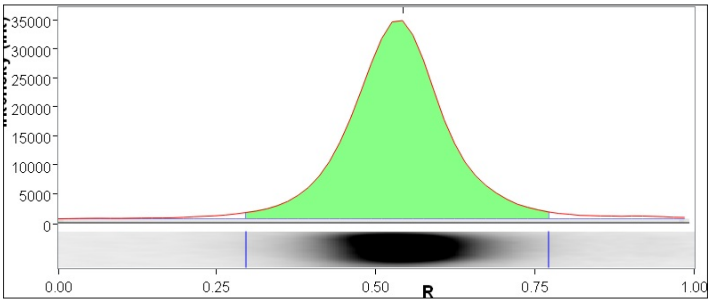

Band No.	Band Label	Mol. Wt. (KDa)	Relative Front	Adj. Volume (Int)	Volume (Int)	Abs. Quant.	Rel. Quant.	Band %	Lane %
1		N/A	0,557	13 208 292	13 959 972	N/A	N/A	100,0	97,2

Band Detection	Automatically detected bands with sensitivity: Low
Lane Background	Lane background subtracted with disk size: 24.1
Lane Width	4.70 mm

Lane 14

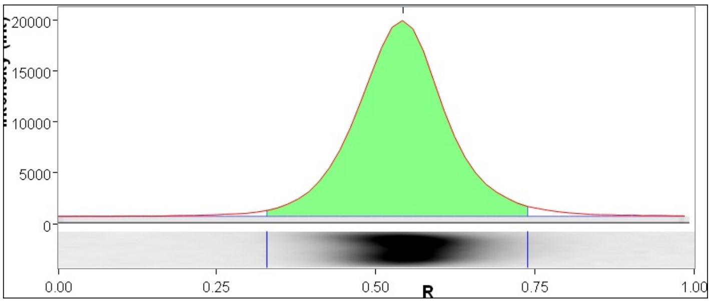

Band No.	Band Label	Mol. Wt. (KDa)	Relative Front	Adj. Volume (Int)	Volume (Int)	Abs. Quant.	Rel. Quant.	Band %	Lane %
1		N/A	0,557	8 088 732	8 761 716	N/A	N/A	100,0	97,4

Band Detection	Automatically detected bands with sensitivity: Low
Lane Background	Lane background subtracted with disk size: 24.1
Lane Width	4.70 mm

Lane 15

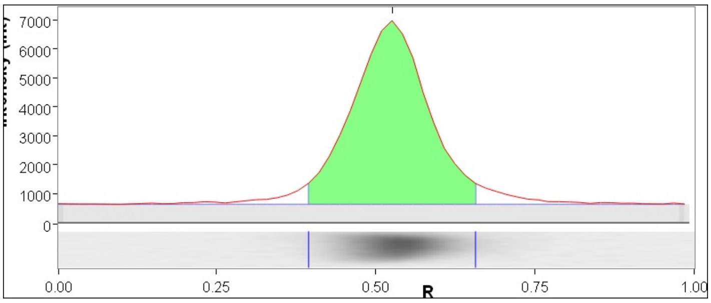

Band No.	Band Label	Mol. Wt. (KDa)	Relative Front	Adj. Volume (Int)	Volume (Int)	Abs. Quant.	Rel. Quant.	Band %	Lane %
1		N/A	0,541	1 968 228	2 365 416	N/A	N/A	100,0	91,9

Band Detection	Automatically detected bands with sensitivity: Low
Lane Background	Lane background subtracted with disk size: 24.1
Lane Width	4.70 mm

Image Report: Supplementary Fig. 4c, NQO1 panel

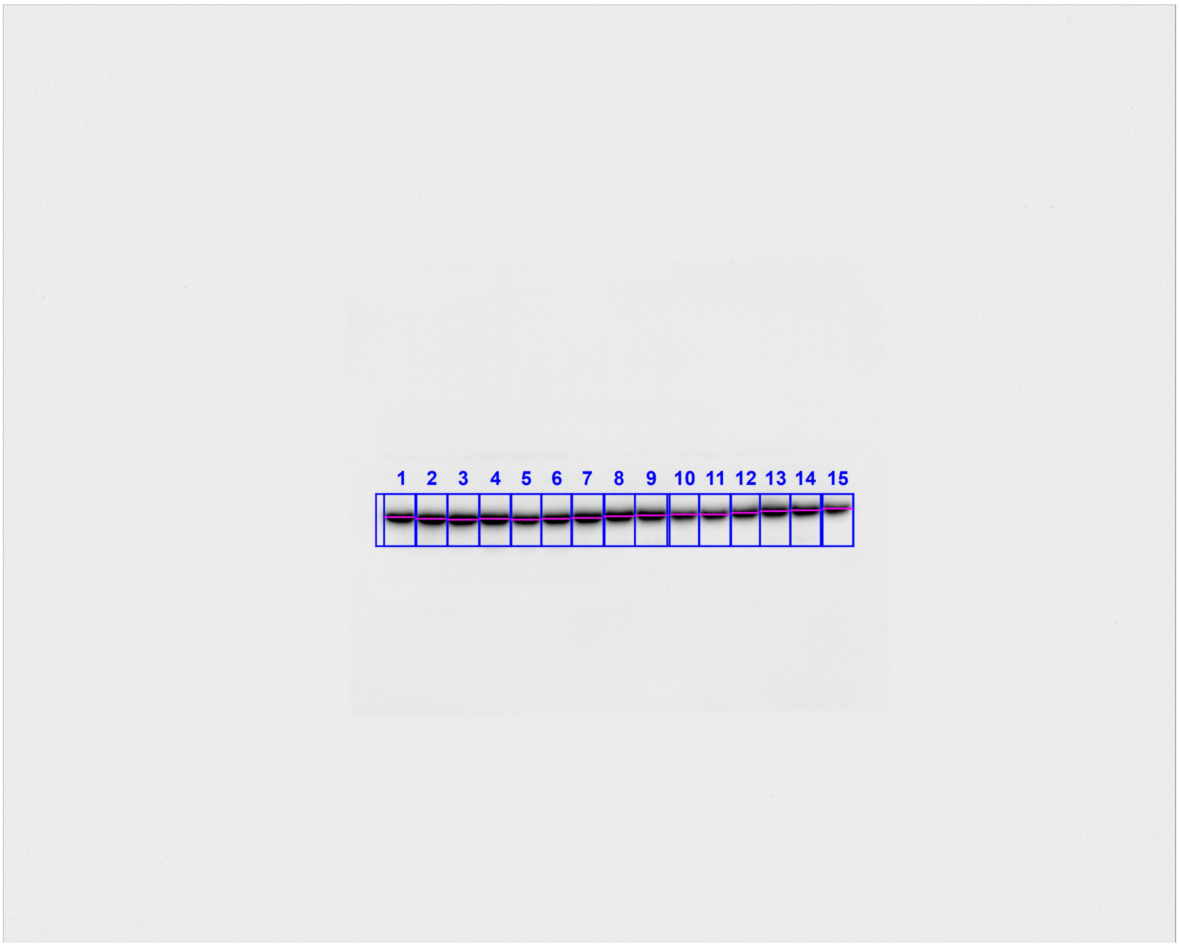

Acquisition Information

Imager	ChemiDoc Touch
Exposure Time (sec)	120.000 (Signal Accumulation)
Serial Number	732BR0263
Software Version	2.3.0.07
Application	Chemiluminescence
Excitation Source	No Illumination
Emission Filter	No Filter
Binning	2x2

Image Information

Acquisition Date	2/21/2023 11:53:05
User Name	m
Image Area (mm)	X: 180.0 Y: 144.1
Pixel Size (µm)	X: 130.5 Y: 130.5

Data Range (Int)	500 - 43426

Analysis Settings

Detection	Lane detection:<br>Manually created lanes (Copied)<br><br>Band detection:<br>Automatically detected bands with sensitivity: Low<br><br>Lane Background Subtraction:<br>Lane background subtracted with disk size: 24.1<br><br>Lane width: Variable

Lane Statistics

Lane No.	Adj. Total Band Vol. (Int)	Total Band Vol. (Int)	Adj. Total Lane Vol. (Int)	Total Lane Vol. (Int)	Bkgd. Vol. (Int)	Norm. Factor
1	11 188 758	12 291 860	11 483 600	13 803 918	2 320 318	N/A
2	13 855 864	15 038 576	14 298 488	16 625 760	2 327 272	N/A
3	14 284 162	15 590 146	14 553 050	17 042 582	2 489 532	N/A
4	13 378 090	14 707 178	13 676 162	16 209 736	2 533 574	N/A
5	9 950 652	11 048 940	10 198 152	12 508 344	2 310 192	N/A
6	11 562 732	12 773 412	11 819 592	14 281 308	2 461 716	N/A
7	12 840 594	14 096 745	13 127 868	15 599 649	2 471 781	N/A
8	12 057 042	13 158 162	12 313 859	14 480 579	2 166 720	N/A
9	11 411 628	12 542 508	11 650 724	13 876 004	2 225 280	N/A
10	7 528 955	8 325 695	7 838 250	9 574 005	1 735 755	N/A
11	7 869 086	8 684 270	8 123 313	9 965 025	1 841 712	N/A
12	8 719 674	9 499 600	8 996 128	10 636 662	1 640 534	N/A
13	10 704 456	11 693 448	10 888 884	12 663 252	1 774 368	N/A
14	9 767 376	10 726 992	9 991 872	11 821 140	1 829 268	N/A
15	6 615 396	7 445 376	6 721 704	8 467 524	1 745 820	N/A

Lane And Band Analysis

Lane 1

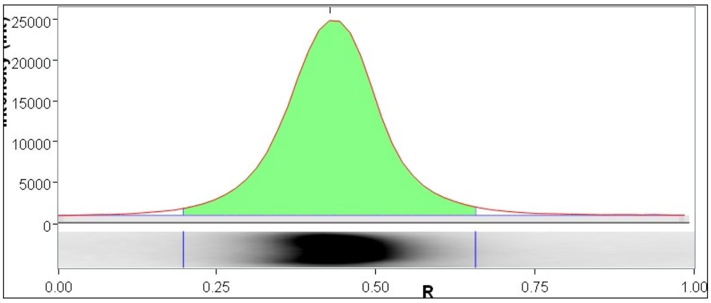

Band No.	Band Label	Mol. Wt. (KDa)	Relative Front	Adj. Volume (Int)	Volume (Int)	Abs. Quant.	Rel. Quant.	Band %	Lane %
1		N/A	0,443	11 188 758	12 291 860	N/A	N/A	100,0	97,4

Band Detection	Automatically detected bands with sensitivity: Low
Lane Background	Lane background subtracted with disk size: 24.1
Lane Width	4.96 mm

Lane 2

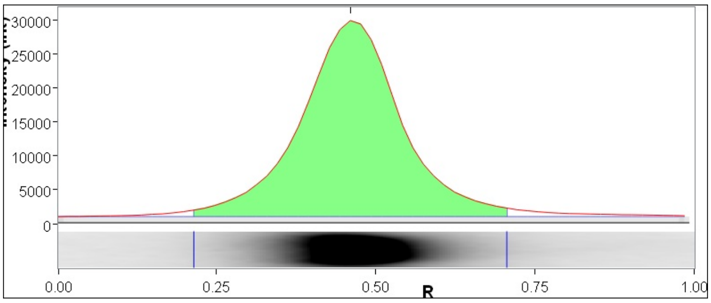

Band No.	Band Label	Mol. Wt. (KDa)	Relative Front	Adj. Volume (Int)	Volume (Int)	Abs. Quant.	Rel. Quant.	Band %	Lane %
1		N/A	0,475	13 855 864	15 038 576	N/A	N/A	100,0	96,9

Band Detection	Automatically detected bands with sensitivity: Low
Lane Background	Lane background subtracted with disk size: 24.1
Lane Width	4.96 mm

Lane 3

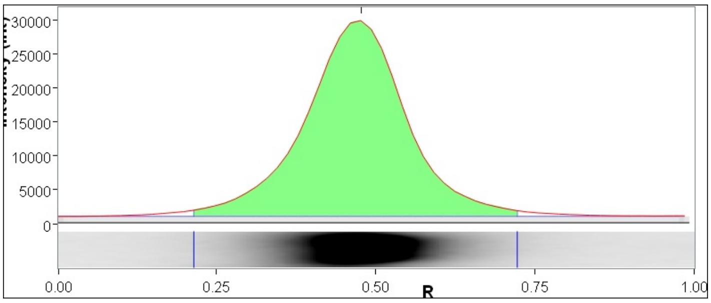

Band No.	Band Label	Mol. Wt. (KDa)	Relative Front	Adj. Volume (Int)	Volume (Int)	Abs. Quant.	Rel. Quant.	Band %	Lane %
1		N/A	0,492	14 284 162	15 590 146	N/A	N/A	100,0	98,2

Band Detection	Automatically detected bands with sensitivity: Low
Lane Background	Lane background subtracted with disk size: 24.1
Lane Width	4.96 mm

Lane 4

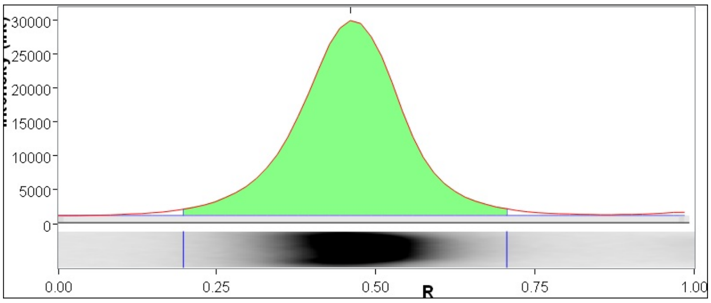

Band No.	Band Label	Mol. Wt. (KDa)	Relative Front	Adj. Volume (Int)	Volume (Int)	Abs. Quant.	Rel. Quant.	Band %	Lane %
1		N/A	0,475	13 378 090	14 707 178	N/A	N/A	100,0	97,8

Band Detection	Automatically detected bands with sensitivity: Low
Lane Background	Lane background subtracted with disk size: 24.1
Lane Width	4.96 mm

Lane 5

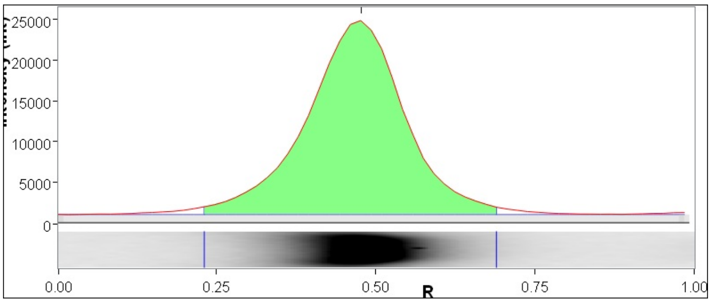

Band No.	Band Label	Mol. Wt. (KDa)	Relative Front	Adj. Volume (Int)	Volume (Int)	Abs. Quant.	Rel. Quant.	Band %	Lane %
1		N/A	0,492	9 950 652	11 048 940	N/A	N/A	100,0	97,6

Band Detection	Automatically detected bands with sensitivity: Low
Lane Background	Lane background subtracted with disk size: 24.1
Lane Width	4.70 mm

Lane 6

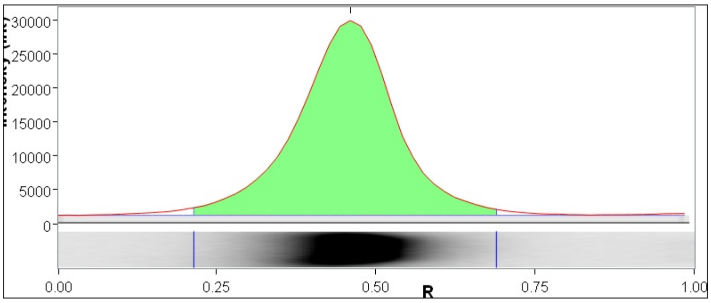

Band No.	Band Label	Mol. Wt. (KDa)	Relative Front	Adj. Volume (Int)	Volume (Int)	Abs. Quant.	Rel. Quant.	Band %	Lane %
1		N/A	0,475	11 562 732	12 773 412	N/A	N/A	100,0	97,8

Band Detection	Automatically detected bands with sensitivity: Low
Lane Background	Lane background subtracted with disk size: 24.1
Lane Width	4.70 mm

Lane 7

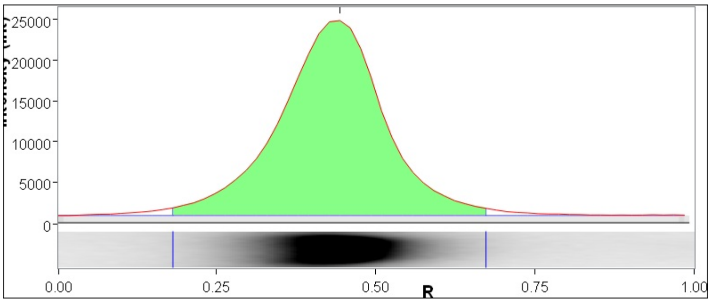

Band No.	Band Label	Mol. Wt. (KDa)	Relative Front	Adj. Volume (Int)	Volume (Int)	Abs. Quant.	Rel. Quant.	Band %	Lane %
1		N/A	0,459	12 840 594	14 096 745	N/A	N/A	100,0	97,8

Band Detection	Automatically detected bands with sensitivity: Low
Lane Background	Lane background subtracted with disk size: 24.1
Lane Width	5.09 mm

Lane 8

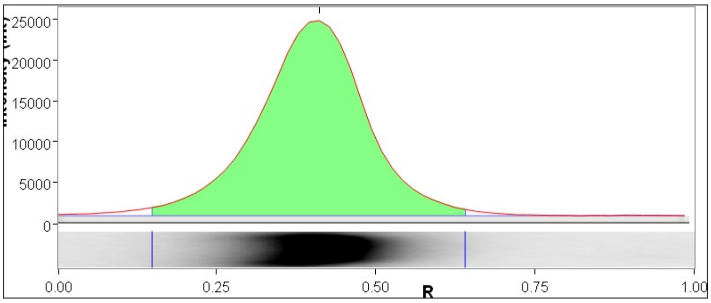

Band No.	Band Label	Mol. Wt. (KDa)	Relative Front	Adj. Volume (Int)	Volume (Int)	Abs. Quant.	Rel. Quant.	Band %	Lane %
1		N/A	0,426	12 057 042	13 158 162	N/A	N/A	100,0	97,9

Band Detection	Automatically detected bands with sensitivity: Low
Lane Background	Lane background subtracted with disk size: 24.1
Lane Width	4.83 mm

Lane 9

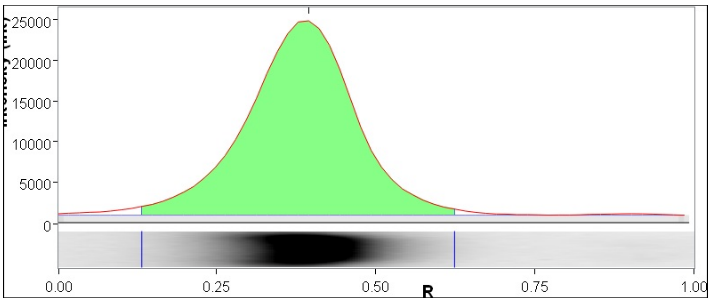

Band No.	Band Label	Mol. Wt. (KDa)	Relative Front	Adj. Volume (Int)	Volume (Int)	Abs. Quant.	Rel. Quant.	Band %	Lane %
1		N/A	0,410	11 411 628	12 542 508	N/A	N/A	100,0	97,9

Band Detection	Automatically detected bands with sensitivity: Low
Lane Background	Lane background subtracted with disk size: 24.1
Lane Width	4.96 mm

Lane 10

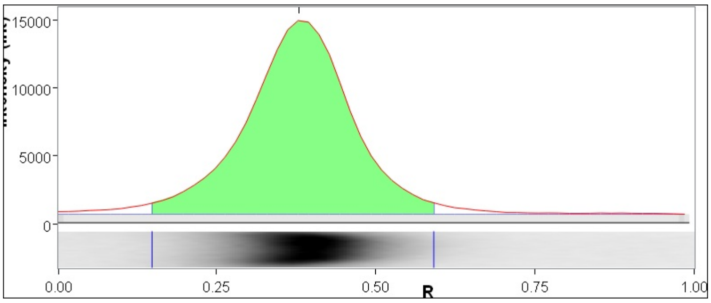

Band No.	Band Label	Mol. Wt. (KDa)	Relative Front	Adj. Volume (Int)	Volume (Int)	Abs. Quant.	Rel. Quant.	Band %	Lane %
1		N/A	0,393	7 528 955	8 325 695	N/A	N/A	100,0	96,1

Band Detection	Automatically detected bands with sensitivity: Low
Lane Background	Lane background subtracted with disk size: 24.1
Lane Width	4.57 mm

Lane 11

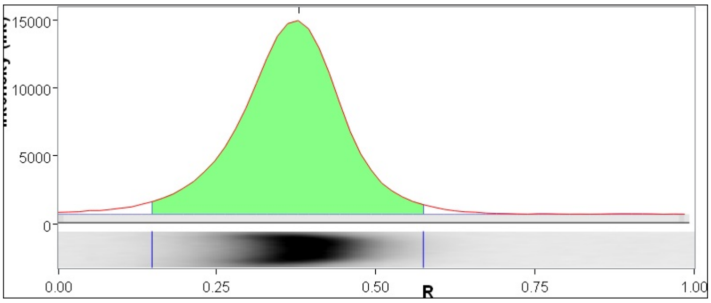

Band No.	Band Label	Mol. Wt. (KDa)	Relative Front	Adj. Volume (Int)	Volume (Int)	Abs. Quant.	Rel. Quant.	Band %	Lane %
1		N/A	0,393	7 869 086	8 684 270	N/A	N/A	100,0	96,9

Band Detection	Automatically detected bands with sensitivity: Low
Lane Background	Lane background subtracted with disk size: 24.1
Lane Width	4.83 mm

Lane 12

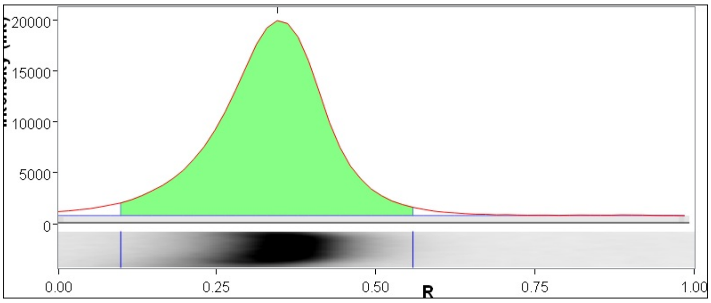

Band No.	Band Label	Mol. Wt. (KDa)	Relative Front	Adj. Volume (Int)	Volume (Int)	Abs. Quant.	Rel. Quant.	Band %	Lane %
1		N/A	0,361	8 719 674	9 499 600	N/A	N/A	100,0	96,9

Band Detection	Automatically detected bands with sensitivity: Low

Lane Background	Lane background subtracted with disk size: 24.1
Lane Width	4.44 mm

Lane 13

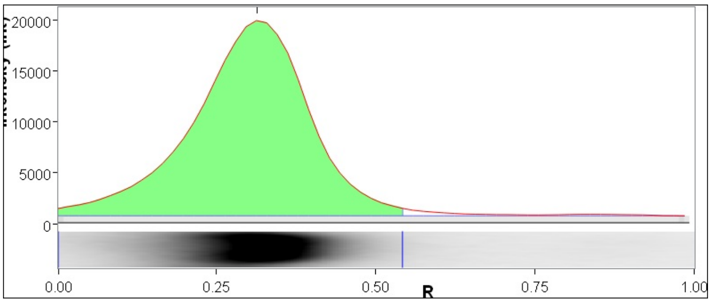

Band No.	Band Label	Mol. Wt. (KDa)	Relative Front	Adj. Volume (Int)	Volume (Int)	Abs. Quant.	Rel. Quant.	Band %	Lane %
1		N/A	0,328	10 704 456	11 693 448	N/A	N/A	100,0	98,3

Band Detection	Automatically detected bands with sensitivity: Low
Lane Background	Lane background subtracted with disk size: 24.1
Lane Width	4.70 mm

Lane 14

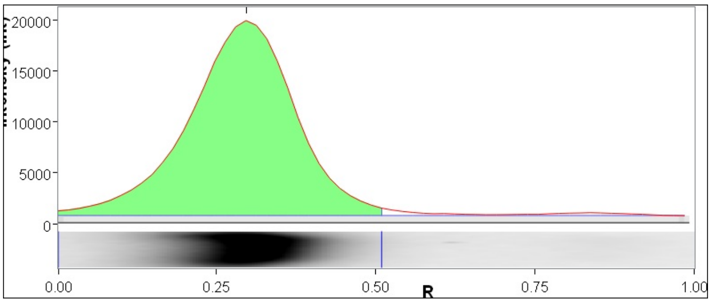

Band No.	Band Label	Mol. Wt. (KDa)	Relative Front	Adj. Volume (Int)	Volume (Int)	Abs. Quant.	Rel. Quant.	Band %	Lane %
1		N/A	0,311	9 767 376	10 726 992	N/A	N/A	100,0	97,8

Band Detection	Automatically detected bands with sensitivity: Low
Lane Background	Lane background subtracted with disk size: 24.1
Lane Width	4.70 mm

Lane 15

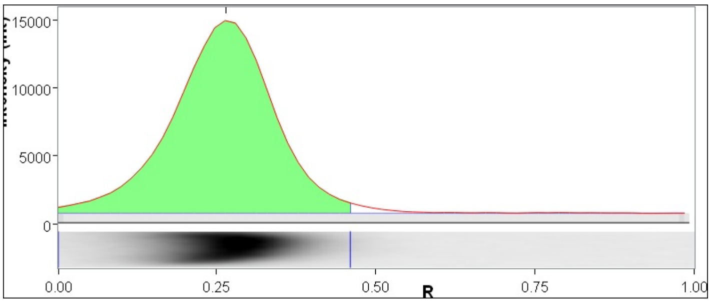

Band No.	Band Label	Mol. Wt. (KDa)	Relative Front	Adj. Volume (Int)	Volume (Int)	Abs. Quant.	Rel. Quant.	Band %	Lane %
1		N/A	0,279	6 615 396	7 445 376	N/A	N/A	100,0	98,4

Band Detection	Automatically detected bands with sensitivity: Low
Lane Background	Lane background subtracted with disk size: 24.1
Lane Width	4.70 mm

Image Report: Supplementary Fig. 4c, OSGIN1 panel

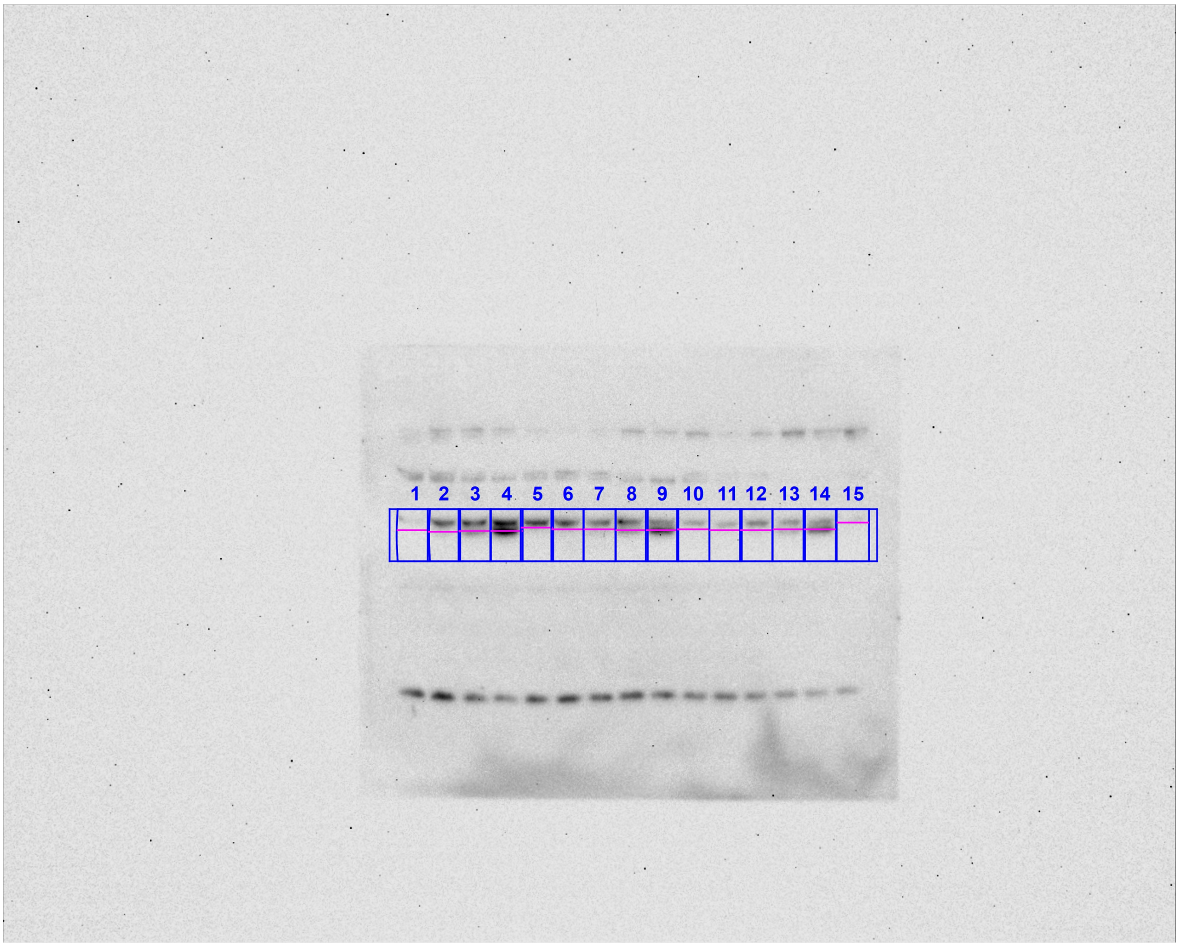

C:\Users\zahelb\OneDrive - KI.SE\Dokument\PhD\IDH2\miRNA 208b\Oxidative and reductive stress\Treating FF1-C with SF and NAC\Western blotting\ChemiDoc Images 2023-02-22_18.40.34 Osgin1\osgin1 hff1c 20230220_02\osgin1 hff1c 20230220_12.scn

Acquisition Information

Imager	ChemiDoc Touch
Exposure Time (sec)	5999.983 (Signal Accumulation)
Serial Number	732BR0263
Software Version	2.3.0.07
Application	Chemiluminescence
Excitation Source	No Illumination
Emission Filter	No Filter
Binning	2x2

Image Information

Acquisition Date	2/21/2023 3:18:31
User Name	m
Image Area (mm)	X: 180.0 Y: 144.1
Pixel Size (µm)	X: 130.5 Y: 130.5

Data Range (Int)	500 - 48336

Analysis Settings

Detection	Lane detection:<br>Manually created lanes (Copied)<br><br>Band detection:<br>Automatically detected bands with sensitivity: Low<br>Manually adjusted bands<br><br>Lane Background Subtraction:<br>Lane background subtracted with disk size: 24.1<br><br>Lane width: 4.96 mm

Lane Statistics

Lane No.	Adj. Total Band Vol. (Int)	Total Band Vol. (Int)	Adj. Total Lane Vol. (Int)	Total Lane Vol. (Int)	Bkgd. Vol. (Int)	Norm. Factor
1	41 724	318 744	525 274	2 242 798	1 717 524	N/A
2	224 086	534 546	2 050 062	3 974 914	1 924 852	N/A
3	901 626	1 289 910	3 217 802	5 069 618	1 851 816	N/A
4	3 420 418	3 950 784	6 206 578	8 140 854	1 934 276	N/A
5	355 566	915 002	2 428 162	4 468 458	2 040 296	N/A
6	287 736	762 812	2 276 960	4 380 868	2 103 908	N/A
7	406 790	938 182	1 934 238	3 993 382	2 059 144	N/A
8	746 320	1 183 092	2 740 142	4 674 418	1 934 276	N/A
9	2 224 444	2 759 332	3 583 628	5 426 020	1 842 392	N/A
10	158 574	624 910	991 724	2 798 776	1 807 052	N/A
11	136 496	561 564	826 196	2 708 640	1 882 444	N/A
12	205 618	617 918	1 365 036	3 190 936	1 825 900	N/A
13	494 418	961 970	1 425 494	3 237 258	1 811 764	N/A
14	1 445 254	1 876 174	2 577 198	4 358 334	1 781 136	N/A
15	141 284	615 524	470 364	2 308 044	1 837 680	N/A

Lane And Band Analysis

Lane 1

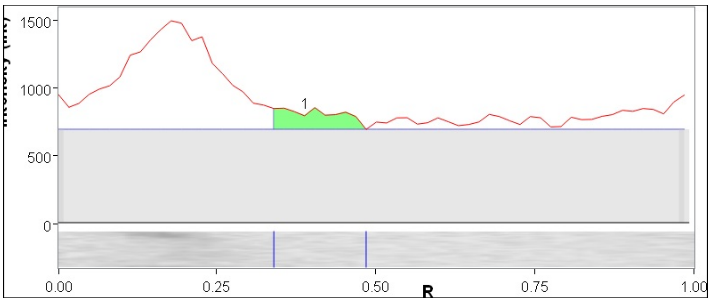

Band No.	Band Label	Mol. Wt. (KDa)	Relative Front	Adj. Volume (Int)	Volume (Int)	Abs. Quant.	Rel. Quant.	Band %	Lane %
1		N/A	0,403	41 724	318 744	N/A	N/A	100,0	7,9

Band Detection	Automatically detected bands with sensitivity: Low
Lane Background	Lane background subtracted with disk size: 24.1
Lane Width	4.96 mm

Lane 2

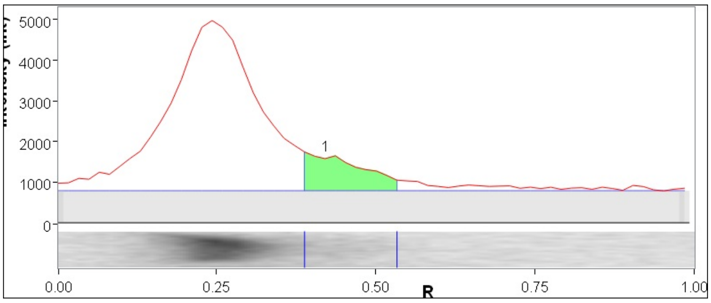

Band No.	Band Label	Mol. Wt. (KDa)	Relative Front	Adj. Volume (Int)	Volume (Int)	Abs. Quant.	Rel. Quant.	Band %	Lane %
1		N/A	0,435	224 086	534 546	N/A	N/A	100,0	10,9

Band Detection	Automatically detected bands with sensitivity: Low
Lane Background	Lane background subtracted with disk size: 24.1
Lane Width	4.96 mm

Lane 3

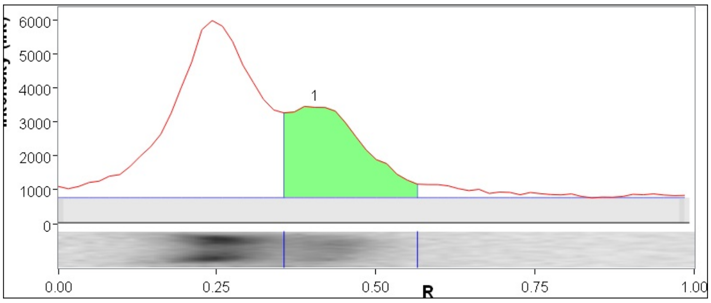

Band No.	Band Label	Mol. Wt. (KDa)	Relative Front	Adj. Volume (Int)	Volume (Int)	Abs. Quant.	Rel. Quant.	Band %	Lane %
1		N/A	0,419	901 626	1 289 910	N/A	N/A	100,0	28,0

Band Detection	Automatically detected bands with sensitivity: Low
Lane Background	Lane background subtracted with disk size: 24.1
Lane Width	4.96 mm

Lane 4

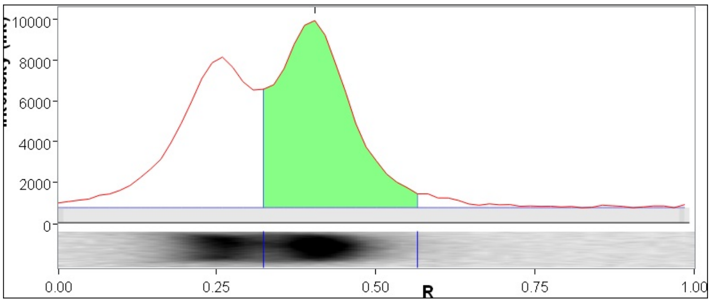

Band No.	Band Label	Mol. Wt. (KDa)	Relative Front	Adj. Volume (Int)	Volume (Int)	Abs. Quant.	Rel. Quant.	Band %	Lane %
1		N/A	0,419	3 420 418	3 950 784	N/A	N/A	100,0	55,1

Band Detection	Automatically detected bands with sensitivity: Low
Lane Background	Lane background subtracted with disk size: 24.1
Lane Width	4.96 mm

Lane 5

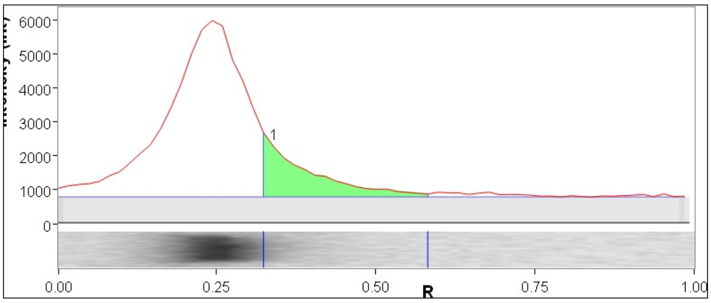

Band No.	Band Label	Mol. Wt. (KDa)	Relative Front	Adj. Volume (Int)	Volume (Int)	Abs. Quant.	Rel. Quant.	Band %	Lane %
1		N/A	0,355	355 566	915 002	N/A	N/A	100,0	14,6

Band Detection	Automatically detected bands with sensitivity: Low
Lane Background	Lane background subtracted with disk size: 24.1
Lane Width	4.96 mm

Lane 6

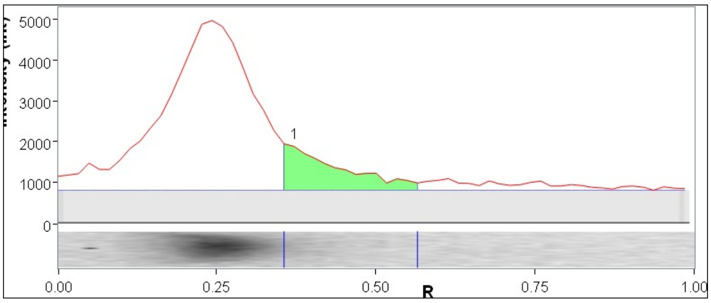

Band No.	Band Label	Mol. Wt. (KDa)	Relative Front	Adj. Volume (Int)	Volume (Int)	Abs. Quant.	Rel. Quant.	Band %	Lane %
1		N/A	0,387	287 736	762 812	N/A	N/A	100,0	12,6

Band Detection	Automatically detected bands with sensitivity: Low
Lane Background	Lane background subtracted with disk size: 24.1
Lane Width	4.96 mm

Lane 7

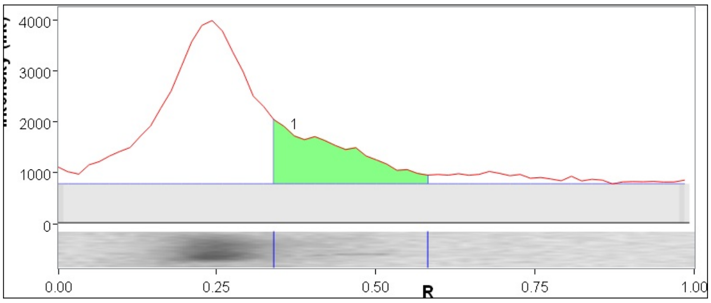

Band No.	Band Label	Mol. Wt. (KDa)	Relative Front	Adj. Volume (Int)	Volume (Int)	Abs. Quant.	Rel. Quant.	Band %	Lane %
1		N/A	0,387	406 790	938 182	N/A	N/A	100,0	21,0

Band Detection	Automatically detected bands with sensitivity: Low
Lane Background	Lane background subtracted with disk size: 24.1
Lane Width	4.96 mm

Lane 8

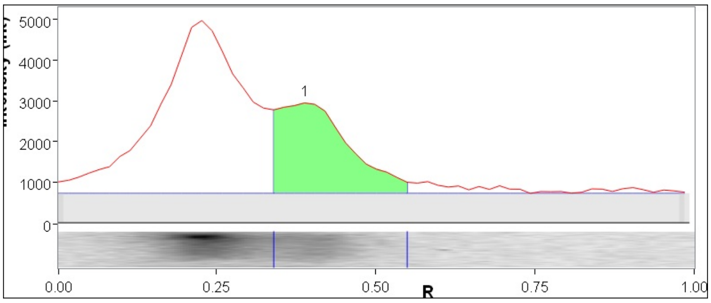

Band No.	Band Label	Mol. Wt. (KDa)	Relative Front	Adj. Volume (Int)	Volume (Int)	Abs. Quant.	Rel. Quant.	Band %	Lane %
1		N/A	0,403	746 320	1 183 092	N/A	N/A	100,0	27,2

Band Detection	Automatically detected bands with sensitivity: Low
Lane Background	Lane background subtracted with disk size: 24.1
Lane Width	4.96 mm

Lane 9

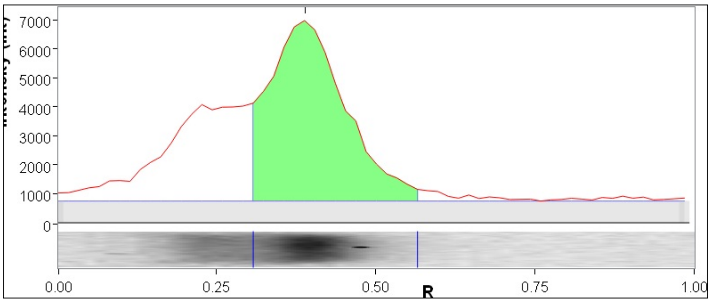

Band No.	Band Label	Mol. Wt. (KDa)	Relative Front	Adj. Volume (Int)	Volume (Int)	Abs. Quant.	Rel. Quant.	Band %	Lane %
1		N/A	0,403	2 224 444	2 759 332	N/A	N/A	100,0	62,1

Band Detection	Automatically detected bands with sensitivity: Low
Lane Background	Lane background subtracted with disk size: 24.1
Lane Width	4.96 mm

Lane 10

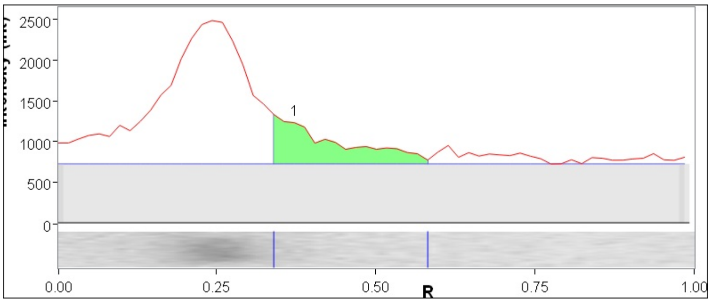

Band No.	Band Label	Mol. Wt. (KDa)	Relative Front	Adj. Volume (Int)	Volume (Int)	Abs. Quant.	Rel. Quant.	Band %	Lane %
1		N/A	0,387	158 574	624 910	N/A	N/A	100,0	16,0

Band Detection	Automatically detected bands with sensitivity: Low
Lane Background	Lane background subtracted with disk size: 24.1
Lane Width	4.96 mm

Lane 11

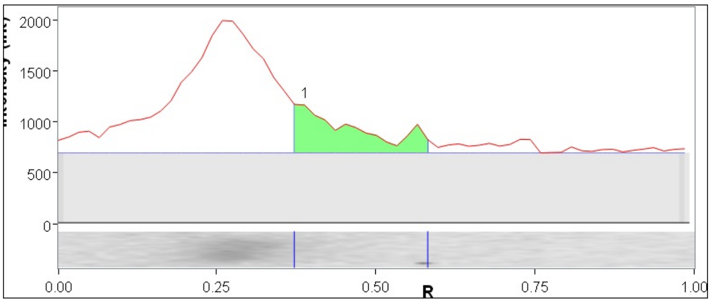

Band No.	Band Label	Mol. Wt. (KDa)	Relative Front	Adj. Volume (Int)	Volume (Int)	Abs. Quant.	Rel. Quant.	Band %	Lane %
1		N/A	0,403	136 496	561 564	N/A	N/A	100,0	16,5

Band Detection	Automatically detected bands with sensitivity: Low
Lane Background	Lane background subtracted with disk size: 24.1
Lane Width	4.96 mm

Lane 12

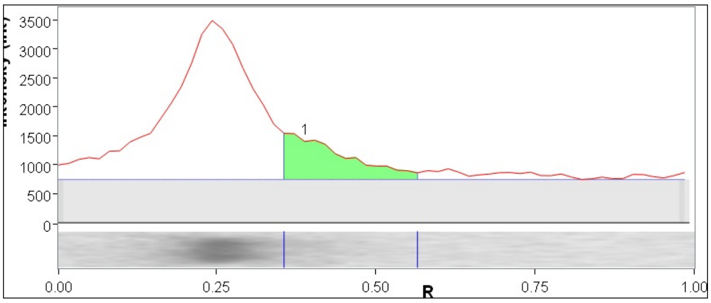

Band No.	Band Label	Mol. Wt. (KDa)	Relative Front	Adj. Volume (Int)	Volume (Int)	Abs. Quant.	Rel. Quant.	Band %	Lane %
1		N/A	0,403	205 618	617 918	N/A	N/A	100,0	15,1

Band Detection	Automatically detected bands with sensitivity: Low

Lane Background	Lane background subtracted with disk size: 24.1
Lane Width	4.96 mm

Lane 13

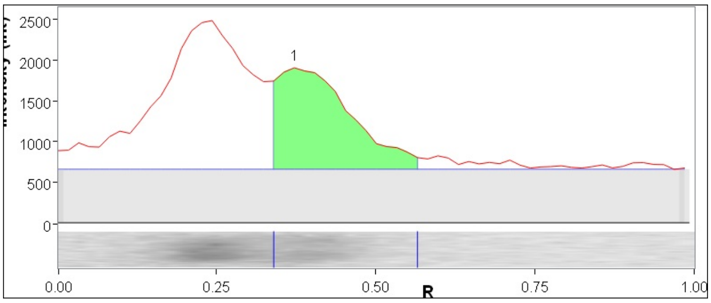

Band No.	Band Label	Mol. Wt. (KDa)	Relative Front	Adj. Volume (Int)	Volume (Int)	Abs. Quant.	Rel. Quant.	Band %	Lane %
1		N/A	0,387	494 418	961 970	N/A	N/A	100,0	34,7

Band Detection	Automatically detected bands with sensitivity: Low
Lane Background	Lane background subtracted with disk size: 24.1
Lane Width	4.96 mm

Lane 14

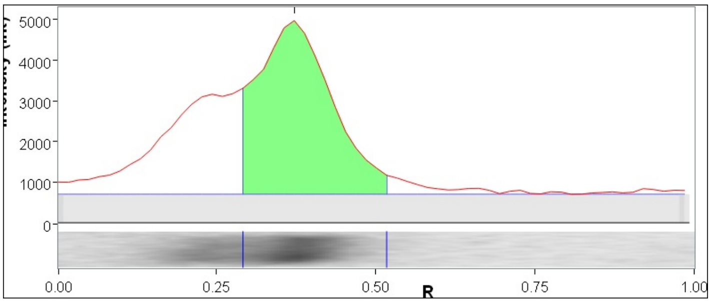

Band No.	Band Label	Mol. Wt. (KDa)	Relative Front	Adj. Volume (Int)	Volume (Int)	Abs. Quant.	Rel. Quant.	Band %	Lane %
1		N/A	0,387	1 445 254	1 876 174	N/A	N/A	100,0	56,1

Band Detection	Automatically detected bands with sensitivity: Low
Lane Background	Lane background subtracted with disk size: 24.1
Lane Width	4.96 mm

Lane 15

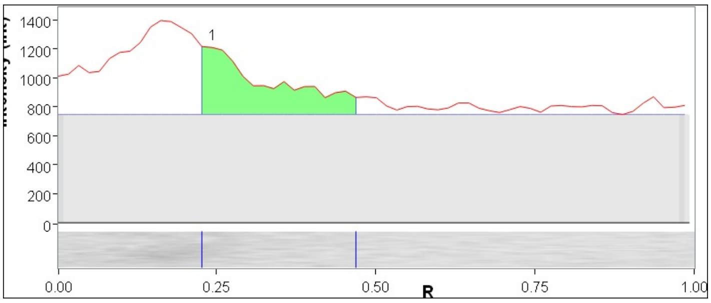

Band No.	Band Label	Mol. Wt. (KDa)	Relative Front	Adj. Volume (Int)	Volume (Int)	Abs. Quant.	Rel. Quant.	Band %	Lane %
1		N/A	0,258	141 284	615 524	N/A	N/A	100,0	30,0

Band Detection	Automatically detected bands with sensitivity: Low
Lane Background	Lane background subtracted with disk size: 24.1
Lane Width	4.96 mm

Image Report: Supplementary Fig. 4c, caspae 3

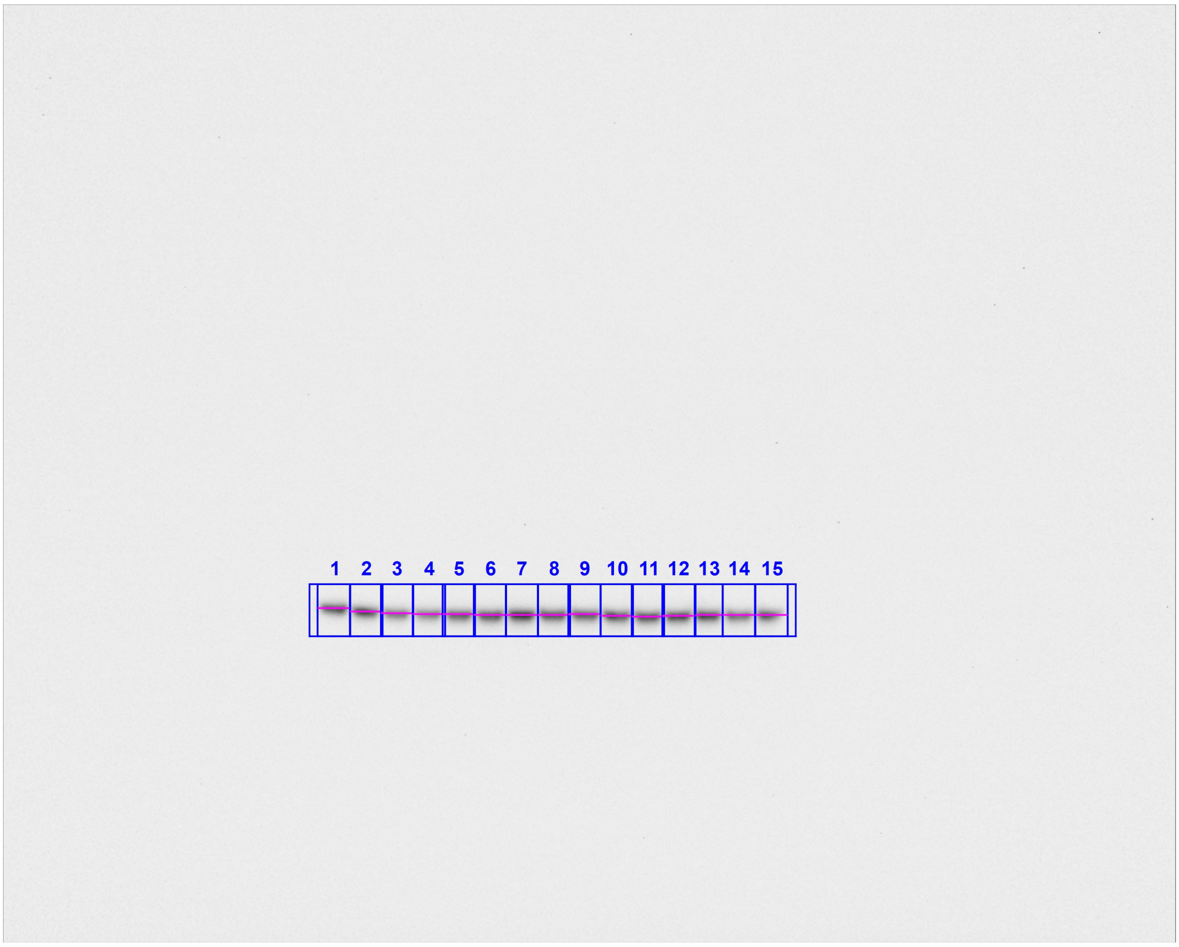

Acquisition Information

Imager	ChemiDoc Touch
Exposure Time (sec)	100.000 (Signal Accumulation)
Serial Number	732BR0263
Software Version	2.3.0.07
Application	Chemiluminescence
Excitation Source	No Illumination
Emission Filter	No Filter
Binning	2x2

Image Information

Acquisition Date	2/22/2023 8:56:18
User Name	m
Image Area (mm)	X: 180.0 Y: 144.1
Pixel Size (µm)	X: 130.5 Y: 130.5

Data Range (Int)	500 - 16113

Analysis Settings

Detection	Lane detection:<br>Manually created lanes (Copied)<br><br>Band detection:<br>Bands detected with different sensitivity per lane<br>Manually adjusted bands<br><br>Lane Background Subtraction:<br>Lane background subtracted with disk size: 24.1<br><br>Lane width: Variable

Lane Statistics

Lane No.	Adj. Total Band Vol. (Int)	Total Band Vol. (Int)	Adj. Total Lane Vol. (Int)	Total Lane Vol. (Int)	Bkgd. Vol. (Int)	Norm. Factor
1	3 637 170	4 328 124	3 722 328	5 175 714	1 453 386	N/A
2	3 762 722	4 431 636	3 890 098	5 297 124	1 407 026	N/A
3	2 644 534	3 284 758	2 760 054	4 206 486	1 446 432	N/A
4	2 609 308	3 252 610	2 754 392	4 207 778	1 453 386	N/A
5	3 543 196	4 283 056	3 651 800	5 156 182	1 504 382	N/A
6	4 065 734	4 837 324	4 185 966	5 704 256	1 518 290	N/A
7	5 071 328	5 866 592	5 201 326	6 717 298	1 515 972	N/A
8	4 140 176	4 903 520	4 272 492	5 774 556	1 502 064	N/A
9	3 768 650	4 510 790	3 878 888	5 387 906	1 509 018	N/A
10	4 136 642	4 885 850	4 260 750	5 734 998	1 474 248	N/A
11	4 064 062	4 814 448	4 197 176	5 673 742	1 476 566	N/A
12	4 260 294	5 020 104	4 405 568	5 900 678	1 495 110	N/A
13	3 877 344	4 524 128	3 989 216	5 261 920	1 272 704	N/A
14	3 067 398	3 742 924	3 198 232	4 619 166	1 420 934	N/A
15	3 105 626	3 780 050	3 238 968	4 657 584	1 418 616	N/A

Lane And Band Analysis

Lane 1

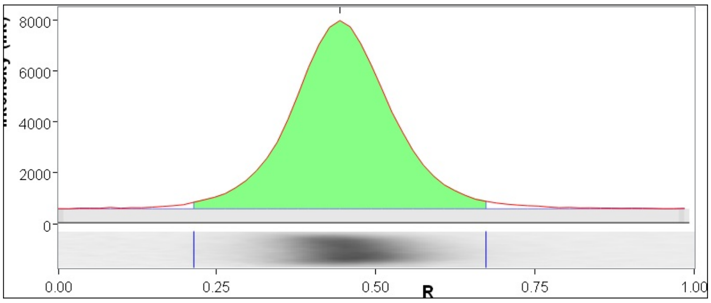

Band No.	Band Label	Mol. Wt. (KDa)	Relative Front	Adj. Volume (Int)	Volume (Int)	Abs. Quant.	Rel. Quant.	Band %	Lane %
1		N/A	0,459	3 637 170	4 328 124	N/A	N/A	100,0	97,7

Band Detection	Automatically detected bands with sensitivity: Low
Lane Background	Lane background subtracted with disk size: 24.1
Lane Width	4.96 mm

Lane 2

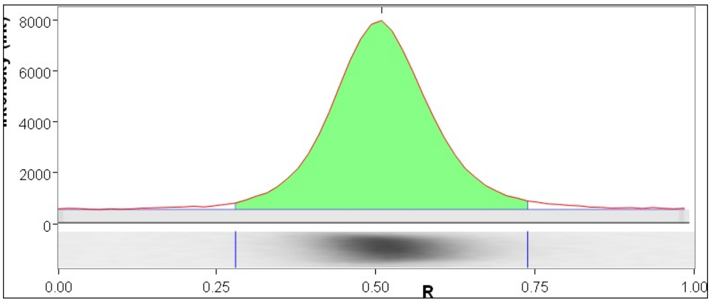

Band No.	Band Label	Mol. Wt. (KDa)	Relative Front	Adj. Volume (Int)	Volume (Int)	Abs. Quant.	Rel. Quant.	Band %	Lane %
1		N/A	0,525	3 762 722	4 431 636	N/A	N/A	100,0	96,7

Band Detection	Automatically detected bands with sensitivity: Low
Lane Background	Lane background subtracted with disk size: 24.1
Lane Width	4.96 mm

Lane 3

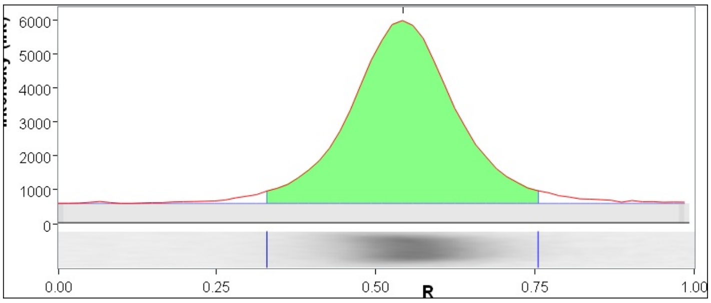

Band No.	Band Label	Mol. Wt. (KDa)	Relative Front	Adj. Volume (Int)	Volume (Int)	Abs. Quant.	Rel. Quant.	Band %	Lane %
1		N/A	0,557	2 644 534	3 284 758	N/A	N/A	100,0	95,8

Band Detection	Automatically detected bands with sensitivity: Low
Lane Background	Lane background subtracted with disk size: 24.1
Lane Width	4.96 mm

Lane 4

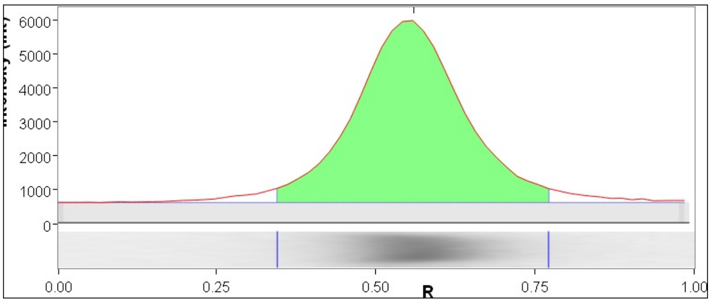

Band No.	Band Label	Mol. Wt. (KDa)	Relative Front	Adj. Volume (Int)	Volume (Int)	Abs. Quant.	Rel. Quant.	Band %	Lane %
1		N/A	0,574	2 609 308	3 252 610	N/A	N/A	100,0	94,7

Band Detection	Automatically detected bands with sensitivity: Low
Lane Background	Lane background subtracted with disk size: 24.1
Lane Width	4.96 mm

Lane 5

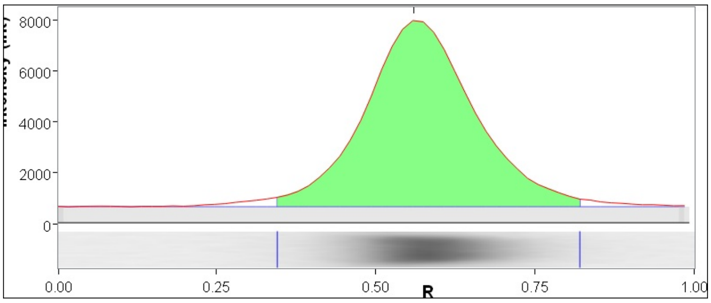

Band No.	Band Label	Mol. Wt. (KDa)	Relative Front	Adj. Volume (Int)	Volume (Int)	Abs. Quant.	Rel. Quant.	Band %	Lane %
1		N/A	0,574	3 543 196	4 283 056	N/A	N/A	100,0	97,0

Band Detection	Automatically detected bands with sensitivity: Low
Lane Background	Lane background subtracted with disk size: 24.1
Lane Width	4.96 mm

Lane 6

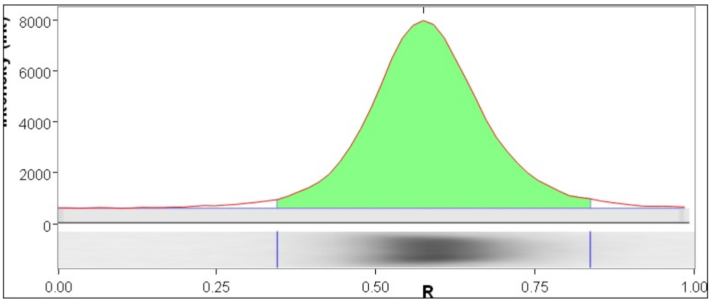

Band No.	Band Label	Mol. Wt. (KDa)	Relative Front	Adj. Volume (Int)	Volume (Int)	Abs. Quant.	Rel. Quant.	Band %	Lane %
1		N/A	0,590	4 065 734	4 837 324	N/A	N/A	100,0	97,1

Lane Background	Lane background subtracted with disk size: 24.1
Lane Width	4.96 mm

Lane 7

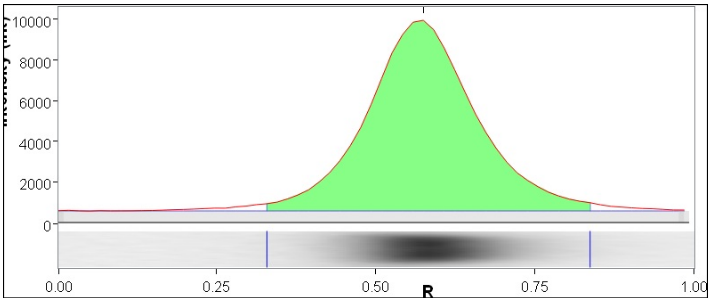

Band No.	Band Label	Mol. Wt. (KDa)	Relative Front	Adj. Volume (Int)	Volume (Int)	Abs. Quant.	Rel. Quant.	Band %	Lane %
1		N/A	0,590	5 071 328	5 866 592	N/A	N/A	100,0	97,5

Band Detection	Automatically detected bands with sensitivity: Low
Lane Background	Lane background subtracted with disk size: 24.1
Lane Width	4.96 mm

Lane 8

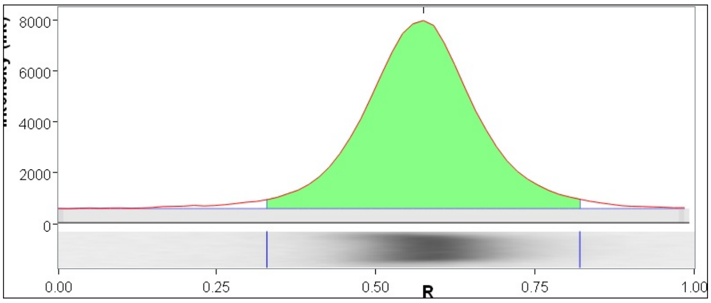

Band No.	Band Label	Mol. Wt. (KDa)	Relative Front	Adj. Volume (Int)	Volume (Int)	Abs. Quant.	Rel. Quant.	Band %	Lane %
1		N/A	0,590	4 140 176	4 903 520	N/A	N/A	100,0	96,9

Band Detection	Automatically detected bands with sensitivity: Low
Lane Background	Lane background subtracted with disk size: 24.1
Lane Width	4.96 mm

Lane 9

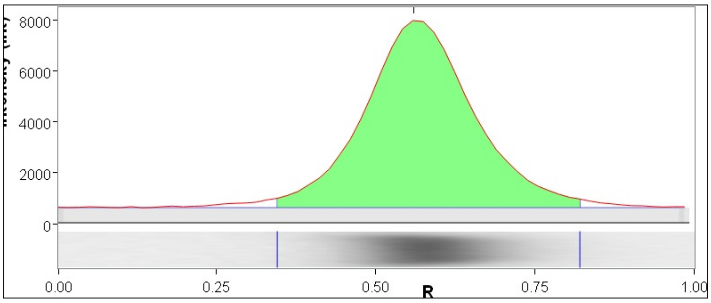

Band No.	Band Label	Mol. Wt. (KDa)	Relative Front	Adj. Volume (Int)	Volume (Int)	Abs. Quant.	Rel. Quant.	Band %	Lane %
1		N/A	0,574	3 768 650	4 510 790	N/A	N/A	100,0	97,2

Band Detection	Automatically detected bands with sensitivity: Low
Lane Background	Lane background subtracted with disk size: 24.1
Lane Width	4.96 mm

Lane 10

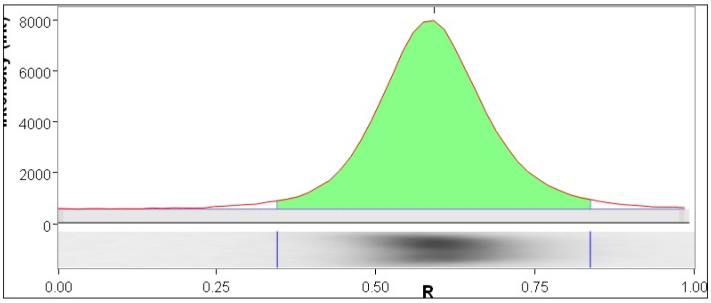

Band No.	Band Label	Mol. Wt. (KDa)	Relative Front	Adj. Volume (Int)	Volume (Int)	Abs. Quant.	Rel. Quant.	Band %	Lane %
1		N/A	0,607	4 136 642	4 885 850	N/A	N/A	100,0	97,1

Band Detection	Automatically detected bands with sensitivity: Low
Lane Background	Lane background subtracted with disk size: 24.1
Lane Width	4.96 mm

Lane 11

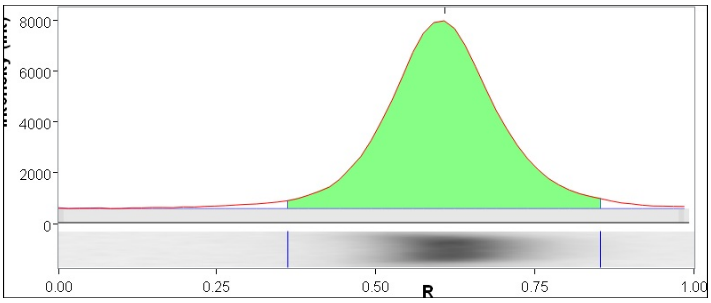

Band No.	Band Label	Mol. Wt. (KDa)	Relative Front	Adj. Volume (Int)	Volume (Int)	Abs. Quant.	Rel. Quant.	Band %	Lane %
1		N/A	0,623	4 064 062	4 814 448	N/A	N/A	100,0	96,8

Band Detection	Automatically detected bands with sensitivity: Low
Lane Background	Lane background subtracted with disk size: 24.1
Lane Width	4.96 mm

Lane 12

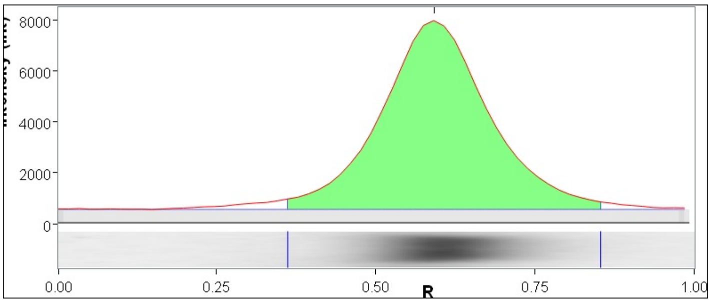

Band No.	Band Label	Mol. Wt. (KDa)	Relative Front	Adj. Volume (Int)	Volume (Int)	Abs. Quant.	Rel. Quant.	Band %	Lane %
1		N/A	0,607	4 260 294	5 020 104	N/A	N/A	100,0	96,7

Band Detection	Automatically detected bands with sensitivity: Low

Lane Background	Lane background subtracted with disk size: 24.1
Lane Width	4.96 mm

Lane 13

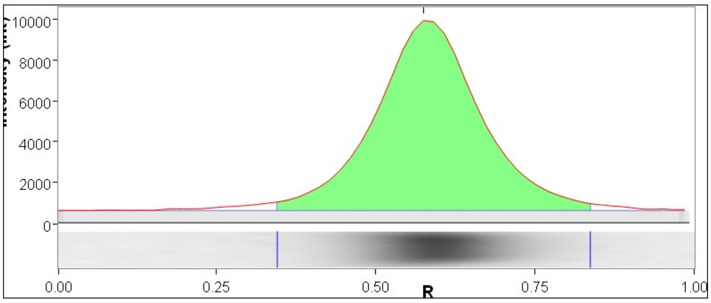

Band No.	Band Label	Mol. Wt. (KDa)	Relative Front	Adj. Volume (Int)	Volume (Int)	Abs. Quant.	Rel. Quant.	Band %	Lane %
1		N/A	0,590	3 877 344	4 524 128	N/A	N/A	100,0	97,2

Band Detection	Automatically detected bands with sensitivity: Low
Lane Background	Lane background subtracted with disk size: 24.1
Lane Width	4.18 mm

Lane 14

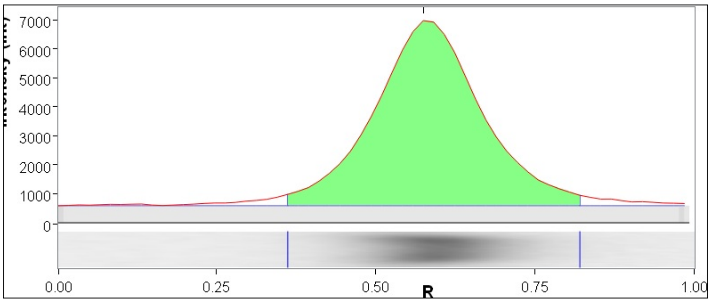

Band No.	Band Label	Mol. Wt. (KDa)	Relative Front	Adj. Volume (Int)	Volume (Int)	Abs. Quant.	Rel. Quant.	Band %	Lane %
1		N/A	0,590	3 067 398	3 742 924	N/A	N/A	100,0	95,9

Band Detection	Automatically detected bands with sensitivity: Low
Lane Background	Lane background subtracted with disk size: 24.1
Lane Width	4.96 mm

Lane 15

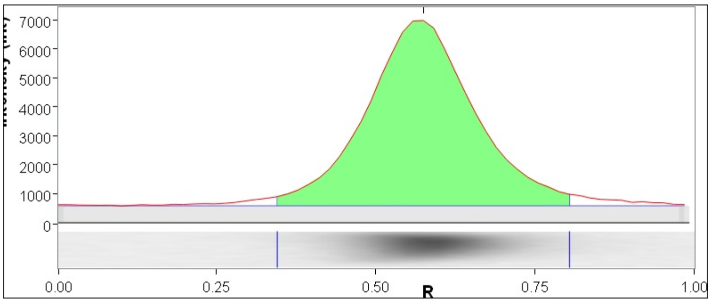

Band No.	Band Label	Mol. Wt. (KDa)	Relative Front	Adj. Volume (Int)	Volume (Int)	Abs. Quant.	Rel. Quant.	Band %	Lane %
1		N/A	0,590	3 105 626	3 780 050	N/A	N/A	100,0	95,9

Band Detection	Automatically detected bands with sensitivity: Low
Lane Background	Lane background subtracted with disk size: 24.1
Lane Width	4.96 mm

Image Report: Supplementary Fig. 4c, Gapdh panel

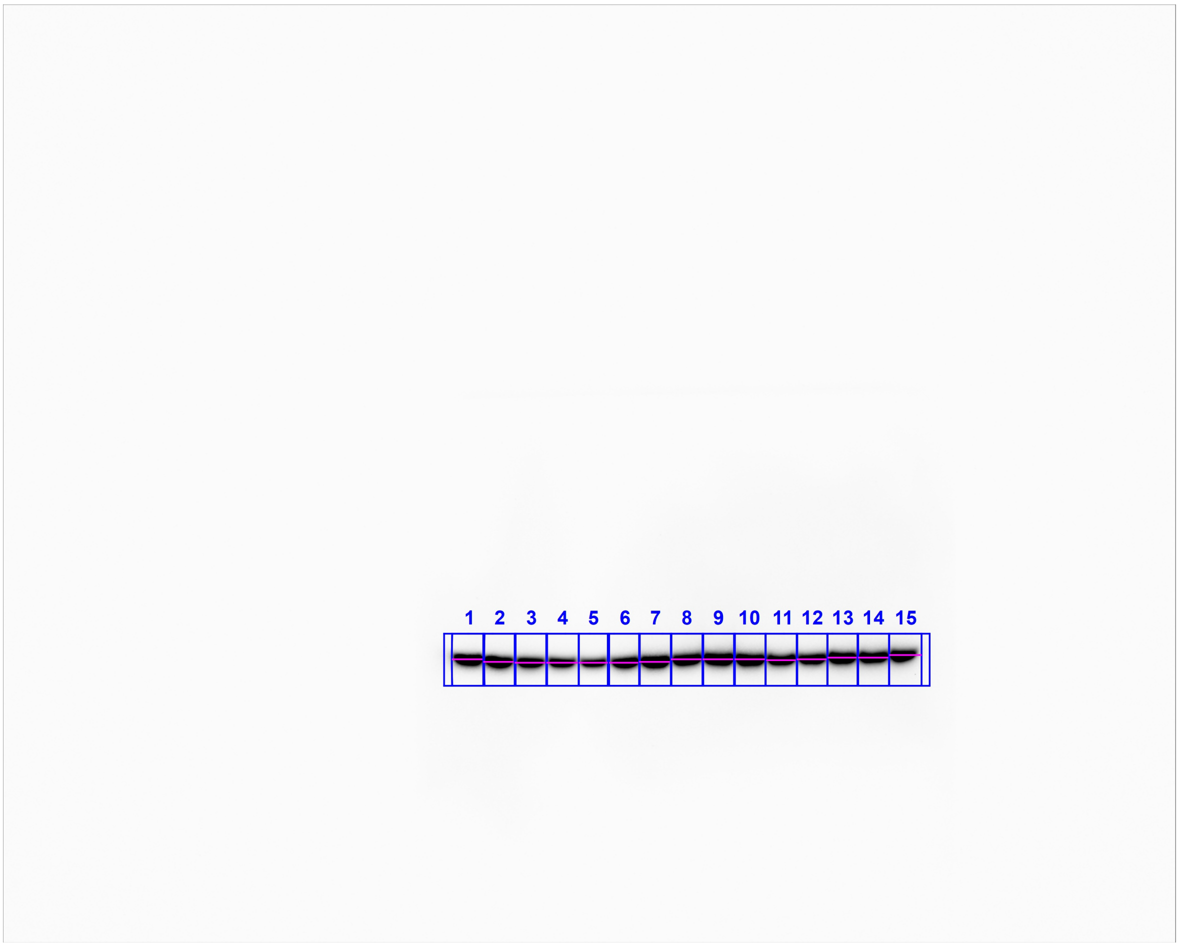

Acquisition Information

Imager	ChemiDoc Touch
Exposure Time (sec)	535.565 (Optimal Auto-exposure)
Serial Number	732BR0263
Software Version	2.3.0.07
Application	Chemiluminescence
Excitation Source	No Illumination
Emission Filter	No Filter
Binning	2x2

Image Information

Acquisition Date	2/24/2023 1:07:36
User Name	m
Image Area (mm)	X: 180.0 Y: 144.1
Pixel Size (µm)	X: 130.5 Y: 130.5

Data Range (Int)	500 - 64458

Analysis Settings

Detection	Lane detection:<br>Manually created lanes (Copied)<br><br>Band detection:<br>Automatically detected bands with sensitivity: Low<br><br>Lane Background Subtraction:<br>Lane background subtracted with disk size: 24.1<br><br>Lane width: Variable

Lane Statistics

Lane No.	Adj. Total Band Vol. (Int)	Total Band Vol. (Int)	Adj. Total Lane Vol. (Int)	Total Lane Vol. (Int)	Bkgd. Vol. (Int)	Norm. Factor
1	21 156 424	22 345 026	21 576 058	23 914 920	2 338 862	N/A
2	20 716 536	22 054 136	21 155 702	23 705 502	2 549 800	N/A
3	16 232 574	17 385 836	16 676 984	18 946 306	2 269 322	N/A
4	13 539 666	14 534 772	13 931 066	16 024 220	2 093 154	N/A
5	10 778 102	11 527 190	11 166 144	12 858 528	1 692 384	N/A
6	16 969 500	18 083 268	17 445 636	19 637 244	2 191 608	N/A
7	23 567 676	25 021 176	24 145 542	26 753 292	2 607 750	N/A
8	20 446 774	21 870 558	21 213 500	23 767 936	2 554 436	N/A
9	24 164 428	25 751 118	24 998 186	27 763 560	2 765 374	N/A
10	22 579 065	24 262 565	23 231 782	26 165 882	2 934 100	N/A
11	17 274 449	18 808 913	17 852 685	20 777 757	2 925 072	N/A
12	16 809 876	18 173 844	17 374 464	19 974 528	2 600 064	N/A
13	20 698 416	22 392 432	21 308 832	24 348 096	3 039 264	N/A
14	19 496 114	21 243 365	20 218 428	23 448 195	3 229 767	N/A
15	18 109 090	19 825 816	18 708 236	21 881 578	3 173 342	N/A

Lane And Band Analysis

Lane 1

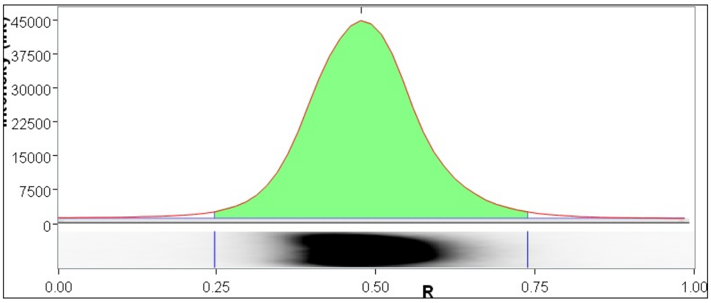

Band No.	Band Label	Mol. Wt. (KDa)	Relative Front	Adj. Volume (Int)	Volume (Int)	Abs. Quant.	Rel. Quant.	Band %	Lane %
1		N/A	0,492	21 156 424	22 345 026	N/A	N/A	100,0	98,1

Band Detection	Automatically detected bands with sensitivity: Low
Lane Background	Lane background subtracted with disk size: 24.1
Lane Width	4.96 mm

Lane 2

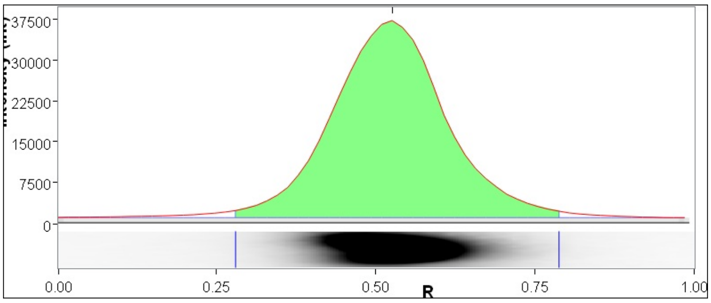

Band No.	Band Label	Mol. Wt. (KDa)	Relative Front	Adj. Volume (Int)	Volume (Int)	Abs. Quant.	Rel. Quant.	Band %	Lane %
1		N/A	0,541	20 716 536	22 054 136	N/A	N/A	100,0	97,9

Band Detection	Automatically detected bands with sensitivity: Low
Lane Background	Lane background subtracted with disk size: 24.1
Lane Width	4.96 mm

Lane 3

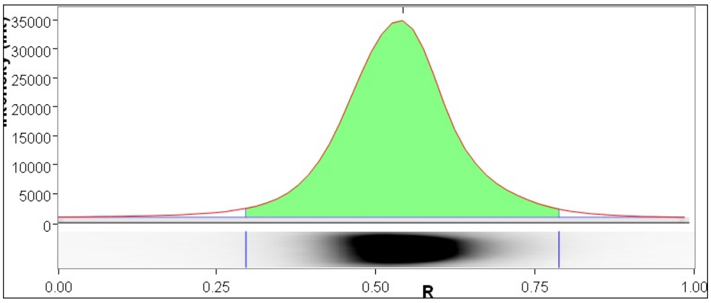

Band No.	Band Label	Mol. Wt. (KDa)	Relative Front	Adj. Volume (Int)	Volume (Int)	Abs. Quant.	Rel. Quant.	Band %	Lane %
1		N/A	0,557	16 232 574	17 385 836	N/A	N/A	100,0	97,3

Band Detection	Automatically detected bands with sensitivity: Low
Lane Background	Lane background subtracted with disk size: 24.1
Lane Width	4.96 mm

Lane 4

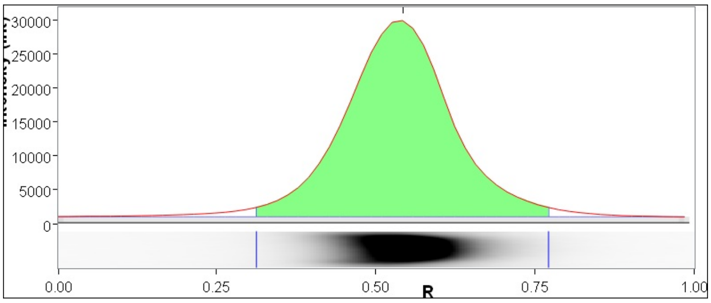

Band No.	Band Label	Mol. Wt. (KDa)	Relative Front	Adj. Volume (Int)	Volume (Int)	Abs. Quant.	Rel. Quant.	Band %	Lane %
1		N/A	0,557	13 539 666	14 534 772	N/A	N/A	100,0	97,2

Band Detection	Automatically detected bands with sensitivity: Low
Lane Background	Lane background subtracted with disk size: 24.1
Lane Width	4.96 mm

Lane 5

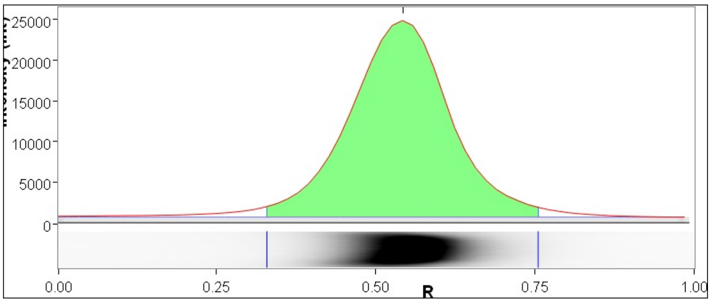

Band No.	Band Label	Mol. Wt. (KDa)	Relative Front	Adj. Volume (Int)	Volume (Int)	Abs. Quant.	Rel. Quant.	Band %	Lane %
1		N/A	0,557	10 778 102	11 527 190	N/A	N/A	100,0	96,5

Band Detection	Automatically detected bands with sensitivity: Low
Lane Background	Lane background subtracted with disk size: 24.1
Lane Width	4.44 mm

Lane 6

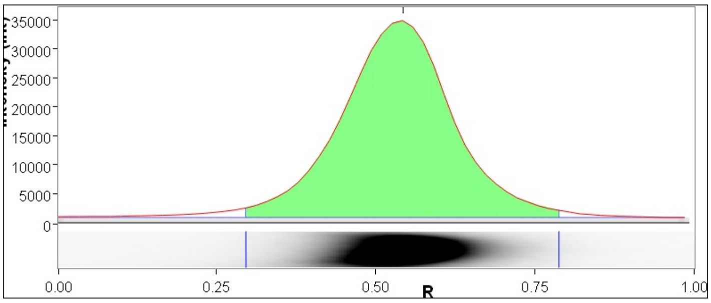

Band No.	Band Label	Mol. Wt. (KDa)	Relative Front	Adj. Volume (Int)	Volume (Int)	Abs. Quant.	Rel. Quant.	Band %	Lane %
1		N/A	0,557	16 969 500	18 083 268	N/A	N/A	100,0	97,3

Band Detection	Automatically detected bands with sensitivity: Low
Lane Background	Lane background subtracted with disk size: 24.1
Lane Width	4.70 mm

Lane 7

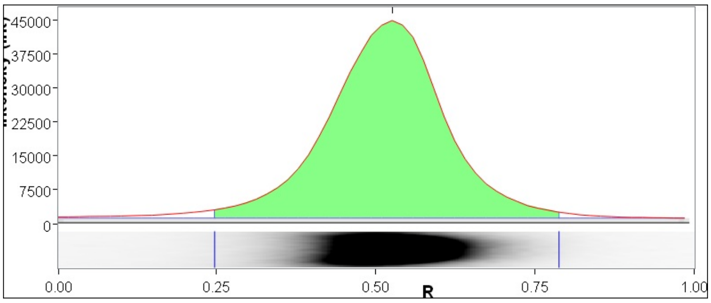

Band No.	Band Label	Mol. Wt. (KDa)	Relative Front	Adj. Volume (Int)	Volume (Int)	Abs. Quant.	Rel. Quant.	Band %	Lane %
1		N/A	0,541	23 567 676	25 021 176	N/A	N/A	100,0	97,6

Band Detection	Automatically detected bands with sensitivity: Low
Lane Background	Lane background subtracted with disk size: 24.1
Lane Width	4.96 mm

Lane 8

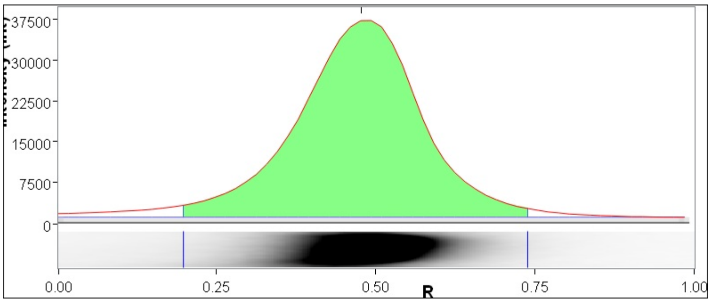

Band No.	Band Label	Mol. Wt. (KDa)	Relative Front	Adj. Volume (Int)	Volume (Int)	Abs. Quant.	Rel. Quant.	Band %	Lane %
1		N/A	0,492	20 446 774	21 870 558	N/A	N/A	100,0	96,4

Band Detection	Automatically detected bands with sensitivity: Low
Lane Background	Lane background subtracted with disk size: 24.1
Lane Width	4.96 mm

Lane 9

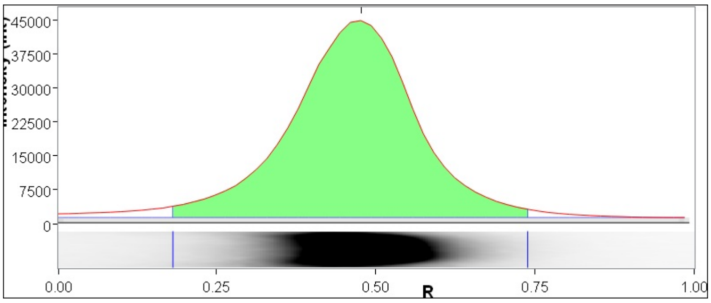

Band No.	Band Label	Mol. Wt. (KDa)	Relative Front	Adj. Volume (Int)	Volume (Int)	Abs. Quant.	Rel. Quant.	Band %	Lane %
1		N/A	0,492	24 164 428	25 751 118	N/A	N/A	100,0	96,7

Band Detection	Automatically detected bands with sensitivity: Low
Lane Background	Lane background subtracted with disk size: 24.1
Lane Width	4.96 mm

Lane 10

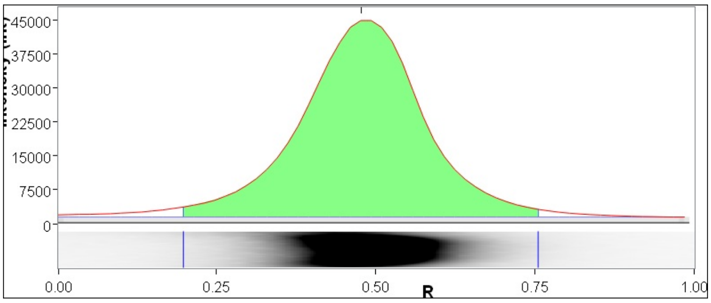

Band No.	Band Label	Mol. Wt. (KDa)	Relative Front	Adj. Volume (Int)	Volume (Int)	Abs. Quant.	Rel. Quant.	Band %	Lane %
1		N/A	0,492	22 579 065	24 262 565	N/A	N/A	100,0	97,2

Band Detection	Automatically detected bands with sensitivity: Low
Lane Background	Lane background subtracted with disk size: 24.1
Lane Width	4.83 mm

Lane 11

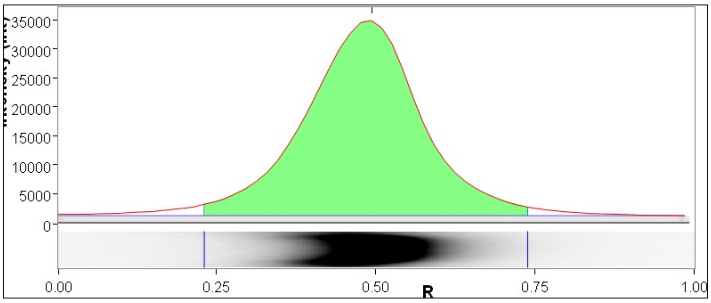

Band No.	Band Label	Mol. Wt. (KDa)	Relative Front	Adj. Volume (Int)	Volume (Int)	Abs. Quant.	Rel. Quant.	Band %	Lane %
1		N/A	0,508	17 274 449	18 808 913	N/A	N/A	100,0	96,8

Band Detection	Automatically detected bands with sensitivity: Low
Lane Background	Lane background subtracted with disk size: 24.1
Lane Width	4.83 mm

Lane 12

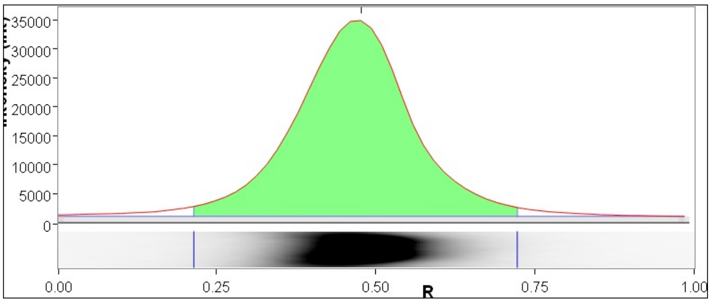

Band No.	Band Label	Mol. Wt. (KDa)	Relative Front	Adj. Volume (Int)	Volume (Int)	Abs. Quant.	Rel. Quant.	Band %	Lane %
1		N/A	0,492	16 809 876	18 173 844	N/A	N/A	100,0	96,8

Band Detection	Automatically detected bands with sensitivity: Low

Lane Background	Lane background subtracted with disk size: 24.1
Lane Width	4.70 mm

Lane 13

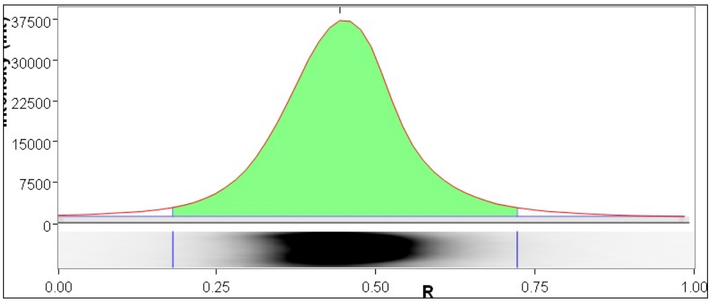

Band No.	Band Label	Mol. Wt. (KDa)	Relative Front	Adj. Volume (Int)	Volume (Int)	Abs. Quant.	Rel. Quant.	Band %	Lane %
1		N/A	0,459	20 698 416	22 392 432	N/A	N/A	100,0	97,1

Band Detection	Automatically detected bands with sensitivity: Low
Lane Background	Lane background subtracted with disk size: 24.1
Lane Width	4.70 mm

Lane 14

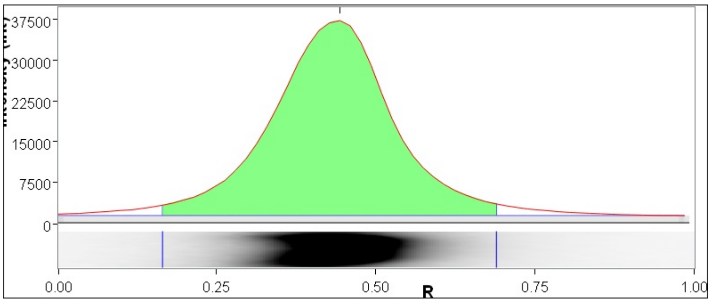

Band No.	Band Label	Mol. Wt. (KDa)	Relative Front	Adj. Volume (Int)	Volume (Int)	Abs. Quant.	Rel. Quant.	Band %	Lane %
1		N/A	0,459	19 496 114	21 243 365	N/A	N/A	100,0	96,4

Band Detection	Automatically detected bands with sensitivity: Low
Lane Background	Lane background subtracted with disk size: 24.1
Lane Width	4.83 mm

Lane 15

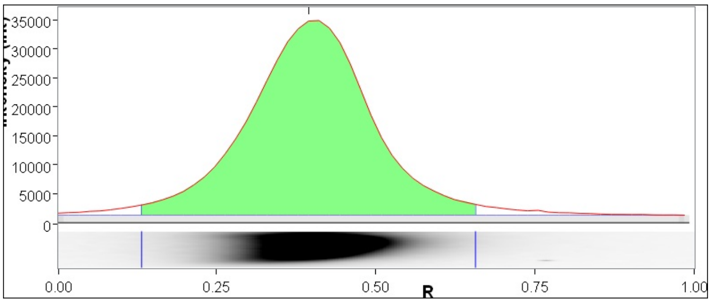

Band No.	Band Label	Mol. Wt. (KDa)	Relative Front	Adj. Volume (Int)	Volume (Int)	Abs. Quant.	Rel. Quant.	Band %	Lane %
1		N/A	0,410	18 109 090	19 825 816	N/A	N/A	100,0	96,8

Band Detection	Automatically detected bands with sensitivity: Low
Lane Background	Lane background subtracted with disk size: 24.1
Lane Width	4.96 mm
