# Supplementary material for: Epigenetic modulators link mitochondrial redox homeostasis to cardiac function in a sex-dependent manner
Source: Nat Commun. 2024 Mar 20;15:2358. doi: 10.1038/s41467-024-46384-8 (PMC10954618; doi:10.1038/s41467-024-46384-8)
Supplement: Supplementary file 8 — Reporting Summary [file 41467_2024_46384_MOESM8_ESM.pdf]

Corresponding author(s): Zaher ElBeck

Last updated by author(s): Feb 4, 2024

Reporting Summary

Nature Portfolio wishes to improve the reproducibility of the work that we publish. This form provides structure for consistency and transparency in reporting. For further information on Nature Portfolio policies, see our Editorial Policies and the Editorial Policy Checklist.

Statistics

For all statistical analyses, confirm that the following items are present in the figure legend, table legend, main text, or Methods section.

n/a	Confirmed	
☐	☒	The exact sample size (*n*) for each experimental group/condition, given as a discrete number and unit of measurement
☐	☒	A statement on whether measurements were taken from distinct samples or whether the same sample was measured repeatedly
☐	☒	The statistical test(s) used AND whether they are one- or two-sided<br>*Only common tests should be described solely by name; describe more complex techniques in the Methods section.*
☒	☐	A description of all covariates tested
☐	☒	A description of any assumptions or corrections, such as tests of normality and adjustment for multiple comparisons
☐	☒	A full description of the statistical parameters including central tendency (e.g. means) or other basic estimates (e.g. regression coefficient) AND variation (e.g. standard deviation) or associated estimates of uncertainty (e.g. confidence intervals)
☐	☒	For null hypothesis testing, the test statistic (e.g. *F*, *t*, *r*) with confidence intervals, effect sizes, degrees of freedom and *P* value noted<br>*Give P values as exact values whenever suitable.*
☒	☐	For Bayesian analysis, information on the choice of priors and Markov chain Monte Carlo settings
☒	☐	For hierarchical and complex designs, identification of the appropriate level for tests and full reporting of outcomes
☐	☒	Estimates of effect sizes (e.g. Cohen's *d*, Pearson's *r*), indicating how they were calculated

Our web collection on statistics for biologists contains articles on many of the points above.

Software and code

Policy information about availability of computer code

Data collection

Ultrasound Vevo 3100 System was used for echocardiographic data collection. .

Ultimate™ 3000 UPLC coupled with a heated electrospray ion source to an Orbitrap™ Fusion Lumos™ tribrid mass spectrometer (ThermoFisher Scientific) was utilized for mass spectrometric data collection.

Bioanalyzer (2100, Agilent) for analysing the quality of the extracted RNA.

Qubit 3.0 Fluorometer (v3, ThermoFisher Scientific) for estimating the concentration of extracted RNA.

S Plan Fluor Ph2 ELWD 60x/0.70 objective on a Nikon Ti2 microscope equipped with a CREST Optics V3 spinning disk confocal was used for acquiring fluorescence images.
Hitachi HT 7700 (Tokyo, Japan) was utilized for transmission electron microscopy data collection.

Illumina NextSeq 550 / Illumina HiSeq3000 were used for sequencing.
CFX384 Touch™ Real-Time PCR Detection System (BioRad). was used for qPCR.

SpectraMax® i3 plate reader (Molecular Devices, US) was used for all spectrophotometric data collection.

ChemiDoc™ gel imaging system (BioRad) were used for imaging of Western Blotting membranes.

XCell SureLock Mini-Cell Electrophoresis System (Invitrogen).

No software was used to collect data.

Data analysis

Excel (Microsoft 365, v 2312).

Echocardiographic data were analyzed using Vevolab software (version 5.5.0).

Skyline (v20.1.0.155) (MacCoss Lab, Dept. of Genome Sciences, University of Washington) was used for mass spectrometric data analysis.

Florescence images were analyzed for the size and diameter of the cells with a custom script written in Fiji (2.9.0). The code is deposited at Github and it can be accessed through this link (https://github.com/BIIFSweden/CellGeometryProfiling).

For RNA sequencing; sequenced reads in fastq files were quality controlled and read counts were derived with the bcbio pipeline (v 1.1.7-b). Reads were aligned to the mouse genome mm10 or to the rat genome Rnor_6.0 using STAR (v 2.6.1d) with genome annotation from ENSEMBL (version 91). Counts were calculated using featureCounts (v 1.6.4). Differential gene expression analysis was performed with DESeq2 (R package v 1.26.0).

For epigenetic analysis of BS and OxBS publicly available data, we utilized codes written in R. The codes are deposited at Github, and it can be accessed through this link (https://github.com/HSiga/BSseq2). Using R (v.3.6), CytosineReports files were normalized by using methylKit library (v1.12.0), MLML2R in (R package v0.3.3) was used to estimate 5mC, 5hmC and non-methylated C at every CpG.

QIAGEN Ingenuity Pathway Analysis (QIAGEN IPA) (IPA Winter Release, December 2023)
QPCR data was analyzed with Bio-Rad CFX Manager 3.1 (3.1.1517.0823).

Intestines of Western blotting bands were analyzed with Image Lab (v6.1, BioRad), and Excel.

All statistical analyses were done using GraphPad Prism (v.8).

SoftMax Pro 7 to analyse all spectrophotometric data.

Visualized images in Fig 5e and supplementary Fig. 5d were prepared using OMERO (v5.5)

For manuscripts utilizing custom algorithms or software that are central to the research but not yet described in published literature, software must be made available to editors and reviewers. We strongly encourage code deposition in a community repository (e.g. GitHub). See the Nature Portfolio guidelines for submitting code & software for further information.

Data

Policy information about availability of data

All manuscripts must include a data availability statement. This statement should provide the following information, where applicable:

- Accession codes, unique identifiers, or web links for publicly available datasets
- A description of any restrictions on data availability
- For clinical datasets or third party data, please ensure that the statement adheres to our policy

The data supporting conclusions drawn in this study are accessible within the article itself and the Supplementary Information provided. The sequencing data generated in this study have been deposited in the Sequence Read Archive (SRA) under accession numbers ¨PRJNA821374 [https://dataview.ncbi.nlm.nih.gov/object/PRJNA821374]¨ for the in vivo materials, and ¨PRJNA773866 [https://dataview.ncbi.nlm.nih.gov/object/PRJNA773866]¨ for the in vitro materials. All quantified raw images are available on BioImage Archive ¨Accession number S-BIAD1033 [https://www.ebi.ac.uk/biostudies/bioimages/studies/S-BIAD1033]¨. Processed sequencing data and the densitograms for the quantification of Western blotting images are provided in Supplementary Data files 1-4. Further information and reasonable requests for resources and reagents should be directed to the lead contact Zaher ElBeck (zaher.elbeck@ki.se). Source data are provided with this paper.

The codes used for BS and OxBS analysis is available through this link ¨DOI: 10.5281/zenodo.10632409 [https://github.com/HSiga/BSseq2]¨. Whereas the codes used for image analysis is available through this link ¨DOI: 10.5281/zenodo.10631363) [https://github.com/BIIFSweden/CellGeometryProfiling¨

Research involving human participants, their data, or biological material

Policy information about studies with human participants or human data. See also policy information about sex, gender (identity/presentation), and sexual orientation and race, ethnicity and racism.

Reporting on sex and gender	The study involved only ex-planted heart samples from human patients and matched controls. The sex of all individuals is provided in Supplementary table 1.<br>hiPSCs were generated from a control donor, healthy, female, 52 years (Borchert, T. et al 2017).
Reporting on race, ethnicity, or other socially relevant groupings	Not applicable.
Population characteristics	Information about each sample regarding age, mutated gene, clinical notes is provided in supplementary table S1.
Recruitment	Samples were obtained from Sydney Heart Bank.
Ethics oversight	The use of human samples was approved by the local ethics committee in Stockholm (2015/559-31/2) and Human Research

Ethics oversight	Ethics Committees at the University of Sydney (2016/7326), and St Vincent's Hospital (H03/118). All procedures conducted in this study on hiPSC-CMs adhered to the principles outlined in the Declaration of Helsinki and received approval from the local ethics committee of the University Medicine of Göttingen (Az-10/9/15). Informed consent was signed by all tissue donors.

Note that full information on the approval of the study protocol must also be provided in the manuscript.

Field-specific reporting

Please select the one below that is the best fit for your research. If you are not sure, read the appropriate sections before making your selection.

☒ Life sciences ☐ Behavioural & social sciences ☐ Ecological, evolutionary & environmental sciences

For a reference copy of the document with all sections, see nature.com/documents/nr-reporting-summary-flat.pdf

Life sciences study design

All studies must disclose on these points even when the disclosure is negative.

Sample size	The sample size in the in vivo study using AZ925 was determined based on our previous experience and the results obtained from a pilot study using this compound. We estimated that 6 animals per group would be sufficient to observe a significant improvement in the cardiac function of male homozygous Mlp-knockout mice if exogenous antioxidant administration could enhance their cardiac function. These numbers were derived from our previous experience with in vivo studies using this murine model of heart failure (Grote Beverborg et al., 2021, Nature Communications, DOI: /10.1038/s41467-021-25439-0). Conversely, considering previous observations related to redox characteristics, it was anticipated that cardiac function in female mice would not be enhanced by exogenous antioxidant treatment. The same reasoning was also utilized to estimate the sample size of animals utilized for other in vivo studies and subsequent ex vivo experiments, with having a minimum of 6 animals per group. In our in vitro studies, we typically conduct 3-6 technical replicates, depending on the accuracy of the assay, and according also to our previous experience (Grote Beverborg et al., 2021, Nature Communications, DOI: /10.1038/s41467-021-25439-0), in which significant changes can be consistently reproduced and are often biologically meaningful with such sample size.
Data exclusions	No data points in any of the in vivo, in vitro, ex vivo studies were excluded.
Replication	When relevant, experiments were replicated a minimum of two or more times, either as technical replicates, by analyzing another set of biological samples, or by employing two different techniques, such as qPCR and RNA sequencing, or by deriving data from various cell types or models using the same experimental settings.. The resulting data demonstrated a consistent trend similar to the representative results presented. A description of the number of experimental replicates is provided in the Source Data file for certain figures.<br>Regarding the in vivo trial involving the Nrf2 activator, a preliminary trial was conducted with a limited sample size, followed by a larger-scale trial. Nrf2 activation was notably observed in both studies. However, the impact on cardiac function was solely evaluated in the larger trial, in which male Mlp-/- showed a significant improvement, whereas females did not exhibit any tendency of improvement, but rather exhibited the opposite effect. Consequently, due to ethical considerations, this experiment was not repeated.
Randomization	Randomization was implemented for all in vivo studies, considering both sex and body weight as criteria. For all other experiments, samples were randomly assigned to experimental groups.
Blinding	The experimenter in the in vivo study was blinded during all procedures. In all other experimenters the experimenter were blinded only during sample processing, but not during instrumental measurements, as the research materials involved different treatments and genotypes, which needed to be independently labelled to avoid confusion with each other.

Reporting for specific materials, systems and methods

We require information from authors about some types of materials, experimental systems and methods used in many studies. Here, indicate whether each material, system or method listed is relevant to your study. If you are not sure if a list item applies to your research, read the appropriate section before selecting a response.

Materials & experimental systems

n/a	Involved in the study	
☐	☒	Antibodies
☐	☒	Eukaryotic cell lines
☒	☐	Palaeontology and archaeology
☐	☒	Animals and other organisms
☐	☒	Clinical data
☒	☐	Dual use research of concern
☒	☐	Plants

Methods

n/a	Involved in the study	
☒	☐	ChIP-seq
☒	☐	Flow cytometry
☒	☐	MRI-based neuroimaging

Antibodies

Antibodies used	Western blotting:

Antibodies used

OxPhos cocktail (Invitrogen #45-8099, Dilution 1:500): C-I-20 (ND6) (clone) 20E9DH10C12 - Bovine Complex I, C-II-30 (FeS) (clone) 21A11AE7 - Bovine Heart Complex II, C-III-Core 2 (clone) 13G12AF12BB11 - Bovine Heart Complex II, III, C-IV-I (clone) 1D6E1A8 - Human Complex IV subunit I and C-V-alpha (clone) 15H4C4 - Bovine Complex V.

IDH2 (Invitrogen #MA5-17271, Dilution 1:1000): Clone (GT673), monoclonal.

GAPDH (Invitrogen #MA5-15738, Dilution 1:2500): Clone (GA1R), monoclonal.

MLP/CSRP3 (Abcam #ab155538, Dilution 1:2000): The exact immunogen sequence used to generate this antibody is proprietary information, polyclonal.

Succinyllysine (PTM Biolabs #PTM-401, Dilution 1:500), polyclonal.

L2HGDH (Proteintech #15707-1-AP, Dilution 1:200), polyclonal.

β-actin (Sigma-Aldrich #A5441, Dilution 1:5000), monoclonal.

SDHA (Cell Signalling #5839, Dilution 1:1000), the immunogen is a synthetic peptide corresponding to residues near the carboxy terminus of human SDHA protein, polyclonal.

HMOX1 (Proteintech #10701-1-AP, Dilution 1:500), immunogen (HMOX1 fusion protein Ag1190), polyclonal.

NQO1 (Proteintech #11451-1-AP, Dilution 1:500), immunogen (NQO1 fusion protein Ag2009), polyclonal.

OSGIN1 (Proteintech #15248-1-AP, Dilution 1:500), immunogen (OSGIN1 fusion protein Ag4295), polyclonal.

NRF2 (Proteintech #16396-1-AP, Dilution 1:1500), immunogen (NRF2 fusion protein Ag9489), polyclonal.

Caspase 3 (Cell Signalling #9662, Dilution 1:1000), immunogen (synthetic peptide corresponding to residues surrounding the cleavage site of human caspase-3), polyclonal.

Secondary anti-rabbit IgG (Sigma-Aldrich #NA934, Dilution 1:5000), peroxidase conjugate, F(ab')2 fragment of affinity isolated antibody.

Secondary anti-mouse IgG (Sigma-Aldrich #A9044, Dilution 1:5000), peroxidase conjugate,
IgG fraction of antiserum.

Antibodies against Vdac, Ndufb8 and Cox4-1 were a kind gift from a kind gift from Peter Rehling, Göttingen. The antibodies were self-made, and published in Poerschke et al., Molecular Cell, 2023.

Immunofluorescence:
α-actinin (#A7811, Sigma-Aldrich, 1:1000): clone EA-53, monoclonal.

Goat anti-mouse, Alexa Fluor 568-conjugated antibody (#ab175473, Abcam, 1:1000), polyclonal.

Validation

All antibodies used in this study were obtained from commercial sources (except for antibodies against Vdac, Ndufb8 and Cox4-1). The validation for the antibodies can be found below in the links to the manufacturing companies. Prior to use, the antibodies were optimized to ensure that the migratory molecular weight of the target protein matched the weight reported by the manufacturer and UniProt.

OxPhos cocktail (Invitrogen #45-8099): https://www.thermofisher.com/antibody/product/OxPhos-Rodent-WB-Antibody-clone-Cocktail-Cocktail/45-8099

IDH2 (Invitrogen #MA5-17271): https://www.thermofisher.com/antibody/product/IDH2-Antibody-clone-GT673-Monoclonal/MA5-17271

GAPDH (Invitrogen #MA5-15738): https://www.thermofisher.com/antibody/product/GAPDH-Loading-Control-Antibody-clone-GA1R-Monoclonal/MA5-15738

MLP/CSRP3 (Abcam #ab155538): https://www.abcam.com/en-se/products/primary-antibodies/csrp3-antibody-ab155538

Succinyllysine (PTM Biolabs #PTM-401): https://ptmbio.com/products/anti-succinyllysine-rabbit-pab/PTM-401.htm

L2HGDH (Proteintech #15707-1-AP): https://www.ptglab.com/products/L2HGDH-Antibody-15707-1-AP.htm

β-actin (Sigma-Aldrich #A5441): https://www.sigmaaldrich.com/SE/en/product/sigma/a5441

SDHA (Cell Signalling #5839, Dilution 1:1000): https://www.cellsignal.com/products/primary-antibodies/sdha-antibody/5839

HMOX1 (Proteintech #10701-1-AP): https://www.ptglab.com/products/HMOX1-Antibody-10701-1-AP.htm

NQO1 (Proteintech #11451-1-AP): https://www.ptglab.com/products/NQO1-Antibody-11451-1-AP.htm

OSGIN1 (Proteintech #15248-1-AP): https://www.ptglab.com/products/OSGIN1-Antibody-15248-1-AP.htm

NRF2 (Proteintech #16396-1-AP): https://www.ptglab.com/products/NFE2L2,NRF2-Antibody-16396-1-AP.htm

Caspase 3 (Cell Signalling #9662): https://www.cellsignal.com/products/primary-antibodies/caspase-3-antibody/9662

Secondary anti-rabbit IgG (Sigma-Aldrich #NA934): https://www.sigmaaldrich.com/SE/en/product/sigma/gena9341ml
Secondary anti-mouse IgG (Sigma-Aldrich #A9044): https://www.sigmaaldrich.com/SE/en/product/sigma/a9044?utm_source=google&utm_medium=cpc&utm_campaign=8939553830&utm_content=100673246340&gclid=CjwKCAiA8YyuBhBSEiwA5R3-EzcIeLh47OJ0AlmPWkyhZvW7FnYUv36Gh5PULeTOKfxeZkuVF9uLGRoCZu8QAvD_BwE

Immunofluorescence:
α-actinin (#A7811, Sigma-Aldrich): https://www.sigmaaldrich.com/SE/en/product/sigma/a7732?utm_source=google&utm_medium=cpc&utm_campaign=8939553962&utm_content=97228929004&gclid=CjwKCAiA8YyuBhBSEiwA5R3-E5L89H2Vs_cPllnsgi84oCT-cGiQXwohdVv86e4HxUq3OZuBwIUCLBoCBDYQAvD_BwE
Goat anti-mouse, Alexa Fluor 568-conjugated antibody (#ab175473, Abcam): https://www.abcam.com/en-se/products/secondary-antibodies/goat-mouse-igg-h-l-alexa-fluor-568-ab175473

When the pattern of expression did not match the expected regulation, and extra validation step were performed. Validation of the L2HGDH antibody was conducted in neonatal rat cardiomyocytes after knocking down L2hgdh using multiple titers of ShRNA, as is described ElBeck et al., 2023 (http://hdl.handle.net/10616/48793), and also shown in Figure 3g.
The correct molecular weight of NRF2 (?95–110 kDa) was adapted from Lau et al., 2013, published in Antioxidants & Redox Signaling, and was further validated by activating NRF2 in hFF1 cells with sulforaphane (supplementary Fig. 4e), and by activating Nrf2 in vivo with with Keap1 inhibitor (AZ925) (supplementary Fig. 4e).
The correct band that correspond to OSGIN1 was validated by activating NRF2 in hFF1 cells with sulforaphane (supplementary Fig. 4e).

Eukaryotic cell lines

Policy information about cell lines and Sex and Gender in Research

Cell line source(s): Neonatal rat cardiomyocytes were primary cells isolated from rats pups at the age of 3 days.
Human foreskin fibroblast cell line (hFF1, #SCRC-1041™) was obtained from ATCC through a collaborator. Human ventricular induced pluripotent stem cell-derived cardiomyocytes (hiPS-CM) generated from a healthy control female donor.

Authentication: We observed the morphology of the cells and monitored their general behavior throughout the culture period.

Mycoplasma contamination: A mycoplasma test was regularly conducted in the lab, and ensured that all utilized cell lines were free from mycoplasma contamination.

Commonly misidentified lines (See ICLAC register): No commonly misidentified lines were used.

Animals and other research organisms

Policy information about studies involving animals; ARRIVE guidelines recommended for reporting animal research, and Sex and Gender in Research

Laboratory animals: The background of muscle lim protein-deficient mice (Mlp-/-), also known as cysteine and glycine-rich protein 3 (Csrp3-/-), is a hybrid cross of the original 129/Sv background with the C57BL/6N strain. The mice were bred in-house for multiple generations. Heterozygous males and females were bred to obtain both wild-type (WT) and Mlp-/- animals. Both male and female mice aged 10-14 weeks were used for experiments. In cases where animals of other ages were used, detailed descriptions can be found in the figure legends. Animals were housed on a 12 h light/12 h dark cycle with free access to chow and water, in 45–65% humidity and ambient temperature at 20–24°C.
Rats from Sprague Dawley strain were purchased from Charles River Laboratories and bred in-house to obtain neonatal pups, which were dissected at the age of 3 days to harvest their hearts.

Wild animals: The study did not involve wild animals.

Reporting on sex: Sex differences were carefully considered in the study, and the sex of the samples is clearly indicated in all relevant experiments, both within the figures and in the Source data file.

Field-collected samples: The study did not involve field-collected samples.

Ethics oversight: Animal experiments were performed in accordance with European ethical regulation (Directive 2010/63/EU) and approved by local animal ethics committee of Linköping (permit numbers S43-15, 1369 and 2713) and Göteborg (permit 1852-2018) in Sweden, and the responsible government agency of Unterfranken (RUF-55.2.2-2532-2-659) in Germany. Animals were housed on a 12 h light/12 h dark cycle with free access to chow and water. All animal experiments were performed in accordance to the ARRIVE guidelines.

Note that full information on the approval of the study protocol must also be provided in the manuscript.

Clinical data

Policy information about clinical studies

All manuscripts should comply with the ICMJE guidelines for publication of clinical research and a completed CONSORT checklist must be included with all submissions.

Clinical trial registration	The study did not involve clinical trial.
Study protocol	Not applicable.
Data collection	Not applicable.
Outcomes	Not applicable.

Plants

Seed stocks	The study did not involve plants.
Novel plant genotypes	Not applicable.
Authentication	Not applicable.
